# Understanding the selectivity of nonsteroidal anti-inflammatory drugs for cyclooxygenases using quantum crystallography and electrostatic interaction energy

**DOI:** 10.1107/S2052252525000053

**Published:** 2025-01-30

**Authors:** S. Pawlędzio, M. Ziemniak, X. Wang, K. Woźniak, M. Malinska

**Affiliations:** a Neutron Scattering Division Oak Ridge National Laboratory Oak Ridge TN37831 USA; b Faculty of Chemistry, Biological and Chemical Research Centre University of Warsaw 02-093Warsaw Poland; Universidad de Oviedo, Spain

**Keywords:** multipole model, NSAIDs, ibuprofen, flurbiprofen, meloxicam, celecoxib, protein crystallography, exact potential/multipole model, EPMM

## Abstract

Quantum crystallography methods have been employed to investigate complex formation between nonsteroidal anti-inflammatory drugs (NSAIDs) and cyclooxygenase (COX) enzymes, with particular focus on the COX-1 and COX-2 isoforms. This study analyzed the electrostatic interaction energies of selected NSAIDs (flurbiprofen, ibuprofen, meloxicam and celecoxib) with the active sites of COX-1 and COX-2, revealing significant differences in binding profiles. Flurbiprofen exhibited the strongest interactions with both COX-1 and COX-2, indicating its potent binding affinity. Celecoxib and meloxicam showed a preference for COX-2, consistent with their known selectivity for this isoform, while ibuprofen showed comparable interaction energies with both isoforms, reflecting its nonselective inhibition pattern. Key amino-acid residues, including Arg120, Arg/His513 and Tyr355, were identified as critical determinants of NSAID selectivity and binding affinity. The findings highlight the complex interplay between interaction energy and selectivity, suggesting that while electrostatic interactions play a fundamental role, additional factors such as enzyme dynamics and the hydrophobic effect also contribute to the therapeutic efficacy and safety profiles of NSAIDs. These insights provide valuable guidance for the rational design of NSAIDs with enhanced therapeutic benefits and minimized adverse effects.

## Abstract 2

This study employs quantum crystallography to elucidate the selectivity of nonsteroidal anti-inflammatory drugs (NSAIDs), including ibuprofen, flurbiprofen, meloxicam and celecoxib, for cyclooxygenase-1 and cyclooxygenase-2 enzymes by analyzing binding energy and electrostatic interactions. The findings reveal key structural determinants of NSAID selectivity, providing valuable insights for the rational design of safer and more effective anti-inflammatory drugs.

## 1. Introduction

Nonsteroidal anti-inflammatory drugs (NSAIDs) are widely used for their analgesic, anti-inflammatory and antipyretic effects (Ghlichloo & Gerriets, 2023 ▸). They exert their effects by inhibiting cyclooxygenases (COXs), which are enzymes involved in the synthesis of prostaglandins (Vane & Botting, 1998 ▸). However, NSAIDs have been associated with various adverse effects, such as gastrointestinal complications including duodenal ulcers, cardiovascular diseases and musculoskeletal pathology (Collen & Abdulian, 1996 ▸; Tenenbaum, 1999 ▸; Roth, 1995 ▸), especially when used on a chronic basis (Collen & Abdulian, 1996 ▸; Tenenbaum, 1999 ▸). NSAIDs are associated with significant health concerns, including cardiovascular toxicity. Studies have linked NSAID use to an increased risk of mortality in patients with chronic heart failure and myocardial infarction (Lai *et al.*, 2019 ▸; Pereira-Leite *et al.*, 2021 ▸). Gastrointestinal complications are another major concern, particularly in older populations where multimorbidity and polypharmacy elevate the risk of adverse drug events (Gnjidic *et al.*, 2014 ▸; Tielemans *et al.*, 2014 ▸). At a molecular level, NSAIDs can affect ion channels and are implicated in hyperalgesia mediated by spinal glutamate or substance P receptors (Malmberg & Yaksh, 1992 ▸; Gwanyanya *et al.*, 2012 ▸). While NSAIDs have shown potential chemopreventive effects in certain cancers, their diverse adverse effects necessitate careful consideration before use (Ayub & Islam, 2015 ▸).

**Figure 1:**
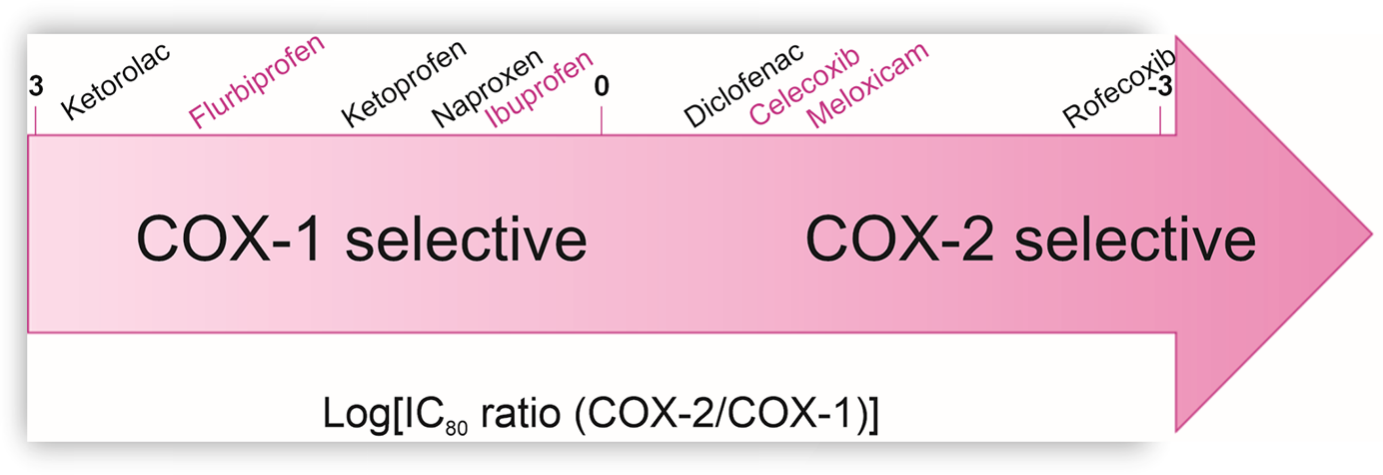
Relative degree of COX-1 and COX-2 selectivity. This figure is reproduced according to Warner & Mitchell (2004 ▸). Inhibitors in this study are marked in pink.

NSAIDs act by inhibiting COX enzymatic activity, blocking the conversion of arachidonic acid to prostaglandins (PGs; Tegeder *et al.*, 2001 ▸), thereby producing anti-inflammatory and analgesic effects. There are at least four isoforms of the COX enzyme, but COX-1 and COX-2 are the major targets of NSAIDs, including aspirin (Vane & Botting, 2003 ▸), ibuprofen (Orlando *et al.*, 2015 ▸) and newer COX-2 inhibitors (Hermanson *et al.*, 2014 ▸). COX-1 is active under physiological conditions in many organs, including the kidneys, blood vessels and stomach (Brune & Patrignani, 2015 ▸), as well as in blood components. The activity of the second isoform, COX-2, increases rapidly in tissues affected by the inflammatory process (Vane & Botting, 1998 ▸). Importantly, NSAIDs differ in their specificity of action towards these two cyclooxygenase isoforms (Fig. 1 ▸). From a kinetic standpoint, aspirin, which covalently modifies Ser530 in the cyclooxygenase active site, is an irreversible and time-dependent inhibitor. Ibuprofen, in contrast, is a rapidly reversible inhibitor with moderate potency (IC_50_ values in the micromolar range), while indomethacin and flurbiprofen represent intermediate cases as time-dependent, noncovalent inhibitors.

Classic NSAIDs such as ibuprofen or aspirin are active against both the COX-1 and COX-2 isoforms (Mitchell *et al.*, 1993 ▸; Gilroy *et al.*, 1998 ▸; Basson *et al.*, 2001 ▸). Importantly, the mechanism of action of aspirin differs fundamentally from that of other NSAIDs, as it causes the irreversible inactivation of COX-1 and COX-2, preventing the oxidative conversion of arachidonic acid to PGG_2_ and PGH_2_ (Brune & Patrignani, 2015 ▸), which plays a significant role in the development of inflammatory symptoms. Inhibition of COX-2 activity, and thus reduced prostaglandin synthesis, underlies the analgesic and anti-inflammatory effects of some NSAIDs. In contrast, the antithrombotic mechanism of action relies on platelet COX-1 inhibition, which leads to suppression of thromboxane A2 synthesis and a subsequent reduction in platelet aggregation. Thus, inhibiting the physiological activity of COX-1 may lead to side effects associated with NSAID use, such as the development of gastric lesions and renal toxicity (Masferrer *et al.*, 1994 ▸). Therefore, new NSAID drugs that are selective only for COX-2 have been developed (Funk & FitzGerald, 2007 ▸; Penning *et al.*, 1997 ▸; Habeeb *et al.*, 2001 ▸; McGettigan & Henry, 2006 ▸).

The interactions between ligands and COX-1/COX-2 are crucial in understanding the mechanisms of action of various compounds, including NSAIDs. The binding of ligands to COX-1 and COX-2 involves complex intermolecular inter­actions within the active sites, including hydrogen bonding, hydrophobic interactions and specific contacts with amino-acid residues (Krzyżak *et al.*, 2020 ▸; Xu *et al.*, 2014 ▸; Rowlinson *et al.*, 2003 ▸). For instance, it has been observed that inhibitors form hydrogen bonds with specific amino-acid residues inside the active sites of both COX-1 and COX-2, indicating the importance of these interactions in ligand binding (Krzyżak *et al.*, 2020 ▸). Many inhibitors contain carboxylic groups that, similarly to the carboxylate of arachidonic acid, establish polar interactions with Arg120 or Tyr355 near the substrate entrance to the binding cavity. Additionally, the binding between the ligand and the active site of COX-2 can lead to conformational changes of the COX-2 protein, further emphasizing the significance of these interactions (Tuwalaid *et al.*, 2022 ▸). A number of distinctive binding modes of inhibitors in the COX active site have been described, including ion pairing of the carboxylic group of an inhibitor with specific amino-acid residues as well as the insertion of arylsulfon­amides or sulfones into the COX-2 side pocket (Rowlinson *et al.*, 2003 ▸). These interactions are essential for understanding the selectivity and mechanism of action of the COX inhibitors. Additionally, the binding energies and key amino-acid residues involved in the binding of flavonoids (including flavones, flavonols, anthocyanidins and isoflavonols) to both COX-1 and COX-2 have been revealed (Perez *et al.*, 2019 ▸).

A comprehensive understanding of protein–ligand inter­action energies is essential for predicting binding affinities and stability. Various theoretical methods and approaches have been developed to estimate these interaction energies, such as the linear interaction energy (LIE) approach, which utilizes interaction energies with adjustable parameters to estimate protein–ligand binding free energies (Duan *et al.*, 2016 ▸). In addition to computational approaches, experimental charge-density analysis based on multipolar refinement has been proposed as a powerful tool for evaluating the nature and strength of intermolecular interactions in protein–ligand complexes from X-ray crystallography (Coppens, 1997 ▸). An experimental charge-density distribution was determined for meloxicam hydrate, showcasing its utility in understanding the detailed electrostatic properties and hydrogen-bonding patterns within pharmaceutical molecules (Devi Rajendran *et al.*, 2018 ▸). However, the data resolution required for such studies should be at least 0.5 Å, which is still very challenging for protein–ligand complexes. Fortunately, it has been shown that the multipole atomic parameters from the Hansen–Coppens multipole model are transferable between molecules if a similar chemical environment is provided (Brock *et al.*, 1991 ▸). Dominiak *et al.* (2007 ▸) developed a theoretical databank of transferable aspherical pseudoatoms, called UBDB, and demonstrated its application to electrostatic interaction energy calculations. The combination of UBDB with the exact potential/multipole model (EPMM) method has made it possible to obtain interaction energies of high accuracy for a wide range of intermolecular distances and interaction types (Bojarowski *et al.*, 2022 ▸). Hence, this approach offers a comprehensive resource for evaluating electrostatic inter­actions in protein complexes (Malińska *et al.*, 2014 ▸, 2015 ▸; Li *et al.*, 2006 ▸; Dominiak *et al.*, 2009 ▸; Czyżnikowska *et al.*, 2010 ▸; Budniak *et al.*, 2022 ▸). UBDB+EPMM is also applicable in the computation of the electrostatic energies of hydrogen-bonded nucleic acid–base complexes, despite potential underestimation when compared with *ab initio* results (Czyżnikowska *et al.*, 2010 ▸). When applied to predict interaction energies between phosphate groups and amino-acid residues, UBDB+EPMM revealed notable improvements compared with traditional force-field point charges (Budniak *et al.*, 2022 ▸). Expanding beyond single protein–ligand interactions, this approach has been extended to analyze monomer–monomer interactions in multimeric proteins, providing a broader context for understanding protein–ligand dynamics (Kumar & Dominiak, 2021 ▸). A key advantage of using UBDB in X-ray crystal structure refinement, particularly at subatomic or atomic resolution, is its ability to produce *X*–H distances that are closer to those observed in neutron diffraction data. In X-ray diffraction refinement using the independent-atom model, H atoms are often positioned inaccurately due to spherical atom assumptions, but UBDB can improve these distances at suitable resolutions (Malinska & Dauter, 2016 ▸; Guillot *et al.*, 2001 ▸, 2008 ▸; Takaba *et al.*, 2019 ▸). Firstly, in terms of atom number, hydrogens constitute approximately half of the structure of a protein (Engler *et al.*, 2003 ▸). Secondly, many of these H atoms participate in critical hydrogen bonds, particularly in protein active centers, where precise knowledge of their positions is essential for understanding processes such as proton-transfer reactions (Kossiakoff & Spencer, 1981 ▸).

The selectivity of NSAIDs between COX-1 and COX-2 is a critical factor in their therapeutic potential and safety. Rofecoxib, marketed by Merck & Co. under the brand name Vioxx, is a notable example of a COX-2-selective nonsteroidal anti-inflammatory drug developed to treat conditions such as osteoarthritis, rheumatoid arthritis, acute pain and migraines. While initially hailed for its selective inhibition of COX-2, thus avoiding many of the gastrointestinal side effects associated with COX-1 inhibition (Vane & Botting, 1998 ▸), its high COX-2 selectivity ultimately contributed to adverse cardiovascular effects (Jüni *et al.*, 2004 ▸; Solomon *et al.*, 2005 ▸). This led to its withdrawal from the market, highlighting that highly elevated COX-2 selectivity can carry significant risks.

**Figure 2:**
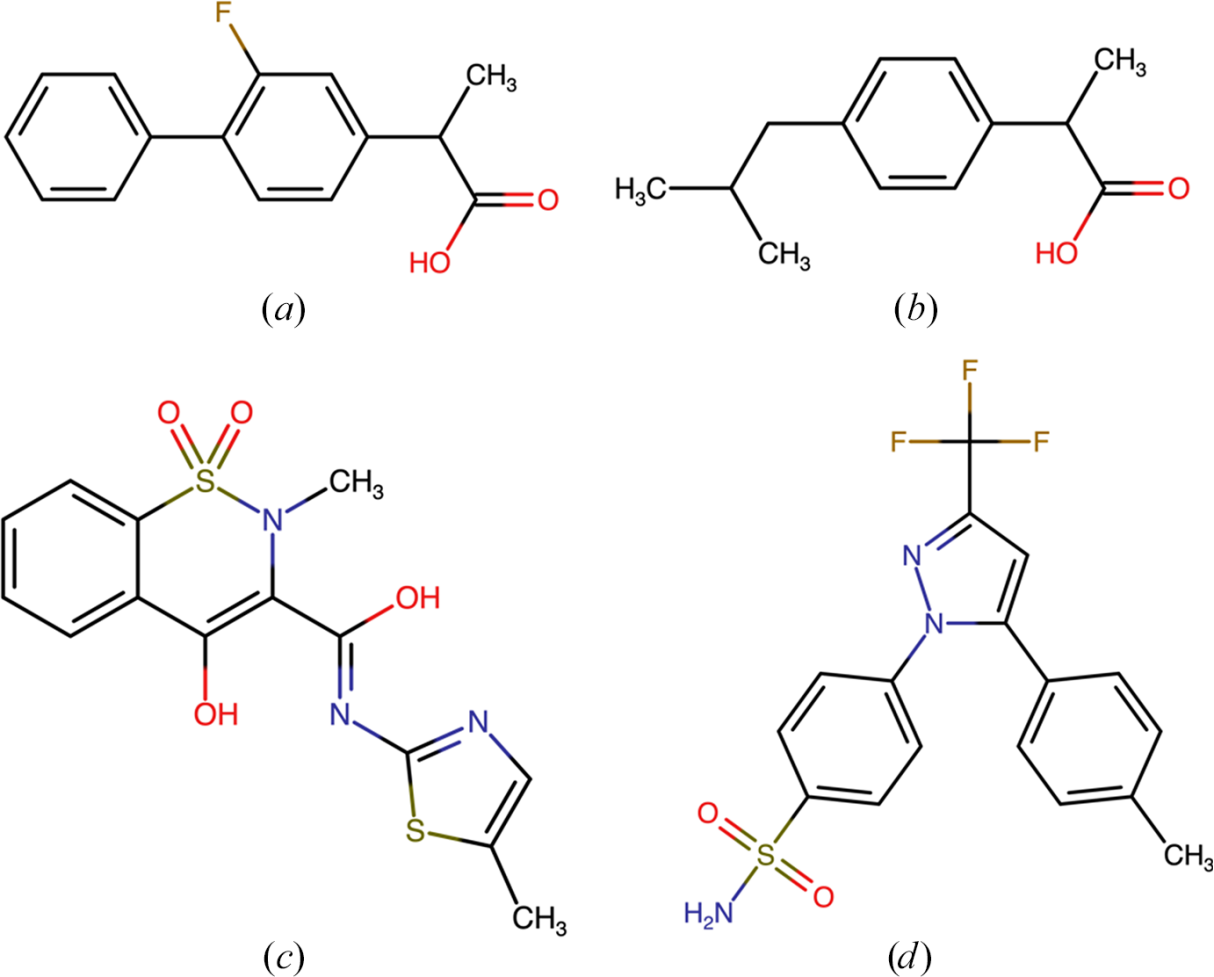
Chemical structures of the selected inhibitors: (*a*) flurbiprofen, (*b*) ibuprofen, (*c*) meloxicam and (*d*) celecoxib.

Numerous efforts to develop COX-2-selective NSAIDs coupled with computational studies predicting selectivity profiles demonstrate the importance of isoform selectivity in drug development and therapeutic outcomes (Aktar *et al.*, 2016 ▸; Parambil *et al.*, 2020 ▸). However, challenges such as variability in reported IC_50_ values, reliance on moderate-resolution structural data and the absence of direct entropic contributions in binding models highlight the need for rigorous, integrative approaches. This study employs quantum crystallography methods to analyze the interaction dynamics between NSAIDs and COX isoforms (COX-1 and COX-2). This analysis primarily focuses on comparative electrostatic interaction energy (*E*_es_) calculations to elucidate the isoform-specific binding characteristics of selected NSAIDs. These inhibitors are shown in Fig. 2 ▸.

## 2. Methods

### 2.1. Protein structure preparations

All calculations for the COX-1/COX-2–NSAID complexes (Selinsky *et al.*, 2001 ▸; Orlando *et al.*, 2015 ▸; Kurumbail *et al.*, 1996 ▸; Xu *et al.*, 2014 ▸; Rimon *et al.*, 2010 ▸; Wang *et al.*, 2010 ▸) were performed using the structures deposited in the RCSB Protein Data Bank (Berman *et al.*, 2000 ▸) as presented in Table 1 ▸. All files have been carefully checked, and for the sake of proper calculations some changes have been made.

The side chain in PDB entry 3kk6 (Rimon *et al.*, 2010 ▸) was originally incomplete and thus was corrected into all-atom protein using the Schrödinger *Protein Preparation Wizard* (Madhavi Sastry *et al.*, 2013 ▸; Schrödinger Release 2021-4: Protein Preparation Wizard). Hydrogen atoms were absent in all deposited structures. Therefore, for all studied COX-1/COX-2–NSAID complexes we added H atoms with *Mol­Probity* (Williams *et al.*, 2018 ▸) and positioned them based on the X-ray suitable electron cloud distances (Williams *et al.*, 2018 ▸). Arg, Lys, Asp and Glu residues were treated as ionized. All possible flips proposed by the program were accepted. After adding H atoms, molecules of water, other organic compounds and ions as well as heme cofactors were removed and the structures were split into the respective numbers of chains.

### 2.2. Electrostatic interaction calculations

To reconstruct the electron-density distribution in the investigated complexes we used UBDB (Jarzembska & Dominiak, 2012 ▸) and transferred multipole parameters (Su & Coppens, 1992 ▸) for the atom types using *LSDB* (Volkov *et al.*, 2004 ▸). Two missing N and S atom types for the meloxicam ligand were calculated based on the structures deposited in the Cambridge Structural Database (CSD; Groom *et al.*, 2016 ▸) and were added to the databank to enable the analysis. All H-atom positions were extended to the values obtained from neutron studies as reported by Allen & Bruno (2010 ▸). After the transfer of electron-density parameters from UBDB (Jarzembska & Dominiak, 2012 ▸), all amino-acid residues of COX-1/COX-2 proteins and NSAID molecules were scaled independently according to their formal charges, with celecoxib and meloxicam scaled to 0 and ibuprofen and flurbiprofen scaled to −1. The corresponding electrostatic interaction energies were calculated for every pair of amino-acid and NSAID molecules using the EPMM method as implemented in the *XDPROP* module available in the *XD*2016 software (Volkov *et al.*, 2016 ▸).

### 2.3. Binding-pocket definition

For further analysis, we compared the proximal binding pocket defined by residues Arg120, Val349, Ser353, Tyr355, Ile523, Ala527 and His513/Arg513, the central binding pocket, defined by Tyr348, Leu352, Leu384, Tyr385, Trp387, Gly526, Ser530, Phe381, Phe518 and Met522, and the distal binding pocket, defined by Phe205, Phe209, Val228, Val344, Ile377, Gly533 and Leu534. In PDB entries 4o1z (Xu *et al.*, 2014 ▸) and 4m11 (Xu *et al.*, 2014 ▸) the inhibitors were disordered into two distinct conformations. In our study, we have accounted for both conformations and treated them independently. To include the conformational variability of the protein structures, after the computation of *E*_es_ for each chain we calculated the average and standard error of the mean (SEM). The error for the residues forming the binding pocket was calculated as the square root of the sum of the squared errors (RSE) for each residue. The following structures were included in the analysis: PDB entries 3kk6 (two chains), 3ln1 (two chains), 1eqh (two chains), 3pgh (three chains), 1eqg (two chains), 4ph9 (two chains), 4oz1 (two chains and two meloxicam conformations) and 4m11 (four chains). One chain in 4m11 was excluded from averaging due to an incorrect conformation of the meloxicam amidosulfonyl group in chain *C*.

## 3. Results

### 3.1. Comparison of COX-1 and COX-2

**Figure 3:**
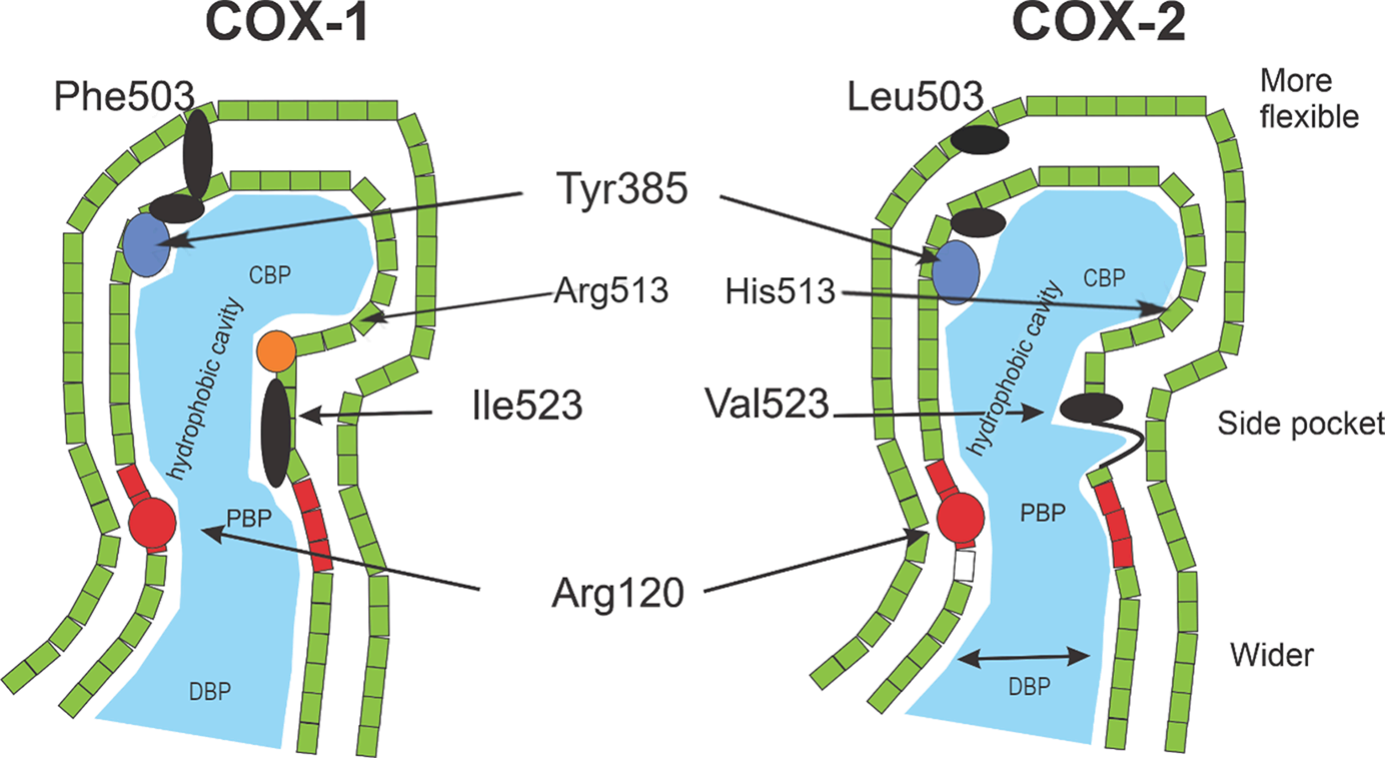
Schematic representation of the active site of COX-1 and COX-2. CBD, PBP and DBP denote the central binding pocket, proximal binding pocket and distal binding pocket, respectively. This figure was reproduced from *The Lancet* (Hawkey, 1999 ▸) with permission from Elsevier.

The crystal structures of COX-1 and COX-2 are well known in the literature (Browner, 1996 ▸; Miciaccia *et al.*, 2021 ▸). They display high homology, with a sequence identity of almost 60% and a similarity score of 84% (Blobaum & Marnett, 2007 ▸). Both isoforms contain three domains: (i) an EGF-like region responsible for protein folding, (ii) a membrane-binding domain and (iii) a catalytic domain. The membrane-binding domain is associated with hydrophobic residues, which create a long and narrow substrate channel leading to the active site (Fig. 3 ▸). Despite sharing high homology, COX-1 and COX-2 exhibit significant functional differences due to some structural heterogeneity. The formation of ionic interactions in the constriction of the proximal binding pocket is less important for the potency of inhibition of COX-2 compared to COX-1 as Arg120 and Glu524 form a salt bridge in COX-2 (Vecchio *et al.*, 2010 ▸). Table 2 ▸ also highlights the importance of electrostatic interactions between Arg120 and Glu524 in determining the selectivity profiles for these NSAIDs, with COX-2-selective inhibitors exhibiting stronger interactions in COX-2 and COX-1-selective inhibitors exhibiting stronger interactions in COX-1. Notably, ibuprofen shows very similar interaction energies between the two enzymes, which aligns with its known nonselective inhibition of both COX-1 and COX-2. In contrast, flurbiprofen exhibits significantly stronger interaction energy for COX-1 compared to COX-2, underscoring its higher selectivity for COX-1. Both celecoxib and meloxicam exhibit stronger interaction energies with Arg120 and Glu524 in COX-2 relative to COX-1, consistent with their experimentally observed COX-2 selectivity. These findings further emphasize the selectivity profiles of these NSAIDs, with celecoxib and meloxicam favoring COX-2, ibuprofen being nonselective and flurbiprofen favoring COX-1. Most inhibitors extend into the central binding pocket, establishing hydrophobic contacts with residues such as Leu352, Leu384, Tyr385, Trp387, Phe381, Phe518, Met522, Gly526 or Ser530. In some cases, polar interactions are established with Ser530 or Tyr385. One of the hallmark differences between COX-1 and COX-2 is the substitution of cyclooxygenase-channel residues Ile434, His513 and Ile523 in COX-1 with Val434, Arg513 and Val523, respectively, in COX-2. The substitution of Ile434 with Val434 is particularly significant, as the smaller Val434 residue creates an accessible side pocket adjacent to the COX-2 active site that is inaccessible in COX-1. Additionally, the positively charged guanidinium group of Arg513 enhances its ability to participate in hydrogen-bonding and electrostatic interactions within a water-mediated binding network involving His90, Tyr355 and Glu524. This network stabilizes the constriction at the entrance of the COX-2 active site, facilitating greater accessibility for certain inhibitors. The pocket, lined by residues such as His90, Gln192, Leu352, Ser353, Tyr355, Arg513, Ala516, Phe518 and Val523, enhances the binding affinity of COX-2 for selective inhibitors. Celecoxib, for example, interacts with Arg513 within this side pocket, exploiting the additional binding space for increased specificity.

### 3.2. Interaction of flurbiprofen with COX-1 and COX-2

**Figure 4:**
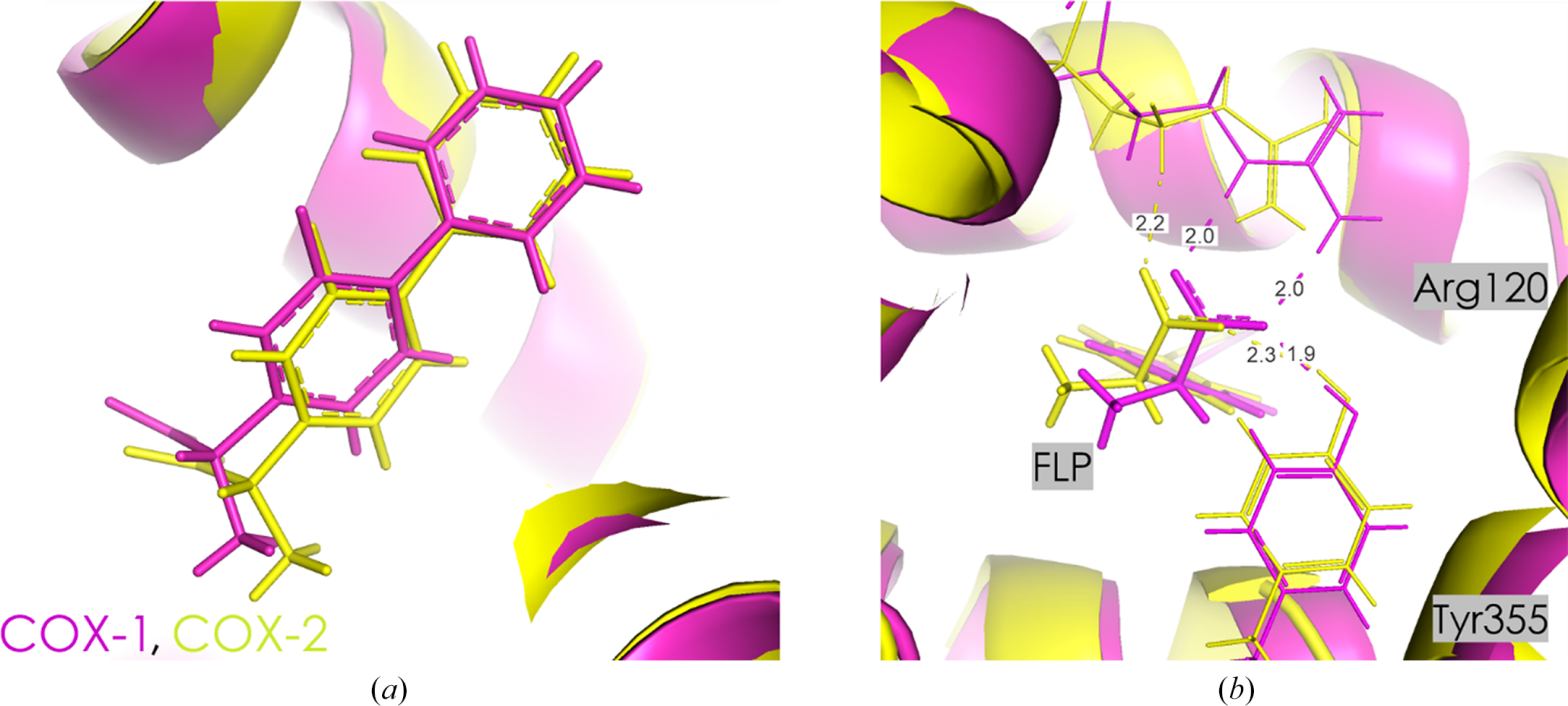
(*a*) FLP in the active site of COX-1 (pink) and COX-2 (yellow). (*b*) Orientation of Arg120 and Tyr355 in COX-1 and COX-2. Contact distances are shown in Å.

**Figure 5:**
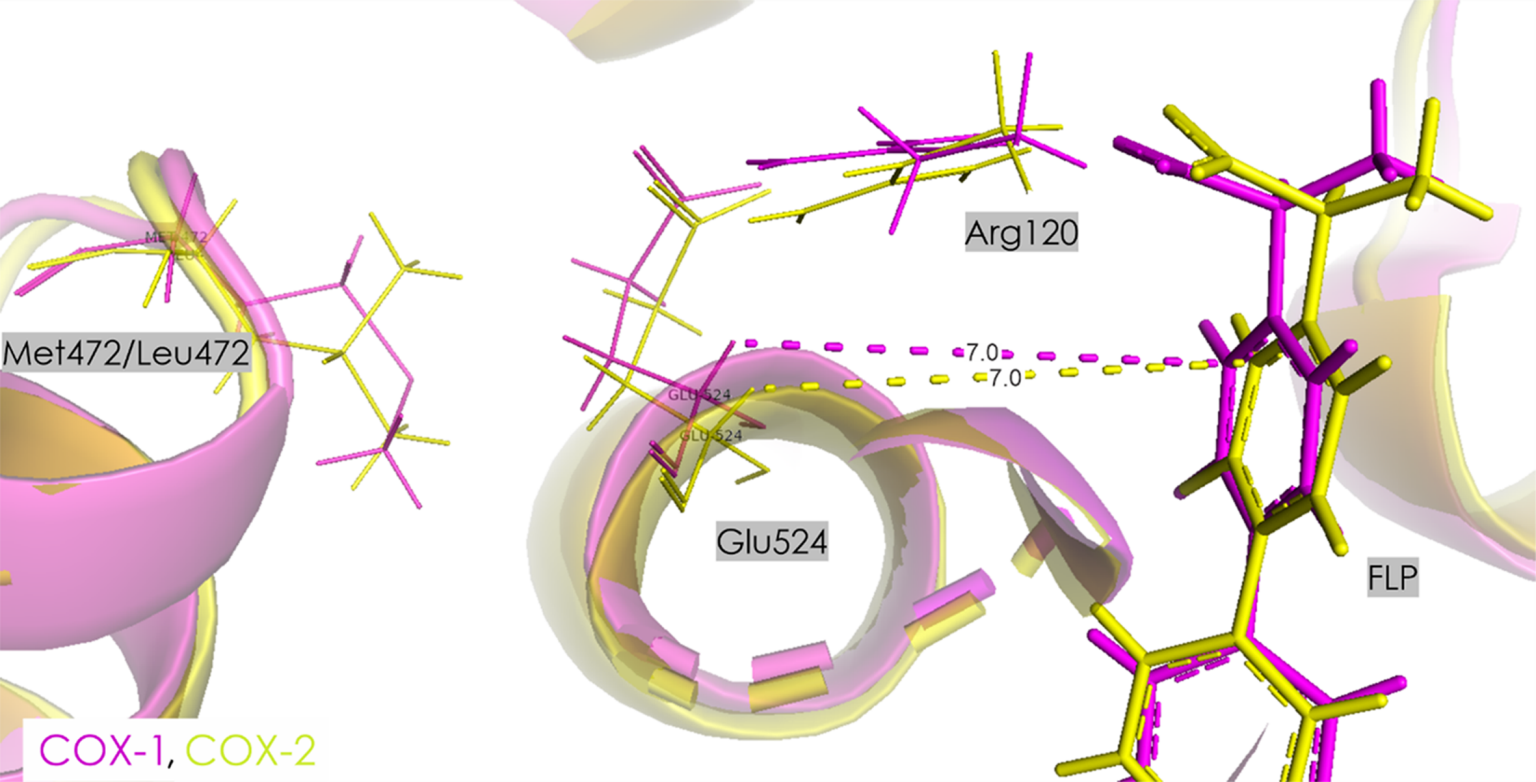
Orientation of Glu524, Arg120 and Met/Leu472 in the active pocket of COX-1 (pink) and COX-2 (yellow) with the FLP ligand. Contact distances are shown in Å.

Flurbiprofen (FLP) is generally recognized as a nonselective inhibitor of COX, effectively blocking both the COX-1 and COX-2 enzymes (Cryer & Feldman, 1998 ▸). However, some studies have reported it to have greater selectivity for COX-1. This discrepancy is likely to arise from differences in the experimental conditions, assay methods or the biological systems used to evaluate selectivity (Fig. 4 ▸). The binding site of flurbiprofen in both COXs is very similar; however, some minor changes in the orientations of amino acids are visible. The carboxylic group of flurbiprofen interacts with two residues, forming a salt bridge to Arg120 and a hydrogen bond to Tyr355. The orientation of Arg120 in COX-1 is different from that in COX-2 [Figs. 4 ▸(*b*) and 5 ▸], which is reflected in the differing strengths of the inhibitor–Arg120 interactions (Table 3 ▸). Similarly, the orientation of Tyr355 varies slightly between the two enzymes, resulting in inhibitor–Tyr355 interactions of different strengths (Table 3 ▸). These dissimilarities in orientation can explain the reduced inhibitor–Arg120 interaction energy in COX-1 compared with COX-2, which may suggest charge dilution among the carboxylic, guanidino and phenol groups in COX-1. Interestingly, the electrostatic energy between glutamic acid at position 524 and flurbiprofen is markedly different for COX-1 and COX-2 (50.2 ± 0.7 and 199.2 ± 5 kJ mol^−1^, respectively). This difference is primarily due to the interactions between Glu524 and Arg120, which are strongly related to the orientation of Arg120 (Fig. 5 ▸). Additionally, the substitution of Met472 in COX-1 by Leu472 in COX-2 might play a key role in the strength of the Glu524–FLP interaction (Fig. 5 ▸).

The FLP–protein complex is stabilized by several non­covalent interactions, such as C—H⋯π (for example Met522) and C—H⋯O (for example Leu531) contacts and, interestingly the electrostatic contribution to these interactions differs significantly between COX-1 and COX-2. For example, the electrostatic interaction energy of FLP with Leu359 and Leu531 is much more negative for COX-2 than for COX-1, despite their similar spatial orientations. A similar difference in electrostatic contribution is observed with Met522, with interaction energies of −10.2 ± 0.5 kJ mol^−1^ for COX-1 and 8.1 ± 4.4 kJ mol^−1^ for COX-2. Additionally, it was found that the electrostatic interaction between FLP and Ser530 has a stabilizing character (−18.9 ± 3.2 kJ mol^−1^) only in the case of COX-2, owing to O—H⋯π contacts.

It is worth noting that certain substitutions, such as those of Ile523 in COX-1 by valine in COX-2, of His513 in COX-1 by arginine in COX-2 and of Leu357 in COX-1 by phenyl­alanine in COX-2, result in different strengths of the intermolecular interactions between the inhibitor and the enzyme (Supplementary Tables S1–S3). The His513-to-Arg513 sub­stitution in COX-2 is critical, as the longer side chain and the positively charged guanidinium group of arginine enable stronger long-range electrostatic interactions that can stabilize binding within the active-site environment. This substitution enhances the binding affinity in COX-2, even for inhibitors such as flurbiprofen that do not form direct contacts with Arg513.

In the proximal binding pocket, FLP interacts very strongly with Arg120 in both COX-1 and COX-2, with interaction energies of −210.1 ± 15.5 and −377.6 ± 26.8 kJ mol^−1^, respectively (Table 3 ▸). The interaction with Tyr355 is also significant, with energies of −79.3 ± 6.6 kJ mol^−1^ in COX-1 and −91.3 ± 23.8 kJ mol^−1^ in COX-2. The total interaction energy in this pocket is much more negative for COX-2 (−659.1 ± 38.7 kJ mol^−1^) compared with COX-1 (−310.9 ± 17.1 kJ mol^−1^), indicating a stronger and more stable interaction in COX-2. This difference is largely driven by the much stronger interaction of FLP with Arg120 in COX-2.

In the central binding pocket, COX-1 exhibits a relatively weak overall interaction energy of −10.4 ± 6.7 kJ mol^−1^. The interactions with Tyr385 (−16.8 ± 1.9 kJ mol^−1^) and Met522 (−10.2 ± 0.5 kJ mol^−1^) are the most significant contributors in COX-1. In contrast, COX-2 shows a stronger total interaction energy of −51.6 ± 29.0 kJ mol^−1^ in this pocket, with notable contributions from Ser530 (−18.9 ± 3.2 kJ mol^−1^) and Trp387 (−16.5 ± 5.3 kJ mol^−1^). This indicates that the central binding pocket in COX-2 provides a more stabilizing environment for FLP compared with COX-1. In the distal binding pocket, COX-1 shows a total interaction energy of 2.3 ± 14.6 kJ mol^−1^, showing almost no electrostatic interaction with this part of the binding pocket. COX-2, however, exhibits a slightly more stabilizing interaction energy of −14.0 ± 0.7 kJ mol^−1^, with contributions from Leu534 (−8.1 ± 0.5 kJ mol^−1^) and a destabilizing effect from Val344 (10.1 ± 0.2 kJ mol^−1^). This suggests that while both isoforms show weak interaction energies in this pocket, COX-2 exhibits a higher overall affinity. Analysis of cumulative interaction energies reveals important differences between COX-1 and COX-2. In the proximal binding pocket, COX-2 shows a significantly more negative total energy (−659.1 ± 38.7 kJ mol^−1^) compared with COX-1 (−310.9 ± 17.1 kJ mol^−1^), highlighting a much stronger overall interaction for FLP in COX-2. In the central binding pocket, COX-2 again shows a stronger interaction, with a cumulative energy of −51.6 ± 29.0 kJ mol^−1^ compared with −10.4 ± 6.7 kJ mol^−1^ for COX-1. Finally, in the distal binding pocket, COX-2 is slightly more stabilizing with a cumulative energy of −14.0 ± 0.7 kJ mol^−1^, compared with 2.3 ± 14.6 kJ mol^−1^ in COX-1. These differences in the sum values underscore the overall stronger interaction of FLP with COX-2, despite its widely recognized preference for COX-1.

### 3.3. Interaction of ibuprofen with COX-1 and COX-2

**Figure 6:**
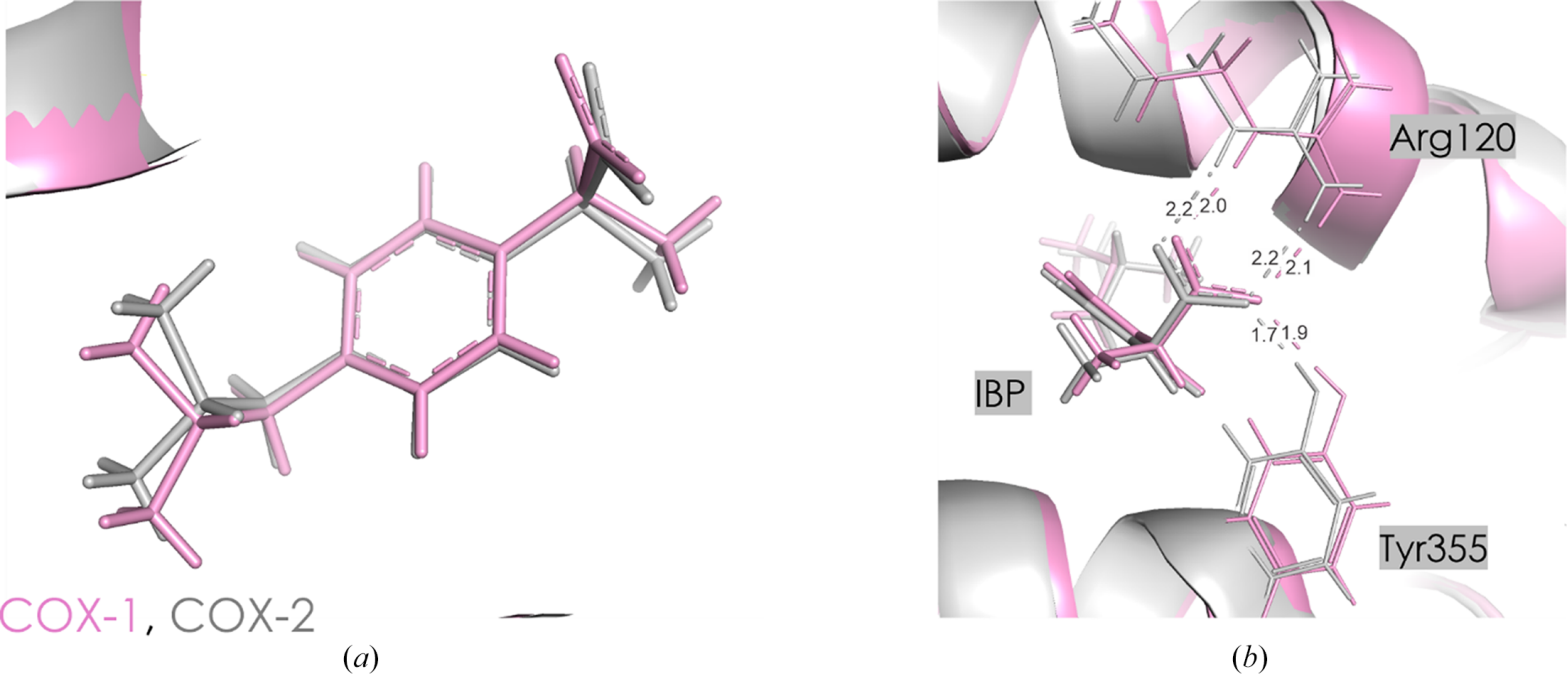
(*a*) IBP in the active site of COX-1 (pink) and COX-2 (gray). (*b*) Orientation of Arg120 and Tyr355 in COX-1 (pink) and COX-2 (gray). Contact distances are shown in Å.

Ibuprofen (IBP) is one of the most popular pharmaceuticals in the world. The anti-inflammatory and analgesic effects of IBP are primarily due to its inhibition of COX-2, although it also exhibits similar IC_50_ inhibition values for COX-1 (Table 2 ▸). IBP occupies the active sites of both COX isoforms in a typical arylpropionic acid orientation and reversibly binds to both COXs, acting as a competitive inhibitor of arachidonic acid. Structural comparisons of IBP bound to COX-1 and COX-2 have shown nearly identical binding modes in COX-1 and COX-2, maintaining the characteristic orientation of arylpropionic acids [Fig. 6 ▸(*a*)].

**Figure 7:**
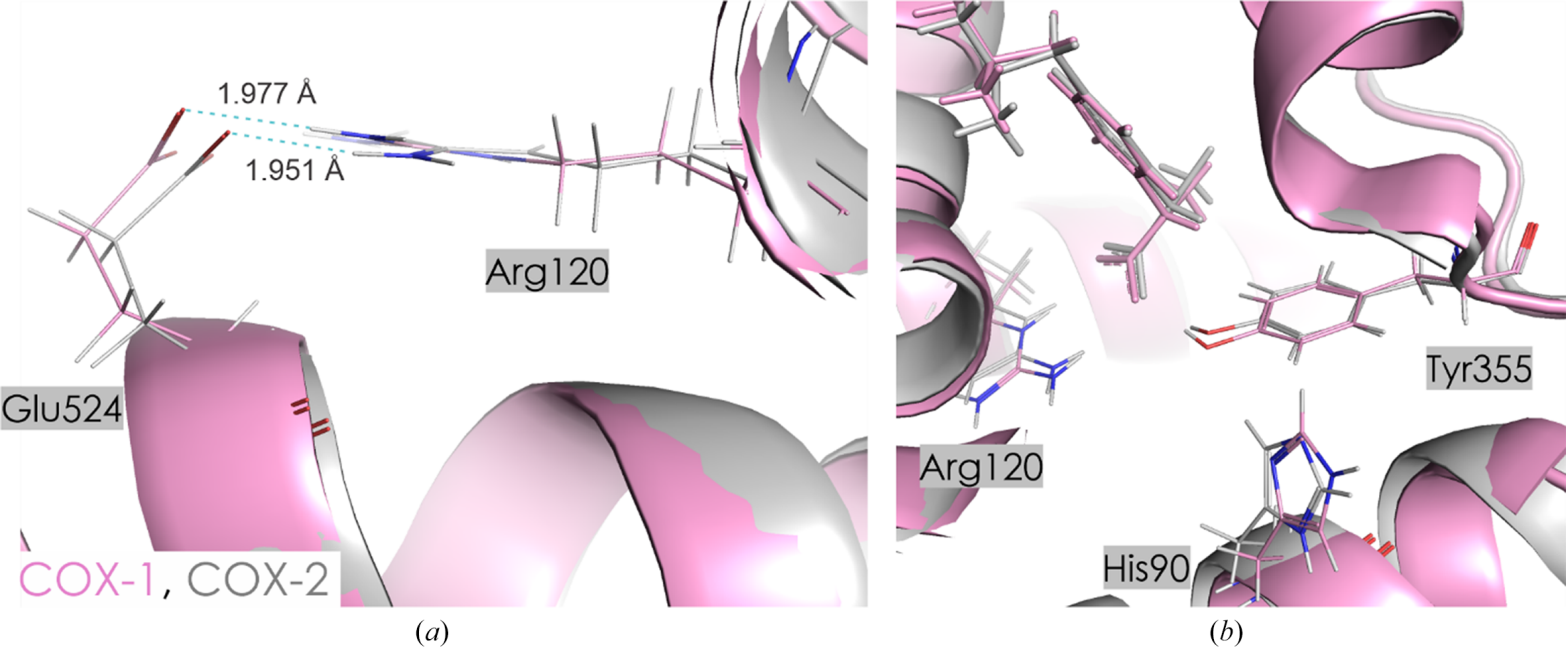
Conformational differences of amino-acid residues in the COX-1 (pink) and COX-2 (gray) active sites with IBP ligand. Contact distances are shown in Å.

Although the orientation of IBP is almost identical in both COX isoforms, the residue-specific interaction energies differ between COX-1 and COX-2. When examining the most negative interaction energies, there is a difference of almost 25 kJ mol^−1^ between COX-1 and COX-2 for both the Arg120–IBP and Tyr355–IBP pairs, despite a lack of significant difference in amino-acid orientations in both enzymes [Fig. 6 ▸(*b*)]. However, these discrepancies primarily arise from subtle variations in the spatial arrangement of active-site residues within the active-site channel. For example, the distance and angle of interaction between the amino group (Arg120) and the carboxylic group (Glu524) differ in COX-1 and COX-2. This results in a slightly different direction of the H atom from the –NH_2_ group (Arg120) towards the –OH group (Glu524), weakening the Arg120–IBP interaction in COX-2 [Fig. 7 ▸(*a*), Table 4 ▸]. On the other hand, the C—H⋯O interaction between IBP and Leu93 is less screened by Tyr355 in the COX-2 complex, resulting in a more stabilizing character of this interaction (Supplementary Tables S4 and S5) than in COX-1. This may also be associated with a different orientation of the neighboring His90, where in COX-2 the −NH group is directed towards Tyr355, while in COX-1 it is oriented away from the active pocket [Fig. 7 ▸(*b*)]. This could alter the distribution of charge [Figs. 6 ▸(*b*), 7 ▸(*a*) and 7 ▸(*b*)].

In the proximal binding pocket, ibuprofen interacts with Arg120 in COX-1 and Arg121 in COX-2, showing interaction energies of −242.1 ± 28.5 and −215.5 ± 44.4 kJ mol^−1^, respectively. The interaction with Tyr355 in COX-1 and Tyr356 in COX-2 is also significant, with energies of −76.8 ± 10.0 and −100.3 ± 7.4 kJ mol^−1^, respectively. The total interaction energy in this pocket is similar between COX-1 and COX-2, with COX-2 having a slightly more negative value at −330.6 ± 30.3 kJ mol^−1^ compared with −337.6 ± 45.1 kJ mol^−1^ for COX-1. These findings suggest that the comparable binding stability of ibuprofen in the proximal binding pockets of both COX isoforms reflects its nonselective inhibition profile. In the central binding pocket, COX-1 exhibits a total interaction energy of 38.0 ± 1.9 kJ mol^−1^, which indicates a destabilizing interaction overall. The interaction with Leu352 is the most significant in COX-1, contributing 26.5 ± 0.1 kJ mol^−1^. In COX-2, the total interaction energy in the central binding pocket is also destabilizing but is slightly higher at 57.6 ± 1.7 kJ mol^−1^, with Leu353 contributing 37.4 ± 0.2 kJ mol^−1^. This indicates that ibuprofen encounters a less favorable energetic environment in the central binding pocket of COX-2 relative to COX-1. In the distal binding pocket, ibuprofen shows a small positive cumulative energy of 13.8 ± 0.2 kJ mol^−1^ in COX-1, with a major contribution from Leu534 at 10.4 ± 0.1 kJ mol^−1^. Similarly, in COX-2 the total interaction energy is slightly higher at 14.4 ± 0.4 kJ mol^−1^, with Leu535 contributing 11.7 ± 0.4 kJ mol^−1^. The interaction energy profiles indicate comparable binding patterns for ibuprofen in the distal binding pockets of both isoforms, despite slightly destabilizing effects. Analysis of cumulative interaction energies across all binding pockets suggests that ibuprofen interacts almost equally with both COX-1 and COX-2, with only minor differences in specific residues. The proximal binding pocket shows similar stabilizing interactions in both isoforms, while the central and distal pockets exhibit slightly more destabilizing interactions in COX-2. These findings correlate with the known nonselective binding profile of ibuprofen, confirming its lack of COX isoform preference.

### 3.4. Interaction of meloxicam with COX-1 and COX-2

**Figure 8:**
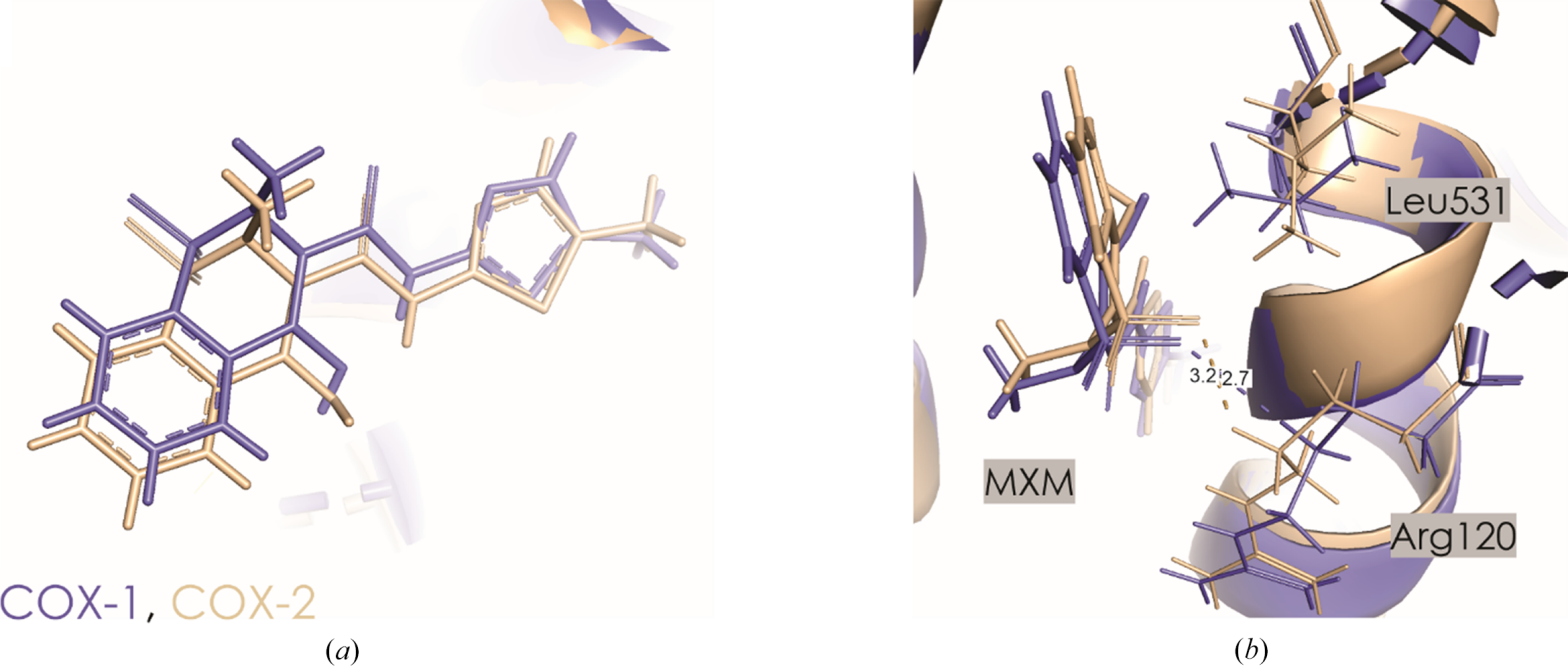
(*a*) MXM in the active site of COX-1 (purple) and COX-2 (beige). (*b*) Orientation of Arg120 and Leu531 in COX-1 (purple) and COX-2 (beige). Contact distances are shown in Å.

Meloxicam (MXM) is considered to be moderately selective for COX-2 *in vitro* (Table 2 ▸). Fig. 8 ▸(*a*) shows the differences in the orientation of the drug in the active pocket of COX-1 and COX-2, which are mostly located around the thiazole ring.

**Figure 9:**
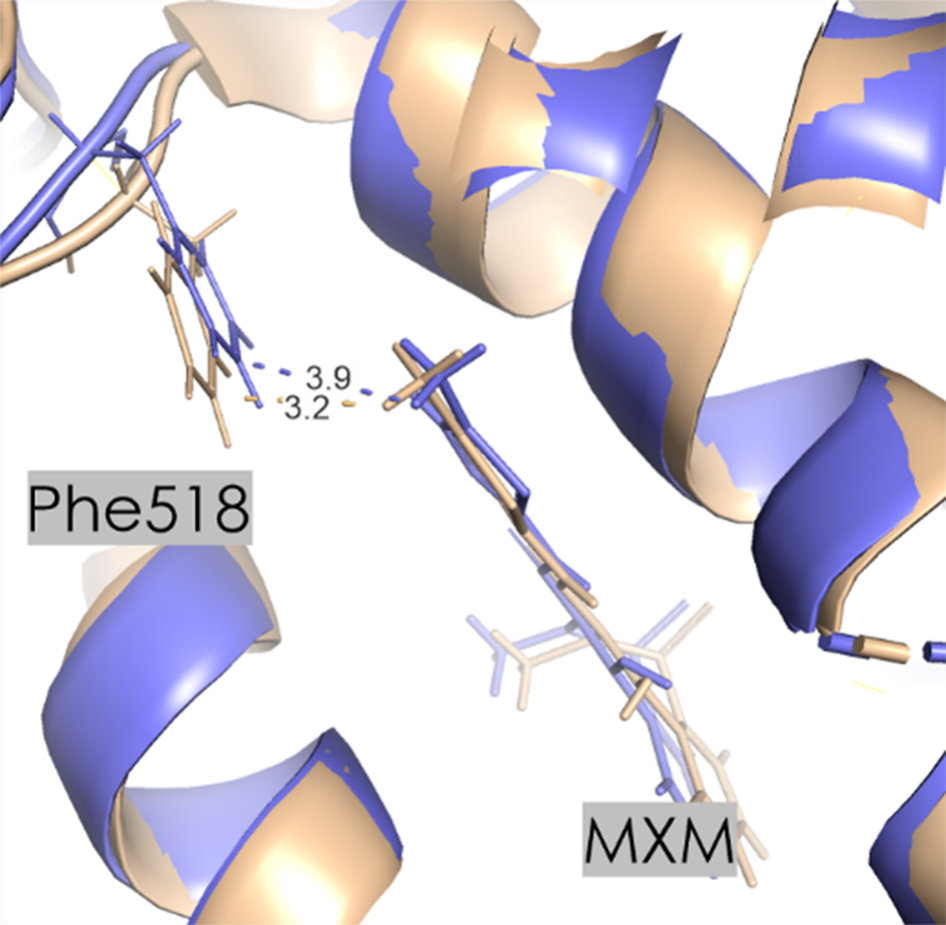
Changes in the Phe18 orientation in COX-1 (purple) and COX-2 (beige). The distance between MXM and Phe518 is longer in COX-1. Contact distances are shown in Å.

The selectivity of MXM is not caused by an Ile523-to-Val523 substitution in COX-2, which governs the selectivity of other inhibitors including rofecoxib and celecoxib (Xu *et al.*, 2014 ▸). Instead, it is attributed to structural changes near Phe518 caused by the Ile434-to-Val 434 substitution in COX-2. This substitution in COX-1 forces Phe518 into the active site, restricting space near the thiazole ring of meloxicam (Fig. 9 ▸).

The different orientation of Phe518 in COX-1 and COX-2 was first noted with the COX-2 inhibitor SC-558 (Sai Ram *et al.*, 2006 ▸). While SC-558 binding relies on protein flexibility, the selectivity of meloxicam seems to involve a direct interaction with Phe518. Site-directed mutagenesis studies confirmed that subtle structural variations around Phe518 influence COX-2 selectivity across different inhibitor classes (Xu *et al.*, 2014 ▸).

However, our calculations present a different picture (Table 5 ▸). Among the most stabilizing interactions, those involving Arg120 and Leu531 provide the most favorable contribution [Supplementary Tables S6–S8 and Fig. 8 ▸(*b*)]. Additionally, the substitution of His513 in COX-1 with Arg in COX-2 plays an important role in the moderate selectivity of meloxicam for COX-2. Arg513 can engage in stronger electrostatic and hydrogen-bonding interactions compared with His513, potentially increasing the binding stability of meloxicam in COX-2. The orientation of Leu531 also differs between the isoforms, which, along with Arg513, contributes to the observed selectivity favoring COX-2. On the other hand, Phe518 shows *E*_es_ interaction energies of −3.0 ± 7.7 and −3.1 ± 1.7 kJ mol^−1^ for COX-1 and COX-2, a relatively small difference (Table 5 ▸). This suggests that selectivity is not primarily driven by electrostatic forces in this case, but rather is driven by dispersion forces and other interactions. It implies that interaction with Leu531 emerges as a key determinant of selectivity based on interaction energy calculations (Supplementary Tables S6–S8). The main discrepancy arises from distinct rotameric states of Phe518, which affect interaction energies. One would expect these structural differences to contribute significantly to the energy differences. However, the energy values do not show a significant difference, which suggests that the rotamer variations might not be the sole or the most significant contributors to the selectivity. Other factors, possibly involving additional residue interactions (including Leu531) or overall structural conformations, might also play important roles. These observations indicate the need for comprehensive investigation beyond individual pairwise interactions to fully understand the complete molecular picture of the COX-2 selectivity of meloxicam. Thus, the complete protein environment, not just individual amino-acid interactions, must be considered when interpreting such data to achieve an accurate interpretation of all selectivity determinants. While the interaction energy data support the idea that there are differences in how Phe518 interacts with meloxicam in COX-1 and COX-2, the extent to which these differences contribute to the selectivity of the drug might be more complex than initially suggested by the statement focusing solely on rotamer variations.

In the proximal binding pocket, meloxicam exhibits similar interaction energies with Arg120 in both COX-1 and COX-2, with values of −135.7 ± 41.0 and −140.4 ± 16.5 kJ mol^−1^, respectively. However, the total interaction energy in this pocket is slightly more favorable for COX-2 (−229.4 ± 19.4 kJ mol^−1^) than COX-1 (−204.8 ± 44.9 kJ mol^−1^). In the central binding pocket, the overall interaction energy is similar for both COX-1 (−91.4 ± 54.8 kJ mol^−1^) and COX-2 (−100.0 ± 21.3 kJ mol^−1^). Notably, the interaction with Ser530 is significantly more stabilizing in COX-2 (−80.3 ± 9.7 kJ mol^−1^) than in COX-1 (−47.5 ± 43.3 kJ mol^−1^). The interaction with Phe518, although showing only a small difference, suggests that dispersion forces may play a more important role than electrostatic interactions in this case. In the distal binding pocket, the interaction energies of meloxicam indicate a stabilizing interaction in COX-2 with a total energy of −13.1 ± 6.7 kJ mol^−1^, in contrast to COX-1, which shows a sum of −3.4 ± 27.0 kJ mol^−1^. These differences suggest that the selectivity of meloxicam for COX-2 is likely to result from a combination of factors, including the specific contributions of residues such as Leu531 and the broader structural environment, rather than being driven solely by the electrostatic interactions with Phe518.

### 3.5. Interaction of celecoxib with COX-1 and COX-2

**Figure 10:**
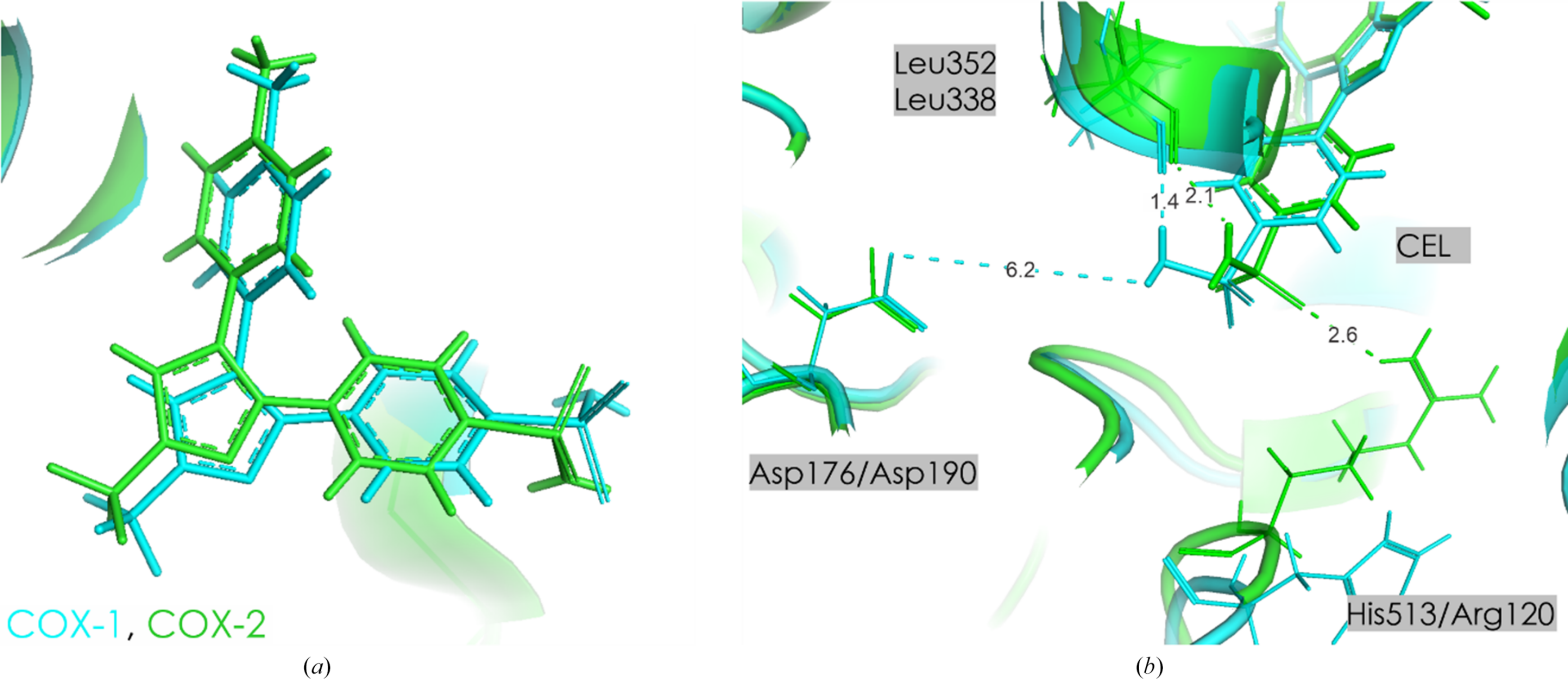
(*a*) CEL in the active site of COX-1 (blue) and COX-2 (green). (*b*) Orientation of Arg120/His513, Leu352/338 and Asp176/190 in COX-1 and COX-2. Contact distances are shown in Å.

Celecoxib (CEL) is a selective COX-2 inhibitor which interacts with both COX-1 and COX-2 (Fig. 10 ▸). The IC_50_ of celecoxib for COX-1 and COX-2 inhibition has been reported to be 6.7 ± 0.9 and 0.87 ± 0.18 µ*M*, respectively (Grösch *et al.*, 2001 ▸). Additionally, it displays both anti-inflammatory and analgesic properties, indicating its interaction with COX-2 (Smith *et al.*, 1998 ▸).

Table 6 ▸ and Supplementary Tables S9–S11 illustrate the interactions between the drug celecoxib and specific residues in both the COX-1 and COX-2 isoforms. Notably, the residues making the strongest interactions with celecoxib differ between these isoforms, suggesting variations in composition and the spatial arrangements of their binding sites. A key difference is the substitution of His513 in COX-1 with Arg499 in COX-2; the latter residue contributes an interaction energy of −49.3 ± 3.7 kJ mol^−1^ and creates a more favorable environment in the COX-2 binding cavity, which is likely to be a critical factor in the selectivity of celecoxib for COX-2. There are some other differences: Leu352 and Asp190 are critical in COX-1, whereas Arg499 and Leu338 play a notable role in COX-2 (Supplementary Tables S9 and S10). Although some residues are conserved between isoforms, they exhibit different interaction energies with celecoxib. For example, Leu359 interacts with celecoxib in both COX-1 and COX-2, but shows a stronger interaction in COX-1 (−14.0 ± 2.4 kJ mol^−1^) than in COX-2 (−9.3 ± 3.1 kJ mol^−1^) (Supplementary Tables S9 and S10). Some other residues also show isoform-specific interaction strengths (based on COX-1 numbering): Gln192, Leu352 and Leu359 interact more strongly in COX-1, while Ser353 and Tyr355 show stronger interactions in COX-2. These isoform-specific differences in interaction energies provide a molecular basis for the selectivity of celecoxib.

In the proximal binding pocket, CEL shows a clear preference for COX-2 over COX-1. The total electrostatic interaction energy in COX-2 is significantly more stabilizing at −175.2 ± 16.2 kJ mol^−1^ compared to −118.7 ± 43.8 kJ mol^−1^ in COX-1. This stronger interaction in COX-2 is primarily due to key residues such as Arg499, which contributes −49.3 ± 3.7 kJ mol^−1^, Ser339 with −37.9 ± 14.4 kJ mol^−1^ and Tyr341 with −19.0 ± 0.5 kJ mol^−1^. In contrast, the interaction in COX-1 is less stabilizing, with Ile523 contributing the most at −60.2 ± 4.3 kJ mol^−1^. The more stabilizing interaction in COX-2 suggests that the proximal binding pocket plays a crucial role in the selectivity of CEL for COX-2. The central binding pocket shows modest differences in interaction energies between COX-1 and COX-2. In COX-1 the total *E*_es_ is −83.0 ± 59.3 kJ mol^−1^, which reflects a balance between a strong stabilizing contribution from Leu352 at −88.1 ± 52.8 kJ mol^−1^ and destabilization by Leu384, which adds +19.4 ± 12.8 kJ mol^−1^. In COX-2, the total *E*_es_ is slightly less stabilizing (−57.2 ± 31.8 kJ mol^−1^), with Leu338 and Met508 contributing −40.0 ± 27.2 and −13.8 ± 3.9 kJ mol^−1^, respectively. Although COX-1 shows more favorable interactions in this pocket, the energetic difference is less pronounced than in the proximal binding pocket, suggesting that this region plays a smaller role in the selectivity of CEL. The distal binding pocket exhibits a more stabilizing inter­action in COX-2 compared to COX-1, although the differences are smaller. In COX-1, the total *E*_es_ is slightly destabilizing at +17.1 ± 13.7 kJ mol^−1^, largely due to Leu534 contributing +11.8 ± 13.4 kJ mol^−1^. On the other hand, in COX-2 the total *E*_es_ is more stabilizing at +6.6 ± 0.6 kJ mol^−1^, with no significant contributions from individual residues. While this pocket shows a more stabilizing inter­action in COX-2, it is likely to play a secondary role in the selectivity of CEL. Overall, the most pronounced energetic differences between COX-1 and COX-2 occur in the proximal binding pocket, where COX-2 forms stronger interactions with CEL. This suggests that the selectivity of CEL for COX-2 is largely driven by the stronger interactions in this pocket, particularly with residues such as Arg120, Ser339 and Arg499. The central and distal pockets, while contributing to the overall binding, appear to be only secondary determinants of selectivity.

## 4. COX-1 and COX-2 interaction energies

Electrostatic interaction energies contribute to our understanding of the interactions between NSAIDs and the COX-1 and COX-2 enzymes, which can influence both their therapeutic effects and side effects. While the selectivity of NSAIDs towards these enzymes is a key factor in determining their clinical outcomes, it is important to note that electrostatic interactions alone may not directly correlate with selectivity. While selectivity is indeed related to the free energy of binding, it also includes contributions from factors such as solvation changes, which may differ between COX-1 and COX-2 due to variations in their binding-site environments. These differences in solvation and binding dynamics can influence selective inhibition, even with the same ligand (Luong *et al.*, 1996 ▸; Copeland *et al.*, 2006 ▸). Inhibition efficiency is influenced by the specific fit to the active site and inter­actions with certain residues which are critical for the activity of the enzyme. For example, in COX-1 there are only two charged residues in the binding pocket, which limits the contribution of electrostatic interactions compared to COX-2. Additionally, the binding pocket of COX-2 is more flexible than that of COX-1, resulting in a more favorable entropy contribution in COX-2. This entropic factor can offset enthalpic gains, further influencing the free energy of binding and selectivity (Luong *et al.*, 1996 ▸; Copeland *et al.*, 2006 ▸; Duggan *et al.*, 2010 ▸). Thus, while the *E*_es_ between an NSAID molecule and the active sites of either COX-1 or COX-2 can provide useful insights, it should be considered in the context of other factors (Table 7 ▸). The electrostatic component of the enthalpy of binding can be influenced by multiple protonation equilibria, and its physical significance may vary significantly depending on the given system (Piłat & Antosiewicz, 2008 ▸). Studies have shown that electrostatic interactions can make a substantial contribution to the free energy of binding in various molecular complexes, such as avidin–biotin binding (Tong *et al.*, 2010 ▸) and protein–drug interactions (Bitencourt-Ferreira & de Azevedo Junior, 2021 ▸). However, the contributions of different interaction types to the binding energy can vary among protein–ligand complexes. For instance, in the brazilin–hIAPP complex, hydrophobic interactions were found to contribute more significantly to the free energy of binding compared to electrostatic interactions (Guo *et al.*, 2017 ▸). Similarly, in the interactions between protein A and human immunoglobulin G1, the hydrophobic effect dominates the binding free energy, with electrostatic interactions playing only a minor role (Huang *et al.*, 2011 ▸). While electrostatic interactions are a crucial component of the binding free energy, their contribution varies across molecular systems and their interplay with other types of interactions, such as hydrophobic forces and van der Waals interactions, determines the overall binding affinity.

In general, stronger electrostatic interactions suggest higher binding affinity, while weaker interactions imply reduced affinity. However, the relationship between *E*_es_ and the selectivity of an enzyme can be complex. For example, celecoxib has stronger electrostatic interactions with COX-2 (−225.8 ± 35.7 kJ mol^−1^) than with COX-1 (−184.6 ± 75.0 kJ mol^−1^), which correlates with its lower IC_50_ for COX-2 and indicates a selective preference for COX-2. Similarly, meloxicam has a slightly more favorable *E*_es_ for COX-2 (−342.5 ± 29.5 kJ mol^−1^) compared to COX-1 (−299.6 ± 75.8 kJ mol^−1^), aligning with its IC_50_ values and supporting its selectivity for COX-2. On the other hand, ibuprofen exhibits a slightly stronger *E*_es_ for COX-1 (−278.8 ± 30.4 kJ mol^−1^) than for COX-2 (−265.6 ± 45.1 kJ mol^−1^), yet its similar IC_50_ values for both enzymes suggest a lack of strong selectivity, consistent with its non­selective inhibition profile. However, in the case of flurbiprofen, the *E*_es_ for COX-2 (−724.7 ± 48.3 kJ mol^−1^) is significantly stronger than for COX-1 (−318.9 ± 23.5 kJ mol^−1^), yet it has a lower IC_50_ for COX-1 (0.08 µ*M*), indicating a preference for COX-1 compared with COX-2 (5.50 µ*M*). This discrepancy highlights the role of factors beyond *E*_es_, such as enzyme conformational dynamics, solvation and binding-site interactions, in determining selectivity. Additionally, the average energy and conformational changes indicate that celecoxib and meloxicam preferentially stabilize the structure of COX-2, consistent with their experimentally observed COX-2 selectivity. Conversely, flurbiprofen demonstrates greater stabilization of COX-1, as reflected by its selectivity profile.

Quantitative analysis of residue-specific interaction energies between NSAIDs and both COX isoforms (Tables 3 ▸, 4 ▸, 5 ▸ and 6 ▸) shows that certain amino acids emerge as crucial across all ligands. Arg120 in the proximal binding pocket plays a fundamental role in ligand recognition, showing consistently strong interactions across both COX-1 and COX-2. The highly favorable interaction energies of this residue, particularly in COX-2, indicate its significant role in ligand binding. It is exemplified by its robust interactions with flurbiprofen and ibuprofen, underscoring the contribution of Arg120 to the overall binding affinity. Tyr355 and Ser353 also play critical roles in the proximal binding pocket. Tyr355 generally shows negative interaction energies across both COX-1 and COX-2, although the magnitude of these interactions varies depending on the ligand. Ser353 in the proximal pocket also provides a consistent contribution to binding, with interactions that are particularly important in determining the COX-2 selectivity of certain drugs, such as celecoxib and meloxicam.

Arg513 in COX-2 (and the corresponding His513 in COX-1) is another crucial residue that significantly influences drug selectivity. The substitution of His513 in COX-1 with Arg513 in COX-2 introduces a positive charge that can form stronger hydrogen bonds and electrostatic interactions with certain NSAIDs, particularly celecoxib and meloxicam. This substitution also plays a critical role in stabilizing the salt bridge between Arg120 and Glu524 in COX-2, further enhancing the binding affinity and selectivity of these drugs for COX-2. The interaction energies involving Arg513 are notably more stabilizing in COX-2, underscoring its importance for selective binding to COX-2.

Leu352 in the central binding pocket represents another key residue that exhibits significant but variable interaction energies. Its contribution to binding affinity varies among different inhibitors, showing context-dependent favorable or unfavorable effects. This variability suggests that Leu352 plays a role in the selectivity of the drugs between COX-1 and COX-2. In addition, Phe518 in the central binding pocket and Gly533 in the distal binding pocket also play vital roles in binding. Phe518 demonstrates context-dependent favorable interactions that vary with both inhibitor type and COX isoform. Although Gly533 shows modest interaction energies compared with other residues, it still contributes to the binding affinity and specificity, particularly in the distal pocket.

On the other hand, several amino acids make a negative contribution to binding between studied NSAIDs and COX isoforms. Tyr355 in the proximal binding pocket exhibits isoform-specific effects: it shows some destabilizing inter­actions in COX-1, particularly with celecoxib, while showing some stabilizing effects in COX-2. Similarly, Val349 shows positive interaction energies with certain drugs such as celecoxib and ibuprofen in COX-1, indicating a resistance to binding that can affect the selectivity of the drug. In the central binding pocket, Met522 also may have a destabilizing energetic effect, especially in the interaction with celecoxib in COX-1. This suggests that Met522 may reduce the binding affinity of some NSAIDs in this pocket. In certain situations, such as the binding of celecoxib and flurbiprofen to COX-2, Phe381 also displays positive interaction energies in certain contexts, contributing to destabilization in the central pocket. In the distal binding pocket, the destabilizing interactions are more prominent for Val344 and Phe205. These residues frequently show positive interaction energies, particularly in the interaction of ibuprofen and flurbiprofen with COX-1, suggesting that they play a role in weakening the binding in this region.

## 5. Conclusions

The structural differences between COX-1 and COX-2 significantly influence how NSAIDs interact with these enzymes, often manifested in both the amino-acid composition and their spatial arrangement at their active sites. The isoform selectivity of NSAIDs fundamentally determines their therapeutic efficacy and safety profile. COX-1 is constitutively expressed and plays a role in maintaining key physiological functions, including gastric mucosal integrity and platelet aggregation. In contrast, COX-2 is predominantly inducible, serving as a primary mediator of inflammation and pain. Therefore, inhibitors with higher selectivity for COX-2 are desirable for anti-inflammatory and analgesic effects, while minimizing the gastrointestinal and cardiovascular side effects associated with COX-1 inhibition. The case of rofecoxib (Vioxx) exemplifies the importance of this selectivity. Vioxx was initially favored for its COX-2 selectivity and associated reduction in gastrointestinal side effects. However, excessive COX-2 selectivity contributed to increased cardiovascular risks, leading to its market withdrawal, underscoring the complexity and clinical importance of balancing COX-1 and COX-2 inhibition.

Understanding the relationship between *E*_es_ and selectivity can facilitate the rational design of novel NSAIDs with improved therapeutic profiles. By leveraging computational and experimental approaches, researchers can optimize the selectivity of NSAIDs to enhance their clinical benefits while minimizing adverse effects. The EPMM method is a valuable tool for increasing our understanding of the interactions between NSAIDs and COX isoforms. Electrostatic interaction energies can potentially help to explain the selectivity of NSAIDs for COX-1 or COX-2, as a more negative total interaction energy indicates a stronger overall interaction with the enzyme. This energy is a good predictor of binding affinity with COX isoforms in the case of meloxicam, celecoxib and ibuprofen. However, this pattern does not hold universally, as shown by flurbiprofen, indicating that factors beyond interaction energy, such as entropic contributions, can significantly influence drug selectivity. Entropic contributions to COX-2 binding warrant careful consideration, as they can counterbalance enthalpic effects in determining the overall binding free energy. Comprehensive analysis of this enthalpy–entropy compensation across diverse ligand sets could reveal fundamental principles governing the molecular-recognition process.

Importantly, selectivity is not solely determined by the strength of interaction, as was shown by the analysis of *E*_es_ values for Phe518 in meloxicam–COX-1 and meloxicam–COX-2 complexes, where these energies do not necessarily provide a complete explanation of the selectivity profile. Therefore, it is essential to understand the differences between the COX-1 and COX-2 inhibition mechanisms by certain NSAIDs. From an analysis of the influence of stabilization of different conformations represented by different chains in the structure, it can be seen which ligand has a higher selectivity for one of the COX isoforms. Such selectivity is influenced by the ability of the drug to fit into the active site and its interaction with specific residues critical for the activity of the enzyme. Even slight differences in the positions and conformations of these residues in the active site can influence the *E*_es_ and consequently the selectivity and efficacy of NSAIDs. A prominent example is the substitution of His513 in COX-1 with Arg513 in COX-2, a critical modification that significantly alters the binding landscape. Arg513 provides additional opportunities for hydrogen-bonding and electrostatic interactions, enhancing the binding affinity of COX-2-selective inhibitors such as celecoxib. Moreover, compared with His513, Arg513 further stabilizes the salt bridge between Arg120 and Glu524, reinforcing the overall stability of COX-2. Understanding these subtle yet impactful molecular determinants of binding is crucial for rational drug-design strategies facilitating the development of next-generation NSAIDs with optimized isoform selectivity and improved safety profiles.

## Supplementary Material

Detailed electrostatic interaction energy results. DOI: 10.1107/S2052252525000053/pen5007sup1.pdf

## Figures and Tables

**Table 1:** PDB codes for the investigated COX-1/COX-2–NSAID complexes and their X-ray diffraction resolutions

NSAID	Abbreviation	COX	Resolution (Å)	PDB code
Flurbiprofen	FLP	COX-1	2.7	1eqh (Selinsky *et al.*, 2001 ▸)
		COX-2	2.5	3pgh (Kurumbail *et al.*, 1996 ▸)
Ibuprofen	IBP	COX-1	2.6	1eqg (Selinsky *et al.*, 2001 ▸)
		COX-2	1.81	4ph9 (Orlando *et al.*, 2015 ▸)
Celecoxib	CEL	COX-1	2.75	3kk6 (Rimon *et al.*, 2010 ▸)
		COX-2	2.4	3ln1 (Wang *et al.*, 2010 ▸)
Meloxicam	MXM	COX-1	2.4	4o1z (Xu *et al.*, 2014 ▸)
		COX-2	2.45	4m11 (Xu *et al.*, 2014 ▸)

**Table 2:** Average *E*_es_ values calculated between Arg120 and Glu524 for each NSAID in COX-1 and COX-2 chains, along with experimental IC_50_ values from whole blood assays for both COX-1 and COX-2 (Rao & Knaus, 2008 ▸) The shortest N⋯O distances for the N—H⋯O salt bridge/hydrogen bond are also provided for each enzyme chain. Values are reported as means with standard deviations where applicable.

	Whole blood assay IC_50_ (µ*M*)		*E*_es_ (kJ mol^−1^)		Shortest N⋯O distance (Å)	
NSAID	COX-1	COX-2	COX-1	COX-2	COX-1	COX-2
						
Celecoxib	6.70	0.87	−308.9 ± 44.7	−334.3 ± 2.1	3.0 (7)	2.7
Meloxicam	5.70	2.10	−348.1 ± 61.7	−351.1 ± 43.8	3.1 (4)	2.9 (2)
Ibuprofen	7.60	7.20	−379.8 ± 39.0	−394.8 ± 23.8	3.0 (2)	2.85 (7)
Flurbiprofen	0.08	5.50	−397.8 ± 2.5	−335.5 ± 5.6	2.8	2.75 (6)

**Table 3:** Selected electrostatic interaction energies *E*_es_ (kJ mol^−1^) for amino-acid residues of the COX-1/COX-2 protein in the binding pocket interacting with FLP

	COX-1				COX-2			
	Amino acid	No.	*E*_es_	SEM	Amino acid	No.	*E*_es_	SEM
Proximal binding pocket	Arg	120	−210.1	15.5	Arg	120	−377.6	26.8
	Tyr	355	−79.3	6.6	Tyr	355	−91.3	23.8
	Val	349	−0.1	0.3	Val	349	−3.4	6.4
	Ser	353	4.9	2.9	Ser	353	−6.5	9.0
	Ile	523	−3.8	0.2	Val	523	6.4	6.0
	Ala	527	−21.4	0.5	Ala	527	−39.2	6.3
	His	513	−1.1	0.0	Arg	513	−147.5	4.3
	Sum		−310.9	17.1	Sum		−659.1	38.7
Central binding pocket	Tyr	348	1.2	0.2	Tyr	348	7.7	0.2
	Leu	352	8.3	0.2	Leu	352	0.6	0.7
	Phe	381	−0.5	0.0	Phe	381	−6.4	0.8
	Leu	384	8.3	0.1	Leu	384	−1.4	0.1
	Tyr	385	−16.8	1.9	Tyr	385	−13.1	10.6
	Trp	387	0.5	0.3	Trp	387	−16.5	5.3
	Phe	518	0.3	0.1	Phe	518	−6.0	0.3
	Met	522	−10.2	0.5	Met	522	8.1	4.4
	Gly	526	0.1	0.3	Gly	526	−5.7	25.8
	Ser	530	−1.5	6.4	Ser	530	−18.9	3.2
	Sum		−10.4	6.7	Sum		−51.6	29.0
Distal binding pocket	Phe	205	1.5	0.1	Phe	205	−2.8	0.1
	Phe	209	1.9	0.0	Phe	209	−3.4	0.1
	Val	228	0.8	0.0	Val	228	−3.6	0.3
	Val	344	−3.0	0.1	Val	344	10.1	0.2
	Ile	377	1.0	0.0	Ile	377	−1.4	0.1
	Gly	533	−2.0	3.7	Gly	533	−4.8	0.1
	Leu	534	2.1	14.1	Leu	534	−8.1	0.5
	Sum		2.3	14.6	Sum		−14.0	0.7

**Table 4:** Selected electrostatic interaction energies *E*_es_ (kJ mol^−1^) for amino-acid residues of the COX-1/COX-2 protein in the binding pocket interacting with IBP

			COX-1			COX-2		
	Amino acid	No.	*E*_es_	SEM	Amino acid	No.	*E*_es_	SEM
Proximal binding pocket	Arg	120	−242.1	28.5	Arg	121	−215.5	44.4
	Val	349	7.1	1.8	Val	350	−6.8	0.7
	Ser	353	8.6	0.4	Ser	354	9.3	0.1
	Tyr	355	−76.8	10.0	Tyr	356	−100.3	7.4
	His	513	−1.3	0.0	Arg	514	−12.9	0.0
	Ile	523	4.8	1.4	Val	524	3.1	0.5
	Ala	527	−16.8	0.7	Ala	528	−14.5	0.9
	Sum		−330.6	30.3	Sum		−337.6	45.1
Central binding pocket	Tyr	348	0.2	0.2	Tyr	349	0.2	0.1
	Leu	352	26.5	0.1	Leu	353	37.4	0.2
	Phe	381	0.3	0.1	Phe	382	0.4	0.0
	Leu	384	10.8	0.1	Leu	385	18.4	0.7
	Tyr	385	−3.7	0.2	Tyr	386	3.3	1.0
	Trp	387	6.3	0.1	Trp	388	5.5	0.2
	Gly	526	−2.5	0.3	Gly	527	−9.7	0.8
	Phe	518	5.6	0.1	Phe	519	−3.2	0.0
	Met	522	−9.5	0.9	Met	523	6.3	0.1
	Ser	530	3.9	1.7	Ser	531	−1.2	0.7
	Sum		38.0	1.9	Sum		57.6	1.7
Distal binding pocket	Phe	205	1.2	0.1	Phe	206	1.2	0.0
	Phe	209	1.5	0.0	Phe	210	1.5	0.0
	Val	228	0.7	0.0	Val	229	0.6	0.0
	Val	344	−2.3	0.0	Val	345	−2.1	0.0
	Ile	377	1.0	0.0	Ile	378	0.7	0.0
	Gly	533	1.3	0.1	Gly	534	0.8	0.0
	Leu	534	10.4	0.1	Leu	535	11.7	0.4
	Sum		13.8	0.2	Sum		14.4	0.4

**Table 5:** Selected electrostatic interaction energies *E*_es_ (kJ mol^−1^) for amino-acid residues of the COX-1/COX-2 protein in the binding pocket interacting with MXM

	COX-1				COX-2			
	Amino acid	No.	*E*_es_	SEM	Amino acid	No.	*E*_es_	SEM
Proximal binding pocket	Arg	120	−135.7	41.0	Arg	120	−140.4	16.5
	Val	349	−9.4	3.6	Val	349	−6.3	5.0
	Ser	353	−5.6	5.0	Ser	353	−8.6	3.4
	Tyr	355	−10.9	1.6	Tyr	355	−13.3	4.3
	Ile	523	5.2	10.6	Val	523	9.8	2.6
	Ala	527	−38.9	3.4	Ala	527	−25.2	4.6
	Hip	513	−9.4	12.9	Arg	513	−45.3	4.4
	Sum		−204.8	44.9	Sum		−229.4	19.4
Central binding pocket	Tyr	348	2.6	0.8	Tyr	348	2.8	1.5
	Leu	352	−20.6	27.5	Leu	352	−13.5	15.3
	Tyr	385	−0.5	1.1	Tyr	385	−6.8	4.2
	Trp	387	−15.3	4.8	Trp	387	−3.0	4.8
	Gly	526	11.8	6.9	Gly	526	13.3	3.4
	Ser	530	−47.5	43.3	Ser	530	−80.3	9.7
	Phe	381	−2.9	1.4	Phe	381	−3.6	0.7
	Phe	518	−3.0	7.7	Phe	518	−3.1	1.7
	Leu	384	−9.6	15.5	Leu	384	−8.6	8.0
	Met	522	−6.3	2.0	Met	522	2.8	1.8
	Sum		−91.4	54.8	Sum		−100.0	21.3
Distal binding pocket	Phe	205	−0.3	1.1	Phe	205	−1.0	1.0
	Phe	209	−0.4	1.3	Phe	209	−1.1	0.9
	Val	228	−0.3	1.6	Val	228	−1.5	0.9
	Val	344	1.8	1.9	Val	344	3.0	3.1
	Ile	377	−0.1	0.8	Ile	377	−0.4	0.4
	Gly	534	−4.3	3.3	Gly	534	−6.2	1.4
	Leu	535	0.2	26.6	Leu	535	−6.0	5.5
	Sum		−3.4	27.0	Sum		−13.1	6.7

**Table 6:** Selected electrostatic interaction energies *E*_es_ (kJ mol^−1^) for amino-acid residues of the COX-1/COX-2 protein in the binding pocket interacting with CEL

	COX-1				COX-2			
	Amino acid	No.	*E*_es_	SEM	Amino acid	No.	*E*_es_	SEM
Proximal binding pocket	Arg	120	−6.1	36.4	Arg	106	−42.0	1.3
	Tyr	355	−18.3	12.8	Tyr	341	−19.0	0.5
	Val	349	−1.5	2.5	Val	335	−3.0	3.3
	Ser	353	−29.5	13.0	Ser	339	−37.9	14.4
	His	513	−2.3	15.0	Arg	499	−49.3	3.7
	Ile	523	−60.2	4.3	Val	509	−16.1	2.7
	Ala	527	−0.8	3.2	Ala	513	−7.8	4.7
	Sum		−118.7	43.8	Sum		−175.2	16.2
Central binding pocket	Tyr	348	−1.6	3.2	Tyr	334	0.4	2.3
	Leu	352	−88.1	52.8	Leu	338	−40.0	27.2
	Phe	381	0.8	0.7	Phe	367	0.3	0.4
	Leu	384	19.4	12.8	Leu	370	−1.2	1.1
	Tyr	385	4.2	2.2	Tyr	371	−4.4	1.0
	Trp	387	3.0	5.1	Trp	373	−3.5	2.6
	Phe	518	−5.9	21.5	Phe	504	−6.2	13.1
	Met	522	−29.2	5.3	Met	508	−13.8	3.9
	Gly	526	0.6	0.6	Gly	512	−0.2	6.6
	Ser	530	13.9	5.7	Ser	516	11.4	5.2
	Sum		−83.0	59.3	Sum		−57.2	31.8
Distal binding pocket	Phe	205	0.8	0.4	Phe	191	0.7	0.1
	Phe	209	1.5	0.8	Phe	195	1.1	0.1
	Val	228	1.2	1.0	Val	214	0.6	0.1
	Val	344	-0.8	2.4	Val	330	1.6	0.6
	Ile	377	0.9	0.6	Ile	363	0.4	0.1
	Gly	533	1.7	1.2	Gly	519	1.1	0.2
	Leu	534	11.8	13.4	Leu	520	1.1	0.1
	Sum		17.1	13.7	Sum		6.6	0.6

**Table 7:** Sum of the electrostatic interaction energies (kJ mol^−1^) calculated for the investigated NSAIDs and COX-1/COX-2 in the active pockets, along with RSE values averaged across different chains in the analyzed structures

	COX-1		COX-2	
NSADID	*E*_es_	RSE	*E*_es_	RSE
Celeoxib	−184.6	75.0	−225.8	35.7
Meloxicam	−299.6	75.8	−342.5	29.5
Ibuprofen	−278.8	30.4	−265.6	45.1
Flurbiprofen	−318.9	23.5	−724.7	48.3

## References

[bb1] Aktar, S., Khan, M. F., Rahman, M. M. & Rashid, M. A. (2016). *Dhaka Univ. J. Pharm. Sci.* **15**, 37–45.

[bb2] Allen, F. H. & Bruno, I. J. (2010). *Acta Cryst.* B**66**, 380–386. 10.1107/S0108768110012048 20484809

[bb3] Ayub, M. & Islam, A. (2015). *OALib*, **2**, 1–11.

[bb4] Basson, M. D., Panzini, L. & Palmer, R. H. (2001). *Aliment. Pharmacol. Ther.* **15**, 539–542. 10.1046/j.1365-2036.2001.00948.x 11284783

[bb5] Berman, H. M., Westbrook, J., Feng, Z., Gilliland, G., Bhat, T. N., Weissig, H., Shindyalov, I. N. & Bourne, P. E. (2000). *Nucleic Acids Res.* **28**, 235–242. 10.1093/nar/28.1.235 PMC102472 10592235

[bb6] Bitencourt-Ferreira, G. & de Azevedo Junior, W. F. (2021). *Curr. Med. Chem.* **28**, 4954–4971. 10.2174/0929867328666210201150842 33593246

[bb7] Blobaum, A. L. & Marnett, L. J. (2007). *J. Med. Chem.* **50**, 1425–1441. 10.1021/jm0613166 17341061

[bb8] Bojarowski, S. A., Gruza, B., Trzybiński, D., Kamiński, R., Hoser, A. A., Kumar, P., Woźniak, K. & Dominiak, P. M. (2022). *Acta Cryst.* B**78**, 823–834.

[bb9] Brock, C. P., Dunitz, J. D. & Hirshfeld, F. L. (1991). *Acta Cryst.* B**47**, 789–797.

[bb10] Browner, M. F. (1996). *New Targets in Inflammation: Inhibitors of COX-2 or Adhesion Molecules*, edited by N. Bazan, J. Botting & J. Vane, pp. 71–74. Dordrecht: Springer.

[bb11] Brune, K. & Patrignani, P. (2015). *J. Pain Res.* **8**, 105–118. 10.2147/JPR.S75160 PMC4346004 25759598

[bb12] Budniak, U. A., Karolak, N. K., Kulik, M., Młynarczyk, K., Górna, M. W. & Dominiak, P. M. (2022). *J. Phys. Chem. B*, **126**, 9152–9167. 10.1021/acs.jpcb.2c04519 PMC9677429 36326196

[bb13] Collen, M. J. & Abdulian, J. D. (1996). *J. Gastro Hepatol.* **11**, 520–523. 10.1111/j.1440-1746.1996.tb01695.x 8792303

[bb14] Copeland, R. A., Pompliano, D. L. & Meek, T. D. (2006). *Nat. Rev. Drug Discov.* **5**, 730–739. 10.1038/nrd2082 16888652

[bb15] Coppens, P. (1997). *X-ray Charge Densities and Chemical Bonding*. Oxford University Press.

[bb16] Cryer, B. & Feldman, M. (1998). *Am. J. Med.* **104**, 413–421. 10.1016/s0002-9343(98)00091-6 9626023

[bb17] Czyżnikowska, Z., Góra, R. W., Zaleśny, R., Lipkowski, P., Jarzembska, K. N., Dominiak, P. M. & Leszczynski, J. (2010). *J. Phys. Chem. B*, **114**, 9629–9644. 10.1021/jp101258q 20604521

[bb18] Devi Rajendran, N., David Stephen, A., Jelsch, C. & Escudero-Adán, E. C. (2018). *Croat. Chem. Acta*, **91**, 221–232.

[bb19] Dominiak, P. M., Volkov, A., Dominiak, A. P., Jarzembska, K. N. & Coppens, P. (2009). *Acta Cryst.* D**65**, 485–499. 10.1107/S0907444909009433 PMC2672818 19390154

[bb20] Dominiak, P. M., Volkov, A., Li, X., Messerschmidt, M. & Coppens, P. (2007). *J. Chem. Theory Comput.* **3**, 232–247. 10.1021/ct6001994 26627168

[bb21] Duan, L., Liu, X. & Zhang, J. Z. H. (2016). *J. Am. Chem. Soc.* **138**, 5722–5728. 10.1021/jacs.6b02682 27058988

[bb22] Duggan, K. C., Walters, M. J., Musee, J., Harp, J. M., Kiefer, J. R., Oates, J. A. & Marnett, L. J. (2010). *J. Biol. Chem.* **285**, 34950–34959. 10.1074/jbc.M110.162982 PMC2966109 20810665

[bb23] Engler, N., Ostermann, A., Niimura, N. & Parak, F. G. (2003). *Proc. Natl Acad. Sci. USA*, **100**, 10243–10248. 10.1073/pnas.1834279100 PMC193546 12937341

[bb24] Funk, C. D. & FitzGerald, G. A. (2007). *J. Cardiovasc. Pharmacol.* **50**, 470–479. 10.1097/FJC.0b013e318157f72d 18030055

[bb25] Ghlichloo, I. & Gerriets, V. (2023). *StatPearls.* Treasure Island: StatPearls Publishing. 31613522

[bb26] Gilroy, D. W., Tomlinson, A. & Willoughby, D. A. (1998). *Inflamm. Res.* **47**, 79–85. 10.1007/s000110050285 9535546

[bb27] Gnjidic, D., Blyth, F. M., Le Couteur, D. G., Cumming, R. G., McLachlan, A. J., Handelsman, D. J., Seibel, M., Waite, L. & Naganathan, V. (2014). *Pain*, **155**, 1814–1820. 10.1016/j.pain.2014.06.009 24954164

[bb28] Groom, C. R., Bruno, I. J., Lightfoot, M. P. & Ward, S. C. (2016). *Acta Cryst.* B**72**, 171–179. 10.1107/S2052520616003954 PMC4822653 27048719

[bb29] Grösch, S., Tegeder, I., Niederberger, E., Bräutigam, L. & Geisslinger, G. (2001). *FASEB J.* **15**, 1–22. 10.1096/fj.01-0299fje 11606477

[bb30] Guillot, B., Jelsch, C., Podjarny, A. & Lecomte, C. (2008). *Acta Cryst.* D**64**, 567–588. 10.1107/S0907444908006082 18453693

[bb31] Guillot, B., Viry, L., Guillot, R., Lecomte, C. & Jelsch, C. (2001). *J. Appl. Cryst.* **34**, 214–223.

[bb32] Guo, J., Sun, W., Li, L., Liu, F. & Lu, W. (2017). *RSC Adv.* **7**, 43491–43501.

[bb33] Gwanyanya, A., Macianskiene, R. & Mubagwa, K. (2012). *J. Pharm. Pharmacol.* **64**, 1359–1375. 10.1111/j.2042-7158.2012.01479.x 22943167

[bb34] Habeeb, A. G., Praveen Rao, P. N. & Knaus, E. E. (2001). *J. Med. Chem.* **44**, 3039–3042. 10.1021/jm010153c 11520213

[bb35] Hawkey, C. J. (1999). *Lancet*, **353**, 307–314. 10.1016/s0140-6736(98)12154-2 9929039

[bb36] Hermanson, D. J., Gamble-George, J. C., Marnett, L. J. & Patel, S. (2014). *Trends Pharmacol. Sci.* **35**, 358–367. 10.1016/j.tips.2014.04.006 PMC4074568 24845457

[bb37] Huang, B., Liu, F.-F., Dong, X.-Y. & Sun, Y. (2011). *J. Phys. Chem. B*, **115**, 4168–4176. 10.1021/jp111216g 21425823

[bb38] Jarzembska, K. N. & Dominiak, P. M. (2012). *Acta Cryst.* A**68**, 139–147. 10.1107/S0108767311042176 22186290

[bb39] Jüni, P., Nartey, L., Reichenbach, S., Sterchi, R., Dieppe, P. A. & Egger, M. (2004). *Lancet*, **364**, 2021–2029. 10.1016/S0140-6736(04)17514-4 15582059

[bb40] Kossiakoff, A. A. & Spencer, S. A. (1981). *Biochemistry*, **20**, 6462–6474. 10.1021/bi00525a027 7030393

[bb41] Krzyżak, E., Szkatuła, D., Wiatrak, B., Gębarowski, T. & Marciniak, A. (2020). *Molecules*, **25**, 2934. 10.3390/molecules25122934 PMC7355801 32630594

[bb42] Kumar, P. & Dominiak, P. M. (2021). *Molecules*, **26**, 3872. 10.3390/molecules26133872 PMC8270314 34202892

[bb43] Kurumbail, R. G., Stevens, A. M., Gierse, J. K., McDonald, J. J., Stegeman, R. A., Pak, J. Y., Gildehaus, D., Miyashiro, J. M., Penning, T. D., Seibert, K., Isakson, P. C. & Stallings, W. C. (1996). *Nature*, **384**, 644–648. 10.1038/384644a0 8967954

[bb44] Lai, K. M., Chen, T.-L., Chang, C.-C., Chen, H.-H. & Lee, Y.-W. (2019). *Clin. Epidemiol.* **11**, 429–441. 10.2147/CLEP.S204322 PMC6549765 31213924

[bb45] Li, X., Volkov, A. V., Szalewicz, K. & Coppens, P. (2006). *Acta Cryst.* D**62**, 639–647. 10.1107/S0907444906013072 16699191

[bb46] Luong, C., Miller, A., Barnett, J., Chow, J., Ramesha, C. & Browner, M. F. (1996). *Nat. Struct. Mol. Biol.* **3**, 927–933. 10.1038/nsb1196-927 8901870

[bb47] Madhavi Sastry, G., Adzhigirey, M., Day, T., Annabhimoju, R. & Sherman, W. (2013). *J. Comput. Aided Mol. Des.* **27**, 221–234. 10.1007/s10822-013-9644-8 23579614

[bb48] Malinska, M. & Dauter, Z. (2016). *Acta Cryst.* D**72**, 770–779. 10.1107/S2059798316006355 PMC4908868 27303797

[bb49] Malińska, M., Jarzembska, K. N., Goral, A. M., Kutner, A., Woźniak, K. & Dominiak, P. M. (2014). *Acta Cryst.* D**70**, 1257–1270. 10.1107/S1399004714002351 24816095

[bb50] Malinska, M., Kutner, A. & Woźniak, K. (2015). *Steroids*, **104**, 220–229. 10.1016/j.steroids.2015.10.007 26476188

[bb51] Malmberg, A. B. & Yaksh, T. L. (1992). *Science*, **257**, 1276–1279. 10.1126/science.1381521 1381521

[bb52] Masferrer, J. L., Zweifel, B. S., Manning, P. T., Hauser, S. D., Leahy, K. M., Smith, W. G., Isakson, P. C. & Seibert, K. (1994). *Proc. Natl Acad. Sci. USA*, **91**, 3228–3232. 10.1073/pnas.91.8.3228 PMC43549 8159730

[bb53] McGettigan, P. & Henry, D. (2006). *JAMA*, **296**, 1633–1644. 10.1001/jama.296.13.jrv60011 16968831

[bb54] Miciaccia, M., Belviso, B. D., Iaselli, M., Cingolani, G., Ferorelli, S., Cappellari, M., Loguercio Polosa, P., Perrone, M. G., Caliandro, R. & Scilimati, A. (2021). *Sci. Rep.* **11**, 4312. 10.1038/s41598-021-83438-z PMC7900114 33619313

[bb55] Mitchell, J. A., Akarasereenont, P., Thiemermann, C., Flower, R. J. & Vane, J. R. (1993). *Proc. Natl Acad. Sci. USA*, **90**, 11693–11697. 10.1073/pnas.90.24.11693 PMC48050 8265610

[bb56] Orlando, B. J., Lucido, M. J. & Malkowski, M. G. (2015). *J. Struct. Biol.* **189**, 62–66. 10.1016/j.jsb.2014.11.005 PMC4276492 25463020

[bb57] Parambil, S. H. K., Parambil, H. A. T., Hamza, S. P., Parameswaran, A. T., Thayyil, M. S., Karuvanthodi, M., Parambil, S. H. K., Parambil, H. A. T., Hamza, S. P., Parameswaran, A. T., Thayyil, M. S. & Karuvanthodi, M. (2020). *Density Functional Theory Calculations.* Rijeka: IntechOpen.

[bb58] Penning, T. D., Talley, J. J., Bertenshaw, S. R., Carter, J. S., Collins, P. W., Docter, S., Graneto, M. J., Lee, L. F., Malecha, J. W., Miyashiro, J. M., Rogers, R. S., Rogier, D. J., Yu, S. S., Anderson, G. D., Burton, E. G., Cogburn, J. N., Gregory, S. A., Koboldt, C. M., Perkins, W. E., Seibert, K., Veenhuizen, A. W., Zhang, Y. Y. & Isakson, P. C. (1997). *J. Med. Chem.* **40**, 1347–1365. 10.1021/jm960803q 9135032

[bb59] Pereira-Leite, C., Figueiredo, M., Burdach, K., Nunes, C. & Reis, S. (2021). *Membranes*, **11**, 24. 10.3390/membranes11010024 PMC7824678 33383697

[bb60] Perez, Y. R., Alvarez, D. & Combariza, A. (2019). *Ligand–Protein Interactions: A Hybrid Ab Initio/Molecular Mechanics Computational Study.*https://doi.org/10.20944/preprints201902.0124.v1.

[bb61] Piłat, Z. & Antosiewicz, J. M. (2008). *J. Phys. Chem. B*, **112**, 15074–15085. 10.1021/jp8029659 18950218

[bb62] Rao, P. & Knaus, E. E. (2008). *J. Pharm. Pharm. Sci.* **11**, 81. 10.18433/j3t886 19203472

[bb63] Rimon, G., Sidhu, R. S., Lauver, D. A., Lee, J. Y., Sharma, N. P., Yuan, C., Frieler, R. A., Trievel, R. C., Lucchesi, B. R. & Smith, W. L. (2010). *Proc. Natl Acad. Sci. USA*, **107**, 28–33. 10.1073/pnas.0909765106 PMC2806742 19955429

[bb64] Roth, S. H. (1995). *Drugs Aging*, **6**, 358–367. 10.2165/00002512-199506050-00003 7647425

[bb65] Rowlinson, S. W., Kiefer, J. R., Prusakiewicz, J. J., Pawlitz, J. L., Kozak, K. R., Kalgutkar, A. S., Stallings, W. C., Kurumbail, R. G. & Marnett, L. J. (2003). *J. Biol. Chem.* **278**, 45763–45769. 10.1074/jbc.M305481200 12925531

[bb66] Sai Ram, K. V. V. M., Rambabu, G., Sarma, J. A. R. P. & Desiraju, G. R. (2006). *J. Chem. Inf. Model.* **46**, 1784–1794. 10.1021/ci050142i 16859310

[bb68] Selinsky, B. S., Gupta, K., Sharkey, C. T. & Loll, P. J. (2001). *Biochemistry*, **40**, 5172–5180. 10.1021/bi010045s 11318639

[bb69] Smith, C. J., Zhang, Y., Koboldt, C. M., Muhammad, J., Zweifel, B. S., Shaffer, A., Talley, J. J., Masferrer, J. L., Seibert, K. & Isakson, P. C. (1998). *Proc. Natl Acad. Sci. USA*, **95**, 13313–13318. 10.1073/pnas.95.22.13313 PMC23795 9789085

[bb70] Solomon, S. D., McMurray, J. J. V., Pfeffer, M. A., Wittes, J., Fowler, R., Finn, P., Anderson, W. F., Zauber, A., Hawk, E., Bertagnolli, M. & Adenoma Prevention with Celecoxib (APC) Study Investigators (2005). *N. Engl. J. Med.* **352**, 1071–1080. 10.1056/NEJMoa050405 15713944

[bb71] Su, Z. & Coppens, P. (1992). *Acta Cryst.* A**48**, 188–197. 10.1107/s0108767391009820 1575938

[bb72] Takaba, K., Tai, Y., Eki, H., Dao, H.-A., Hanazono, Y., Hasegawa, K., Miki, K. & Takeda, K. (2019). *IUCrJ*, **6**, 387–400. 10.1107/S205225251900246X PMC6503917 31098020

[bb73] Tegeder, I., Pfeilschifter, J. & Geisslinger, G. (2001). *FASEB J.* **15**, 2057–2072. 10.1096/fj.01-0390rev 11641233

[bb74] Tenenbaum, J. (1999). *Can. J. Gastroenterol. Hepatol.* **13**, 119–122. 10.1155/1999/361651 10203429

[bb75] Tielemans, M. M., van Rossum, L. G. M., Eikendal, T., Focks, J. J., Laheij, R. J. F., Jansen, J. B. M. J. & van Oijen, M. G. H. (2014). *Int. J. Clin. Pract.* **68**, 512–519. 10.1111/ijcp.12346 24499203

[bb76] Tong, Y., Mei, Y., Li, Y. L., Ji, C. G. & Zhang, J. Z. H. (2010). *J. Am. Chem. Soc.* **132**, 5137–5142. 10.1021/ja909575j 20302307

[bb77] Tuwalaid, B., Iswantini, D. & Tri Wahyudi, S. (2022). *Indones. J. Chem.* **22**, 754–769.

[bb78] Vane, J. R. & Botting, R. M. (1998). *Inflamm. Res.* **47**, 78–87. 10.1007/s000110050284 9831328

[bb79] Vane, J. R. & Botting, R. M. (2003). *Thromb. Res.* **110**, 255–258. 10.1016/s0049-3848(03)00379-7 14592543

[bb80] Vecchio, A. J., Simmons, D. M. & Malkowski, M. G. (2010). *J. Biol. Chem.* **285**, 22152–22163. 10.1074/jbc.M110.119867 PMC2903402 20463020

[bb81] Volkov, A., Li, X., Koritsanszky, T. & Coppens, P. (2004). *J. Phys. Chem. A*, **108**, 4283–4300.

[bb82] Volkov, A., Macchi, C., Farrugia, L. J., Gatti, P., Mallinson, P. R., Richter, T. & Koritsanszky, T. (2016). *XD2016: A Computer Program Package for Multipole Refinement, Topological Analysis of Charge Densities and Evaluation of Intermolecular Energies from Experimental and Theoretical Structure Factors*. https://www.chem.gla.ac.uk/~louis/xd-home/.

[bb83] Wang, J. L., Limburg, D., Graneto, M. J., Springer, J., Hamper, J. R. B., Liao, S., Pawlitz, J. L., Kurumbail, R. G., Maziasz, T., Talley, J. J., Kiefer, J. R. & Carter, J. (2010). *Bioorg. Med. Chem. Lett.* **20**, 7159–7163. 10.1016/j.bmcl.2010.07.054 20709553

[bb84] Warner, T. D. & Mitchell, J. A. (2004). *FASEB J.* **18**, 790–804. 10.1096/fj.03-0645rev 15117884

[bb85] Williams, C. J., Headd, J. J., Moriarty, N. W., Prisant, M. G., Videau, L. L., Deis, L. N., Verma, V., Keedy, D. A., Hintze, B. J., Chen, V. B., Jain, S., Lewis, S. M., Arendall, W. B., Snoeyink, J., Adams, P. D., Lovell, S. C., Richardson, J. S. & Richardson, D. C. (2018). *Protein Sci.* **27**, 293–315. 10.1002/pro.3330 PMC5734394 29067766

[bb86] Xu, S., Hermanson, D. J., Banerjee, S., Ghebreselasie, K., Clayton, G. M., Garavito, R. M. & Marnett, L. J. (2014). *J. Biol. Chem.* **289**, 6799–6808. 10.1074/jbc.M113.517987 PMC3945341 24425867

