# Supplementary material for: Understanding the selectivity of nonsteroidal anti-inflammatory drugs for cyclooxygenases using quantum crystallography and electrostatic interaction energy
Source: IUCrJ. 2025 Jan 30;12(Pt 2):208–22. doi: 10.1107/S2052252525000053 (PMC11878451; doi:10.1107/S2052252525000053)
Supplement: Supplementary file 1 [file m-12-00208-sup1.pdf]

IUCrJ

Volume 12 (2025)

Supporting information for article:

Understanding the selectivity of nonsteroidal anti-inflammatory drugs for cyclooxygenases using quantum crystallography and electrostatic interaction energy

S. Pawlędzio, M. Ziemniak, X. Wang, K. Woźniak and M. Malinska

Table of Contents

Table S1. Electrostatic interaction energies Ees [kJ/mol] for amino acid residues of chain A and B of the COX-1 protein with the FLP. 1

Table S2. Electrostatic interaction energies Ees [kJ/mol] for amino acid residues of chain A and B of the COX-2 protein with the FLP. 6

Table S3. Electrostatic interaction energies Ees [kJ/mol] for amino acid residues of chain C and D of the COX-2 protein with the FLP. 10

Table S4. Electrostatic interaction energies E_{es} [kJ/mol] for amino acid residues of chain A and B of the COX-1 protein with the IBP. 14

Table S5. Electrostatic interaction energies E_{es} [kJ/mol] for amino acid residues of chain A and B of the COX-2 protein with the IBP. 19

Table S6. Electrostatic interaction energies Ees [kJ/mol] for amino acid residues of chain A and B (conformers A) of the COX-1 protein with the MXM. 23

Table S7. Electrostatic interaction energies Ees [kJ/mol] for amino acid residues of chain A and B (conformers A) of the COX-2 protein with the MXM. 27

Table S8. Electrostatic interaction energies Ees [kJ/mol] for amino acid residues of chain C and D (conformers A) of the COX-2 protein with the MXM. 31

Table S9. Electrostatic interaction energies Ees [kJ/mol] for amino acid residues of chain A and B of the COX-1 protein with the CEL 36

Table S10. Electrostatic interaction energies E_{es} [kJ/mol] for amino acid residues of chain A and B of the COX-2 protein with the CEL. 40

Table S1. Electrostatic interaction energies Ees [kJ/mol] for amino acid residues of chain A and B of the COX-1 protein with the FLP.

Chain A	Chain B

aa	NO	kJ	aa	NO	kJ	aa	NO	kJ	aa	NO	kJ	aa	NO	kJ	aa	NO	kJ
ASN	34	0.2	GLY	217	-0.1	ASP	401	-2.8	ASN	34	0.2	GLY	217	-0.2	ASP	401	-2.8
PRO	35	0.1	PRO	218	0.1	TYR	402	-0.1	PRO	35	0.1	PRO	218	0.1	TYR	402	0.0
CYS	36	0.1	GLY	219	0.2	SER	403	-0.2	CYS	36	0.1	GLY	219	0.2	SER	403	-0.2
CYS	37	0.0	PHE	220	-0.5	TYR	404	-0.1	CYS	37	0.0	PHE	220	-0.5	TYR	404	-0.1
TYR	38	0.1	THR	221	0.1	GLU	405	-3.0	TYR	38	0.1	THR	221	0.3	GLU	405	-3.0
TYR	39	0.2	LYS	222	-0.7	GLN	406	-0.2	TYR	39	0.2	LYS	222	-0.7	GLN	406	-0.2
PRO	40	0.3	ALA	223	0.1	PHE	407	0.0	PRO	40	0.3	ALA	223	0.1	PHE	407	0.1
CYS	41	0.1	LEU	224	1.3	LEU	408	1.8	CYS	41	0.1	LEU	224	1.4	LEU	408	1.8
GLN	42	-0.2	GLY	225	-0.3	PHE	409	0.1	GLN	42	-0.2	GLY	225	-0.3	PHE	409	0.1
HIS	43	0.3	HIS	226	-0.8	ASN	410	-0.1	HIS	43	0.3	HIS	226	-0.9	ASN	410	-0.1
GLN	44	-1.1	GLY	227	-0.1	THR	411	0.0	GLN	44	-1.1	GLY	227	-0.2	THR	411	0.1
GLY	45	-0.3	VAL	228	0.7	SER	412	0.0	GLY	45	-0.3	VAL	228	0.8	SER	412	0.0
ILE	46	-0.1	ASP	229	-7.3	MET	413	-0.2	ILE	46	-0.1	ASP	229	-7.3	MET	413	-0.2
CYS	47	-0.2	LEU	230	4.0	LEU	414	1.9	CYS	47	-0.2	LEU	230	4.0	LEU	414	1.9
VAL	48	0.0	GLY	231	0.2	VAL	415	0.0	VAL	48	0.0	GLY	231	0.2	VAL	415	0.0
ARG	49	-0.5	HIS	232	1.0	ASP	416	-2.7	ARG	49	-0.5	HIS	232	0.8	ASP	416	-2.7
PHE	50	0.0	ILE	233	0.5	TYR	417	0.1	PHE	50	0.0	ILE	233	0.5	TYR	417	0.0
GLY	51	0.0	TYR	234	0.2	GLY	418	-0.1	GLY	51	0.0	TYR	234	0.2	GLY	418	-0.1
LEU	52	-0.1	GLY	235	0.1	VAL	419	-0.2	LEU	52	-0.1	GLY	235	0.1	VAL	419	-0.2
ASP	53	0.7	ASP	236	-4.7	GLU	420	-3.5	ASP	53	0.7	ASP	236	-4.7	GLU	420	-3.5
ARG	54	-0.9	ASN	237	0.2	ALA	421	-0.2	ARG	54	-0.9	ASN	237	0.2	ALA	421	-0.2
TYR	55	0.0	LEU	238	1.1	LEU	422	1.5	TYR	55	0.0	LEU	238	1.1	LEU	422	1.6
GLN	56	0.1	GLU	239	-2.7	VAL	423	-0.2	GLN	56	0.1	GLU	239	-2.7	VAL	423	-0.2
CYS	57	0.1	ARG	240	4.3	ASP	424	-3.6	CYS	57	0.1	ARG	240	4.3	ASP	424	-3.6
ASP	58	2.3	GLN	241	0.3	ALA	425	-0.2	ASP	58	2.3	GLN	241	0.3	ALA	425	-0.2
CYS	59	0.2	TYR	242	0.0	PHE	426	-0.2	CYS	59	0.2	TYR	242	0.0	PHE	426	-0.2
THR	60	0.3	GLN	243	0.0	SER	427	0.0	THR	60	0.3	GLN	243	0.0	SER	427	0.0
ARG	61	-2.7	LEU	244	1.6	ARG	428	3.3	ARG	61	-2.7	LEU	244	1.7	ARG	428	3.3
THR	62	0.5	ARG	245	3.0	GLN	429	-0.3	THR	62	0.5	ARG	245	3.1	GLN	429	-0.3
GLY	63	0.0	LEU	246	1.1	PRO	430	-0.3	GLY	63	0.0	LEU	246	1.1	PRO	430	-0.3
TYR	64	-0.1	PHE	247	0.1	ALA	431	0.5	TYR	64	-0.1	PHE	247	0.1	ALA	431	0.5
SER	65	0.0	LYS	248	-0.2	GLY	432	-0.3	SER	65	0.0	LYS	248	-0.2	GLY	432	-0.3
GLY	66	0.0	ASP	249	-2.2	ARG	433	1.1	GLY	66	0.0	ASP	249	-2.2	ARG	433	1.1
PRO	67	0.0	GLY	250	-0.2	ILE	434	1.2	PRO	67	0.0	GLY	250	-0.2	ILE	434	1.2
ASN	68	0.1	LYS	251	-0.3	GLY	435	0.3	ASN	68	0.1	LYS	251	-0.3	GLY	435	0.3
CYS	69	0.2	LEU	252	1.7	GLY	436	0.0	CYS	69	0.2	LEU	252	1.7	GLY	436	0.0
THR	70	0.1	LYS	253	-0.4	GLY	437	0.1	THR	70	0.2	LYS	253	-0.3	GLY	437	0.2
ILE	71	0.2	TYR	254	0.1	ARG	438	1.3	ILE	71	0.2	TYR	254	0.1	ARG	438	1.3
PRO	72	-0.3	GLN	255	0.1	ASN	439	0.0	PRO	72	-0.2	GLN	255	0.1	ASN	439	0.0
GLU	73	4.6	MET	256	-0.1	ILE	440	0.4	GLU	73	4.6	MET	256	-0.1	ILE	440	0.4
ILE	74	0.4	LEU	257	1.0	ASP	441	-3.9	ILE	74	0.4	LEU	257	1.0	ASP	441	-4.0
TRP	75	0.7	ASN	258	0.1	HIS	442	2.4	TRP	75	0.7	ASN	258	0.1	HIS	442	2.3
THR	76	0.5	GLY	259	-0.1	HIS	443	-1.0	THR	76	0.5	GLY	259	-0.1	HIS	443	-1.0

TRP	77	0.6	GLU	260	-2.9	ILE	444	0.0	TRP	77	0.6	GLU	260	-2.9	ILE	444	0.0
LEU	78	-1.7	VAL	261	-0.2	LEU	445	1.4	LEU	78	-1.8	VAL	261	-0.2	LEU	445	1.4
ARG	79	-10.3	TYR	262	0.0	HIS	446	0.4	ARG	79	-10.6	TYR	262	0.0	HIS	446	0.4
THR	80	0.1	PRO	263	0.3	VAL	447	-0.1	THR	80	-0.1	PRO	263	0.2	VAL	447	-0.1
THR	81	0.3	PRO	264	-0.1	ALA	448	0.3	THR	81	0.2	PRO	264	-0.1	ALA	448	0.3
LEU	82	-2.5	SER	265	0.0	VAL	449	-0.2	LEU	82	-2.5	SER	265	0.0	VAL	449	-0.2
ARG	83	-22.6	VAL	266	0.0	ASP	450	-4.3	ARG	83	-22.6	VAL	266	0.0	ASP	450	-4.3
PRO	84	1.3	GLU	267	-2.1	VAL	451	0.3	PRO	84	1.3	GLU	267	-2.1	VAL	451	0.3
SER	85	2.5	GLU	268	-2.3	ILE	452	0.2	SER	85	2.5	GLU	268	-2.3	ILE	452	0.2
PRO	86	0.1	ALA	269	0.0	LYS	453	-0.2	PRO	86	0.1	ALA	269	0.0	LYS	453	-0.2
SER	87	0.5	PRO	270	-0.1	GLU	454	-5.3	SER	87	-0.3	PRO	270	-0.1	GLU	454	-5.4
PHE	88	1.0	VAL	271	-0.1	SER	455	0.6	PHE	88	1.1	VAL	271	-0.1	SER	455	0.5
ILE	89	-1.5	LEU	272	1.2	ARG	456	2.2	ILE	89	-1.5	LEU	272	1.2	ARG	456	2.2
HIS	90	1.0	MET	273	0.0	VAL	457	0.1	HIS	90	-6.5	MET	273	0.0	VAL	457	0.1
PHE	91	-0.3	HIS	274	0.2	LEU	458	1.9	PHE	91	-0.3	HIS	274	-0.2	LEU	458	1.9
LEU	92	-6.5	TYR	275	0.1	ARG	459	1.2	LEU	92	-6.5	TYR	275	0.1	ARG	459	1.2
LEU	93	-14.0	PRO	276	0.1	LEU	460	1.8	LEU	93	-14.1	PRO	276	0.1	LEU	460	1.8
THR	94	-1.6	ARG	277	2.3	GLN	461	0.0	THR	94	-1.6	ARG	277	2.4	GLN	461	0.0
HIS	95	-0.7	GLY	278	0.0	PRO	462	0.1	HIS	95	-0.6	GLY	278	0.0	PRO	462	0.1
GLY	96	-0.1	ILE	279	0.1	PHE	463	0.7	GLY	96	-0.1	ILE	279	0.1	PHE	463	0.7
ARG	97	-0.7	PRO	280	-0.1	ASN	464	0.7	ARG	97	-0.7	PRO	280	-0.1	ASN	464	0.7
TRP	98	0.0	PRO	281	-0.1	GLU	465	3.2	TRP	98	0.0	PRO	281	-0.1	GLU	465	3.2
LEU	99	-2.1	GLN	282	-0.2	TYR	466	1.2	LEU	99	-2.1	GLN	282	-0.2	TYR	466	1.2
TRP	100	-1.4	SER	283	-0.1	ARG	467	-6.2	TRP	100	-1.4	SER	283	-0.1	ARG	467	-6.2
ASP	101	1.1	GLN	284	-0.1	LYS	468	2.3	ASP	101	1.1	GLN	284	-0.1	LYS	468	2.3
PHE	102	-0.1	MET	285	-0.2	ARG	469	-4.5	PHE	102	-0.1	MET	285	-0.2	ARG	469	-4.4
VAL	103	-0.3	ALA	286	-0.2	PHE	470	2.9	VAL	103	-0.3	ALA	286	-0.1	PHE	470	2.9
ASN	104	-0.2	VAL	287	-0.3	GLY	471	0.5	ASN	104	0.0	VAL	287	-0.3	GLY	471	0.5
ALA	105	0.0	GLY	288	-0.1	MET	472	-0.5	ALA	105	0.0	GLY	288	-0.1	MET	472	-0.5
THR	106	0.1	GLN	289	0.9	LYS	473	0.0	THR	106	0.1	GLN	289	0.9	LYS	473	0.0
ARG	106	127.2	GLU	290	-5.4	PRO	474	0.1	ARG	106	127.2	GLU	290	-5.4	PRO	474	0.1
PHE	107	0.4	VAL	291	-0.1	TYR	475	0.0	PHE	107	0.4	VAL	291	-0.2	TYR	475	0.1
ILE	108	0.5	PHE	292	0.4	THR	476	-0.4	ILE	108	0.6	PHE	292	0.4	THR	476	-0.4
ARG	109	1.7	GLY	293	0.1	SER	477	0.0	ARG	109	1.6	GLY	293	0.1	SER	477	0.0
ASP	110	2.9	LEU	294	2.0	PHE	478	0.2	ASP	110	3.0	LEU	294	2.0	PHE	478	0.2
THR	111	1.3	LEU	295	2.6	GLN	479	0.1	THR	111	1.2	LEU	295	2.6	GLN	479	0.1
LEU	112	-2.5	PRO	296	-0.3	GLU	480	4.6	LEU	112	-2.8	PRO	296	-0.4	GLU	480	4.6
MET	113	1.8	GLY	297	-0.3	LEU	481	-0.5	MET	113	1.8	GLY	297	-0.3	LEU	481	-0.5
ARG	114	-3.9	LEU	298	3.5	THR	482	0.4	ARG	114	-4.0	LEU	298	3.5	THR	482	0.5
LEU	115	-4.6	MET	299	0.1	GLY	483	-0.1	LEU	115	-5.2	MET	299	0.1	GLY	483	0.0
VAL	116	2.5	LEU	300	2.3	GLU	484	0.8	VAL	116	1.3	LEU	300	2.3	GLU	484	0.8
LEU	117	-0.8	TYR	301	0.3	LYS	485	-0.5	LEU	117	-1.1	TYR	301	0.4	LYS	485	-0.4
THR	118	1.9	ALA	302	0.3	GLU	486	-0.1	THR	118	1.8	ALA	302	0.3	GLU	486	0.0
VAL	119	0.9	THR	303	0.1	MET	487	-0.2	VAL	119	0.7	THR	303	0.1	MET	487	-0.2

ARG	120	-221.1	ILE	304	0.2	ALA	488	-0.1	ARG	120	-199.1	ILE	304	0.2	ALA	488	-0.1
SER	121	-3.1	TRP	305	1.0	ALA	489	-0.1	SER	121	-3.3	TRP	305	1.0	ALA	489	-0.1
ASN	122	-2.6	LEU	306	1.9	GLU	490	-0.6	ASN	122	-2.6	LEU	306	1.9	GLU	490	-0.6
LEU	123	-10.1	ARG	307	4.0	LEU	491	0.2	LEU	123	-9.8	ARG	307	4.0	LEU	491	0.2
ILE	124	-0.2	GLU	308	-4.6	GLU	492	0.8	ILE	124	-0.2	GLU	308	-4.6	GLU	492	0.8
PRO	125	-0.5	HIS	309	0.1	GLU	493	-0.1	PRO	125	-0.5	HIS	309	0.0	GLU	493	-0.1
SER	126	-0.1	ASN	310	0.1	LEU	494	0.3	SER	126	0.1	ASN	310	0.1	LEU	494	0.3
PRO	127	0.1	ARG	311	3.8	TYR	495	0.0	PRO	127	0.1	ARG	311	3.8	TYR	495	0.0
PRO	128	0.3	VAL	312	0.1	GLY	496	0.1	PRO	128	0.3	VAL	312	0.1	GLY	496	0.1
THR	129	-0.3	CYS	313	0.4	ASP	497	1.2	THR	129	-0.3	CYS	313	0.4	ASP	497	1.2
TYR	130	-0.4	ASP	314	-2.4	ILE	498	-0.1	TYR	130	-0.3	ASP	314	-2.4	ILE	498	-0.1
ASN	131	0.2	LEU	315	1.2	ASP	499	1.8	ASN	131	0.2	LEU	315	1.2	ASP	499	1.8
ILE	132	0.3	LEU	316	1.2	ALA	500	-0.2	ILE	132	0.3	LEU	316	1.2	ALA	500	-0.2
ALA	133	0.2	LYS	317	0.0	LEU	501	-0.1	ALA	133	0.2	LYS	317	0.0	LEU	501	-0.1
HIS	134	0.0	ALA	318	0.1	GLU	502	-2.0	HIS	134	0.0	ALA	318	0.1	GLU	502	-2.0
ASP	135	-0.5	GLU	319	-1.7	PHE	503	0.0	ASP	135	-0.5	GLU	319	-1.7	PHE	503	0.0
TYR	136	-0.1	HIP	320	-2.9	TYR	504	0.9	TYR	136	-0.1	HIP	320	1.4	TYR	504	0.9
ILE	137	-0.1	PRO	321	0.0	PRO	505	0.2	ILE	137	-0.1	PRO	321	0.0	PRO	505	0.2
SER	138	-0.1	THR	322	0.0	GLY	506	0.1	SER	138	-0.1	THR	322	0.0	GLY	506	0.1
TRP	139	-0.2	TRP	323	0.0	LEU	507	3.4	TRP	139	-0.2	TRP	323	0.0	LEU	507	3.4
GLU	140	-1.7	GLY	324	-0.1	LEU	508	3.6	GLU	140	-1.7	GLY	324	-0.1	LEU	508	3.6
SER	141	-0.3	ASP	325	-2.5	LEU	509	1.2	SER	141	-0.3	ASP	325	-2.5	LEU	509	1.2
PHE	142	-0.3	GLU	326	-2.6	GLU	510	4.6	PHE	142	-0.2	GLU	326	-2.6	GLU	510	4.7
SER	143	-0.2	GLN	327	-0.1	LYS	511	-0.4	SER	143	-0.2	GLN	327	-0.1	LYS	511	-0.4
ASN	144	-0.1	LEU	328	0.7	CYS	512	-0.1	ASN	144	-0.1	LEU	328	0.7	CYS	512	0.0
VAL	145	-0.4	PHE	329	-0.1	HIS	513	-1.0	VAL	145	-0.4	PHE	329	-0.1	HIS	513	-1.1
SER	146	-0.3	GLN	330	-0.3	PRO	514	-0.7	SER	146	-0.3	GLN	330	-0.3	PRO	514	-0.7
TYR	147	-0.3	THR	331	-0.2	ASN	515	0.1	TYR	147	-0.3	THR	331	-0.2	ASN	515	0.1
TYR	148	0.3	ALA	332	-0.3	SER	516	-1.4	TYR	148	0.3	ALA	332	-0.3	SER	516	-1.4
THR	149	0.0	ARG	333	4.7	ILE	517	-0.1	THR	149	0.0	ARG	333	4.7	ILE	517	-0.1
ARG	150	2.3	LEU	334	1.2	PHE	518	0.3	ARG	150	2.4	LEU	334	1.2	PHE	518	0.3
ILE	151	0.5	ILE	335	-0.6	GLY	519	1.8	ILE	151	0.6	ILE	335	-0.6	GLY	519	1.8
LEU	152	-0.9	LEU	336	1.6	GLU	520	16.7	LEU	152	-0.8	LEU	336	1.6	GLU	520	16.1
PRO	153	-0.2	ILE	337	-0.7	SER	521	-1.1	PRO	153	-0.2	ILE	337	-0.7	SER	521	-1.3
SER	154	0.3	GLY	338	-0.6	MET	522	-10.5	SER	154	0.3	GLY	338	-0.6	MET	522	-9.9
VAL	155	0.0	GLU	339	-7.2	ILE	523	-4.0	VAL	155	0.0	GLU	339	-7.1	ILE	523	-3.6
PRO	156	-0.1	THR	340	-1.3	GLU	524	50.7	PRO	156	-0.1	THR	340	-1.3	GLU	524	49.6
ARG	157	1.2	ILE	341	-1.3	MET	525	-3.9	ARG	157	1.2	ILE	341	-1.3	MET	525	-3.7
ASP	158	0.1	LYS	342	-3.4	GLY	526	-0.1	ASP	158	0.0	LYS	342	-3.4	GLY	526	0.3
CYS	159	0.0	ILE	343	-2.0	ALA	527	-21.0	CYS	159	0.0	ILE	343	-2.0	ALA	527	-21.8
PRO	160	-0.1	VAL	344	-3.0	PRO	528	-6.9	PRO	160	-0.1	VAL	344	-3.0	PRO	528	-7.1
THR	161	-0.1	ILE	345	-0.9	PHE	529	-1.7	THR	161	-0.1	ILE	345	-0.7	PHE	529	-1.8
PRO	162	0.0	GLU	346	-8.1	SER	530	3.0	PRO	162	0.0	GLU	346	-8.0	SER	530	-6.1
MET	163	0.1	GLU	347	-10.3	LEU	531	-2.3	MET	163	0.1	GLU	347	-10.2	LEU	531	-1.0

GLY	164	0.1	TYR	348	1.0	LYS	532	-3.4	GLY	164	0.1	TYR	348	1.3	LYS	532	-3.4
THR	165	-0.1	VAL	349	0.1	GLY	533	0.6	THR	165	-0.1	VAL	349	-0.4	GLY	533	0.6
LYS	166	-0.5	GLN	350	0.6	LEU	534	12.0	LYS	166	-0.5	GLN	350	0.8	LEU	534	12.1
GLY	167	-0.1	GLN	351	1.7	LEU	535	2.3	GLY	167	0.0	GLN	351	1.6	LEU	535	2.2
LYS	168	0.0	LEU	352	8.4	GLY	536	0.3	LYS	168	0.0	LEU	352	8.2	GLY	536	0.1
LYS	169	-0.3	SER	353	2.9	ASN	537	1.6	LYS	169	-0.3	SER	353	6.9	ASN	537	1.4
GLN	170	0.1	GLY	354	1.7	PRO	538	0.2	GLN	170	0.1	GLY	354	1.6	PRO	538	0.2
LEU	171	0.4	TYR	355	-74.6	ILE	539	0.5	LEU	171	0.4	TYR	355	-83.9	ILE	539	0.5
PRO	172	-0.2	PHE	356	-0.9	CYS	540	0.3	PRO	172	-0.2	PHE	356	-0.8	CYS	540	0.5
ASP	173	-1.9	LEU	357	-4.6	SER	541	0.1	ASP	173	-1.9	LEU	357	-4.9	SER	541	0.2
ALA	174	-0.1	GLN	358	0.3	PRO	542	-0.2	ALA	174	-0.1	GLN	358	0.3	PRO	542	-0.2
GLU	175	-2.6	LEU	359	10.7	GLU	543	-1.0	GLU	175	-2.6	LEU	359	11.9	GLU	543	-1.0
PHE	176	-0.1	LYS	360	-2.5	TYR	544	-0.1	PHE	176	-0.1	LYS	360	-2.3	TYR	544	-0.1
LEU	177	0.6	PHE	361	0.8	TRP	545	0.3	LEU	177	0.6	PHE	361	0.9	TRP	545	0.3
SER	178	-0.1	ASP	362	-0.7	LYS	546	-0.5	SER	178	-0.1	ASP	362	-0.7	LYS	546	-0.5
ARG	179	2.5	PRO	363	0.9	ALA	547	-0.1	ARG	179	2.5	PRO	363	0.9	ALA	547	-0.1
ARG	180	1.1	GLU	364	0.3	SER	548	-0.2	ARG	180	1.1	GLU	364	0.3	SER	548	-0.2
PHE	181	-0.2	LEU	365	0.1	THR	549	-0.1	PHE	181	-0.2	LEU	365	0.1	THR	549	0.0
LEU	182	1.4	LEU	366	-1.3	PHE	550	0.1	LEU	182	1.4	LEU	366	-1.3	PHE	550	0.1
LEU	183	1.1	PHE	367	0.4	GLY	551	0.1	LEU	183	1.1	PHE	367	0.4	GLY	551	0.1
ARG	184	4.4	GLY	368	0.2	GLY	552	-0.1	ARG	184	4.4	GLY	368	0.2	GLY	552	-0.1
ARG	185	1.7	ALA	369	-0.4	GLU	553	-1.8	ARG	185	1.7	ALA	369	-0.4	GLU	553	-1.8
LYS	186	-0.3	GLN	370	0.6	VAL	554	-0.3	LYS	186	-0.3	GLN	370	0.6	VAL	554	-0.3
PHE	187	0.3	PHE	371	-0.4	GLY	555	-0.2	PHE	187	0.3	PHE	371	-0.4	GLY	555	-0.2
ILE	188	-0.3	GLN	372	-0.8	PHE	556	-0.3	ILE	188	-0.3	GLN	372	-0.8	PHE	556	-0.3
PRO	189	0.1	TYR	373	-0.1	ASN	557	-0.3	PRO	189	0.1	TYR	373	-0.1	ASN	557	-0.4
ASP	190	-3.5	ARG	374	1.3	LEU	558	1.0	ASP	190	-3.5	ARG	374	1.4	LEU	558	1.0
PRO	191	-0.2	ASN	375	0.8	VAL	559	-0.5	PRO	191	-0.2	ASN	375	0.9	VAL	559	-0.5
GLN	192	0.7	ARG	376	2.8	LYS	560	-1.1	GLN	192	0.7	ARG	376	2.8	LYS	560	-1.1
GLY	193	-0.5	ILE	377	1.0	THR	561	-0.3	GLY	193	-0.5	ILE	377	1.0	THR	561	-0.3
THR	194	0.0	ALA	378	-0.8	ALA	562	-0.2	THR	194	0.0	ALA	378	-0.8	ALA	562	-0.2
ASN	195	-1.3	MET	379	-0.7	THR	563	-0.3	ASN	195	-1.3	MET	379	-0.6	THR	563	-0.3
LEU	196	1.9	GLU	380	-6.6	LEU	564	4.1	LEU	196	1.9	GLU	380	-6.6	LEU	564	4.1
MET	197	-0.9	PHE	381	-0.5	LYS	565	-0.5	MET	197	-0.9	PHE	381	-0.5	LYS	565	-0.5
PHE	198	-0.4	ASN	382	-1.5	LYS	566	-0.4	PHE	198	-0.4	ASN	382	-1.5	LYS	566	-0.4
ALA	199	-1.0	GLN	383	-1.6	LEU	567	3.2	ALA	199	-0.8	GLN	383	-1.6	LEU	567	3.2
PHE	200	-1.2	LEU	384	8.2	VAL	568	0.2	PHE	200	-1.2	LEU	384	8.4	VAL	568	0.2
PHE	201	-0.9	TYR	385	-15.5	CYS	569	0.1	PHE	201	-0.9	TYR	385	-18.2	CYS	569	0.1
ALA	202	0.0	HIS	386	0.4	LEU	570	1.5	ALA	202	0.3	HIS	386	0.4	LEU	570	1.5
GLN	203	-0.4	TRP	387	0.7	ASN	571	0.3	GLN	203	-0.3	TRP	387	0.2	ASN	571	0.3
HIS	204	-0.1	HIS	388	-0.8	THR	572	0.1	HIS	204	-0.5	HIS	388	-1.0	THR	572	0.1
PHE	205	1.6	PRO	389	0.2	LYS	573	-0.1	PHE	205	1.5	PRO	389	0.2	LYS	573	-0.1
THR	206	3.6	LEU	390	6.2	THR	574	-0.2	THR	206	3.5	LEU	390	6.2	THR	574	-0.2
HIS	207	1.6	MET	391	0.2	CYS	575	-0.2	HIS	207	-0.3	MET	391	0.1	CYS	575	-0.2

GLN	208	1.9	PRO	392	0.1	PRO	576	0.0	GLN	208	1.9	PRO	392	0.1	PRO	576	0.0
PHE	209	1.9	ASP	393	-3.3	TYR	577	-0.2	PHE	209	1.9	ASP	393	-3.3	TYR	577	-0.2
PHE	210	1.7	SER	394	-0.1	VAL	578	-0.3	PHE	210	1.8	SER	394	-0.1	VAL	578	-0.3
LYS	211	-0.4	PHE	395	0.3	SER	579	-0.5	LYS	211	-0.5	PHE	395	0.3	SER	579	-0.2
THR	212	0.4	ARG	396	2.6	PHE	580	0.6	THR	212	0.4	ARG	396	2.6	PHE	580	0.7
SER	213	0.2	VAL	397	0.0	HIS	581	1.7	SER	213	0.2	VAL	397	0.0	HIS	581	1.8
GLY	214	0.2	GLY	398	0.1	VAL	582	0.1	GLY	214	0.2	GLY	398	0.1	VAL	582	0.1
LYS	215	0.6	PRO	399	0.0				LYS	215	0.6	PRO	399	0.0			
MET	216	0.2	GLN	400	-0.2				MET	216	0.2	GLN	400	-0.2			

Table S2. Electrostatic interaction energies Ees [kJ/mol] for amino acid residues of chain A and B of the COX-2 protein with the FLP.

**Chain A**									**Chain B**								
**aa**	**NO**	**kJ**	**aa**	**NO**	**kJ**	**aa**	**NO**	**kJ**	**aa**	**NO**	**kJ**	**aa**	**NO**	**kJ**	**aa**	**NO**	**kJ**
ASN	34	0.5	PRO	218	-0.3	TYR	402	0.3	ASN	34	0.4	PRO	218	-0.3	TYR	402	0.3
PRO	35	1.0	GLY	219	-0.4	SER	403	0.5	PRO	35	1.0	GLY	219	-0.5	SER	403	0.5
CYS	36	1.4	PHE	220	1.4	PHE	404	-1.2	CYS	36	1.4	PHE	220	1.3	PHE	404	-1.3
CYS	37	1.1	THR	221	-2.1	LYS	405	-41.3	CYS	37	1.2	THR	221	-2.1	LYS	405	-41.2
SER	38	1.6	ARG	222	-48.9	GLN	406	1.0	SER	38	1.6	ARG	222	-48.8	GLN	406	1.0
ASN	39	2.0	GLY	223	0.5	PHE	407	-0.7	ASN	39	2.0	GLY	223	0.5	PHE	407	-0.7
PRO	40	1.0	LEU	224	1.5	LEU	408	-1.2	PRO	40	1.0	LEU	224	1.4	LEU	408	-1.3
CYS	41	1.3	GLY	225	1.9	TYR	409	-0.6	CYS	41	1.3	GLY	225	1.8	TYR	409	-0.6
GLN	42	2.2	HIS	226	4.7	ASN	410	0.1	GLN	42	2.3	HIS	226	4.7	ASN	410	0.2
ASN	43	2.5	GLY	227	-0.9	ASN	411	0.2	ASN	43	2.4	GLY	227	-0.8	ASN	411	0.2
ARG	44	-81.0	VAL	228	-3.7	SER	412	0.2	ARG	44	-80.5	VAL	228	-3.6	SER	412	0.2
GLY	45	-0.1	ASP	229	59.2	ILE	413	-0.1	GLY	45	-0.2	ASP	229	58.5	ILE	413	0.0
GLU	46	51.8	LEU	230	-2.9	LEU	414	-0.8	GLU	46	51.7	LEU	230	-2.8	LEU	414	-0.8
CYS	47	-0.9	ASN	231	-2.6	LEU	415	-0.3	CYS	47	-1.0	ASN	231	-2.6	LEU	415	-0.3
MET	48	-0.8	HIS	232	-4.2	GLU	416	34.4	MET	48	-1.0	HIS	232	-4.1	GLU	416	34.3
SER	49	-0.5	ILE	233	-2.1	HIS	417	0.6	SER	49	-0.5	ILE	233	-2.0	HIS	417	0.7
THR	50	-0.5	TYR	234	-0.8	GLY	418	0.3	THR	50	-0.5	TYR	234	-0.9	GLY	418	0.3
GLY	51	-0.5	GLY	235	0.0	LEU	419	0.4	GLY	51	-0.5	GLY	235	0.0	LEU	419	0.5
PHE	52	0.5	GLU	236	47.7	THR	420	1.0	PHE	52	0.5	GLU	236	47.5	THR	420	0.9
ASP	53	33.3	THR	237	-0.2	GLN	421	1.5	ASP	53	33.4	THR	237	-0.2	GLN	421	1.6
GLN	54	0.9	LEU	238	-0.4	PHE	422	1.4	GLN	54	0.9	LEU	238	-0.4	PHE	422	1.4
TYR	55	-0.5	ASP	239	35.5	VAL	423	0.6	TYR	55	-0.5	ASP	239	35.6	VAL	423	0.7
LYS	56	-39.7	ARG	240	-42.3	GLU	424	44.7	LYS	56	-39.4	ARG	240	-42.1	GLU	424	44.9
CYS	57	0.9	GLN	241	-1.7	SER	425	1.2	CYS	57	0.9	GLN	241	-1.6	SER	425	1.2
ASP	58	48.9	HIS	242	-0.6	PHE	426	2.1	ASP	58	48.5	HIS	242	-0.6	PHE	426	2.2
CYS	59	0.9	LYS	243	-35.1	THR	427	1.4	CYS	59	0.8	LYS	243	-35.0	THR	427	1.8
THR	60	1.5	LEU	244	-0.4	ARG	428	-50.3	THR	60	1.5	LEU	244	-0.5	ARG	428	-50.8
ARG	61	-51.3	ARG	245	-39.0	GLN	429	1.1	ARG	61	-50.8	ARG	245	-38.6	GLN	429	1.1

THR	62	1.4	LEU	246	-0.7	ILE	430	0.0	THR	62	1.4	LEU	246	-0.7	ILE	430	0.0
GLY	63	-0.4	PHE	247	-0.7	ALA	431	-0.3	GLY	63	-0.4	PHE	247	-0.7	ALA	431	-0.3
PHE	64	-1.7	LYS	248	-30.9	GLY	432	3.3	PHE	64	-1.7	LYS	248	-30.7	GLY	432	3.4
TYR	65	0.6	ASP	249	34.6	ARG	433	-77.2	TYR	65	0.6	ASP	249	34.4	ARG	433	-79.3
GLY	66	0.6	GLY	250	0.9	VAL	434	-4.2	GLY	66	0.6	GLY	250	0.9	VAL	434	-4.5
GLU	67	40.7	LYS	251	-36.1	ALA	435	-4.6	GLU	67	40.9	LYS	251	-35.9	ALA	435	-5.0
ASN	68	1.3	LEU	252	-1.2	GLY	436	-2.9	ASN	68	1.4	LEU	252	-1.2	GLY	436	-3.1
CYS	69	1.9	LYS	253	-33.7	GLY	437	-1.5	CYS	69	2.0	LYS	253	-33.5	GLY	437	-1.7
THR	70	0.4	TYR	254	-0.6	ARG	438	-49.7	THR	70	0.4	TYR	254	-0.8	ARG	438	-50.6
THR	71	1.0	GLN	255	-0.5	ASN	439	-2.8	THR	71	0.9	GLN	255	-0.5	ASN	439	-2.9
PRO	72	-1.5	VAL	256	-0.3	VAL	440	-1.7	PRO	72	-1.5	VAL	256	-0.2	VAL	440	-1.7
GLU	73	51.3	ILE	257	0.0	PRO	441	-1.0	GLU	73	51.1	ILE	257	0.0	PRO	441	-1.1
PHE	74	2.0	GLY	258	0.0	ILE	442	-0.3	PHE	74	1.9	GLY	258	-0.1	ILE	442	-0.3
LEU	75	1.8	GLY	259	0.3	ALA	443	0.0	LEU	75	1.8	GLY	259	0.3	ALA	443	0.0
THR	76	0.8	GLU	260	37.7	VAL	444	0.0	THR	76	0.7	GLU	260	37.7	VAL	444	0.0
ARG	77	-46.3	VAL	261	0.6	GLN	445	1.0	ARG	77	-46.5	VAL	261	0.6	GLN	445	1.0
ILE	78	2.8	TYR	262	0.3	ALA	446	1.0	ILE	78	2.8	TYR	262	0.2	ALA	446	1.0
LYS	79	-64.5	PRO	263	-1.3	VAL	447	0.9	LYS	79	-63.4	PRO	263	-1.3	VAL	447	0.9
LEU	80	1.5	PRO	264	0.2	ALA	448	0.1	LEU	80	1.5	PRO	264	0.2	ALA	448	0.1
LEU	81	1.2	THR	265	0.4	LYS	449	-43.3	LEU	81	1.2	THR	265	0.4	LYS	449	-43.4
LEU	82	3.3	VAL	266	-0.6	ALA	450	1.0	LEU	82	3.3	VAL	266	-0.6	ALA	450	1.0
LYS	83	-102.2	LYS	267	-30.0	SER	451	-0.7	LYS	83	-102.5	LYS	267	-30.2	SER	451	0.8
PRO	84	1.2	ASP	268	31.7	ILE	452	-0.5	PRO	84	1.8	ASP	268	31.7	ILE	452	-0.6
THR	85	8.0	THR	269	-0.2	ASP	453	46.7	THR	85	8.5	THR	269	-0.2	ASP	453	46.9
PRO	86	1.7	GLN	270	0.2	GLN	454	1.8	PRO	86	2.3	GLN	270	0.3	GLN	454	1.9
ASN	87	2.4	VAL	271	-0.3	SER	455	-0.1	ASN	87	2.4	VAL	271	-0.4	SER	455	-0.2
THR	88	3.6	GLU	272	36.2	ARG	456	-49.1	THR	88	3.6	GLU	272	36.3	ARG	456	-49.7
VAL	89	-0.6	MET	273	0.2	GLU	457	46.2	VAL	89	-0.2	MET	273	0.2	GLU	457	46.4
HIS	90	-15.5	ILE	274	-0.3	MET	458	0.3	HIS	90	-16.6	ILE	274	-0.3	MET	458	0.0
TYR	91	0.2	TYR	275	-0.3	LYS	459	-43.8	TYR	91	0.3	TYR	275	-0.3	LYS	459	-44.0
ILE	92	1.8	PRO	276	-0.8	TYR	460	-2.1	ILE	92	2.1	PRO	276	-0.8	TYR	460	-2.2
LEU	93	-11.2	PRO	277	-0.4	GLN	461	2.0	LEU	93	-11.0	PRO	277	-0.4	GLN	461	2.0
THR	94	-4.2	HIS	278	-0.8	SER	462	3.5	THR	94	-4.4	HIS	278	-0.8	SER	462	3.5
HIS	95	-0.4	ILE	279	-0.9	LEU	463	2.0	HIS	95	-2.7	ILE	279	-0.9	LEU	463	1.9
PHE	96	0.6	PRO	280	-0.3	ASN	464	4.3	PHE	96	0.6	PRO	280	-0.3	ASN	464	4.3
LYS	97	-70.7	GLU	281	30.3	GLU	465	64.9	LYS	97	-71.1	GLU	281	30.2	GLU	465	65.5
GLY	98	0.5	ASN	282	0.5	TYR	466	2.6	GLY	98	0.4	ASN	282	0.5	TYR	466	2.6
VAL	99	1.0	LEU	283	0.8	ARG	467	-87.6	VAL	99	1.0	LEU	283	0.8	ARG	467	-89.7
TRP	100	-4.2	GLN	284	0.1	LYS	468	-59.3	TRP	100	-4.2	GLN	284	0.1	LYS	468	-60.1
ASN	101	-2.5	PHE	285	0.3	ARG	469	-83.9	ASN	101	-3.5	PHE	285	0.3	ARG	469	-83.8
ILE	102	1.3	ALA	286	1.1	PHE	470	3.3	ILE	102	1.2	ALA	286	1.1	PHE	470	3.3
VAL	103	-0.7	VAL	287	0.7	SER	471	1.1	VAL	103	-0.7	VAL	287	0.7	SER	471	1.4
ASN	104	-0.8	GLY	288	0.6	LEU	472	-6.8	ASN	104	-1.1	GLY	288	0.6	LEU	472	-7.0
ASN	105	-0.8	GLN	289	-3.1	LYS	473	-71.2	ASN	105	-2.5	GLN	289	-3.0	LYS	473	-72.5

PRO	106	3.1	GLU	290	43.5	PRO	474	-0.5	PRO	106	3.0	GLU	290	43.5	PRO	474	-0.5
PHE	107	5.0	VAL	291	-0.2	TYR	475	-2.6	PHE	107	4.9	VAL	291	-0.3	TYR	475	-2.7
LEU	108	5.6	PHE	292	-1.5	THR	476	-2.2	LEU	108	5.4	PHE	292	-1.4	THR	476	-2.2
ARG	109	-87.0	GLY	293	-0.3	SER	477	1.7	ARG	109	-85.7	GLY	293	-0.3	SER	477	1.8
SER	110	3.8	LEU	294	0.2	PHE	478	2.0	SER	110	3.7	LEU	294	0.2	PHE	478	2.0
LEU	111	7.3	VAL	295	0.0	GLU	479	52.9	LEU	111	6.8	VAL	295	0.0	GLU	479	53.9
ILE	112	9.5	PRO	296	1.3	GLU	480	66.3	ILE	112	8.6	PRO	296	1.3	GLU	480	67.7
MET	113	10.7	GLY	297	1.0	LEU	481	2.2	MET	113	10.3	GLY	297	0.9	LEU	481	2.3
LYS	114	-75.3	LEU	298	-0.5	THR	482	0.3	LYS	114	-74.2	LEU	298	-0.6	THR	482	0.2
TYR	115	7.7	MET	299	-0.2	GLY	483	-1.1	TYR	115	7.3	MET	299	-0.3	GLY	483	-1.2
VAL	116	-5.0	MET	300	0.3	GLU	484	52.7	VAL	116	-3.8	MET	300	0.2	GLU	484	53.7
LEU	117	0.1	TYR	301	-1.0	LYS	485	-47.1	LEU	117	0.1	TYR	301	-0.9	LYS	485	-48.2
THR	118	6.3	ALA	302	-0.9	GLU	486	44.7	THR	118	6.2	ALA	302	-1.0	GLU	486	45.2
SER	119	8.5	THR	303	-0.4	MET	487	-0.2	SER	119	8.6	THR	303	-0.4	MET	487	-0.1
ARG	120	-372.9	ILE	304	-0.5	ALA	488	-1.2	ARG	120	-371.0	ILE	304	-0.6	ALA	488	-1.3
SER	121	-9.0	TRP	305	-3.2	ALA	489	-0.4	SER	121	-8.0	TRP	305	-3.3	ALA	489	-0.4
TYR	122	-1.7	LEU	306	-0.1	GLU	490	44.6	TYR	122	-1.3	LEU	306	-0.1	GLU	490	45.3
LEU	123	-7.6	ARG	307	-43.1	LEU	491	-1.7	LEU	123	-7.2	ARG	307	-42.9	LEU	491	-1.8
ILE	124	-6.3	GLU	308	50.2	LYS	492	-44.1	ILE	124	-6.2	GLU	308	49.7	LYS	492	-44.6
ASP	125	74.5	HIS	309	-0.4	ALA	493	-0.2	ASP	125	73.6	HIS	309	-0.3	ALA	493	-0.2
SER	126	-2.6	GLN	310	0.2	LEU	494	-0.3	SER	126	-2.6	GLN	310	0.1	LEU	494	-0.3
PRO	127	1.3	ARG	311	-47.4	TYR	495	-0.4	PRO	127	1.4	ARG	311	-47.1	TYR	495	-0.5
PRO	128	-1.9	VAL	312	-1.5	SER	496	-0.1	PRO	128	-1.9	VAL	312	-1.4	SER	496	-0.1
THR	129	1.3	CYS	313	-4.0	ASP	497	48.5	THR	129	0.6	CYS	313	-3.9	ASP	497	49.6
TYR	130	0.6	ASP	314	37.3	ILE	498	0.7	TYR	130	0.6	ASP	314	37.0	ILE	498	0.7
ASN	131	-1.2	ILE	315	-0.9	ASP	499	53.9	ASN	131	-1.1	ILE	315	-0.9	ASP	499	54.1
VAL	132	-1.4	LEU	316	-1.3	VAL	500	0.9	VAL	132	-1.3	LEU	316	-1.3	VAL	500	1.0
HIS	133	-0.8	LYS	317	-33.6	MET	501	0.0	HIS	133	-0.7	LYS	317	-33.3	MET	501	-0.2
TYR	134	0.8	GLN	318	-0.2	GLU	502	57.4	TYR	134	0.8	GLN	318	-0.2	GLU	502	58.1
GLY	135	0.5	GLU	319	36.8	LEU	503	0.5	GLY	135	0.5	GLU	319	36.7	LEU	503	0.6
TYR	136	0.4	HIS	320	-1.6	TYR	504	1.9	TYR	136	0.2	HIS	320	-1.5	TYR	504	2.0
LYS	137	-51.1	PRO	321	-0.1	PRO	505	2.5	LYS	137	-50.9	PRO	321	-0.2	PRO	505	2.6
SER	138	1.2	GLU	322	33.6	ALA	506	2.9	SER	138	1.1	GLU	322	33.3	ALA	506	3.1
TRP	139	0.9	TRP	323	-1.0	LEU	507	0.2	TRP	139	0.9	TRP	323	-1.0	LEU	507	0.2
GLU	140	43.9	GLY	324	0.5	LEU	508	-0.5	GLU	140	43.7	GLY	324	0.5	LEU	508	-0.2
ALA	141	1.1	ASP	325	36.5	VAL	509	1.3	ALA	141	1.0	ASP	325	36.3	VAL	509	1.4
PHE	142	0.4	GLU	326	38.9	GLU	510	95.3	PHE	142	0.4	GLU	326	38.5	GLU	510	98.1
SER	143	0.0	GLN	327	0.3	LYS	511	-68.0	SER	143	0.0	GLN	327	0.3	LYS	511	-69.5
ASN	144	2.4	LEU	328	1.5	PRO	512	2.8	ASN	144	2.3	LEU	328	1.4	PRO	512	3.0
LEU	145	0.8	PHE	329	1.2	ARG	513	-145.2	LEU	145	0.8	PHE	329	1.2	ARG	513	-152.7
SER	146	1.2	GLN	330	2.6	PRO	514	-1.7	SER	146	1.2	GLN	330	2.5	PRO	514	-1.7
TYR	147	1.7	THR	331	1.6	ASP	515	77.1	TYR	147	1.6	THR	331	1.5	ASP	515	78.6
TYR	148	0.0	SER	332	1.7	ALA	516	-3.3	TYR	148	-0.1	SER	332	1.6	ALA	516	-3.6
THR	149	-0.5	LYS	333	-48.5	ILE	517	-2.5	THR	149	-0.6	LYS	333	-48.0	ILE	517	-2.7

ARG	150	-60.8	LEU	334	2.3	PHE	518	-5.8	ARG	150	-61.3	LEU	334	2.2	PHE	518	-6.3
ALA	151	-2.4	ILE	335	2.7	GLY	519	8.3	ALA	151	-2.5	ILE	335	2.7	GLY	519	8.9
LEU	152	-1.9	LEU	336	3.3	GLU	520	120.0	LEU	152	-1.9	LEU	336	3.1	GLU	520	123.7
PRO	153	0.3	ILE	337	3.4	THR	521	8.6	PRO	153	0.4	ILE	337	3.4	THR	521	9.0
PRO	154	-0.3	GLY	338	2.8	MET	522	10.7	PRO	154	-0.3	GLY	338	2.9	MET	522	2.2
VAL	155	-1.1	GLU	339	64.1	VAL	523	9.6	VAL	155	-1.2	GLU	339	63.4	VAL	523	-1.9
ALA	156	-0.4	THR	340	4.4	GLU	524	196.1	ALA	156	-0.5	THR	340	4.3	GLU	524	203.7
ASP	157	40.9	ILE	341	4.8	LEU	525	11.4	ASP	157	41.0	ILE	341	4.7	LEU	525	10.7
ASP	158	37.3	LYS	342	-74.0	GLY	526	8.4	ASP	158	37.5	LYS	342	-73.3	GLY	526	5.1
CYS	159	0.7	ILE	343	7.5	ALA	527	-34.7	CYS	159	0.6	ILE	343	7.4	ALA	527	-44.8
PRO	160	0.1	VAL	344	10.1	PRO	528	-7.8	PRO	160	0.1	VAL	344	9.8	PRO	528	-8.7
THR	161	0.8	ILE	345	4.1	PHE	529	0.1	THR	161	0.8	ILE	345	4.3	PHE	529	-1.2
PRO	162	1.4	GLU	346	94.7	SER	530	-20.9	PRO	162	1.4	GLU	346	92.9	SER	530	-15.3
MET	163	1.7	ASP	347	94.1	LEU	531	-32.6	MET	163	1.7	ASP	347	94.0	LEU	531	-27.4
GLY	164	-0.9	TYR	348	7.9	LYS	532	-103.5	GLY	164	-0.9	TYR	348	7.8	LYS	532	-100.8
VAL	165	-0.6	VAL	349	-4.7	GLY	533	-4.7	VAL	165	-0.5	VAL	349	-1.1	GLY	533	-4.7
LYS	166	-47.2	GLN	350	-0.2	LEU	534	-8.1	LYS	166	-47.5	GLN	350	0.4	LEU	534	-7.8
GLY	167	-0.8	HIS	351	8.2	MET	535	-6.9	GLY	167	-0.8	HIS	351	9.6	MET	535	-6.2
ASN	168	0.2	LEU	352	0.6	GLY	536	-4.1	ASN	168	0.2	LEU	352	1.1	GLY	536	-3.7
LYS	169	-34.9	SER	353	-12.4	ASN	537	-3.7	LYS	169	-35.1	SER	353	-11.2	ASN	537	-3.7
GLU	170	37.6	GLY	354	-3.8	PRO	538	0.3	GLU	170	37.9	GLY	354	-4.0	PRO	538	0.2
LEU	171	-1.4	TYR	355	-88.3	ILE	539	-0.1	LEU	171	-1.4	TYR	355	-118.7	ILE	539	-0.1
PRO	172	0.0	HIS	356	-1.1	CYS	540	-6.8	PRO	172	0.0	HIS	356	-1.4	CYS	540	-6.6
ASP	173	41.5	PHE	357	-0.4	SER	541	-2.3	ASP	173	42.0	PHE	357	-0.1	SER	541	-2.2
SER	174	-0.2	LYS	358	-76.7	PRO	542	-1.7	SER	174	-0.1	LYS	358	-76.1	PRO	542	-1.6
LYS	175	-41.0	LEU	359	-19.8	GLN	543	0.6	LYS	175	-41.2	LEU	359	-15.7	GLN	543	0.5
GLU	176	41.0	LYS	360	-83.6	TYR	544	-0.6	GLU	176	41.3	LYS	360	-81.5	TYR	544	-0.7
VAL	177	0.4	PHE	361	-2.8	TRP	545	-2.3	VAL	177	0.5	PHE	361	-2.7	TRP	545	-2.3
LEU	178	0.4	ASP	362	78.1	LYS	546	-47.6	LEU	178	0.4	ASP	362	76.0	LYS	546	-46.9
GLU	179	41.7	PRO	363	0.2	PRO	547	-0.3	GLU	179	42.1	PRO	363	0.2	PRO	547	-0.3
LYS	180	-39.7	GLU	364	65.3	SER	548	0.7	LYS	180	-40.3	GLU	364	63.7	SER	548	0.7
VAL	181	1.3	LEU	365	-2.1	THR	549	-1.1	VAL	181	1.4	LEU	365	-1.9	THR	549	-1.1
LEU	182	0.0	LEU	366	-5.2	PHE	550	-1.6	LEU	182	0.0	LEU	366	-4.8	PHE	550	-1.6
LEU	183	0.8	PHE	367	-1.3	GLY	551	-0.5	LEU	183	0.8	PHE	367	-1.2	GLY	551	-0.5
ARG	184	-56.1	ASN	368	1.7	GLY	552	0.7	ARG	184	-57.0	ASN	368	1.8	GLY	552	0.7
ARG	185	-45.6	GLN	369	-3.2	GLU	553	48.3	ARG	185	-46.4	GLN	369	-2.7	GLU	553	47.6
GLU	186	48.4	GLN	370	5.5	VAL	554	1.5	GLU	186	49.1	GLN	370	5.3	VAL	554	1.5
PHE	187	0.1	PHE	371	-4.0	GLY	555	1.7	PHE	187	0.1	PHE	371	-3.7	GLY	555	1.7
ILE	188	1.6	GLN	372	1.4	PHE	556	1.4	ILE	188	1.6	GLN	372	1.3	PHE	556	1.4
PRO	189	2.2	TYR	373	-0.4	LYS	557	-46.2	PRO	189	2.3	TYR	373	-0.3	LYS	557	-45.8
ASP	190	86.5	GLN	374	3.1	ILE	558	2.9	ASP	190	88.4	GLN	374	2.7	ILE	558	2.8
PRO	191	0.6	ASN	375	0.1	ILE	559	2.3	PRO	191	0.7	ASN	375	0.4	ILE	559	2.2
GLN	192	-6.9	ARG	376	-59.5	ASN	560	3.4	GLN	192	-7.1	ARG	376	-58.7	ASN	560	3.4
GLY	193	-0.7	ILE	377	-1.4	THR	561	0.3	GLY	193	-0.9	ILE	377	-1.3	THR	561	0.3

SER	194	-3.4	ALA	378	-0.8	ALA	562	2.4	SER	194	-3.3	ALA	378	-0.9	ALA	562	2.3
ASN	195	2.5	SER	379	3.0	SER	563	-1.3	ASN	195	2.7	SER	379	3.1	SER	563	-1.3
MET	196	1.7	GLU	380	82.9	ILE	564	-3.7	MET	196	1.8	GLU	380	83.4	ILE	564	-3.7
MET	197	0.8	PHE	381	-6.4	GLN	565	-2.4	MET	197	0.7	PHE	381	-5.4	GLN	565	-2.4
PHE	198	-3.0	ASN	382	1.0	SER	566	-2.3	PHE	198	-3.3	ASN	382	1.2	SER	566	-2.3
ALA	199	0.1	THR	383	1.3	LEU	567	-2.6	ALA	199	-0.1	THR	383	1.3	LEU	567	-2.6
PHE	200	2.1	LEU	384	-1.2	ILE	568	-2.3	PHE	200	2.1	LEU	384	-1.6	ILE	568	-2.4
PHE	201	0.0	TYR	385	-7.4	CYS	569	-1.2	PHE	201	0.0	TYR	385	-8.0	CYS	569	-1.2
ALA	202	-2.4	HIS	386	-0.9	ASN	570	-2.5	ALA	202	-2.5	HIS	386	-0.5	ASN	570	-2.5
GLN	203	0.4	TRP	387	-12.0	ASN	571	-2.3	GLN	203	0.3	TRP	387	-24.2	ASN	571	-2.3
HIS	204	2.2	HIS	388	-1.7	VAL	572	-1.1	HIS	204	2.3	HIS	388	-1.5	VAL	572	-1.0
PHE	205	-2.8	PRO	389	0.5	LYS	573	-36.2	PHE	205	-2.7	PRO	389	0.6	LYS	573	-36.2
THR	206	-5.0	LEU	390	-3.5	GLY	574	0.2	THR	206	-5.1	LEU	390	-3.6	GLY	574	0.2
HIS	207	-2.3	LEU	391	-1.1	CYS	575	1.2	HIS	207	-2.2	LEU	391	-1.1	CYS	575	1.3
GLN	208	-3.6	PRO	392	-1.0	PRO	576	1.4	GLN	208	-3.5	PRO	392	-1.0	PRO	576	1.4
PHE	209	-3.4	ASP	393	47.4	PHE	577	0.5	PHE	209	-3.4	ASP	393	48.0	PHE	577	0.5
PHE	210	-4.4	THR	394	1.0	THR	578	0.3	PHE	210	-4.4	THR	394	1.1	THR	578	0.3
LYS	211	-55.4	PHE	395	-1.3	SER	579	1.7	LYS	211	-55.1	PHE	395	-1.3	SER	579	1.8
THR	212	-1.1	ASN	396	-0.6	PHE	580	-0.6	THR	212	-1.1	ASN	396	-0.7	PHE	580	-0.3
ASP	213	44.3	ILE	397	-1.0	ASN	581	-0.8	ASP	213	44.2	ILE	397	-1.0	ASN	581	-0.2
HIS	214	-2.5	GLU	398	42.4	VAL	582	-0.2	HIS	214	-2.4	GLU	398	42.3	VAL	582	-0.1
LYS	215	-44.2	ASP	399	34.8				LYS	215	-44.1	ASP	399	34.9			
ARG	216	-42.7	GLN	400	1.6				ARG	216	-42.5	GLN	400	1.6			
GLY	217	1.0	GLU	401	40.6				GLY	217	1.0	GLU	401	40.9			

Table S3. Electrostatic interaction energies Ees [kJ/mol] for amino acid residues of chain C and D of the COX-2 protein with the FLP.

				Chain C									Chain D				
aa	NO	kJ	aa	NO	kJ	aa	NO	kJ	aa	NO	kJ	aa	NO	kJ	aa	NO	kJ
ASN	34	0.4	PRO	218	-0.3	TYR	402	0.6	ASN	34	0.5	GLY	217	1.0	GLN	400	1.6
PRO	35	1.0	GLY	219	-0.4	SER	403	0.5	PRO	35	1.0	PRO	218	-0.3	GLU	401	41.0
CYS	36	1.4	PHE	220	1.3	PHE	404	-1.2	CYS	36	1.4	GLY	219	-0.5	TYR	402	0.3
CYS	37	1.1	THR	221	-2.3	LYS	405	-41.4	CYS	37	1.2	PHE	220	1.3	SER	403	0.5
SER	38	1.6	ARG	222	-49.7	GLN	406	1.0	SER	38	1.7	THR	221	-2.2	PHE	404	-1.3
ASN	39	2.0	GLY	223	0.6	PHE	407	-0.7	ASN	39	2.1	ARG	222	-48.8	LYS	405	-41.0
PRO	40	1.0	LEU	224	1.5	LEU	408	-1.2	PRO	40	1.1	GLY	223	0.5	GLN	406	1.0
CYS	41	1.3	GLY	225	1.9	TYR	409	-0.3	CYS	41	1.3	LEU	224	1.5	PHE	407	-0.7
GLN	42	-0.8	HIS	226	4.7	ASN	410	0.1	GLN	42	2.4	GLY	225	1.8	LEU	408	-1.2
ASN	43	2.5	GLY	227	-0.9	ASN	411	0.2	ASN	43	2.4	HIS	226	4.7	TYR	409	-0.6
ARG	44	-81.2	VAL	228	-3.9	SER	412	0.2	ARG	44	-81.2	GLY	227	-0.8	ASN	410	0.1
GLY	45	-0.1	ASP	229	60.2	ILE	413	0.0	GLY	45	-0.1	VAL	228	-3.2	ASN	411	0.3
GLU	46	52.1	LEU	230	-3.0	LEU	414	-0.9	GLU	46	52.0	ASP	229	58.7	SER	412	0.2
CYS	47	-0.9	ASN	231	-2.6	LEU	415	-0.3	CYS	47	-0.9	LEU	230	-2.8	ILE	413	0.0
MET	48	-1.3	HIS	232	-4.5	GLU	416	34.4	MET	48	-1.0	ASN	231	-2.5	LEU	414	-0.8

SER	49	-0.5	ILE	233	-2.1	HIS	417	0.6	SER	49	-0.5	HIS	232	-4.2	LEU	415	-0.3
THR	50	-0.5	TYR	234	-0.6	GLY	418	0.3	THR	50	-0.5	ILE	233	-2.1	GLU	416	34.2
GLY	51	-0.5	GLY	235	0.0	LEU	419	0.5	GLY	51	-0.5	TYR	234	-0.8	HIS	417	0.7
PHE	52	0.5	GLU	236	48.4	THR	420	0.8	PHE	52	0.5	GLY	235	0.0	GLY	418	0.3
ASP	53	33.4	THR	237	-0.2	GLN	421	1.6	ASP	53	33.4	GLU	236	47.5	LEU	419	0.5
GLN	54	1.0	LEU	238	-0.4	PHE	422	1.4	GLN	54	1.0	THR	237	-0.1	THR	420	0.8
TYR	55	-0.3	ASP	239	36.0	VAL	423	0.6	TYR	55	-0.6	LEU	238	-0.4	GLN	421	1.5
LYS	56	-39.8	ARG	240	-42.8	GLU	424	44.8	LYS	56	-39.4	ASP	239	35.6	PHE	422	1.4
CYS	57	0.9	GLN	241	-1.6	SER	425	1.1	CYS	57	0.8	ARG	240	-42.1	VAL	423	-8.0
ASP	58	49.0	HIS	242	-0.7	PHE	426	2.1	ASP	58	49.0	GLN	241	-1.6	GLU	424	44.5
CYS	59	0.9	LYS	243	-35.6	THR	427	1.4	CYS	59	0.9	HIS	242	-0.7	SER	425	1.1
THR	60	1.5	LEU	244	-0.5	ARG	428	-50.7	THR	60	1.6	LYS	243	-35.3	PHE	426	2.2
ARG	61	-51.4	ARG	245	-39.2	GLN	429	1.1	ARG	61	-51.4	LEU	244	-0.5	THR	427	1.5
THR	62	1.4	LEU	246	-0.7	ILE	430	0.0	THR	62	1.5	ARG	245	-38.8	ARG	428	-50.2
GLY	63	-0.5	PHE	247	-0.7	ALA	431	-0.3	GLY	63	-0.4	LEU	246	-0.7	GLN	429	1.1
PHE	64	-1.7	LYS	248	-31.2	GLY	432	3.4	PHE	64	-1.8	PHE	247	-0.7	ILE	430	0.0
TYR	65	0.8	ASP	249	34.8	ARG	433	-77.6	TYR	65	0.5	LYS	248	-30.7	ALA	431	-0.3
GLY	66	0.6	GLY	250	0.9	VAL	434	-4.4	GLY	66	0.6	ASP	249	34.4	GLY	432	3.3
GLU	67	40.8	LYS	251	-36.4	ALA	435	-4.8	GLU	67	40.9	GLY	250	0.8	ARG	433	-78.5
ASN	68	1.4	LEU	252	-1.2	GLY	436	-3.0	ASN	68	1.4	LYS	251	-35.9	VAL	434	-3.7
CYS	69	1.9	LYS	253	-34.0	GLY	437	-1.6	CYS	69	2.0	LEU	252	-1.2	ALA	435	-4.6
THR	70	0.4	TYR	254	-0.4	ARG	438	-49.8	THR	70	0.4	LYS	253	-33.5	GLY	436	-2.9
THR	71	1.0	GLN	255	-0.5	ASN	439	-2.7	THR	71	1.0	TYR	254	-0.6	GLY	437	-1.7
PRO	72	-1.5	VAL	256	-0.3	VAL	440	-1.8	PRO	72	-1.6	GLN	255	-0.5	ARG	438	-50.1
GLU	73	51.1	ILE	257	0.0	PRO	441	-1.1	GLU	73	51.5	VAL	256	-0.1	ASN	439	-2.8
PHE	74	1.9	GLY	258	-0.1	ILE	442	-0.2	PHE	74	2.0	ILE	257	0.0	VAL	440	-1.4
LEU	75	1.7	GLY	259	0.3	ALA	443	0.0	LEU	75	1.8	GLY	258	0.0	PRO	441	-1.1
THR	76	0.7	GLU	260	38.0	VAL	444	0.0	THR	76	0.7	GLY	259	0.3	ILE	442	-0.2
ARG	77	-46.2	VAL	261	0.6	GLN	445	1.0	ARG	77	-46.7	GLU	260	37.5	ALA	443	0.0
ILE	78	2.7	TYR	262	0.5	ALA	446	1.1	ILE	78	2.9	VAL	261	0.8	VAL	444	0.3
LYS	79	-63.5	PRO	263	-1.3	VAL	447	0.9	LYS	79	-64.2	TYR	262	0.3	GLN	445	1.0
LEU	80	1.4	PRO	264	0.2	ALA	448	0.0	LEU	80	1.5	PRO	263	-1.3	ALA	446	1.0
LEU	81	1.1	THR	265	0.4	LYS	449	-43.7	LEU	81	1.2	PRO	264	0.2	VAL	447	1.1
LEU	82	3.1	VAL	266	-0.6	ALA	450	1.0	LEU	82	3.3	THR	265	0.4	ALA	448	0.1
LYS	83	-99.9	LYS	267	-30.3	SER	451	0.8	LYS	83	-103.5	VAL	266	-0.4	LYS	449	-43.5
PRO	84	1.4	ASP	268	32.1	ILE	452	-0.5	PRO	84	1.6	LYS	267	-30.1	ALA	450	1.0
THR	85	7.8	THR	269	-0.2	ASP	453	47.1	THR	85	8.4	ASP	268	31.8	SER	451	0.8
PRO	86	1.4	GLN	270	0.3	GLN	454	1.9	PRO	86	1.9	THR	269	-0.2	ILE	452	-0.5
ASN	87	2.2	VAL	271	-0.4	SER	455	-0.1	ASN	87	2.2	GLN	270	0.3	ASP	453	46.8
THR	88	3.3	GLU	272	36.8	ARG	456	-49.6	THR	88	7.3	VAL	271	-0.2	GLN	454	1.9
VAL	89	-0.5	MET	273	0.2	GLU	457	46.8	VAL	89	0.2	GLU	272	36.3	SER	455	-0.1
HIS	90	2.2	ILE	274	-0.3	MET	458	0.2	HIS	90	-16.2	MET	273	0.2	ARG	456	-49.5
TYR	91	0.6	TYR	275	-0.1	LYS	459	-44.2	TYR	91	0.2	ILE	274	-0.3	GLU	457	46.3
ILE	92	1.9	PRO	276	-0.8	TYR	460	-1.7	ILE	92	1.9	TYR	275	-0.3	MET	458	0.2

LEU	93	-9.8	PRO	277	-0.4	GLN	461	2.0	LEU	93	-11.3	PRO	276	-0.8	LYS	459	-44.1
THR	94	-1.8	HIS	278	0.7	SER	462	3.6	THR	94	-4.1	PRO	277	-0.4	TYR	460	-1.8
HIS	95	-0.5	ILE	279	-0.9	LEU	463	2.0	HIS	95	-0.9	HIS	278	-0.8	GLN	461	2.0
PHE	96	0.6	PRO	280	-0.3	ASN	464	4.4	PHE	96	0.4	ILE	279	-0.9	SER	462	3.6
LYS	97	-69.8	GLU	281	30.5	GLU	465	65.3	LYS	97	-70.5	PRO	280	-0.3	LEU	463	2.0
GLY	98	0.5	ASN	282	0.5	TYR	466	3.0	GLY	98	0.5	GLU	281	30.2	ASN	464	4.4
VAL	99	1.0	LEU	283	0.8	ARG	467	-87.6	VAL	99	1.3	ASN	282	0.4	GLU	465	65.6
TRP	100	-3.8	GLN	284	0.0	LYS	468	-59.5	TRP	100	-4.1	LEU	283	0.8	TYR	466	2.7
ASN	101	-2.9	PHE	285	0.3	ARG	469	-84.6	ASN	101	-2.6	GLN	284	0.1	ARG	467	-89.4
ILE	102	1.3	ALA	286	1.1	PHE	470	3.0	ILE	102	1.2	PHE	285	0.3	LYS	468	-59.9
VAL	103	-0.5	VAL	287	0.8	SER	471	1.1	VAL	103	-0.3	ALA	286	1.1	ARG	469	-84.1
ASN	104	-0.9	GLY	288	0.6	LEU	472	-6.8	ASN	104	-1.1	VAL	287	0.9	PHE	470	3.4
ASN	105	-0.7	GLN	289	-3.2	LYS	473	-71.4	ASN	105	-2.4	GLY	288	0.6	SER	471	1.4
PRO	106	3.1	GLU	290	44.2	PRO	474	-0.6	PRO	106	3.0	GLN	289	-3.0	LEU	472	-6.9
PHE	107	5.0	VAL	291	-0.3	TYR	475	-2.2	PHE	107	5.0	GLU	290	43.4	LYS	473	-73.0
LEU	108	5.6	PHE	292	-1.5	THR	476	-2.1	LEU	108	5.6	VAL	291	0.0	PRO	474	-0.6
ARG	109	-86.5	GLY	293	-0.3	SER	477	1.7	ARG	109	-86.2	PHE	292	-1.4	TYR	475	-2.6
SER	110	3.7	LEU	294	0.2	PHE	478	1.9	SER	110	3.7	GLY	293	-0.3	THR	476	-2.3
LEU	111	7.2	VAL	295	0.0	GLU	479	52.8	LEU	111	7.1	LEU	294	0.2	SER	477	1.8
ILE	112	9.4	PRO	296	1.3	GLU	480	65.9	ILE	112	9.1	VAL	295	0.2	PHE	478	1.9
MET	113	10.4	GLY	297	1.0	LEU	481	2.1	MET	113	10.7	PRO	296	1.3	GLU	479	53.5
LYS	114	-75.2	LEU	298	-0.6	THR	482	0.9	LYS	114	-74.7	GLY	297	0.9	GLU	480	67.0
TYR	115	9.0	MET	299	-0.3	GLY	483	-1.1	TYR	115	7.6	LEU	298	-0.6	LEU	481	2.3
VAL	116	-2.4	MET	300	0.2	GLU	484	52.7	VAL	116	-3.4	MET	299	-0.2	THR	482	0.1
LEU	117	-0.3	TYR	301	-0.7	LYS	485	-47.0	LEU	117	0.2	MET	300	0.3	GLY	483	-1.1
THR	118	6.2	ALA	302	-1.0	GLU	486	45.1	THR	118	6.4	TYR	301	-1.0	GLU	484	53.3
SER	119	8.7	THR	303	-0.4	MET	487	-0.1	SER	119	9.0	ALA	302	-1.0	LYS	485	-47.5
ARG	120	-351.5	ILE	304	-0.6	ALA	488	-1.2	ARG	120	-415.0	THR	303	-0.4	GLU	486	45.0
SER	121	-7.3	TRP	305	-3.3	ALA	489	-0.4	SER	121	-8.4	ILE	304	-0.6	MET	487	-0.1
TYR	122	-0.9	LEU	306	-0.1	GLU	490	45.0	TYR	122	-1.5	TRP	305	-3.2	ALA	488	-1.2
LEU	123	-7.4	ARG	307	-43.4	LEU	491	-1.7	LEU	123	-7.4	LEU	306	-0.1	ALA	489	-0.4
ILE	124	-6.6	GLU	308	50.5	LYS	492	-43.9	ILE	124	-6.2	ARG	307	-42.8	GLU	490	45.0
ASP	125	75.2	HIS	309	-0.5	ALA	493	-0.2	ASP	125	74.4	GLU	308	49.7	LEU	491	-1.7
SER	126	-2.6	GLN	310	0.1	LEU	494	-0.3	SER	126	-2.7	HIS	309	-0.4	LYS	492	-44.3
PRO	127	1.4	ARG	311	-47.7	TYR	495	-0.3	PRO	127	1.4	GLN	310	0.1	ALA	493	-0.2
PRO	128	-2.0	VAL	312	-1.5	SER	496	-0.1	PRO	128	-1.9	ARG	311	-47.0	LEU	494	-0.3
THR	129	0.7	CYS	313	-4.0	ASP	497	48.9	THR	129	1.2	VAL	312	-1.2	TYR	495	-0.5
TYR	130	1.0	ASP	314	37.5	ILE	498	0.8	TYR	130	0.6	CYS	313	-3.9	SER	496	-0.1
ASN	131	-1.3	ILE	315	-0.9	ASP	499	53.8	ASN	131	-1.2	ASP	314	37.0	ASP	497	49.2
VAL	132	-1.4	LEU	316	-1.3	VAL	500	1.0	VAL	132	-1.1	ILE	315	-0.9	ILE	498	0.7
HIS	133	-1.0	LYS	317	-34.1	MET	501	-0.1	HIS	133	-0.7	LEU	316	-1.2	ASP	499	54.0
TYR	134	1.1	GLN	318	-0.3	GLU	502	58.0	TYR	134	0.8	LYS	317	-33.6	VAL	500	1.2
GLY	135	0.5	GLU	319	37.1	LEU	503	0.5	GLY	135	0.6	GLN	318	-0.2	MET	501	-0.1
TYR	136	0.7	HIS	320	-1.7	TYR	504	2.4	TYR	136	0.5	GLU	319	36.9	GLU	502	57.9

LYS	137	-51.6	PRO	321	-0.2	PRO	505	2.4	LYS	137	-51.1	HIS	320	-1.6	LEU	503	0.4
SER	138	1.2	GLU	322	33.8	ALA	506	3.1	SER	138	1.1	PRO	321	-0.2	TYR	504	2.0
TRP	139	1.0	TRP	323	-1.0	LEU	507	0.1	TRP	139	1.0	GLU	322	33.3	PRO	505	2.5
GLU	140	44.4	GLY	324	0.5	LEU	508	-0.5	GLU	140	43.9	TRP	323	-1.0	ALA	506	3.1
ALA	141	1.1	ASP	325	36.9	VAL	509	1.3	ALA	141	1.1	GLY	324	0.5	LEU	507	0.0
PHE	142	0.4	GLU	326	39.3	GLU	510	95.5	PHE	142	0.4	ASP	325	36.3	LEU	508	-0.3
SER	143	0.0	GLN	327	0.3	LYS	511	-67.4	SER	143	-0.1	GLU	326	38.6	VAL	509	1.7
ASN	144	2.5	LEU	328	1.5	PRO	512	2.6	ASN	144	2.3	GLN	327	0.3	GLU	510	97.0
LEU	145	0.9	PHE	329	1.3	ARG	513	-143.0	LEU	145	0.8	LEU	328	1.4	LYS	511	-68.9
SER	146	1.2	GLN	330	2.6	PRO	514	-1.6	SER	146	1.2	PHE	329	1.2	PRO	512	3.0
TYR	147	2.0	THR	331	1.6	ASP	515	76.4	TYR	147	1.6	GLN	330	2.5	ARG	513	-149.4
TYR	148	0.3	SER	332	1.7	ALA	516	-3.0	TYR	148	-0.1	THR	331	1.5	PRO	514	-1.9
THR	149	-0.6	LYS	333	-49.3	ILE	517	-2.3	THR	149	-0.5	SER	332	1.6	ASP	515	77.1
ARG	150	-61.8	LEU	334	2.4	PHE	518	-6.2	ARG	150	-61.2	LYS	333	-48.2	ALA	516	-3.4
ALA	151	-2.6	ILE	335	2.8	GLY	519	8.6	ALA	151	-2.4	LEU	334	2.3	ILE	517	-2.3
LEU	152	-1.8	LEU	336	3.3	GLU	520	119.3	LEU	152	-2.0	ILE	335	2.7	PHE	518	-5.8
PRO	153	0.3	ILE	337	3.5	THR	521	8.9	PRO	153	0.4	LEU	336	3.2	GLY	519	8.9
PRO	154	-0.4	GLY	338	2.9	MET	522	12.0	PRO	154	-0.3	ILE	337	3.3	GLU	520	122.8
VAL	155	-1.1	GLU	339	64.8	VAL	523	11.8	VAL	155	-0.8	GLY	338	2.9	THR	521	8.9
ALA	156	-0.4	THR	340	4.7	GLU	524	193.9	ALA	156	-0.5	GLU	339	63.3	MET	522	7.4
ASP	157	41.4	ILE	341	4.9	LEU	525	11.7	ASP	157	40.9	THR	340	4.4	VAL	523	6.3
ASP	158	37.5	LYS	342	-75.0	GLY	526	8.1	ASP	158	37.5	ILE	341	4.8	GLU	524	203.2
CYS	159	0.7	ILE	343	7.5	ALA	527	-32.9	CYS	159	0.7	LYS	342	-73.6	LEU	525	11.3
PRO	160	0.2	VAL	344	10.0	PRO	528	-6.5	PRO	160	0.1	ILE	343	7.3	GLY	526	5.0
THR	161	0.8	ILE	345	3.5	PHE	529	0.5	THR	161	0.8	VAL	344	10.3	ALA	527	-44.4
PRO	162	1.4	GLU	346	94.6	SER	530	-22.0	PRO	162	1.4	ILE	345	4.0	PRO	528	-9.0
MET	163	1.7	ASP	347	93.9	LEU	531	-36.3	MET	163	1.7	GLU	346	93.4	PHE	529	-0.9
GLY	164	-0.9	TYR	348	7.6	LYS	532	-106.0	GLY	164	-0.9	ASP	347	93.8	SER	530	-17.1
VAL	165	-0.6	VAL	349	-12.9	GLY	533	-4.9	VAL	165	-0.3	TYR	348	7.4	LEU	531	-31.4
LYS	166	-47.2	GLN	350	0.1	LEU	534	-8.9	LYS	166	-47.5	VAL	349	0.9	LYS	532	-102.4
GLY	167	-0.8	HIS	351	7.9	MET	535	-7.3	GLY	167	-0.8	GLN	350	0.3	GLY	533	-4.6
ASN	168	0.2	LEU	352	-0.4	GLY	536	-4.2	ASN	168	0.2	HIS	351	8.8	LEU	534	-7.9
LYS	169	-35.1	SER	353	-11.7	ASN	537	-3.8	LYS	169	-35.3	LEU	352	1.1	MET	535	-6.5
GLU	170	37.9	GLY	354	-4.2	PRO	538	0.2	GLU	170	37.7	SER	353	-10.0	GLY	536	-3.8
LEU	171	-1.4	TYR	355	-61.2	ILE	539	-0.2	LEU	171	-1.4	GLY	354	-3.7	ASN	537	-3.6
PRO	172	0.0	HIS	356	-1.3	CYS	540	-7.0	PRO	172	-0.1	TYR	355	-97.1	PRO	538	0.2
ASP	173	42.0	PHE	357	0.2	SER	541	-2.4	ASP	173	41.6	HIS	356	-1.5	ILE	539	-0.2
SER	174	-0.2	LYS	358	-75.6	PRO	542	-1.6	SER	174	-0.2	PHE	357	-0.2	CYS	540	-6.7
LYS	175	-41.6	LEU	359	-17.2	GLN	543	-1.1	LYS	175	-40.9	LYS	358	-76.5	SER	541	-2.3
GLU	176	41.1	LYS	360	-81.3	TYR	544	-9.3	GLU	176	41.2	LEU	359	-15.4	PRO	542	-1.7
VAL	177	0.5	PHE	361	-2.9	TRP	545	-2.3	VAL	177	0.8	LYS	360	-81.5	GLN	543	0.5
LEU	178	0.4	ASP	362	78.4	LYS	546	-47.9	LEU	178	0.4	PHE	361	-2.7	TYR	544	-0.6
GLU	179	41.9	PRO	363	0.0	PRO	547	-0.3	GLU	179	41.8	ASP	362	77.0	TRP	545	-2.3
LYS	180	-40.1	GLU	364	65.4	SER	548	0.7	LYS	180	-40.1	PRO	363	0.3	LYS	546	-47.2

VAL	181	1.3	LEU	365	-2.2	THR	549	-1.1	VAL	181	1.5	GLU	364	64.6	PRO	547	-0.3
LEU	182	0.0	LEU	366	-5.3	PHE	550	-1.6	LEU	182	0.0	LEU	365	-2.1	SER	548	0.7
LEU	183	0.7	PHE	367	-1.4	GLY	551	-0.6	LEU	183	0.8	LEU	366	-5.0	THR	549	-1.1
ARG	184	-56.5	ASN	368	2.0	GLY	552	0.8	ARG	184	-56.3	PHE	367	-1.3	PHE	550	-1.6
ARG	185	-45.9	GLN	369	-3.1	GLU	553	48.4	ARG	185	-46.0	ASN	368	1.8	GLY	551	-0.5
GLU	186	48.8	GLN	370	5.5	VAL	554	1.5	GLU	186	48.7	GLN	369	-2.9	GLY	552	0.7
PHE	187	0.1	PHE	371	-4.0	GLY	555	1.7	PHE	187	0.1	GLN	370	5.4	GLU	553	47.8
ILE	188	1.7	GLN	372	1.6	PHE	556	1.4	ILE	188	1.5	PHE	371	-3.8	VAL	554	1.8
PRO	189	2.1	TYR	373	0.0	LYS	557	-46.2	PRO	189	2.2	GLN	372	1.3	GLY	555	1.7
ASP	190	86.5	GLN	374	3.2	ILE	558	2.9	ASP	190	86.8	TYR	373	-0.5	PHE	556	1.4
PRO	191	0.6	ASN	375	0.3	ILE	559	2.3	PRO	191	0.6	GLN	374	2.8	LYS	557	-45.9
GLN	192	-6.5	ARG	376	-60.5	ASN	560	3.3	GLN	192	-6.9	ASN	375	0.4	ILE	558	2.8
GLY	193	-0.7	ILE	377	-1.6	THR	561	0.2	GLY	193	-0.8	ARG	376	-59.1	ILE	559	2.3
SER	194	-3.2	ALA	378	-0.8	ALA	562	2.3	SER	194	-3.2	ILE	377	-1.3	ASN	560	3.5
ASN	195	2.7	SER	379	3.1	SER	563	-1.3	ASN	195	2.7	ALA	378	-0.8	THR	561	0.3
MET	196	1.9	GLU	380	84.9	ILE	564	-3.8	MET	196	1.7	SER	379	3.2	ALA	562	2.3
MET	197	0.9	PHE	381	-7.4	GLN	565	-2.4	MET	197	0.7	GLU	380	83.5	SER	563	-1.4
PHE	198	-3.0	ASN	382	1.1	SER	566	-2.3	PHE	198	-3.2	PHE	381	-6.2	ILE	564	-3.6
ALA	199	0.1	THR	383	1.4	LEU	567	-2.6	ALA	199	-0.1	ASN	382	1.1	GLN	565	-2.3
PHE	200	2.2	LEU	384	-1.5	ILE	568	-2.3	PHE	200	2.1	THR	383	1.4	SER	566	-2.2
PHE	201	0.1	TYR	385	-29.0	CYS	569	-1.2	PHE	201	0.0	LEU	384	-1.5	LEU	567	-2.6
ALA	202	-2.2	HIS	386	-0.9	ASN	570	-2.5	ALA	202	-2.5	TYR	385	-7.8	ILE	568	-2.4
GLN	203	0.3	TRP	387	-15.3	ASN	571	-2.3	GLN	203	0.4	HIS	386	-0.7	CYS	569	-1.2
HIS	204	2.1	HIS	388	-1.9	VAL	572	-1.0	HIS	204	2.2	TRP	387	-14.4	ASN	570	-2.5
PHE	205	-2.9	PRO	389	0.5	LYS	573	-36.5	PHE	205	-2.6	HIS	388	-1.7	ASN	571	-2.3
THR	206	-5.3	LEU	390	-3.7	GLY	574	0.2	THR	206	-4.9	PRO	389	0.5	VAL	572	-0.8
HIS	207	-2.5	LEU	391	-1.2	CYS	575	1.2	HIS	207	-2.3	LEU	390	-3.5	LYS	573	-36.1
GLN	208	-3.7	PRO	392	-1.0	PRO	576	1.3	GLN	208	-3.4	LEU	391	-1.1	GLY	574	0.2
PHE	209	-3.6	ASP	393	47.7	PHE	577	0.5	PHE	209	-3.4	PRO	392	-1.0	CYS	575	1.2
PHE	210	-4.7	THR	394	1.1	THR	578	0.3	PHE	210	-4.4	ASP	393	47.5	PRO	576	1.3
LYS	211	-56.6	PHE	395	-1.3	SER	579	1.8	LYS	211	-55.1	THR	394	1.0	PHE	577	0.5
THR	212	-1.1	ASN	396	-0.6	PHE	580	-0.5	THR	212	-1.1	PHE	395	-1.3	THR	578	0.4
ASP	213	44.9	ILE	397	-1.1	ASN	581	-0.7	ASP	213	44.2	ASN	396	-0.6	SER	579	1.7
HIS	214	-2.7	GLU	398	42.4	VAL	582	-0.3	HIS	214	-2.5	ILE	397	-1.0	PHE	580	-0.3
LYS	215	-44.9	ASP	399	35.0				LYS	215	-44.1	GLU	398	42.2	ASN	581	-6.4
ARG	216	-43.3	GLN	400	1.5				ARG	216	-42.6	ASP	399	34.8	VAL	582	0.2
GLY	217	1.0	GLU	401	40.7												

Table S4. Electrostatic interaction energies E_{es} [kJ/mol] for amino acid residues of chain A and B of the COX-1 protein with the IBP.

Chain A									Chain B								
**aa**	**NO**	**kJ**	**aa**	**NO**	**kJ**	**aa**	**NO**	**kJ**	**aa**	**NO**	**kJ**	**aa**	**NO**	**kJ**	**aa**	**NO**	**kJ**
ASN	34	0.2	GLY	217	-0.2	GLN	400	-0.2	ASN	34	0.2	GLY	217	-0.2	GLN	400	-0.3
PRO	35	0.0	PRO	218	0.2	ASP	401	-3.8	PRO	35	0.0	PRO	218	0.2	ASP	401	-3.8

CYS	36	0.0	GLY	219	0.2	TYR	402	-0.1	CYS	36	0.0	GLY	219	0.2	TYR	402	-0.1
CYS	37	0.0	PHE	220	-0.5	SER	403	-0.2	CYS	37	0.0	PHE	220	-0.5	SER	403	-0.2
TYR	38	0.1	THR	221	0.1	TYR	404	0.0	TYR	38	0.1	THR	221	0.3	TYR	404	-0.1
TYR	39	0.2	LYS	222	-0.6	GLU	405	-3.8	TYR	39	0.2	LYS	222	-0.6	GLU	405	-3.9
PRO	40	0.3	ALA	223	0.2	GLN	406	-0.2	PRO	40	0.3	ALA	223	0.2	GLN	406	-0.2
CYS	41	0.1	LEU	224	1.3	PHE	407	0.1	CYS	41	0.1	LEU	224	1.4	PHE	407	0.0
GLN	42	0.4	GLY	225	-0.3	LEU	408	2.2	GLN	42	-0.2	GLY	225	-0.3	LEU	408	2.2
HIS	43	0.3	HIS	226	-0.8	PHE	409	0.1	HIS	43	0.3	HIS	226	-0.8	PHE	409	0.1
GLN	44	-1.1	GLY	227	-0.1	ASN	410	0.0	GLN	44	-1.1	GLY	227	-0.2	ASN	410	0.0
GLY	45	-0.3	VAL	228	0.7	THR	411	0.1	GLY	45	-0.3	VAL	228	0.6	THR	411	0.1
ILE	46	-0.1	ASP	229	-6.7	SER	412	-0.1	ILE	46	-0.1	ASP	229	-6.8	SER	412	-0.1
CYS	47	-0.2	LEU	230	3.6	MET	413	-0.2	CYS	47	-0.2	LEU	230	3.5	MET	413	-0.2
VAL	48	0.0	GLY	231	0.2	LEU	414	2.0	VAL	48	0.0	GLY	231	0.2	LEU	414	2.0
ARG	49	-0.2	HIS	232	0.9	VAL	415	0.0	ARG	49	-0.2	HIS	232	1.0	VAL	415	0.0
PHE	50	-0.1	ILE	233	0.5	ASP	416	-3.0	PHE	50	-0.1	ILE	233	0.5	ASP	416	-3.1
GLY	51	0.0	TYR	234	0.2	TYR	417	0.1	GLY	51	0.0	TYR	234	0.2	TYR	417	0.0
LEU	52	-0.1	GLY	235	0.1	GLY	418	-0.2	LEU	52	-0.1	GLY	235	0.1	GLY	418	-0.2
ASP	53	0.6	ASP	236	-4.7	VAL	419	-0.3	ASP	53	0.6	ASP	236	-4.7	VAL	419	-0.3
ARG	54	-0.8	ASN	237	0.3	GLU	420	-3.8	ARG	54	-0.8	ASN	237	0.2	GLU	420	-3.8
TYR	55	0.0	LEU	238	1.1	ALA	421	-0.2	TYR	55	0.0	LEU	238	1.0	ALA	421	-0.2
GLN	56	0.1	GLU	239	-2.6	LEU	422	1.7	GLN	56	0.1	GLU	239	-2.6	LEU	422	1.7
CYS	57	0.1	ARG	240	4.3	VAL	423	-0.3	CYS	57	0.1	ARG	240	4.4	VAL	423	-0.3
ASP	58	2.5	GLN	241	0.3	ASP	424	-4.3	ASP	58	2.5	GLN	241	0.3	ASP	424	-4.4
CYS	59	0.2	TYR	242	0.0	ALA	425	-0.3	CYS	59	0.2	TYR	242	0.0	ALA	425	-0.3
THR	60	0.3	GLN	243	0.0	PHE	426	-0.3	THR	60	0.3	GLN	243	0.0	PHE	426	-0.4
ARG	61	-3.2	LEU	244	1.6	SER	427	-0.1	ARG	61	-3.2	LEU	244	1.6	SER	427	-0.1
THR	62	0.6	ARG	245	2.8	ARG	428	4.0	THR	62	0.6	ARG	245	2.8	ARG	428	4.0
GLY	63	0.0	LEU	246	1.0	GLN	429	-0.5	GLY	63	0.0	LEU	246	1.1	GLN	429	-0.5
TYR	64	-0.1	PHE	247	0.1	PRO	430	-0.4	TYR	64	-0.1	PHE	247	0.1	PRO	430	-0.4
SER	65	-0.1	LYS	248	-0.2	ALA	431	0.5	SER	65	-0.1	LYS	248	-0.2	ALA	431	0.5
GLY	66	0.0	ASP	249	-2.0	GLY	432	-0.9	GLY	66	0.0	ASP	249	-2.0	GLY	432	-0.9
PRO	67	0.0	GLY	250	-0.2	ARG	433	5.5	PRO	67	0.0	GLY	250	-0.2	ARG	433	5.5
ASN	68	0.1	LYS	251	-0.2	ILE	434	2.4	ASN	68	0.1	LYS	251	-0.3	ILE	434	2.4
CYS	69	0.2	LEU	252	1.6	GLY	435	1.1	CYS	69	0.2	LEU	252	1.6	GLY	435	1.0
THR	70	0.2	LYS	253	-0.3	GLY	436	0.4	THR	70	0.2	LYS	253	-0.3	GLY	436	0.4
ILE	71	0.3	TYR	254	0.1	GLY	437	0.3	ILE	71	0.3	TYR	254	0.1	GLY	437	0.3
PRO	72	-0.2	GLN	255	0.1	ARG	438	3.0	PRO	72	-0.2	GLN	255	0.1	ARG	438	3.0
GLU	73	5.1	MET	256	-0.1	ASN	439	0.3	GLU	73	5.2	MET	256	-0.1	ASN	439	0.3
ILE	74	0.5	LEU	257	1.1	ILE	440	0.5	ILE	74	0.5	LEU	257	1.1	ILE	440	0.5
TRP	75	0.6	ASN	258	0.1	ASP	441	-5.4	TRP	75	0.7	ASN	258	0.1	ASP	441	-5.4
THR	76	0.5	GLY	259	-0.1	HIS	442	2.5	THR	76	0.5	GLY	259	-0.1	HIS	442	0.2
TRP	77	0.7	GLU	260	-2.9	HIS	443	-1.2	TRP	77	0.7	GLU	260	-3.0	HIS	443	-1.2
LEU	78	-2.0	VAL	261	-0.3	ILE	444	0.0	LEU	78	-2.0	VAL	261	-0.3	ILE	444	0.0
ARG	79	-10.7	TYR	262	0.0	LEU	445	2.0	ARG	79	-10.7	TYR	262	0.0	LEU	445	2.0

THR	80	0.0	PRO	263	0.2	HIS	446	0.7	THR	80	-0.1	PRO	263	0.2	HIS	446	0.7
THR	81	0.4	PRO	264	-0.1	VAL	447	-0.1	THR	81	0.4	PRO	264	-0.1	VAL	447	-0.1
LEU	82	-2.7	SER	265	0.0	ALA	448	0.4	LEU	82	-2.7	SER	265	0.0	ALA	448	0.4
ARG	83	-23.9	VAL	266	0.0	VAL	449	-0.2	ARG	83	-23.9	VAL	266	0.0	VAL	449	-0.2
PRO	84	1.2	GLU	267	-2.2	ASP	450	-5.2	PRO	84	1.2	GLU	267	-2.2	ASP	450	-5.2
SER	85	2.8	GLU	268	-2.3	VAL	451	0.3	SER	85	2.8	GLU	268	-2.3	VAL	451	0.3
PRO	86	0.5	ALA	269	-0.1	ILE	452	0.3	PRO	86	0.5	ALA	269	-0.1	ILE	452	0.3
SER	87	0.0	PRO	270	-0.1	LYS	453	-0.3	SER	87	0.0	PRO	270	-0.1	LYS	453	-0.3
PHE	88	1.1	VAL	271	-0.1	GLU	454	-6.4	PHE	88	1.2	VAL	271	-0.1	GLU	454	-6.4
ILE	89	-1.4	LEU	272	1.2	SER	455	0.4	ILE	89	-1.5	LEU	272	1.3	SER	455	0.6
HIS	90	1.5	MET	273	0.0	ARG	456	3.3	HIS	90	1.4	MET	273	0.0	ARG	456	3.2
PHE	91	-0.1	HIS	274	-0.3	VAL	457	0.1	PHE	91	-0.1	HIS	274	0.1	VAL	457	0.1
LEU	92	-7.1	TYR	275	0.1	LEU	458	2.3	LEU	92	-7.1	TYR	275	0.0	LEU	458	2.3
LEU	93	-16.8	PRO	276	0.1	ARG	459	1.8	LEU	93	-16.8	PRO	276	0.1	ARG	459	1.8
THR	94	-1.7	ARG	277	2.7	LEU	460	2.3	THR	94	-1.7	ARG	277	2.7	LEU	460	2.3
HIS	95	-0.6	GLY	278	0.0	GLN	461	-0.1	HIS	95	-0.6	GLY	278	0.0	GLN	461	-0.1
GLY	96	0.1	ILE	279	0.1	PRO	462	0.1	GLY	96	0.2	ILE	279	0.1	PRO	462	0.1
ARG	97	-2.2	PRO	280	-0.1	PHE	463	0.9	ARG	97	-2.0	PRO	280	-0.1	PHE	463	0.9
TRP	98	0.1	PRO	281	-0.1	ASN	464	0.7	TRP	98	0.2	PRO	281	-0.1	ASN	464	0.7
LEU	99	-2.6	GLN	282	-0.1	GLU	465	2.3	LEU	99	-2.5	GLN	282	-0.2	GLU	465	2.3
TRP	100	-1.4	SER	283	-0.1	TYR	466	1.4	TRP	100	-1.3	SER	283	-0.1	TYR	466	1.2
ASP	101	2.7	GLN	284	-0.1	ARG	467	-4.1	ASP	101	2.6	GLN	284	-0.1	ARG	467	-4.1
PHE	102	0.1	MET	285	-0.2	LYS	468	2.2	PHE	102	0.1	MET	285	-0.2	LYS	468	2.2
VAL	103	-0.2	ALA	286	-0.1	ARG	469	-3.7	VAL	103	-0.2	ALA	286	-0.1	ARG	469	-3.7
ASN	104	0.3	VAL	287	-0.3	PHE	470	3.2	ASN	104	0.4	VAL	287	-0.3	PHE	470	3.2
ALA	105	0.0	GLY	288	-0.1	GLY	471	0.6	ALA	105	0.0	GLY	288	-0.1	GLY	471	0.6
THR	106	0.3	GLN	289	0.8	MET	472	-0.7	THR	106	0.3	GLN	289	0.9	MET	472	-0.8
PHE	107	0.7	GLU	290	-5.7	LYS	473	0.0	PHE	107	0.8	GLU	290	-5.7	LYS	473	0.0
ILE	108	1.1	VAL	291	-0.2	PRO	474	0.1	ILE	108	1.1	VAL	291	-0.2	PRO	474	0.1
ARG	109	-2.2	PHE	292	0.4	TYR	475	0.1	ARG	109	-2.4	PHE	292	0.3	TYR	475	0.2
ASP	110	7.2	GLY	293	0.1	THR	476	-0.4	ASP	110	7.2	GLY	293	0.1	THR	476	-0.4
THR	111	1.6	LEU	294	2.1	SER	477	-0.1	THR	111	1.6	LEU	294	2.1	SER	477	-0.1
LEU	112	-3.8	LEU	295	3.0	PHE	478	0.1	LEU	112	-3.9	LEU	295	3.0	PHE	478	0.1
MET	113	3.4	PRO	296	-0.3	GLN	479	0.2	MET	113	3.0	PRO	296	-0.3	GLN	479	0.2
ARG	114	-7.5	GLY	297	-0.2	GLU	480	3.3	ARG	114	-7.8	GLY	297	-0.2	GLU	480	3.3
LEU	115	-6.0	LEU	298	3.8	LEU	481	0.2	LEU	115	-6.7	LEU	298	3.8	LEU	481	0.2
VAL	116	1.7	MET	299	0.0	THR	482	0.5	VAL	116	1.2	MET	299	0.0	THR	482	0.5
LEU	117	-5.5	LEU	300	2.6	GLY	483	0.1	LEU	117	-5.5	LEU	300	2.6	GLY	483	0.1
THR	118	1.5	TYR	301	0.5	GLU	484	-0.7	THR	118	1.5	TYR	301	0.5	GLU	484	-0.8
VAL	119	0.6	ALA	302	0.3	LYS	485	-0.5	VAL	119	0.4	ALA	302	0.3	LYS	485	-0.5
SER	121	-4.0	THR	303	0.2	GLU	486	-1.2	ARG	120	-262.2	THR	303	0.2	GLU	486	-1.2
ASN	122	-3.0	ILE	304	0.3	MET	487	-0.1	SER	121	-4.1	ILE	304	0.3	MET	487	-0.1
LEU	123	-10.3	TRP	305	0.9	ALA	488	0.0	ASN	122	-3.0	TRP	305	1.0	ALA	488	0.0
ILE	124	0.0	LEU	306	1.9	ALA	489	-0.1	LEU	123	-10.2	LEU	306	1.9	ALA	489	-0.1

PRO	125	-0.4	ARG	307	4.1	GLU	490	-1.7	ILE	124	0.0	ARG	307	4.1	GLU	490	-1.6
SER	126	0.1	GLU	308	-4.0	LEU	491	0.8	PRO	125	-0.5	GLU	308	-4.0	LEU	491	0.9
PRO	127	0.1	HIS	309	0.1	GLU	492	0.0	SER	126	0.1	HIS	309	0.1	GLU	492	0.0
PRO	128	0.4	ASN	310	0.1	GLU	493	-0.9	PRO	127	0.1	ASN	310	0.1	GLU	493	-0.9
THR	129	-0.1	ARG	311	3.3	LEU	494	0.7	PRO	128	0.4	ARG	311	3.3	LEU	494	0.7
TYR	130	-0.4	VAL	312	0.1	TYR	495	0.1	THR	129	-0.1	VAL	312	0.2	TYR	495	0.1
ASN	131	0.2	CYS	313	0.3	GLY	496	0.1	TYR	130	-0.4	CYS	313	0.3	GLY	496	0.1
ILE	132	0.3	ASP	314	-2.1	ASP	497	0.3	ASN	131	0.2	ASP	314	-2.1	ASP	497	0.3
ALA	133	0.2	LEU	315	1.0	ILE	498	-0.2	ILE	132	0.3	LEU	315	1.0	ILE	498	-0.2
HIS	134	0.0	LEU	316	1.0	ASP	499	0.9	ALA	133	0.2	LEU	316	1.0	ASP	499	0.9
ASP	135	-0.8	LYS	317	0.0	ALA	500	-0.3	HIS	134	0.0	LYS	317	0.0	ALA	500	-0.3
TYR	136	-0.1	ALA	318	0.1	LEU	501	0.6	ASP	135	-0.8	ALA	318	0.1	LEU	501	0.6
ILE	137	-0.1	GLU	319	-1.1	GLU	502	-3.5	TYR	136	-0.1	GLU	319	-1.1	GLU	502	-3.5
SER	138	-0.1	HIP	320	-2.8	PHE	503	-0.2	ILE	137	-0.2	HIP	320	-2.8	PHE	503	-0.3
TRP	139	-0.2	PRO	321	0.0	TYR	504	0.6	SER	138	-0.1	PRO	321	0.0	TYR	504	0.7
GLU	140	-1.7	THR	322	-0.1	PRO	505	-0.1	TRP	139	-0.2	THR	322	0.0	PRO	505	-0.1
SER	141	-0.3	TRP	323	-0.1	GLY	506	-0.2	GLU	140	-1.7	TRP	323	0.0	GLY	506	-0.2
PHE	142	-0.3	GLY	324	-0.1	LEU	507	4.7	SER	141	-0.3	GLY	324	-0.1	LEU	507	4.7
SER	143	-0.2	ASP	325	-2.2	LEU	508	5.1	PHE	142	-0.3	ASP	325	-2.2	LEU	508	5.1
ASN	144	-0.4	GLU	326	-2.2	LEU	509	2.2	SER	143	-0.2	GLU	326	-2.3	LEU	509	2.2
VAL	145	-0.5	GLN	327	-0.1	GLU	510	0.1	ASN	144	-0.4	GLN	327	-0.1	GLU	510	0.1
SER	146	-0.3	LEU	328	0.6	LYS	511	-1.5	VAL	145	-0.5	LEU	328	0.6	LYS	511	-1.5
TYR	147	-0.3	PHE	329	-0.1	CYS	512	0.6	SER	146	-0.3	PHE	329	-0.1	CYS	512	0.5
TYR	148	0.4	GLN	330	-0.3	HIS	513	-1.3	TYR	147	-0.3	GLN	330	-0.3	HIS	513	-1.2
THR	149	-0.1	THR	331	-0.2	PRO	514	-1.3	TYR	148	0.4	THR	331	-0.2	PRO	514	-1.3
ARG	150	3.1	ALA	332	-0.3	ASN	515	-0.4	THR	149	0.0	ALA	332	-0.3	ASN	515	-0.4
ILE	151	0.7	ARG	333	4.2	SER	516	-0.8	ARG	150	3.1	ARG	333	4.2	SER	516	-0.8
LEU	152	-0.5	LEU	334	0.9	ILE	517	0.9	ILE	151	0.8	LEU	334	0.9	ILE	517	0.9
PRO	153	-0.2	ILE	335	-0.5	PHE	518	5.7	LEU	152	-0.5	ILE	335	-0.5	PHE	518	5.6
SER	154	0.3	LEU	336	1.5	GLY	519	0.2	PRO	153	-0.2	LEU	336	1.5	GLY	519	0.3
VAL	155	0.0	ILE	337	-0.5	GLU	520	13.2	SER	154	0.3	ILE	337	-0.5	GLU	520	12.4
PRO	156	-0.1	GLY	338	-0.4	SER	521	-1.3	VAL	155	0.0	GLY	338	-0.4	SER	521	-1.4
ARG	157	1.6	GLU	339	-6.0	MET	522	-8.8	PRO	156	-0.1	GLU	339	-6.0	MET	522	-10.1
ASP	158	-0.3	THR	340	-1.0	ILE	523	3.8	ARG	157	1.6	THR	340	-1.0	ILE	523	5.8
CYS	159	0.0	ILE	341	-0.8	GLU	524	50.8	ASP	158	-0.3	ILE	341	-0.8	GLU	524	50.3
PRO	160	-0.1	LYS	342	-3.2	MET	525	-4.4	CYS	159	0.0	LYS	342	-3.2	MET	525	-4.1
THR	161	-0.1	ILE	343	-1.8	GLY	526	-2.6	PRO	160	-0.1	ILE	343	-1.8	GLY	526	-2.3
PRO	162	0.0	VAL	344	-2.3	ALA	527	-17.3	THR	161	-0.1	VAL	344	-2.4	ALA	527	-16.3
MET	163	0.1	ILE	345	-0.6	PRO	528	-7.2	PRO	162	0.0	ILE	345	-0.7	PRO	528	-7.2
GLY	164	0.1	GLU	346	-4.6	PHE	529	-1.2	MET	163	0.0	GLU	346	-4.6	PHE	529	-1.4
THR	165	0.0	GLU	347	-10.6	SER	530	5.1	GLY	164	0.1	GLU	347	-10.6	SER	530	2.7
LYS	166	-0.6	TYR	348	0.1	LEU	531	-9.6	THR	165	0.0	TYR	348	0.3	LEU	531	-8.8
GLY	167	0.0	VAL	349	-5.8	LYS	532	-3.0	LYS	166	-0.6	VAL	349	-8.4	LYS	532	-3.4
LYS	168	0.0	GLN	350	-0.8	GLY	533	1.2	GLY	167	0.0	GLN	350	-0.8	GLY	533	1.4

LYS	169	-0.4	GLN	351	0.5	LEU	534	10.4	LYS	168	0.0	GLN	351	0.3	LEU	534	10.5
GLN	170	0.1	LEU	352	26.6	LEU	535	-0.1	LYS	169	-0.3	LEU	352	26.5	LEU	535	-0.2
LEU	171	0.8	SER	353	8.3	GLY	536	0.1	GLN	170	0.1	SER	353	8.9	GLY	536	0.1
PRO	172	-0.2	GLY	354	3.1	ASN	537	1.6	LEU	171	0.8	GLY	354	2.9	ASN	537	1.6
ASP	173	-2.8	TYR	355	-69.7	PRO	538	0.3	PRO	172	-0.2	TYR	355	-83.8	PRO	538	0.3
ALA	174	-0.1	PHE	356	-0.2	ILE	539	0.6	ASP	173	-2.8	PHE	356	-0.2	ILE	539	0.6
GLU	175	-3.5	LEU	357	-8.6	CYS	540	0.5	ALA	174	-0.1	LEU	357	-8.5	CYS	540	0.5
PHE	176	-0.2	GLN	358	1.0	SER	541	0.1	GLU	175	-3.5	GLN	358	1.0	SER	541	0.1
LEU	177	1.1	LEU	359	1.1	PRO	542	-0.3	PHE	176	-0.2	LEU	359	-0.7	PRO	542	-0.3
SER	178	-0.2	LYS	360	-0.8	GLU	543	-0.1	LEU	177	1.1	LYS	360	-0.9	GLU	543	-0.1
ARG	179	3.3	PHE	361	0.6	TYR	544	-0.1	SER	178	-0.2	PHE	361	0.6	TYR	544	-0.1
ARG	180	2.3	ASP	362	2.5	TRP	545	0.1	ARG	179	3.3	ASP	362	2.5	TRP	545	0.1
PHE	181	-0.4	PRO	363	0.9	LYS	546	-0.4	ARG	180	2.2	PRO	363	0.9	LYS	546	-0.4
LEU	182	2.3	GLU	364	2.6	ALA	547	-0.2	PHE	181	-0.4	GLU	364	2.6	ALA	547	-0.2
LEU	183	1.7	LEU	365	-2.0	SER	548	-0.2	LEU	182	2.3	LEU	365	-2.1	SER	548	-0.2
ARG	184	6.8	LEU	366	-3.0	THR	549	-0.1	LEU	183	1.7	LEU	366	-3.0	THR	549	-0.1
ARG	185	3.3	PHE	367	0.3	PHE	550	0.0	ARG	184	6.9	PHE	367	0.3	PHE	550	0.0
LYS	186	-0.8	GLY	368	0.1	GLY	551	0.1	ARG	185	3.3	GLY	368	0.2	GLY	551	0.1
PHE	187	0.2	ALA	369	-0.5	GLY	552	0.0	LYS	186	-0.8	ALA	369	-0.5	GLY	552	0.0
ILE	188	-0.6	GLN	370	0.7	GLU	553	-0.7	PHE	187	0.2	GLN	370	0.7	GLU	553	-0.7
PRO	189	-0.2	PHE	371	-0.6	VAL	554	-0.2	ILE	188	-0.6	PHE	371	-0.6	VAL	554	-0.2
ASP	190	-10.4	GLN	372	-0.8	GLY	555	-0.1	PRO	189	-0.2	GLN	372	-0.8	GLY	555	-0.1
PRO	191	0.0	TYR	373	-0.2	PHE	556	-0.2	ASP	190	-10.5	TYR	373	-0.3	PHE	556	-0.2
GLN	192	1.4	ARG	374	0.7	ASN	557	-0.2	PRO	191	0.0	ARG	374	0.8	ASN	557	-0.2
GLY	193	-0.5	ASN	375	0.9	LEU	558	0.8	GLN	192	1.4	ASN	375	0.8	LEU	558	0.8
THR	194	0.3	ARG	376	2.6	VAL	559	-0.3	GLY	193	-0.5	ARG	376	2.6	VAL	559	-0.3
ASN	195	-1.7	ILE	377	1.0	LYS	560	-0.9	THR	194	0.6	ILE	377	1.0	LYS	560	-1.0
LEU	196	2.9	ALA	378	-0.8	THR	561	-0.4	ASN	195	-1.7	ALA	378	-0.7	THR	561	-0.4
MET	197	-0.9	MET	379	-0.7	ALA	562	-0.1	LEU	196	2.9	MET	379	-0.7	ALA	562	-0.1
PHE	198	0.6	GLU	380	-8.2	THR	563	-0.4	MET	197	-0.9	GLU	380	-8.0	THR	563	-0.4
ALA	199	-0.5	PHE	381	0.4	LEU	564	4.1	PHE	198	0.5	PHE	381	0.3	LEU	564	4.1
PHE	200	-0.9	ASN	382	-1.4	LYS	565	-0.4	ALA	199	-0.4	ASN	382	-1.5	LYS	565	-0.4
PHE	201	-0.7	GLN	383	-1.8	LYS	566	-0.7	PHE	200	-1.0	GLN	383	-1.8	LYS	566	-0.7
ALA	202	0.9	LEU	384	10.7	LEU	567	3.0	PHE	201	-0.9	LEU	384	10.8	LEU	567	3.0
GLN	203	0.6	TYR	385	-3.6	VAL	568	0.2	ALA	202	0.9	TYR	385	-3.9	VAL	568	0.2
HIS	204	-0.2	HIS	386	0.3	CYS	569	0.1	GLN	203	0.6	HIS	386	0.3	CYS	569	0.1
PHE	205	1.3	TRP	387	6.4	LEU	570	1.4	HIS	204	0.1	TRP	387	6.2	LEU	570	1.4
THR	206	3.2	HIS	388	-1.5	ASN	571	0.3	PHE	205	1.2	HIS	388	1.3	ASN	571	0.3
HIS	207	-0.1	PRO	389	0.3	THR	572	0.2	THR	206	3.1	PRO	389	0.3	THR	572	0.2
GLN	208	1.7	LEU	390	8.9	LYS	573	-0.1	HIS	207	0.0	LEU	390	8.9	LYS	573	-0.1
ARG	209	-221.9	MET	391	0.1	THR	574	-0.2	GLN	208	1.7	MET	391	0.1	THR	574	-0.2
PHE	209	1.6	PRO	392	0.2	CYS	575	-0.2	PHE	209	1.5	PRO	392	0.2	CYS	575	-0.2
PHE	210	1.7	ASP	393	-4.9	PRO	576	0.0	PHE	210	1.7	ASP	393	-4.9	PRO	576	0.0
LYS	211	-0.5	SER	394	-0.2	TYR	577	-0.2	LYS	211	-0.5	SER	394	-0.2	TYR	577	-0.2

THR	212	0.3	PHE	395	0.4	VAL	578	-0.3	THR	212	0.4	PHE	395	0.3	VAL	578	-0.3
SER	213	0.2	ARG	396	3.6	SER	579	-0.5	SER	213	0.2	ARG	396	3.6	SER	579	-0.8
GLY	214	0.2	VAL	397	0.0	PHE	580	-0.1	GLY	214	0.2	VAL	397	0.0	PHE	580	-0.1
LYS	215	0.6	GLY	398	0.1	HIS	581	0.7	LYS	215	0.6	GLY	398	0.1	HIS	581	0.6
MET	216	0.2	PRO	399	-0.1	VAL	582	0.2	MET	216	0.1	PRO	399	-0.1	VAL	582	0.2

Table S5. Electrostatic interaction energies E_{es} [kJ/mol] for amino acid residues of chain A and B of the COX-2 protein with the IBP.

Chain A									Chain B								
**aa**	**NO**	**kJ**	**aa**	**NO**	**kJ**	**aa**	**NO**	**kJ**	**aa**	**NO**	**kJ**	**aa**	**NO**	**kJ**	**aa**	**NO**	**kJ**
HIS	34	0.1	ARG	217	3.1	ASP	400	-2.9	HIS	34.0	0.1	ARG	217.0	3.1	ASP	400	-2.9
PRO	35	0.1	GLY	218	-0.2	GLN	401	-0.2	PRO	35.0	0.1	GLY	218	-0.2	GLN	401	-0.2
CYS	36	0.0	PRO	219	-0.3	GLU	402	-3.6	CYS	36.0	0.1	PRO	219	-0.3	GLU	402	-3.7
CYS	37	-0.1	GLY	220	0.0	TYR	403	-0.1	CYS	37.0	-0.1	GLY	220	0.0	TYR	403	-0.1
SER	38	0.1	PHE	221	-0.5	SER	404	-0.2	SER	38.0	0.1	PHE	221	-0.5	SER	404	-0.2
ASN	39	0.2	THR	222	0.3	PHE	405	0.3	ASN	39.0	0.3	THR	222	0.3	PHE	405	0.3
PRO	40	0.2	ARG	223	5.4	LYS	406	-0.5	PRO	40.0	0.3	ARG	223	5.1	LYS	406	-0.2
CYS	41	0.0	GLY	224	0.2	GLN	407	-0.2	CYS	41.0	0.0	GLY	224	0.2	GLN	407	-0.2
GLN	42	0.4	LEU	225	2.6	PHE	408	0.1	GLN	42.0	0.4	LEU	225	2.6	PHE	408	0.1
ASN	43	0.4	GLY	226	0.0	LEU	409	3.8	ASN	43.0	0.4	GLY	226	0.0	LEU	409	3.8
ARG	44	-7.0	HIS	227	-0.7	TYR	410	0.2	ARG	44.0	-7.2	HIS	227	-0.7	TYR	410	0.2
GLY	45	-0.3	GLY	228	-0.1	ASN	411	0.0	GLY	45.0	-0.3	GLY	228	-0.1	ASN	411	0.0
GLU	46	1.7	VAL	229	0.6	ASN	412	-0.1	GLU	46.0	1.8	VAL	229	0.6	ASN	412	-0.1
CYS	47	-0.1	ASP	230	-5.8	SER	413	0.0	CYS	47.0	-0.1	ASP	230	-5.8	SER	413	0.0
MET	48	0.0	LEU	231	4.9	ILE	414	0.1	MET	48.0	0.0	LEU	231	4.8	ILE	414	0.1
SER	49	0.1	ASN	232	0.2	LEU	415	3.1	SER	49.0	0.1	ASN	232	0.2	LEU	415	3.1
THR	50	-0.1	HIS	233	0.9	LEU	416	2.2	THR	50.0	-0.1	HIS	233	0.8	LEU	416	2.2
GLY	51	0.0	ILE	234	0.4	GLU	417	-2.8	GLY	51.0	0.0	ILE	234	0.4	GLU	417	-2.9
PHE	52	0.0	TYR	235	0.1	HIS	418	0.1	PHE	52.0	0.0	TYR	235	0.1	HIS	418	0.1
ASP	53	0.4	GLY	236	0.1	GLY	419	-0.1	ASP	53.0	0.5	GLY	236	0.1	GLY	419	-0.1
GLN	54	0.0	GLU	237	-4.6	LEU	420	2.8	GLN	54.0	0.0	GLU	237	-4.6	LEU	420	2.8
TYR	55	0.0	THR	238	0.0	THR	421	-0.2	TYR	55.0	0.0	THR	238	0.0	THR	421	-0.2
LYS	56	-0.2	LEU	239	1.5	GLN	422	-0.4	LYS	56.0	-0.2	LEU	239	1.5	GLN	422	-0.4
CYS	57	0.0	ASP	240	-2.3	PHE	423	-0.2	CYS	57.0	0.0	ASP	240	-2.3	PHE	423	-0.2
ASP	58	2.3	ARG	241	4.0	VAL	424	-0.3	ASP	58.0	2.4	ARG	241	4.0	VAL	424	-0.3
CYS	59	0.2	GLN	242	0.2	GLU	425	-3.8	CYS	59.0	0.2	GLN	242	0.2	GLU	425	-3.8
THR	60	0.3	HIS	243	0.0	SER	426	-0.2	THR	60.0	0.3	HIS	243	0.0	SER	426	-0.2
ARG	61	-3.3	LYS	244	0.0	PHE	427	-0.4	ARG	61.0	-3.3	LYS	244	0.0	PHE	427	-0.4
THR	62	0.5	LEU	245	2.3	THR	428	-0.2	THR	62.0	0.5	LEU	245	2.3	THR	428	-0.2
GLY	63	0.0	ARG	246	2.4	ARG	429	4.1	GLY	63.0	0.0	ARG	246	2.4	ARG	429	4.1
PHE	64	-0.4	LEU	247	1.5	GLN	430	-0.5	PHE	64.0	-0.4	LEU	247	1.5	GLN	430	-0.5
TYR	65	0.0	PHE	248	0.1	ILE	431	-0.3	TYR	65.0	0.0	PHE	248	0.1	ILE	431	-0.3
GLY	66	0.0	LYS	249	-0.1	ALA	432	0.5	GLY	66.0	0.0	LYS	249	-0.2	ALA	432	0.4
GLU	67	1.5	ASP	250	-1.7	GLY	433	-1.0	GLU	67.0	1.6	ASP	250	-1.7	GLY	433	-1.0

ASN	68	0.1	GLY	251	-0.1	ARG	434	6.9	ASN	68.0	0.1	GLY	251	-0.1	ARG	434	6.9
CYS	69	0.2	LYS	252	-0.2	VAL	435	1.4	CYS	69.0	0.2	LYS	252	-0.2	VAL	435	1.4
THR	70	0.1	LEU	253	2.3	ALA	436	1.1	THR	70.0	0.1	LEU	253	2.3	ALA	436	1.1
THR	71	0.2	LYS	254	-0.3	GLY	437	0.4	THR	71.0	0.2	LYS	254	-0.4	GLY	437	0.4
PRO	72	-0.2	TYR	255	0.1	GLY	438	0.2	PRO	72.0	-0.2	TYR	255	0.1	GLY	438	0.2
GLU	73	4.7	GLN	256	0.1	ARG	439	3.5	GLU	73.0	4.8	GLN	256	0.1	ARG	439	3.5
PHE	74	0.4	VAL	257	-0.1	ASN	440	0.4	PHE	74.0	0.4	VAL	257	-0.1	ASN	440	0.4
LEU	75	-3.1	ILE	258	0.0	VAL	441	0.2	LEU	75.0	-3.7	ILE	258	0.0	VAL	441	0.2
THR	76	0.4	GLY	259	0.0	PRO	442	0.3	THR	76.0	0.4	GLY	259	0.0	PRO	442	0.3
ARG	77	-4.1	GLY	260	-0.1	ILE	443	-0.1	ARG	77.0	-4.1	GLY	260	-0.1	ILE	443	-0.1
ILE	78	0.6	GLU	261	-3.0	ALA	444	-0.1	ILE	78.0	0.6	GLU	261	-3.0	ALA	444	-0.1
LYS	79	0.4	VAL	262	-0.3	VAL	445	-0.2	LYS	79.0	1.0	VAL	262	-0.3	VAL	445	-0.2
LEU	80	-5.0	TYR	263	0.0	GLN	446	-0.2	LEU	80.0	-5.0	TYR	263	0.0	GLN	446	-0.2
LEU	81	-3.8	PRO	264	0.2	ALA	447	-0.2	LEU	81.0	-3.7	PRO	264	0.2	ALA	447	-0.2
LEU	82	-4.8	PRO	265	-0.1	VAL	448	-0.3	LEU	82.0	-4.8	PRO	265	-0.1	VAL	448	-0.3
LYS	83	-1.9	THR	266	0.0	ALA	449	0.2	LYS	83.0	-2.2	THR	266	0.0	ALA	449	0.2
PRO	84	0.8	VAL	267	0.0	LYS	450	-0.7	PRO	84.0	0.8	VAL	267	0.0	LYS	450	-0.8
THR	85	3.5	LYS	268	-0.2	ALA	451	-0.1	THR	85.0	3.5	LYS	268	-0.1	ALA	451	-0.1
PRO	86	0.7	ASP	269	-2.3	SER	452	0.5	PRO	86.0	0.4	ASP	269	-2.3	SER	452	0.5
ASN	87	1.8	THR	270	0.0	ILE	453	0.3	ASN	87.0	1.7	THR	270	0.0	ILE	453	0.3
THR	88	0.8	GLN	271	-0.1	ASP	454	-4.1	THR	88.0	0.6	GLN	271	-0.1	ASP	454	-4.1
VAL	89	-0.1	VAL	272	-0.1	GLN	455	-0.2	VAL	89.0	-0.2	VAL	272	-0.1	GLN	455	-0.2
HIS	90	-2.7	GLU	273	-3.3	SER	456	0.4	HIS	90.0	-2.4	GLU	273	-3.3	SER	456	0.4
TYR	91	-0.3	MET	274	0.0	ARG	457	3.6	TYR	91.0	-0.4	MET	274	0.0	ARG	457	3.7
ILE	92	-1.0	ILE	275	0.0	GLU	458	-3.7	ILE	92.0	-0.8	ILE	275	0.1	GLU	458	-3.6
LEU	93	-28.1	TYR	276	0.0	MET	459	0.2	LEU	93.0	-27.0	TYR	276	0.0	MET	459	0.2
THR	94	-2.2	PRO	277	0.1	LYS	460	-0.3	THR	94.0	-2.0	PRO	277	0.1	LYS	460	-0.5
HIS	95	-0.6	PRO	278	0.0	TYR	461	0.2	HIS	95.0	-0.6	PRO	278	0.0	TYR	461	0.2
PHE	96	-0.1	HIS	279	0.0	GLN	462	-0.2	PHE	96.0	-0.1	HIS	279	0.0	GLN	462	-0.3
LYS	97	1.2	ILE	280	0.2	SER	463	0.0	LYS	97.0	1.9	ILE	280	0.2	SER	463	0.0
GLY	98	0.2	PRO	281	-0.1	LEU	464	2.1	GLY	98.0	0.2	PRO	281	-0.1	LEU	464	2.1
VAL	99	0.2	GLU	282	-2.4	ASN	465	0.5	VAL	99.0	0.2	GLU	282	-2.4	ASN	465	0.5
TRP	100	-1.5	ASN	283	-0.1	GLU	466	1.4	TRP	100.0	-1.5	ASN	283	-0.1	GLU	466	1.5
ASN	101	0.1	LEU	284	1.9	TYR	467	1.1	ASN	101.0	0.1	LEU	284	1.9	TYR	467	1.2
ILE	102	0.2	GLN	285	-0.1	ARG	468	-2.0	ILE	102.0	0.2	GLN	285	-0.1	ARG	468	-2.0
VAL	103	-0.2	PHE	286	-0.1	LYS	469	1.9	VAL	103.0	-0.2	PHE	286	-0.1	LYS	469	2.0
ASN	104	0.5	ALA	287	-0.1	ARG	470	-2.7	ASN	104.0	0.5	ALA	287	-0.1	ARG	470	-3.1
ASN	105	-0.2	VAL	288	-0.1	PHE	471	2.5	ASN	105.0	-0.2	VAL	288	-0.1	PHE	471	2.4
ILE	106	0.3	GLY	289	0.0	SER	472	0.2	ILE	106.0	0.3	GLY	289	0.0	SER	472	0.1
PRO	107	0.7	GLN	290	0.8	LEU	473	-7.4	PRO	107.0	0.7	GLN	290	0.8	LEU	473	-7.5
PHE	108	0.8	GLU	291	-5.1	LYS	474	0.3	PHE	108.0	0.8	GLU	291	-5.1	LYS	474	0.4
LEU	109	-4.5	VAL	292	-0.2	PRO	475	0.1	LEU	109.0	-4.4	VAL	292	-0.2	PRO	475	0.0
ARG	110	-3.5	PHE	293	0.3	TYR	476	0.3	ARG	110.0	-3.6	PHE	293	0.3	TYR	476	0.5
SER	111	1.2	GLY	294	0.0	THR	477	-0.3	SER	111.0	1.2	GLY	294	0.0	THR	477	-0.3

LEU	112	-5.8	LEU	295	4.3	SER	478	-0.1	LEU	112.0	-5.6	LEU	295	4.4	SER	478	-0.1
ILE	113	3.4	VAL	296	-0.1	PHE	479	0.0	ILE	113.0	3.5	VAL	296	-0.1	PHE	479	0.0
MET	114	3.5	PRO	297	-0.3	GLU	480	-0.1	MET	114.0	3.0	PRO	297	-0.3	GLU	480	-0.2
LYS	115	6.2	GLY	298	-0.2	GLU	481	2.1	LYS	115.0	6.3	GLY	298	-0.2	GLU	481	2.0
TYR	116	3.4	LEU	299	6.0	LEU	482	1.0	TYR	116.0	3.4	LEU	299	6.0	LEU	482	1.1
VAL	117	2.8	MET	300	0.1	THR	483	0.2	VAL	117.0	3.3	MET	300	0.1	THR	483	0.2
LEU	118	-11.7	MET	301	0.2	GLY	484	-0.1	LEU	118.0	-12.2	MET	301	0.2	GLY	484	-0.1
THR	119	2.4	TYR	302	0.6	GLU	485	-1.7	THR	119.0	1.3	TYR	302	0.6	GLU	485	-1.8
SER	120	3.3	ALA	303	0.3	LYS	486	-0.4	SER	120.0	2.9	ALA	303	0.3	LYS	486	-0.5
ARG	121	-184.1	THR	304	0.2	GLU	487	-2.1	ARG	121.0	-246.9	THR	304	0.2	GLU	487	-2.1
SER	122	-3.9	ILE	305	0.3	MET	488	-0.2	SER	122.0	-3.9	ILE	305	0.3	MET	488	-0.2
TYR	123	-2.3	TRP	306	0.9	ALA	489	0.0	TYR	123.0	-2.3	TRP	306	0.9	ALA	489	0.0
LEU	124	-14.2	LEU	307	2.8	ALA	490	-0.1	LEU	124.0	-14.5	LEU	307	2.8	ALA	490	-0.1
ILE	125	0.0	ARG	308	3.9	GLU	491	-2.1	ILE	125.0	-0.1	ARG	308	3.8	GLU	491	-1.9
ASP	126	2.2	GLU	309	-3.6	LEU	492	1.9	ASP	126.0	2.4	GLU	309	-3.6	LEU	492	1.9
SER	127	0.2	HIS	310	0.1	LYS	493	-0.4	SER	127.0	0.2	HIS	310	0.1	LYS	493	-0.4
PRO	128	0.1	ASN	311	0.1	ALA	494	0.0	PRO	128.0	0.1	ASN	311	0.1	ALA	494	0.0
PRO	129	0.3	ARG	312	3.1	LEU	495	1.5	PRO	129.0	0.3	ARG	312	3.1	LEU	495	1.6
THR	130	-0.5	VAL	313	0.1	TYR	496	0.1	THR	130.0	-0.3	VAL	313	0.1	TYR	496	0.1
TYR	131	-0.4	CYS	314	0.3	SER	497	0.1	TYR	131.0	-0.5	CYS	314	0.3	SER	497	0.2
ASN	132	0.1	ASP	315	-1.9	ASP	498	-0.2	ASN	132.0	0.1	ASP	315	-1.9	ASP	498	-0.2
VAL	133	0.2	ILE	316	0.1	ILE	499	-0.1	VAL	133.0	0.2	ILE	316	0.2	ILE	499	-0.1
HIS	134	0.2	LEU	317	1.3	ASP	500	0.4	HIS	134.0	0.2	LEU	317	1.2	ASP	500	0.4
TYR	135	0.1	LYS	318	0.0	VAL	501	-0.3	TYR	135.0	0.1	LYS	318	0.1	VAL	501	-0.3
GLY	136	0.2	GLN	319	0.1	MET	502	-0.1	GLY	136.0	0.2	GLN	319	0.1	MET	502	-0.1
TYR	137	-0.1	GLU	320	-0.9	GLU	503	-4.2	TYR	137.0	-0.1	GLU	320	-0.8	GLU	503	-4.3
LYS	138	-0.7	HIP	321	1.0	LEU	504	4.0	LYS	138.0	-0.7	HIP	321	1.1	LEU	504	4.0
SER	139	-0.1	PRO	322	0.0	TYR	505	-0.5	SER	139.0	-0.1	PRO	322	0.0	TYR	505	-0.1
TRP	140	-0.2	GLU	323	-0.9	PRO	506	-0.3	TRP	140.0	-0.2	GLU	323	-0.8	PRO	506	-0.3
GLU	141	-1.6	TRP	324	0.0	ALA	507	-0.6	GLU	141.0	-1.6	TRP	324	-0.1	ALA	507	-0.6
ALA	142	-0.1	GLY	325	-0.1	LEU	508	8.7	ALA	142.0	-0.1	GLY	325	-0.1	LEU	508	8.9
PHE	143	-0.3	ASP	326	-2.0	LEU	509	8.1	PHE	143.0	-0.3	ASP	326	-1.9	LEU	509	8.3
SER	144	-0.2	GLU	327	-1.9	VAL	510	0.2	SER	144.0	-0.2	GLU	327	-1.9	VAL	510	0.2
ASN	145	-0.4	GLN	328	-0.1	GLU	511	-4.2	ASN	145.0	-0.4	GLN	328	-0.1	GLU	511	-4.3
LEU	146	1.4	LEU	329	0.9	LYS	512	-1.7	LEU	146.0	1.4	LEU	329	0.9	LYS	512	-2.1
SER	147	-0.3	PHE	330	-0.1	PRO	513	1.4	SER	147.0	-0.3	PHE	330	-0.1	PRO	513	1.5
TYR	148	-0.3	GLN	331	-0.2	ARG	514	-12.9	TYR	148.0	-0.3	GLN	331	-0.2	ARG	514	-12.9
TYR	149	0.4	THR	332	-0.1	PRO	515	-0.9	TYR	149.0	0.4	THR	332	-0.1	PRO	515	-0.9
THR	150	-0.2	SER	333	-0.2	ASP	516	-2.9	THR	150.0	-0.2	SER	333	-0.2	ASP	516	-2.9
ARG	151	3.9	ARG	334	3.7	ALA	517	-1.0	ARG	151.0	3.8	ARG	334	3.7	ALA	517	-1.0
ALA	152	0.9	LEU	335	1.3	ILE	518	0.8	ALA	152.0	0.9	LEU	335	1.3	ILE	518	0.7
LEU	153	0.0	ILE	336	-0.4	PHE	519	6.4	LEU	153.0	-0.1	ILE	336	-0.4	PHE	519	6.2
PRO	154	-0.3	LEU	337	2.5	GLY	520	0.0	PRO	154.0	-0.3	LEU	337	2.4	GLY	520	0.1
PRO	155	0.3	ILE	338	-0.4	GLU	521	8.7	PRO	155.0	0.3	ILE	338	-0.4	GLU	521	8.6

VAL	156	0.0	GLY	339	-0.4	THR	522	-1.5	VAL	156.0	0.0	GLY	339	-0.4	THR	522	-1.5
ALA	157	-0.1	GLU	340	-5.5	MET	523	-9.1	ALA	157.0	-0.1	GLU	340	-5.4	MET	523	-10.3
ASP	158	-1.4	THR	341	-0.9	VAL	524	2.8	ASP	158.0	-1.4	THR	341	-0.9	VAL	524	3.5
ASP	159	-0.5	ILE	342	-0.7	GLU	525	46.5	ASP	159.0	-0.5	ILE	342	-0.7	GLU	525	48.3
CYS	160	0.0	LYS	343	-3.1	LEU	526	2.0	CYS	160.0	0.0	LYS	343	-3.0	LEU	526	1.5
PRO	161	-0.1	ILE	344	-1.8	GLY	527	-3.2	PRO	161.0	-0.1	ILE	344	-1.6	GLY	527	-3.2
THR	162	-0.1	VAL	345	-2.1	ALA	528	-13.9	THR	162.0	-0.1	VAL	345	-2.0	ALA	528	-15.2
PRO	163	0.0	ILE	346	-1.1	PRO	529	-6.3	PRO	163.0	0.0	ILE	346	-1.1	PRO	529	-6.6
MET	164	0.2	GLU	347	-3.2	PHE	530	-0.2	MET	164.0	0.2	GLU	347	-3.0	PHE	530	-0.2
GLY	165	0.1	ASP	348	-10.2	SER	531	-0.7	GLY	165.0	0.1	ASP	348	-9.8	SER	531	-1.7
VAL	166	-0.1	TYR	349	0.3	LEU	532	-17.5	VAL	166.0	-0.1	TYR	349	0.2	LEU	532	-18.8
LYS	167	-0.7	VAL	350	-7.3	LYS	533	-2.2	LYS	167.0	-0.6	VAL	350	-6.3	LYS	533	-2.2
GLY	168	0.0	GLN	351	-0.7	GLY	534	0.8	GLY	168.0	0.0	GLN	351	-0.9	GLY	534	0.8
ASN	169	0.1	HIS	352	0.2	LEU	535	12.0	ASN	169.0	0.1	HIS	352	0.2	LEU	535	11.4
LYS	170	0.0	LEU	353	37.6	MET	536	-1.0	LYS	170.0	-0.2	LEU	353	37.3	MET	536	-1.1
GLU	171	-1.7	SER	354	9.2	GLY	537	0.0	GLU	171.0	-1.7	SER	354	9.4	GLY	537	0.0
LEU	172	1.5	GLY	355	3.3	ASN	538	1.4	LEU	172.0	1.5	GLY	355	3.3	ASN	538	1.4
PRO	173	-0.1	TYR	356	-105.6	PRO	539	0.3	PRO	173.0	-0.1	TYR	356	-95.1	PRO	539	0.3
ASP	174	-2.9	HIS	357	-2.3	ILE	540	0.6	ASP	174.0	-3.0	HIS	357	-2.3	ILE	540	0.6
SER	175	-0.1	PHE	358	-2.9	CYS	541	0.3	SER	175.0	-0.1	PHE	358	-2.8	CYS	541	0.1
LYS	176	-0.4	LYS	359	2.1	SER	542	0.1	LYS	176.0	-0.4	LYS	359	1.9	SER	542	0.0
GLU	177	-2.7	LEU	360	0.3	PRO	543	-0.3	GLU	177.0	-2.5	LEU	360	-0.7	PRO	543	-0.3
VAL	178	-0.3	LYS	361	-0.6	GLN	544	-0.1	VAL	178.0	-0.3	LYS	361	-0.4	GLN	544	-0.1
LEU	179	3.1	PHE	362	0.6	TYR	545	0.0	LEU	179.0	3.2	PHE	362	0.6	TYR	545	0.0
GLU	180	-3.2	ASP	363	3.9	TRP	546	0.1	GLU	180.0	-3.3	ASP	363	4.2	TRP	546	0.1
LYS	181	-0.6	PRO	364	0.8	LYS	547	-0.3	LYS	181.0	-0.7	PRO	364	0.8	LYS	547	-0.3
VAL	182	-0.5	GLU	365	3.4	PRO	548	-0.2	VAL	182.0	-0.5	GLU	365	3.5	PRO	548	-0.2
LEU	183	3.8	LEU	366	-4.8	SER	549	-0.2	LEU	183.0	3.9	LEU	366	-5.0	SER	549	-0.1
LEU	184	3.2	LEU	367	-5.5	THR	550	-0.1	LEU	184.0	3.3	LEU	367	-5.8	THR	550	-0.2
ARG	185	7.3	PHE	368	0.2	PHE	551	-0.1	ARG	185.0	7.4	PHE	368	0.2	PHE	551	0.0
ARG	186	3.6	ASN	369	0.4	GLY	552	0.0	ARG	186.0	3.7	ASN	369	0.5	GLY	552	0.0
GLU	187	-3.7	GLN	370	-0.1	GLY	553	0.0	GLU	187.0	-3.8	GLN	370	-1.3	GLY	553	-0.1
PHE	188	0.2	GLN	371	1.0	GLU	554	-0.2	PHE	188.0	0.3	GLN	371	1.0	GLU	554	-0.2
ILE	189	-0.6	PHE	372	-0.6	VAL	555	-0.2	ILE	189.0	-0.5	PHE	372	-0.7	VAL	555	-0.2
PRO	190	-0.4	GLN	373	-0.9	GLY	556	-0.1	PRO	190.0	-0.4	GLN	373	-0.8	GLY	556	-0.1
ASP	191	-12.0	TYR	374	-0.4	PHE	557	-0.2	ASP	191.0	-12.2	TYR	374	-0.4	PHE	557	-0.2
PRO	192	0.0	GLN	375	-0.3	LYS	558	-0.2	PRO	192.0	0.0	GLN	375	-0.3	LYS	558	-0.1
GLN	193	0.9	ASN	376	1.1	ILE	559	-0.2	GLN	193.0	0.8	ASN	376	1.1	ILE	559	-0.2
GLY	194	-0.4	ARG	377	2.2	ILE	560	-0.2	GLY	194.0	-0.3	ARG	377	2.1	ILE	560	-0.2
SER	195	0.2	ILE	378	0.7	ASN	561	-0.1	SER	195.0	0.4	ILE	378	0.7	ASN	561	0.0
ASN	196	-1.6	ALA	379	-0.9	THR	562	-0.4	ASN	196.0	-1.6	ALA	379	-0.9	THR	562	-0.4
MET	197	-0.9	SER	380	-1.4	ALA	563	-0.2	MET	197.0	-0.8	SER	380	-1.4	ALA	563	-0.2
MET	198	-0.9	GLU	381	-8.9	SER	564	-0.2	MET	198.0	-0.9	GLU	381	-8.9	SER	564	-0.1
PHE	199	0.6	PHE	382	0.4	ILE	565	0.8	PHE	199.0	0.5	PHE	382	0.4	ILE	565	0.8

ALA	200	-0.5	ASN	383	-2.0	GLN	566	0.2	ALA	200.0	-0.4	ASN	383	-2.2	GLN	566	0.2
PHE	201	-0.9	THR	384	-1.5	SER	567	-0.1	PHE	201.0	-0.9	THR	384	-1.5	SER	567	-0.2
PHE	202	-0.8	LEU	385	17.9	LEU	568	4.3	PHE	202.0	-0.9	LEU	385	18.8	LEU	568	4.2
ALA	203	1.0	TYR	386	2.6	ILE	569	0.5	ALA	203.0	1.0	TYR	386	4.0	ILE	569	0.5
GLN	204	0.2	HIS	387	2.4	CYS	570	0.4	GLN	204.0	0.2	HIS	387	2.5	CYS	570	0.4
HIS	205	-0.3	TRP	388	5.7	ASN	571	0.2	HIS	205.0	-0.3	TRP	388	5.4	ASN	571	0.3
PHE	206	1.2	HIS	389	-2.2	ASN	572	0.3	PHE	206.0	1.2	HIS	389	-2.3	ASN	572	0.3
THR	207	2.9	PRO	390	0.2	VAL	573	0.1	THR	207.0	3.0	PRO	390	0.2	VAL	573	0.1
HIS	208	1.2	LEU	391	13.4	LYS	574	-0.4	HIS	208.0	1.2	LEU	391	13.4	LYS	574	-0.3
GLN	209	1.6	LEU	392	7.0	GLY	575	0.0	GLN	209.0	1.6	LEU	392	7.1	GLY	575	0.0
PHE	210	1.5	PRO	393	0.1	CYS	576	0.0	PHE	210.0	1.5	PRO	393	0.2	CYS	576	-0.1
PHE	211	1.8	ASP	394	-5.3	PRO	577	-0.1	PHE	211.0	1.9	ASP	394	-5.3	PRO	577	-0.1
LYS	212	-0.6	THR	395	-0.2	PHE	578	-0.4	LYS	212.0	-0.6	THR	395	-0.3	PHE	578	-0.3
THR	213	0.3	PHE	396	0.4	THR	579	-0.3	THR	213.0	0.3	PHE	396	0.4	THR	579	-0.3
ASP	214	-4.0	ASN	397	0.1	SER	580	-0.6	ASP	214.0	-4.0	ASN	397	0.1	SER	580	-0.7
HIS	215	0.5	ILE	398	0.2	PHE	581	-0.1	HIS	215.0	0.5	ILE	398	0.3	PHE	581	-0.1
LYS	216	0.3	GLU	399	-3.2	ASN	582	0.1	LYS	216.0	-0.2	GLU	399	-3.1	ASN	582	0.2

Table S6. Electrostatic interaction energies Ees [kJ/mol] for amino acid residues of chain A and B (conformers A) of the COX-1 protein with the MXM.

Chain A									Chain B								
**AA**	**NO**	**kJ**	**AA**	**NO**	**kJ**	**AA**	**NO**	**kJ**	**AA**	**NO**	**kJ**	**AA**	**NO**	**kj**	**AA**	**NO**	**kj**
VAL	33.0	0.1	GLY	217	0.1	ASP	401	2.3	VAL	33	0.0	GLY	217	0.1	ASP	401	2.4
ASN	34.0	0.2	PRO	218	0.1	TYR	402	0.0	ASN	34	0.1	PRO	218	0.1	TYR	402	-0.2
PRO	35.0	0.2	GLY	219	0.1	SER	403	0.0	PRO	35	0.2	GLY	219	0.1	SER	403	0.0
CYS	36.0	0.2	PHE	220	0.0	TYR	404	0.0	CYS	36	0.3	PHE	220	0.0	TYR	404	0.0
CYS	37.0	0.1	THR	221	-0.2	GLU	405	2.0	CYS	37	0.1	THR	221	-0.2	GLU	405	2.1
TYR	38.0	0.2	LYS	222	0.0	GLN	406	0.0	TYR	38	0.2	LYS	222	0.2	GLN	406	0.0
TYR	39.0	0.2	ALA	223	0.2	PHE	407	0.0	TYR	39	0.2	ALA	223	0.1	PHE	407	0.0
PRO	40.0	0.3	LEU	224	-1.2	LEU	408	-0.8	PRO	40	0.3	LEU	224	-1.2	LEU	408	-0.8
CYS	41.0	0.2	GLY	225	0.1	PHE	409	0.0	CYS	41	0.2	GLY	225	0.1	PHE	409	0.0
GLN	42.0	-0.1	HIS	226	0.7	ASN	410	0.0	GLN	42	0.6	HIS	226	0.2	ASN	410	0.0
HIP	43.0	-14.1	GLY	227	-0.3	THR	411	0.0	HIS	43	0.5	GLY	227	-0.2	THR	411	0.0
GLN	44.0	-1.2	VAL	228	0.4	SER	412	0.0	GLN	44	-1.3	VAL	228	0.5	SER	412	0.0
GLY	45.0	-0.2	ASP	229	1.9	MET	413	0.0	GLY	45	-0.2	ASP	229	1.6	MET	413	0.0
ILE	46.0	-0.3	LEU	230	0.0	LEU	414	-0.7	ILE	46	-0.3	LEU	230	0.1	LEU	414	-0.7
CYS	47.0	-0.2	GLY	231	0.0	VAL	415	0.0	CYS	47	-0.2	GLY	231	0.0	VAL	415	0.0
VAL	48.0	-0.1	HIS	232	0.2	ASP	416	2.0	VAL	48	-0.1	HIS	232	0.1	ASP	416	2.0
ARG	49.0	-4.6	ILE	233	0.1	TYR	417	0.0	ARG	49	-4.5	ILE	233	0.1	TYR	417	0.4
PHE	50.0	-0.1	TYR	234	0.0	GLY	418	0.0	PHE	50	-0.1	TYR	234	0.0	GLY	418	0.0
GLY	51.0	0.0	GLY	235	0.0	VAL	419	0.1	GLY	51	0.0	GLY	235	0.0	VAL	419	0.1
LEU	52.0	-1.4	ASP	236	2.6	GLU	420	2.1	LEU	52	-1.3	ASP	236	2.4	GLU	420	2.2

ASP	53.0	4.2	ASN	237	-0.1	ALA	421	0.0	ASP	53	4.1	ASN	237	-0.1	ALA	421	0.0
ARG	54.0	-4.2	LEU	238	-0.9	LEU	422	-0.8	ARG	54	-4.1	LEU	238	-0.9	LEU	422	-0.8
TYR	55.0	-0.1	GLU	239	2.2	VAL	423	0.1	TYR	55	-0.1	GLU	239	2.1	VAL	423	0.1
GLN	56.0	0.3	ARG	240	-2.1	ASP	424	2.7	GLN	56	0.3	ARG	240	-2.0	ASP	424	2.8
CYS	57.0	0.2	GLN	241	-0.1	ALA	425	0.1	CYS	57	0.1	GLN	241	0.0	ALA	425	0.1
ASP	58.0	7.5	TYR	242	0.0	PHE	426	0.1	ASP	58	7.3	TYR	242	0.0	PHE	426	0.2
CYS	59.0	0.3	GLN	243	0.0	SER	427	0.2	CYS	59	0.3	GLN	243	0.0	SER	427	0.2
THR	60.0	0.4	LEU	244	-0.8	ARG	428	-2.8	THR	60	0.4	LEU	244	-0.7	ARG	428	-3.0
ARG	61.0	-8.0	ARG	245	-1.9	GLN	429	0.0	ARG	61	-7.7	ARG	245	-1.8	GLN	429	0.0
THR	62.0	0.5	LEU	246	-0.7	PRO	430	-0.2	THR	62	0.5	LEU	246	-0.7	PRO	430	-0.2
GLY	63.0	-0.1	PHE	247	0.0	ALA	431	0.1	GLY	63	-0.1	PHE	247	0.0	ALA	431	0.1
TYR	64.0	-0.1	LYS	248	0.0	GLY	432	-0.2	TYR	64	-0.3	LYS	248	0.0	GLY	432	-0.2
SER	65.0	-0.1	ASP	249	1.8	ARG	433	-3.8	SER	65	0.1	ASP	249	1.8	ARG	433	-5.1
GLY	66.0	0.1	GLY	250	0.0	ILE	434	1.2	GLY	66	0.0	GLY	250	0.0	ILE	434	1.1
PRO	67.0	0.2	LYS	251	0.0	GLY	435	0.6	PRO	67	0.2	LYS	251	0.0	GLY	435	0.6
ASN	68.0	0.3	LEU	252	-0.7	GLY	436	0.1	ASN	68	0.2	LEU	252	-0.6	GLY	436	0.1
CYS	69.0	0.4	LYS	253	0.0	GLY	437	0.1	CYS	69	0.4	LYS	253	0.0	GLY	437	0.2
THR	70.0	0.0	TYR	254	0.0	ARG	438	-3.1	THR	70	0.1	TYR	254	0.0	ARG	438	-3.3
ILE	71.0	0.3	GLN	255	0.0	ASN	439	0.0	ILE	71	0.3	GLN	255	0.0	ASN	439	0.0
PRO	72.0	-0.3	MET	256	0.0	ILE	440	0.2	PRO	72	-0.3	MET	256	0.0	ILE	440	0.2
GLU	73.0	8.8	LEU	257	-0.7	ASP	441	2.1	GLU	73	8.9	LEU	257	-0.7	ASP	441	2.2
ILE	74.0	0.5	ASN	258	0.0	HIS	442	0.2	ILE	74	0.5	ASN	258	0.0	HIS	442	0.0
TRP	75.0	0.8	GLY	259	0.0	HIP	443	-0.6	TRP	75	0.8	GLY	259	0.0	HIS	443	0.1
THR	76.0	0.5	GLU	260	1.9	ILE	444	0.0	THR	76	0.5	GLU	260	1.9	ILE	444	0.0
TRP	77.0	0.7	VAL	261	0.0	LEU	445	-0.8	TRP	77	0.6	VAL	261	0.0	LEU	445	-0.9
LEU	78.0	-3.3	TYR	262	0.0	HIS	446	0.2	LEU	78	-3.2	TYR	262	0.0	HIS	446	0.1
ARG	79.0	-15.2	PRO	263	0.0	VAL	447	0.1	ARG	79	-15.8	PRO	263	0.0	VAL	447	0.1
THR	80.0	-0.1	PRO	264	0.0	ALA	448	0.2	THR	80	-0.2	PRO	264	0.0	ALA	448	0.2
THR	81.0	0.2	SER	265	0.0	VAL	449	0.1	THR	81	0.3	SER	265	0.0	VAL	449	0.1
LEU	82.0	-3.9	VAL	266	0.0	ASP	450	2.6	LEU	82	-3.9	VAL	266	0.0	ASP	450	2.7
ARG	83.0	-24.1	GLU	267	1.8	VAL	451	0.3	ARG	83	-22.3	GLU	267	1.8	VAL	451	0.3
PRO	84.0	0.7	GLU	268	1.8	ILE	452	0.2	PRO	84	0.9	GLU	268	1.8	ILE	452	0.2
SER	85.0	1.9	ALA	269	0.0	LYS	453	0.3	SER	85	2.0	ALA	269	0.0	LYS	453	0.3
PRO	86.0	0.1	PRO	270	0.0	GLU	454	3.5	PRO	86	0.0	PRO	270	0.0	GLU	454	3.3
SER	87.0	0.6	VAL	271	0.0	SER	455	0.3	SER	87	0.7	VAL	271	0.0	SER	455	0.3
PHE	88.0	1.1	LEU	272	-0.9	ARG	456	-3.6	PHE	88	1.3	LEU	272	-0.8	ARG	456	-3.6
ILE	89.0	-0.2	MET	273	0.0	VAL	457	0.2	ILE	89	-0.3	MET	273	0.0	VAL	457	0.2
HIS	90.0	-17.6	HIS	274	0.2	LEU	458	-1.6	HIS	90	1.6	HIS	274	0.0	LEU	458	-1.6
PHE	91.0	0.4	TYR	275	0.0	ARG	459	-4.2	PHE	91	0.4	TYR	275	0.0	ARG	459	-4.3
LEU	92.0	-7.8	PRO	276	0.0	LEU	460	-2.2	LEU	92	-7.8	PRO	276	0.0	LEU	460	-2.2
LEU	93.0	-16.5	ARG	277	-1.9	GLN	461	0.0	LEU	93	-17.2	ARG	277	-1.9	GLN	461	0.1
THR	94.0	-2.1	GLY	278	0.0	PRO	462	0.4	THR	94	-2.4	GLY	278	0.0	PRO	462	0.5
HIP	95.0	-16.7	ILE	279	0.0	PHE	463	0.9	HIS	95	-0.5	ILE	279	0.0	PHE	463	1.0
GLY	96.0	0.2	PRO	280	0.0	ASN	464	0.9	GLY	96	0.3	PRO	280	0.0	ASN	464	0.9
ARG	97.0	-10.2	PRO	281	0.0	GLU	465	9.3	ARG	97	-10.2	PRO	281	0.0	GLU	465	9.5

TRP	98.0	0.5	GLN	282	0.0	TYR	466	1.1	TRP	98	0.5	GLN	282	0.0	TYR	466	1.1
LEU	99.0	-4.3	SER	283	0.0	ARG	467	-10.8	LEU	99	-4.3	SER	283	0.0	ARG	467	-11.4
TRP	100.0	-1.3	GLN	284	0.0	LYS	468	2.9	TRP	100	-1.3	GLN	284	0.0	LYS	468	3.1
ASP	101.0	9.0	MET	285	0.0	ARG	469	-14.4	ASP	101	9.1	MET	285	0.0	ARG	469	-14.5
PHE	102.0	0.1	ALA	286	0.0	PHE	470	1.0	PHE	102	0.1	ALA	286	0.0	PHE	470	1.5
VAL	103.0	-0.5	VAL	287	0.0	GLY	471	0.5	VAL	103	-0.6	VAL	287	0.0	GLY	471	0.2
ASN	104.0	-0.1	GLY	288	0.1	MET	472	-0.6	ASN	104	-0.2	GLY	288	0.1	MET	472	-0.6
ALA	105.0	-0.2	GLN	289	0.0	LYS	473	0.2	ALA	105	-0.2	GLN	289	0.0	LYS	473	0.3
THR	106.0	0.2	GLU	290	2.2	PRO	474	-0.1	THR	106	0.2	GLU	290	2.2	PRO	474	0.0
PHE	107.0	0.9	VAL	291	0.0	TYR	475	-0.1	PHE	107	0.8	VAL	291	0.0	TYR	475	-0.2
ILE	108.0	1.4	PHE	292	0.0	THR	476	-0.5	ILE	108	1.2	PHE	292	0.0	THR	476	-0.4
ARG	109.0	-7.2	GLY	293	0.0	SER	477	0.1	ARG	109	-7.3	GLY	293	0.0	SER	477	0.2
ASP	110.0	9.9	LEU	294	-0.8	PHE	478	0.1	ASP	110	9.8	LEU	294	-0.8	PHE	478	0.2
THR	111.0	1.7	LEU	295	-0.7	GLN	479	0.4	THR	111	1.6	LEU	295	-0.7	GLN	479	0.4
LEU	112.0	-5.2	PRO	296	0.0	GLU	480	8.2	LEU	112	-5.8	PRO	296	0.0	GLU	480	8.6
MET	113.0	9.8	GLY	297	0.0	LEU	481	-2.3	MET	113	9.5	GLY	297	0.0	LEU	481	-2.5
ARG	114.0	-9.8	LEU	298	-0.7	THR	482	0.2	ARG	114	-8.7	LEU	298	-0.7	THR	482	0.2
LEU	115.0	-7.2	MET	299	0.0	GLY	483	-0.2	LEU	115	-6.4	MET	299	0.0	GLY	483	-0.2
VAL	116.0	-1.1	LEU	300	-0.8	GLU	484	4.6	VAL	116	-0.3	LEU	300	-0.8	GLU	484	4.8
LEU	117.0	-3.7	TYR	301	0.0	LYS	485	-0.1	LEU	117	-1.2	TYR	301	0.0	LYS	485	-0.1
THR	118.0	1.7	ALA	302	0.0	GLU	486	3.7	THR	118	0.7	ALA	302	0.0	GLU	486	3.8
VAL	119.0	1.4	THR	303	0.0	MET	487	-0.1	VAL	119	0.4	THR	303	0.0	MET	487	-0.1
ARG	120.0	-119.8	ILE	304	-0.1	ALA	488	-0.1	ARG	120	-112.9	ILE	304	-0.1	ALA	488	-0.1
SER	121.0	-5.5	TRP	305	0.1	ALA	489	0.0	SER	121	-5.0	TRP	305	0.1	ALA	489	-0.1
ASN	122.0	-3.0	LEU	306	-0.7	GLU	490	3.5	ASN	122	-1.4	LEU	306	-0.7	GLU	490	3.6
LEU	123.0	-15.5	ARG	307	-1.8	LEU	491	-1.6	LEU	123	-15.6	ARG	307	-1.9	LEU	491	-1.7
ILE	124.0	-2.5	GLU	308	1.4	GLU	492	4.2	ILE	124	-2.1	GLU	308	1.4	GLU	492	4.7
PRO	125.0	-0.2	HIS	309	0.1	GLU	493	3.5	PRO	125	-0.3	HIS	309	0.0	GLU	493	3.7
SER	126.0	-0.6	ASN	310	0.0	LEU	494	-1.4	SER	126	-0.5	ASN	310	0.0	LEU	494	-1.4
PRO	127.0	0.5	ARG	311	-1.7	TYR	495	0.0	PRO	127	0.5	ARG	311	-1.6	TYR	495	0.0
PRO	128.0	-0.2	VAL	312	0.1	GLY	496	0.1	PRO	128	-0.1	VAL	312	0.1	GLY	496	0.1
THR	129.0	-0.6	CYS	313	-0.1	ASP	497	5.5	THR	129	-0.4	CYS	313	-0.1	ASP	497	5.6
TYR	130.0	-0.3	ASP	314	1.9	ILE	498	0.0	TYR	130	-0.3	ASP	314	1.8	ILE	498	0.0
ASN	131.0	0.0	LEU	315	-0.7	ASP	499	6.7	ASN	131	-0.1	LEU	315	-0.7	ASP	499	6.6
ILE	132.0	0.0	LEU	316	-0.6	ALA	500	-0.1	ILE	132	0.0	LEU	316	-0.6	ALA	500	-0.1
ALA	133.0	0.0	LYS	317	0.0	LEU	501	-2.0	ALA	133	0.0	LYS	317	0.0	LEU	501	-2.0
HIS	134.0	-3.5	ALA	318	0.0	GLU	502	4.2	HIS	134	0.3	ALA	318	0.0	GLU	502	4.4
ASP	135.0	6.0	GLU	319	2.0	PHE	503	-0.6	ASP	135	5.9	GLU	319	2.0	PHE	503	-0.6
TYR	136.0	0.0	HIS	320	0.2	TYR	504	0.1	TYR	136	0.0	HIS	320	0.0	TYR	504	0.1
ILE	137.0	-0.3	PRO	321	0.0	PRO	505	0.0	ILE	137	-0.4	PRO	321	0.0	PRO	505	0.0
SER	138.0	0.0	THR	322	0.0	GLY	506	-0.1	SER	138	0.1	THR	322	0.0	GLY	506	-0.1
TRP	139.0	0.1	TRP	323	0.0	LEU	507	-0.7	TRP	139	0.1	TRP	323	0.0	LEU	507	-0.8
GLU	140.0	4.2	GLY	324	0.0	LEU	508	0.5	GLU	140	4.1	GLY	324	0.0	LEU	508	0.5
SER	141.0	0.1	ASP	325	1.3	LEU	509	-1.1	SER	141	0.1	ASP	325	1.8	LEU	509	-1.1
PHE	142.0	-0.1	GLU	326	2.0	GLU	510	7.6	PHE	142	-0.1	GLU	326	1.8	GLU	510	8.1

SER	143.0	-0.2	GLN	327	0.0	LYS	511	-0.6	SER	143	-0.1	GLN	327	0.0	LYS	511	-0.5
ASN	144.0	0.1	LEU	328	-0.8	CYS	512	-0.2	ASN	144	0.0	LEU	328	-0.7	CYS	512	-0.3
VAL	145.0	0.0	PHE	329	-0.1	HIP	513	-7.2	VAL	145	0.0	PHE	329	0.0	HIS	513	-1.1
SER	146.0	0.1	GLN	330	-0.1	PRO	514	0.1	SER	146	0.1	GLN	330	-0.1	PRO	514	0.2
TYR	147.0	0.1	THR	331	-0.1	ASN	515	0.6	TYR	147	0.1	THR	331	-0.1	ASN	515	0.6
TYR	148.0	0.2	ALA	332	-0.1	SER	516	-1.3	TYR	148	0.2	ALA	332	-0.1	SER	516	-1.4
THR	149.0	0.2	ARG	333	-1.8	ILE	517	-1.1	THR	149	0.2	ARG	333	-1.7	ILE	517	-1.1
ARG	150.0	-6.2	LEU	334	-0.8	PHE	518	0.7	ARG	150	-6.3	LEU	334	-0.8	PHE	518	0.9
ILE	151.0	-0.6	ILE	335	-0.3	GLY	519	0.6	ILE	151	-0.6	ILE	335	-0.3	GLY	519	1.0
LEU	152.0	-4.4	LEU	336	-0.7	GLU	520	18.7	LEU	152	-4.4	LEU	336	-0.7	GLU	520	20.1
PRO	153.0	-0.1	ILE	337	-0.9	SER	521	0.2	PRO	153	-0.1	ILE	337	-0.9	SER	521	0.5
SER	154.0	0.0	GLY	338	-0.7	MET	522	-8.8	SER	154	-0.1	GLY	338	-0.6	MET	522	-4.7
VAL	155.0	-0.2	GLU	339	-0.3	ILE	523	-10.7	VAL	155	0.2	GLU	339	-0.3	ILE	523	9.6
PRO	156.0	-0.2	THR	340	-1.0	GLU	524	48.3	PRO	156	-0.1	THR	340	-0.9	GLU	524	51.9
ARG	157.0	-4.1	ILE	341	-2.2	MET	525	1.3	ARG	157	-4.1	ILE	341	-1.9	MET	525	1.3
ASP	158.0	4.5	LYS	342	-2.7	GLY	526	8.6	ASP	158	4.4	LYS	342	-2.5	GLY	526	8.2
CYS	159.0	0.1	ILE	343	-0.2	ALA	527	-35.9	CYS	159	0.1	ILE	343	-0.1	ALA	527	-38.0
PRO	160.0	-0.1	VAL	344	0.7	PRO	528	-5.0	PRO	160	-0.1	VAL	344	0.9	PRO	528	-5.5
THR	161.0	0.0	ILE	345	-0.1	PHE	529	-2.3	THR	161	0.1	ILE	345	4.8	PHE	529	-2.4
PRO	162.0	0.2	GLU	346	1.6	SER	530	-29.4	PRO	162	0.2	GLU	346	1.8	SER	530	-24.4
MET	163.0	0.3	GLU	347	4.1	LEU	531	-49.0	MET	163	0.3	GLU	347	4.5	LEU	531	-71.8
GLY	164.0	-0.1	TYR	348	1.6	LYS	532	-4.7	GLY	164	-0.1	TYR	348	2.7	LYS	532	-3.2
THR	165.0	-0.1	VAL	349	-6.9	GLY	533	-2.6	THR	165	-0.2	VAL	349	-8.0	GLY	533	-2.6
LYS	166.0	-0.3	GLN	350	1.0	LEU	534	13.3	LYS	166	-0.5	GLN	350	1.4	LEU	534	13.6
GLY	167.0	-0.1	GLN	351	1.5	LEU	535	8.8	GLY	167	-0.1	GLN	351	1.6	LEU	535	9.5
LYS	168.0	0.2	LEU	352	-9.4	GLY	536	0.0	LYS	168	0.2	LEU	352	-5.7	GLY	536	0.1
LYS	169.0	-0.1	SER	353	-3.5	ASN	537	0.7	LYS	169	-0.1	SER	353	-3.0	ASN	537	0.7
GLN	170.0	0.1	GLY	354	0.2	PRO	538	-0.2	GLN	170	0.0	GLY	354	0.2	PRO	538	-0.2
LEU	171.0	-1.7	TYR	355	-9.3	ILE	539	0.2	LEU	171	-1.7	TYR	355	-10.6	ILE	539	0.1
PRO	172.0	-0.1	PHE	356	-0.1	CYS	540	-0.4	PRO	172	-0.1	PHE	356	-0.1	CYS	540	0.7
ASP	173.0	3.0	LEU	357	-11.7	SER	541	0.0	ASP	173	3.0	LEU	357	-11.1	SER	541	0.0
ALA	174.0	-0.1	GLN	358	0.8	PRO	542	-0.3	ALA	174	0.0	GLN	358	1.0	PRO	542	-0.2
GLU	175.0	2.5	LEU	359	-12.6	GLU	543	3.1	GLU	175	2.6	LEU	359	-12.3	GLU	543	2.9
PHE	176.0	0.0	LYS	360	-7.2	TYR	544	-0.1	PHE	176	0.0	LYS	360	-6.5	TYR	544	0.0
LEU	177.0	-1.4	PHE	361	2.4	TRP	545	0.2	LEU	177	-1.4	PHE	361	2.2	TRP	545	0.2
SER	178.0	0.0	ASP	362	1.9	LYS	546	-0.5	SER	178	0.0	ASP	362	1.3	LYS	546	-0.4
ARG	179.0	-2.4	PRO	363	1.4	ALA	547	-0.1	ARG	179	-2.5	PRO	363	1.4	ALA	547	-0.1
ARG	180.0	-3.0	GLU	364	4.0	SER	548	-0.1	ARG	180	-3.1	GLU	364	3.5	SER	548	-0.1
PHE	181.0	-0.1	LEU	365	-0.7	THR	549	0.0	PHE	181	-0.1	LEU	365	-0.5	THR	549	0.0
LEU	182.0	-0.9	LEU	366	-2.8	PHE	550	0.1	LEU	182	-1.0	LEU	366	-1.7	PHE	550	0.1
LEU	183.0	-0.9	PHE	367	0.6	GLY	551	0.0	LEU	183	-0.9	PHE	367	0.6	GLY	551	0.0
ARG	184.0	-2.0	GLY	368	0.3	GLY	552	0.0	ARG	184	-2.2	GLY	368	0.4	GLY	552	0.0
ARG	185.0	-2.8	ALA	369	-0.4	GLU	553	2.2	ARG	185	-3.1	ALA	369	-0.4	GLU	553	2.0
LYS	186.0	0.0	GLN	370	1.2	VAL	554	-0.1	LYS	186	0.1	GLN	370	1.0	VAL	554	-0.1
PHE	187.0	0.1	PHE	371	-1.0	GLY	555	-0.2	PHE	187	0.2	PHE	371	-0.8	GLY	555	-0.1

ILE	188.0	-0.1	GLN	372	-0.9	PHE	556	-0.2	ILE	188	-0.1	GLN	372	-0.8	PHE	556	-0.2
PRO	189.0	0.1	TYR	373	-0.1	ASN	557	-0.1	PRO	189	0.1	TYR	373	0.0	ASN	557	-0.2
ASP	190.0	4.7	ARG	374	-5.6	LEU	558	-0.8	ASP	190	5.3	ARG	374	-5.3	LEU	558	-0.7
PRO	191.0	0.3	ASN	375	0.9	VAL	559	-0.3	PRO	191	0.2	ASN	375	0.8	VAL	559	-0.2
GLN	192.0	0.0	ARG	376	-5.9	LYS	560	-0.8	GLN	192	-0.3	ARG	376	-5.5	LYS	560	-0.7
GLY	193.0	-0.2	ILE	377	0.3	THR	561	0.0	GLY	193	-0.1	ILE	377	0.3	THR	561	0.0
THR	194.0	0.0	ALA	378	-0.8	ALA	562	0.2	THR	194	-0.1	ALA	378	-0.8	ALA	562	0.1
ASN	195.0	-0.3	MET	379	-0.3	THR	563	0.1	ASN	195	-0.4	MET	379	-0.2	THR	563	-0.1
LEU	196.0	-0.9	GLU	380	7.6	LEU	564	-0.7	LEU	196	-1.0	GLU	380	7.8	LEU	564	-1.0
MET	197.0	-0.2	PHE	381	-2.3	LYS	565	-0.3	MET	197	-0.2	PHE	381	-2.1	LYS	565	-0.3
PHE	198.0	-0.3	ASN	382	-1.1	LYS	566	0.0	PHE	198	-0.4	ASN	382	-1.1	LYS	566	0.1
ALA	199.0	-0.1	GLN	383	-1.3	LEU	567	-0.4	ALA	199	-0.1	GLN	383	-1.2	LEU	567	-0.4
PHE	200.0	-0.1	LEU	384	-1.6	VAL	568	0.0	PHE	200	-0.1	LEU	384	-2.0	VAL	568	0.0
PHE	201.0	0.0	TYR	385	0.2	CYS	569	0.1	PHE	201	0.0	TYR	385	-0.1	CYS	569	0.1
ALA	202.0	0.0	HIS	386	-0.6	LEU	570	-0.8	ALA	202	0.0	HIS	386	-0.6	LEU	570	-0.8
GLN	203.0	0.1	TRP	387	-13.3	ASN	571	0.0	GLN	203	0.1	TRP	387	-19.2	ASN	571	0.0
HIS	204.0	-1.0	HIS	388	0.3	THR	572	0.0	HIS	204	0.1	HIS	388	-0.1	THR	572	0.0
PHE	205.0	0.3	PRO	389	0.3	LYS	573	0.1	PHE	205	0.2	PRO	389	0.4	LYS	573	0.1
THR	206.0	0.1	LEU	390	0.9	THR	574	0.0	THR	206	0.1	LEU	390	0.8	THR	574	0.0
HIS	207.0	-0.1	MET	391	0.2	CYS	575	0.1	HIS	207	0.1	MET	391	0.1	CYS	575	0.1
GLN	208.0	0.3	PRO	392	0.0	PRO	576	0.1	GLN	208	0.2	PRO	392	-0.1	PRO	576	0.1
PHE	209.0	0.2	ASP	393	2.4	TYR	577	0.0	PHE	209	0.3	ASP	393	2.4	TYR	577	0.0
PHE	210.0	-0.2	SER	394	0.0	VAL	578	0.2	PHE	210	-0.2	SER	394	0.0	VAL	578	0.2
LYS	211.0	0.3	PHE	395	0.0	SER	579	0.0	LYS	211	0.2	PHE	395	0.0	SER	579	0.4
THR	212.0	-0.1	ARG	396	-2.4	PHE	580	0.2	THR	212	-0.1	ARG	396	-2.5	PHE	580	0.2
SER	213.0	-0.1	VAL	397	0.0	HIP	581	-1.8	SER	213	0.0	VAL	397	0.0	HIS	581	0.1
GLY	214.0	0.0	GLY	398	0.0	VAL	582	0.2	GLY	214	0.0	GLY	398	0.0	VAL	582	0.2
LYS	215.0	0.1	PRO	399	0.0	PRO	583	0.1	LYS	215	0.1	PRO	399	0.0	PRO	583	0.2
MET	216	0.1	GLN	400	0.0				MET	216	0.1	GLN	400	0.0			

Table S7. Electrostatic interaction energies Ees [kJ/mol] for amino acid residues of chain A and B (conformers A) of the COX-2 protein with the MXM.

			Chain A - konfA									ChainB - konf A					
**AA**	**NO**	**kJ**	**AA**	**NO**	**kJ**	**AA**	**NO**	**kJ**	**AA**	**NO**	**kJ**	**AA**	**NO**	**kJ**	**AA**	**NO**	**kJ**
ASN	34	0.2	GLY	217	0.3	GLU	401	10.5	ASN	34.0	0.2	GLY	217	0.3	GLU	401	9.2
PRO	35	0.3	PRO	218	0.6	TYR	402	0.1	PRO	35.0	0.3	PRO	218	0.5	TYR	402	0.0
CYS	36	0.5	GLY	219	0.2	SER	403	0.1	CYS	36.0	0.5	GLY	219	0.2	SER	403	0.0
CYS	37	0.4	PHE	220	0.5	PHE	404	-0.2	CYS	37.0	0.3	PHE	220	0.4	PHE	404	-0.1
SER	38	0.6	THR	221	-0.6	LYS	405	-9.6	SER	38.0	0.6	THR	221	-0.6	LYS	405	-8.3
ASN	39	0.2	ARG	222	-13.1	GLN	406	0.1	ASN	39.0	0.3	ARG	222	-11.4	GLN	406	0.1
PRO	40	0.4	GLY	223	0.2	PHE	407	-0.1	PRO	40.0	0.4	GLY	223	0.3	PHE	407	0.0
CYS	41	0.4	LEU	224	0.4	LEU	408	-0.3	CYS	41.0	0.4	LEU	224	0.5	LEU	408	-0.2
GLN	42	0.8	GLY	225	0.6	TYR	409	-0.2	GLN	42.0	0.9	GLY	225	0.6	TYR	409	-0.2
ASN	43	0.9	HIS	226	1.5	ASN	410	0.0	ASN	43.0	0.9	HIS	226	1.6	ASN	410	0.0

ARG	44	-27.4	GLY	227	-0.5	ASN	411	0.1	ARG	44.0	-27.6	GLY	227	-0.5	ASN	411	0.0
GLY	45	0.0	VAL	228	-0.6	SER	412	0.0	GLY	45.0	-0.1	VAL	228	-0.5	SER	412	0.0
GLU	46	17.3	ASP	229	14.4	ILE	413	0.0	GLU	46.0	17.0	ASP	229	13.0	ILE	413	0.0
CYS	47	-0.4	LEU	230	-0.3	LEU	414	-0.2	CYS	47.0	-0.4	LEU	230	-0.3	LEU	414	-0.2
MET	48	-0.1	ASN	231	-0.5	LEU	415	-0.1	MET	48.0	-0.1	ASN	231	-0.6	LEU	415	0.0
SER	49	-0.1	HIS	232	-0.7	GLU	416	8.8	SER	49.0	-0.1	HIS	232	-0.5	GLU	416	7.8
THR	50	-0.3	ILE	233	-0.4	HIS	417	0.2	THR	50.0	-0.3	ILE	233	-0.3	HIS	417	0.2
GLY	51	0.1	TYR	234	-0.1	GLY	418	0.2	GLY	51.0	0.0	TYR	234	-0.1	GLY	418	0.2
PHE	52	0.3	GLY	235	0.0	LEU	419	0.2	PHE	52.0	0.3	GLY	235	0.1	LEU	419	0.2
ASP	53	10.1	GLU	236	13.5	THR	420	0.2	ASP	53.0	9.7	GLU	236	12.2	THR	420	0.1
GLN	54	0.2	THR	237	-0.1	GLN	421	0.1	GLN	54.0	0.2	THR	237	-0.1	GLN	421	0.1
TYR	55	-0.2	LEU	238	-0.1	PHE	422	0.4	TYR	55.0	-0.2	LEU	238	-0.1	PHE	422	0.3
LYS	56	-12.1	ASP	239	9.4	VAL	423	0.2	LYS	56.0	-11.8	ASP	239	8.5	VAL	423	0.2
CYS	57	0.3	ARG	240	-10.9	GLU	424	10.9	CYS	57.0	0.3	ARG	240	-9.6	GLU	424	9.8
ASP	58	15.6	GLN	241	-0.4	SER	425	0.3	ASP	58.0	15.4	GLN	241	-0.4	SER	425	0.2
CYS	59	0.4	HIS	242	-0.2	PHE	426	0.6	CYS	59.0	0.4	HIS	242	-0.2	PHE	426	0.5
THR	60	0.6	LYS	243	-9.0	THR	427	0.4	THR	60.0	0.6	LYS	243	-8.1	THR	427	0.3
ARG	61	-16.2	LEU	244	-0.2	ARG	428	-11.3	ARG	61.0	-16.2	LEU	244	-0.1	ARG	428	-10.2
THR	62	0.5	ARG	245	-9.4	GLN	429	0.5	THR	62.0	0.5	ARG	245	-8.6	GLN	429	0.4
GLY	63	-0.2	LEU	246	-0.1	ILE	430	0.0	GLY	63.0	-0.2	LEU	246	-0.1	ILE	430	-0.2
PHE	64	-0.6	PHE	247	-0.1	ALA	431	-0.1	PHE	64.0	-0.6	PHE	247	-0.1	ALA	431	0.0
TYR	65	-0.1	LYS	248	-7.5	GLY	432	0.7	TYR	65.0	-0.1	LYS	248	-7.0	GLY	432	0.4
GLY	66	0.1	ASP	249	8.3	ARG	433	-19.8	GLY	66.0	0.1	ASP	249	7.7	ARG	433	-17.7
GLU	67	12.7	GLY	250	0.2	VAL	434	-0.4	GLU	67.0	12.4	GLY	250	0.1	VAL	434	0.2
ASN	68	0.5	LYS	251	-8.5	ALA	435	-0.7	ASN	68.0	0.5	LYS	251	-7.8	ALA	435	-0.5
CYS	69	0.7	LEU	252	-0.2	GLY	436	-0.7	CYS	69.0	0.7	LEU	252	-0.2	GLY	436	-0.6
THR	70	0.1	LYS	253	-8.3	GLY	437	-0.4	THR	70.0	0.1	LYS	253	-7.6	GLY	437	-0.3
THR	71	0.4	TYR	254	-0.1	ARG	438	-13.6	THR	71.0	0.4	TYR	254	-0.1	ARG	438	-12.0
PRO	72	-0.5	GLN	255	-0.1	ASN	439	-0.6	PRO	72.0	-0.5	GLN	255	-0.1	ASN	439	-0.5
GLU	73	16.2	VAL	256	0.0	VAL	440	-0.5	GLU	73.0	16.2	VAL	256	-0.1	VAL	440	-0.3
PHE	74	0.8	ILE	257	0.0	PRO	441	-0.2	PHE	74.0	0.8	ILE	257	0.0	PRO	441	-0.1
LEU	75	0.6	GLY	258	0.0	ILE	442	0.0	LEU	75.0	0.7	GLY	258	0.0	ILE	442	0.0
THR	76	0.4	GLY	259	0.1	ALA	443	0.0	THR	76.0	0.4	GLY	259	0.1	ALA	443	0.0
ARG	77	-14.6	GLU	260	9.0	VAL	444	0.1	ARG	77.0	-14.6	GLU	260	8.2	VAL	444	0.1
ILE	78	1.0	VAL	261	0.1	GLN	445	0.4	ILE	78.0	1.0	VAL	261	0.1	GLN	445	0.3
LYS	79	-22.4	TYR	262	0.1	ALA	446	0.4	LYS	79.0	-22.8	TYR	262	0.1	ALA	446	0.3
LEU	80	0.1	PRO	263	-0.3	VAL	447	0.3	LEU	80.0	0.1	PRO	263	-0.2	VAL	447	0.3
LEU	81	0.6	PRO	264	0.1	ALA	448	0.1	LEU	81.0	0.6	PRO	264	0.0	ALA	448	0.2
LEU	82	1.3	THR	265	0.1	LYS	449	-11.8	LEU	82.0	1.4	THR	265	0.1	LYS	449	-10.2
LYS	83	-35.0	VAL	266	-0.2	ALA	450	0.4	LYS	83.0	-36.0	VAL	266	-0.1	ALA	450	0.4
PRO	84	1.1	LYS	267	-7.6	SER	451	0.4	PRO	84.0	1.2	LYS	267	-6.8	SER	451	0.4
THR	85	3.1	ASP	268	8.0	ILE	452	0.0	THR	85.0	3.3	ASP	268	7.3	ILE	452	0.1
PRO	86	-0.4	THR	269	0.0	ASP	453	13.7	PRO	86.0	-0.4	THR	269	0.1	ASP	453	11.9
ASN	87	1.9	GLN	270	0.2	GLN	454	0.8	ASN	87.0	2.1	GLN	270	0.2	GLN	454	0.7
THR	88	0.4	VAL	271	0.0	SER	455	0.2	THR	88.0	0.4	VAL	271	0.0	SER	455	0.3

VAL	89	-0.4	GLU	272	10.0	ARG	456	-14.3	VAL	89.0	-0.5	GLU	272	8.8	ARG	456	-12.6
HIS	90	1.2	MET	273	0.2	GLU	457	13.7	HIS	90.0	1.4	MET	273	0.2	GLU	457	12.2
TYR	91	-0.3	ILE	274	-0.1	MET	458	0.4	TYR	91.0	-0.3	ILE	274	-0.1	MET	458	0.5
ILE	92	0.0	TYR	275	0.0	LYS	459	-13.2	ILE	92.0	0.0	TYR	275	0.0	LYS	459	-12.1
LEU	93	-3.6	PRO	276	-0.2	TYR	460	0.0	LEU	93.0	-3.8	PRO	276	-0.1	TYR	460	0.0
THR	94	-3.3	PRO	277	-0.1	GLN	461	0.3	THR	94.0	-3.5	PRO	277	-0.1	GLN	461	0.2
HIS	95	-0.5	HIS	278	-0.1	SER	462	1.3	HIS	95.0	-0.6	HIS	278	-0.1	SER	462	1.3
PHE	96	0.3	ILE	279	-0.2	LEU	463	0.9	PHE	96.0	0.3	ILE	279	-0.2	LEU	463	1.0
LYS	97	-19.7	PRO	280	0.1	ASN	464	1.6	LYS	97.0	-19.5	PRO	280	0.1	ASN	464	1.6
GLY	98	0.0	GLU	281	8.0	GLU	465	21.0	GLY	98.0	0.0	GLU	281	7.1	GLU	465	20.3
VAL	99	0.1	ASN	282	0.3	TYR	466	1.2	VAL	99.0	0.1	ASN	282	0.2	TYR	466	1.4
TRP	100	-1.6	LEU	283	0.2	ARG	467	-27.2	TRP	100.0	-1.6	LEU	283	0.2	ARG	467	-26.6
ASN	101	-0.1	GLN	284	0.1	LYS	468	-18.5	ASN	101.0	-0.1	GLN	284	0.1	LYS	468	-17.9
ILE	102	0.1	PHE	285	0.2	ARG	469	-30.2	ILE	102.0	0.1	PHE	285	0.2	ARG	469	-29.9
VAL	103	-0.7	ALA	286	0.1	PHE	470	0.6	VAL	103.0	-0.7	ALA	286	0.1	PHE	470	0.9
ASN	104	-0.4	VAL	287	0.0	SER	471	0.2	ASN	104.0	-0.3	VAL	287	0.0	SER	471	0.3
ASN	105	-0.7	GLY	288	0.1	LEU	472	-2.4	ASN	105.0	-0.8	GLY	288	0.0	LEU	472	-2.6
ILE	105	0.3	GLN	289	-0.8	LYS	473	-23.1	ILE	105.0	0.4	GLN	289	-0.6	LYS	473	-23.2
PRO	106	0.8	GLU	290	12.0	PRO	474	-0.3	PRO	106.0	0.8	GLU	290	10.4	PRO	474	-0.3
PHE	107	1.1	VAL	291	-0.1	TYR	475	-0.9	PHE	107.0	1.2	VAL	291	-0.1	TYR	475	-0.8
LEU	108	1.2	PHE	292	-0.3	THR	476	-0.4	LEU	108.0	1.3	PHE	292	-0.2	THR	476	-0.5
ARG	109	-18.5	GLY	293	0.0	SER	477	0.5	ARG	109.0	-19.0	GLY	293	0.0	SER	477	0.5
SER	110	1.3	LEU	294	-0.2	PHE	478	0.5	SER	110.0	1.3	LEU	294	-0.2	PHE	478	0.5
LEU	111	2.2	VAL	295	-0.2	GLU	479	14.8	LEU	111.0	2.3	VAL	295	-0.1	GLU	479	14.0
ILE	112	3.4	PRO	296	0.2	GLU	480	20.2	ILE	112.0	3.5	PRO	296	0.2	GLU	480	19.5
MET	113	6.9	GLY	297	0.2	LEU	481	0.4	MET	113.0	7.2	GLY	297	0.1	LEU	481	0.4
LYS	114	-18.0	LEU	298	-0.3	THR	482	0.4	LYS	114.0	-18.2	LEU	298	-0.1	THR	482	0.4
TYR	115	3.8	MET	299	0.0	GLY	483	-0.4	TYR	115.0	3.9	MET	299	0.1	GLY	483	-0.4
VAL	116	7.6	MET	300	0.0	GLU	484	14.7	VAL	116.0	7.8	MET	300	0.1	GLU	484	13.6
LEU	117	6.5	TYR	301	-0.4	LYS	485	-12.8	LEU	117.0	7.3	TYR	301	-0.2	LYS	485	-12.1
THR	118	3.7	ALA	302	-0.3	GLU	486	12.4	THR	118.0	3.9	ALA	302	-0.2	GLU	486	11.3
SER	119	5.2	THR	303	-0.2	MET	487	0.2	SER	119.0	5.6	THR	303	-0.1	MET	487	0.1
ARG	120	-136.8	ILE	304	-0.2	ALA	488	-0.3	ARG	120.0	-145.1	ILE	304	-0.1	ALA	488	-0.3
SER	121	-3.5	TRP	305	-0.6	ALA	489	-0.1	SER	121.0	-3.7	TRP	305	-0.4	ALA	489	-0.1
TYR	122	-2.3	LEU	306	-0.2	GLU	490	12.1	TYR	122.0	-2.6	LEU	306	-0.1	GLU	490	11.0
LEU	123	-3.2	ARG	307	-10.0	LEU	491	-0.3	LEU	123.0	-3.6	ARG	307	-8.9	LEU	491	-0.3
ILE	124	-3.6	GLU	308	10.6	LYS	492	-12.6	ILE	124.0	-3.8	GLU	308	9.8	LYS	492	-11.8
ASP	125	26.0	HIS	309	-0.1	ALA	493	-0.1	ASP	125.0	25.9	HIS	309	-0.1	ALA	493	-0.1
SER	126	-1.3	ASN	310	0.0	LEU	494	0.0	SER	126.0	-1.3	ASN	310	0.0	LEU	494	0.0
PRO	127	0.8	ARG	311	-10.1	TYR	495	-0.1	PRO	127.0	0.8	ARG	311	-9.4	TYR	495	-0.1
PRO	128	-0.7	VAL	312	-0.2	SER	496	0.2	PRO	128.0	-0.7	VAL	312	-0.2	SER	496	0.2
THR	129	-0.8	CYS	313	-0.8	ASP	497	14.8	THR	129.0	-0.9	CYS	313	-0.8	ASP	497	14.0
TYR	130	-0.2	ASP	314	8.7	ILE	498	0.3	TYR	130.0	-0.3	ASP	314	8.1	ILE	498	0.3
ASN	131	-0.3	ILE	315	-0.2	ASP	499	16.7	ASN	131.0	-0.3	ILE	315	-0.1	ASP	499	15.9
VAL	132	-0.5	LEU	316	-0.2	VAL	500	0.3	VAL	132.0	-0.4	LEU	316	-0.2	VAL	500	0.2

HIS	133	-0.2	LYS	317	-7.9	MET	501	0.3	HIS	133.0	-0.1	LYS	317	-7.5	MET	501	0.2
TYR	134	0.4	GLN	318	-0.1	GLU	502	17.1	TYR	134.0	0.4	GLN	318	-0.1	GLU	502	15.1
GLY	135	0.2	GLU	319	8.5	LEU	503	-0.3	GLY	135.0	0.3	GLU	319	8.1	LEU	503	-0.5
TYR	136	0.2	HIS	320	-0.2	TYR	504	0.1	TYR	136.0	0.2	HIS	320	-0.2	TYR	504	-0.1
LYS	137	-17.7	PRO	321	0.0	PRO	505	0.5	LYS	137.0	-17.4	PRO	321	0.0	PRO	505	0.4
SER	138	0.2	GLU	322	7.9	ALA	506	0.2	SER	138.0	0.2	GLU	322	7.6	ALA	506	0.1
TRP	139	0.3	TRP	323	-0.1	LEU	507	-0.8	TRP	139.0	0.3	TRP	323	-0.2	LEU	507	-0.7
GLU	140	13.1	GLY	324	0.1	LEU	508	-0.2	GLU	140.0	12.3	GLY	324	0.1	LEU	508	0.1
ALA	141	0.3	ASP	325	8.8	VAL	509	0.2	ALA	141.0	0.3	ASP	325	8.2	VAL	509	0.3
PHE	142	0.1	GLU	326	9.3	GLU	510	26.8	PHE	142.0	0.1	GLU	326	8.7	GLU	510	25.0
SER	143	-0.1	GLN	327	0.0	LYS	511	-20.1	SER	143.0	-0.2	GLN	327	0.0	LYS	511	-19.4
ASN	144	0.7	LEU	328	0.2	PRO	512	0.4	ASN	144.0	0.6	LEU	328	0.2	PRO	512	0.7
LEU	145	0.3	PHE	329	0.2	ARG	513	-42.9	LEU	145.0	0.2	PHE	329	0.2	ARG	513	-44.5
SER	146	0.4	GLN	330	0.4	PRO	514	0.5	SER	146.0	0.3	GLN	330	0.3	PRO	514	0.4
TYR	147	0.5	THR	331	0.2	ASP	515	21.3	TYR	147.0	0.4	THR	331	0.2	ASP	515	20.2
TYR	148	0.0	SER	332	0.2	ALA	516	-1.1	TYR	148.0	0.1	SER	332	0.2	ALA	516	-1.4
THR	149	-0.4	ARG	333	-11.5	ILE	517	-0.8	THR	149.0	-0.5	ARG	333	-10.5	ILE	517	-0.8
ARG	150	-19.5	LEU	334	0.0	PHE	518	-1.9	ARG	150.0	-17.9	LEU	334	0.0	PHE	518	-2.2
ALA	151	-1.1	ILE	335	0.1	GLY	519	2.2	ALA	151.0	-0.9	ILE	335	0.1	GLY	519	2.9
LEU	152	-0.9	LEU	336	0.1	GLU	520	37.9	LEU	152.0	-0.9	LEU	336	0.1	GLU	520	38.7
PRO	153	0.0	ILE	337	-0.4	THR	521	2.8	PRO	153.0	-0.1	ILE	337	-0.3	THR	521	2.7
PRO	154	-0.2	GLY	338	-0.3	MET	522	3.8	PRO	154.0	-0.1	GLY	338	-0.2	MET	522	1.2
VAL	155	-0.4	GLU	339	11.0	VAL	523	8.5	VAL	155.0	-0.4	GLU	339	10.4	VAL	523	11.6
ALA	156	-0.2	THR	340	-0.6	GLU	524	74.8	ALA	156.0	-0.2	THR	340	-0.5	GLU	524	79.3
ASP	157	12.5	ILE	341	-2.1	LEU	525	7.9	ASP	157.0	11.6	ILE	341	-1.9	LEU	525	7.8
ASP	158	11.7	LYS	342	-11.7	GLY	526	8.9	ASP	158.0	11.0	LYS	342	-11.6	GLY	526	13.2
CYS	159	0.2	ILE	343	0.3	ALA	527	-27.8	CYS	159.0	0.2	ILE	343	0.5	ALA	527	-30.8
PRO	160	0.0	VAL	344	-0.3	PRO	528	-4.9	PRO	160.0	0.0	VAL	344	0.9	PRO	528	-5.2
THR	161	0.3	ILE	345	3.0	PHE	529	-1.5	THR	161.0	0.3	ILE	345	3.4	PHE	529	-0.1
PRO	162	0.4	GLU	346	15.2	SER	530	-74.9	PRO	162.0	0.4	GLU	346	15.7	SER	530	-94.1
MET	163	0.5	ASP	347	18.6	LEU	531	-24.0	MET	163.0	0.6	ASP	347	18.2	LEU	531	-26.2
GLY	164	-0.3	TYR	348	5.3	LYS	532	-37.1	GLY	164.0	-0.3	TYR	348	4.0	LYS	532	-38.9
VAL	165	-0.2	VAL	349	0.7	GLY	533	-4.8	VAL	165.0	-0.2	VAL	349	-1.4	GLY	533	-5.2
LYS	166	-14.2	GLN	350	2.5	LEU	534	0.9	LYS	166.0	-13.5	GLN	350	2.3	LEU	534	0.1
GLY	167	-0.3	HIS	351	2.7	MET	535	-7.1	GLY	167.0	-0.2	HIS	351	3.1	MET	535	-7.8
ASN	168	0.0	LEU	352	-3.0	GLY	536	-1.4	ASN	168.0	0.0	LEU	352	-2.2	GLY	536	-1.7
LYS	169	-10.4	SER	353	-7.0	ASN	537	-0.5	LYS	169.0	-9.8	SER	353	-7.5	ASN	537	-0.5
GLU	170	11.4	GLY	354	-0.8	PRO	538	-0.3	GLU	170.0	10.4	GLY	354	-0.7	PRO	538	-0.3
LEU	171	-0.4	TYR	355	-10.1	ILE	539	0.0	LEU	171.0	-0.4	TYR	355	-10.6	ILE	539	0.0
PRO	172	0.0	HIS	356	-0.4	CYS	540	-1.4	PRO	172.0	0.0	HIS	356	-0.3	CYS	540	-1.5
ASP	173	11.9	PHE	357	-0.6	SER	541	-0.5	ASP	173.0	10.6	PHE	357	-0.4	SER	541	-0.5
SER	174	-0.1	LYS	358	-17.9	PRO	542	-0.5	SER	174.0	-0.1	LYS	358	-18.1	PRO	542	-0.5
LYS	175	-10.2	LEU	359	-5.6	GLN	543	0.0	LYS	175.0	-9.1	LEU	359	-6.2	GLN	543	-0.1
GLU	176	11.5	LYS	360	-18.4	TYR	544	-0.2	GLU	176.0	10.2	LYS	360	-18.8	TYR	544	-0.2
VAL	177	0.0	PHE	361	1.5	TRP	545	-0.2	VAL	177.0	0.0	PHE	361	1.6	TRP	545	-0.2

LEU	178	0.0	ASP	362	14.4	LYS	546	-10.8	LEU	178.0	0.0	ASP	362	14.5	LYS	546	-10.6
GLU	179	11.5	PRO	363	1.1	PRO	547	-0.1	GLU	179.0	10.0	PRO	363	1.1	PRO	547	-0.1
LYS	180	-11.4	GLU	364	15.6	SER	548	0.1	LYS	180.0	-10.2	GLU	364	15.7	SER	548	0.0
VAL	181	0.3	LEU	365	1.1	THR	549	-0.2	VAL	181.0	0.2	LEU	365	1.2	THR	549	-0.2
LEU	182	-0.1	LEU	366	-0.2	PHE	550	-0.1	LEU	182.0	-0.1	LEU	366	-0.2	PHE	550	-0.2
LEU	183	0.2	PHE	367	0.3	GLY	551	-0.1	LEU	183.0	0.2	PHE	367	0.4	GLY	551	-0.1
ARG	184	-14.5	ASN	368	1.0	GLY	552	0.1	ARG	184.0	-11.9	ASN	368	1.0	GLY	552	0.1
ARG	185	-12.5	GLN	369	0.9	GLU	553	10.1	ARG	185.0	-10.9	GLN	369	0.9	GLU	553	9.9
GLU	186	12.6	GLN	370	2.0	VAL	554	0.2	GLU	186.0	11.1	GLN	370	2.1	VAL	554	0.2
PHE	187	0.1	PHE	371	-1.5	GLY	555	0.1	PHE	187.0	0.2	PHE	371	-1.6	GLY	555	0.1
ILE	188	0.4	GLN	372	-0.5	PHE	556	0.1	ILE	188.0	0.2	GLN	372	-0.5	PHE	556	0.1
PRO	189	0.6	TYR	373	0.1	LYS	557	-10.3	PRO	189.0	0.5	TYR	373	0.0	LYS	557	-10.0
ASP	190	22.0	GLN	374	1.2	ILE	558	0.2	ASP	190.0	19.0	GLN	374	1.2	ILE	558	0.2
PRO	191	0.1	ASN	375	0.5	ILE	559	0.1	PRO	191.0	0.1	ASN	375	0.7	ILE	559	0.1
GLN	192	-0.9	ARG	376	-18.1	ASN	560	0.2	GLN	192.0	-1.0	ARG	376	-17.3	ASN	560	0.3
GLY	193	-0.1	ILE	377	0.0	THR	561	0.1	GLY	193.0	-0.1	ILE	377	0.1	THR	561	0.1
SER	194	-0.5	ALA	378	-0.8	ALA	562	0.3	SER	194.0	-0.4	ALA	378	-1.2	ALA	562	0.4
ASN	195	0.6	SER	379	0.8	SER	563	-0.1	ASN	195.0	0.1	SER	379	0.2	SER	563	-0.2
MET	196	0.7	GLU	380	28.0	ILE	564	-0.5	MET	196.0	0.4	GLU	380	24.5	ILE	564	-0.5
MET	197	0.5	PHE	381	-3.1	GLN	565	-0.2	MET	197.0	0.2	PHE	381	-3.2	GLN	565	-0.2
PHE	198	-0.3	ASN	382	-0.3	SER	566	-0.1	PHE	198.0	-0.4	ASN	382	-1.1	SER	566	-0.2
ALA	199	0.2	THR	383	-0.5	LEU	567	-0.3	ALA	199.0	-0.1	THR	383	-1.1	LEU	567	-0.3
PHE	200	0.6	LEU	384	-2.9	ILE	568	-0.5	PHE	200.0	0.3	LEU	384	-3.5	ILE	568	-0.4
PHE	201	0.8	TYR	385	1.4	CYS	569	-0.3	PHE	201.0	0.3	TYR	385	-1.4	CYS	569	-0.2
ALA	202	-0.3	HIS	386	-0.7	ASN	570	-0.3	ALA	202.0	0.0	HIS	386	-0.4	ASN	570	-0.3
GLN	203	0.0	TRP	387	-10.8	ASN	571	-0.3	GLN	203.0	0.1	TRP	387	-0.2	ASN	571	-0.3
HIS	204	0.5	HIS	388	-0.1	VAL	572	-0.2	HIS	204.0	0.4	HIS	388	0.0	VAL	572	-0.1
PHE	205	-0.1	PRO	389	0.6	LYS	573	-8.6	PHE	205.0	0.0	PRO	389	0.5	LYS	573	-7.8
THR	206	-1.5	LEU	390	-0.9	GLY	574	0.1	THR	206.0	-0.4	LEU	390	-0.2	GLY	574	0.1
HIS	207	0.1	LEU	391	-0.3	CYS	575	0.4	HIS	207.0	0.2	LEU	391	0.0	CYS	575	0.4
GLN	208	-0.5	PRO	392	-0.2	PRO	576	0.2	GLN	208.0	-0.1	PRO	392	-0.1	PRO	576	0.2
PHE	209	-0.3	ASP	393	12.5	PHE	577	0.1	PHE	209.0	0.0	ASP	393	10.6	PHE	577	0.0
PHE	210	-1.8	THR	394	0.2	THR	578	0.2	PHE	210.0	-1.1	THR	394	0.2	THR	578	0.2
LYS	211	-15.0	PHE	395	-0.3	SER	579	-0.1	LYS	211.0	-12.9	PHE	395	-0.1	SER	579	-0.2
THR	212	-0.4	ASN	396	0.0	PHE	580	0.5	THR	212.0	-0.2	ASN	396	0.0	PHE	580	0.2
ASP	213	12.7	ILE	397	-0.2	ASN	581	-0.7	ASP	213.0	11.2	ILE	397	-0.2	ASN	581	-0.6
HIS	214	-0.5	GLU	398	9.5	VAL	582	0.0	HIS	214.0	-0.3	GLU	398	8.5	VAL	582	0.1
LYS	215	-11.5	ASP	399	9.2				LYS	215.0	-10.1	ASP	399	8.3			
ARG	216	-12.1	GLN	400	0.3				ARG	216	-10.9	GLN	400	0.2			

Table S8. Electrostatic interaction energies Ees [kJ/mol] for amino acid residues of chain C and D (conformers A) of the COX-2 protein with the MXM.

**Chain C**									**Chain D**								
**AA**	**NO**	**kJ**	**AA**	**NO**	**kJ**	**AA**	**NO**	**kJ**	AA	NO	kJ	AA	NO	kJ	AA	NO	kJ

ASN	34.0	0.2	GLY	217.0	0.5	GLN	400	0.4	ASN	34	0.2	GLY	217	0.7	GLN	400	0.7
PRO	35.0	0.4	PRO	218	0.8	GLU	401	14.1	PRO	35	0.7	PRO	218	1.2	GLU	401	24.9
CYS	36.0	0.6	GLY	219	0.4	TYR	402	0.0	CYS	36	1.0	GLY	219	0.5	TYR	402	0.1
CYS	37.0	0.5	PHE	220	0.5	SER	403	0.2	CYS	37	0.8	PHE	220	1.0	SER	403	0.4
SER	38.0	0.7	THR	221	-1.0	PHE	404	-0.3	SER	38	1.1	THR	221	-1.5	PHE	404	-0.7
ASN	39.0	0.4	ARG	222	-18.4	LYS	405	-14.3	ASN	39	0.5	ARG	222	-30.6	LYS	405	-22.3
PRO	40.0	0.5	GLY	223	0.3	GLN	406	0.4	PRO	40	0.8	GLY	223	0.5	GLN	406	0.7
CYS	41.0	0.6	LEU	224	0.8	PHE	407	-0.1	CYS	41	0.9	LEU	224	1.2	PHE	407	-0.4
GLN	42.0	1.2	GLY	225	1.0	LEU	408	-0.4	GLN	42	1.7	GLY	225	1.4	LEU	408	-0.7
ASN	43.0	1.1	HIS	226	2.5	TYR	409	-0.3	ASN	43	1.4	HIS	226	5.0	TYR	409	-0.4
ARG	44.0	-37.3	GLY	227	-0.6	ASN	410	0.0	ARG	44	-57.1	GLY	227	-0.7	ASN	410	0.0
GLY	45.0	-0.2	VAL	228	-1.4	ASN	411	0.1	GLY	45	-0.1	VAL	228	-2.5	ASN	411	0.3
GLU	46.0	23.4	ASP	229	22.7	SER	412	0.1	GLU	46	36.9	ASP	229	38.3	SER	412	0.1
CYS	47.0	-0.5	LEU	230	-0.9	ILE	413	0.0	CYS	47	-0.6	LEU	230	-1.8	ILE	413	0.0
MET	48.0	-0.1	ASN	231	-1.1	LEU	414	-0.3	MET	48	-0.2	ASN	231	-1.8	LEU	414	-0.5
SER	49.0	-0.1	HIS	232	-1.3	LEU	415	-0.1	SER	49	-0.3	HIS	232	-1.3	LEU	415	-0.2
THR	50.0	-0.3	ILE	233	-0.7	GLU	416	12.5	THR	50	-0.5	ILE	233	-1.3	GLU	416	21.7
GLY	51.0	0.1	TYR	234	-0.3	HIS	417	0.3	GLY	51	0.1	TYR	234	-0.5	HIS	417	1.1
PHE	52.0	0.3	GLY	235	0.0	GLY	418	0.3	PHE	52	0.5	GLY	235	0.0	GLY	418	0.5
ASP	53.0	13.4	GLU	236	20.2	LEU	419	0.2	ASP	53	21.3	GLU	236	33.9	LEU	419	0.4
GLN	54.0	0.3	THR	237	-0.1	THR	420	0.2	GLN	54	0.2	THR	237	-0.1	THR	420	0.5
TYR	55.0	-0.2	LEU	238	-0.2	GLN	421	0.7	TYR	55	-0.3	LEU	238	-0.3	GLN	421	1.2
LYS	56.0	-16.8	ASP	239	13.9	PHE	422	0.4	LYS	56	-25.7	ASP	239	22.9	PHE	422	0.8
CYS	57.0	0.3	ARG	240	-16.3	VAL	423	0.2	CYS	57	0.5	ARG	240	-27.3	VAL	423	0.4
ASP	58.0	21.2	GLN	241	-0.7	GLU	424	15.8	ASP	58	32.8	GLN	241	-1.1	GLU	424	28.1
CYS	59.0	0.5	HIS	242	-0.5	SER	425	0.3	CYS	59	0.8	HIS	242	-0.1	SER	425	0.6
THR	60.0	0.8	LYS	243	-13.3	PHE	426	0.6	THR	60	1.1	LYS	243	-22.2	PHE	426	1.1
ARG	61.0	-23.2	LEU	244	-0.2	THR	427	0.4	ARG	61	-36.0	LEU	244	-0.4	THR	427	0.9
THR	62.0	0.5	ARG	245	-14.8	ARG	428	-16.4	THR	62	0.8	ARG	245	-24.9	ARG	428	-28.8
GLY	63.0	-0.3	LEU	246	-0.2	GLN	429	0.7	GLY	63	-0.4	LEU	246	-0.4	GLN	429	1.2
PHE	64.0	-0.9	PHE	247	-0.3	ILE	430	-0.1	PHE	64	-1.3	PHE	247	-0.5	ILE	430	-0.1
TYR	65.0	0.0	LYS	248	-11.4	ALA	431	-0.2	TYR	65	0.2	LYS	248	-19.0	ALA	431	-0.3
GLY	66.0	0.2	ASP	249	12.7	GLY	432	0.6	GLY	66	0.3	ASP	249	21.8	GLY	432	1.5
GLU	67.0	17.0	GLY	250	0.3	ARG	433	-25.1	GLU	67	27.1	GLY	250	0.5	ARG	433	-46.2
ASN	68.0	0.7	LYS	251	-13.1	VAL	434	-0.3	ASN	68	1.1	LYS	251	-22.7	VAL	434	-1.5
CYS	69.0	0.9	LEU	252	-0.4	ALA	435	-0.5	CYS	69	1.4	LEU	252	-0.8	ALA	435	-2.1
THR	70.0	0.1	LYS	253	-12.5	GLY	436	-0.7	THR	70	0.1	LYS	253	-21.2	GLY	436	-1.6
THR	71.0	0.3	TYR	254	-0.2	GLY	437	-0.4	THR	71	0.8	TYR	254	-0.4	GLY	437	-0.9
PRO	72.0	-0.6	GLN	255	-0.2	ARG	438	-17.4	PRO	72	-0.9	GLN	255	-0.3	ARG	438	-30.6
GLU	73.0	22.3	VAL	256	-0.1	ASN	439	-0.6	GLU	73	34.6	VAL	256	-0.2	ASN	439	-1.3
PHE	74.0	0.8	ILE	257	0.0	VAL	440	-0.5	PHE	74	1.2	ILE	257	0.0	VAL	440	-1.0
LEU	75.0	0.7	GLY	258	0.1	PRO	441	-0.3	LEU	75	1.4	GLY	258	0.3	PRO	441	-0.4
THR	76.0	0.7	GLY	259	0.2	ILE	442	0.0	THR	76	1.1	GLY	259	0.4	ILE	442	0.1
ARG	77.0	-20.1	GLU	260	13.5	ALA	443	0.1	ARG	77	-33.2	GLU	260	23.9	ALA	443	0.1

ILE	78.0	1.3	VAL	261	0.2	VAL	444	0.1	ILE	78	1.8	VAL	261	0.4	VAL	444	0.1
LYS	79.0	-29.1	TYR	262	0.1	GLN	445	0.4	LYS	79	-49.4	TYR	262	0.1	GLN	445	0.7
LEU	80.0	0.1	PRO	263	-0.4	ALA	446	0.4	LEU	80	0.3	PRO	263	-0.8	ALA	446	0.7
LEU	81.0	0.8	PRO	264	0.1	VAL	447	0.3	LEU	81	1.3	PRO	264	0.1	VAL	447	0.6
LEU	82.0	1.8	THR	265	0.1	ALA	448	0.1	LEU	82	2.4	THR	265	0.2	ALA	448	0.1
LYS	83.0	-39.9	VAL	266	-0.2	LYS	449	-15.8	LYS	83	-70.7	VAL	266	-0.3	LYS	449	-26.9
PRO	84.0	1.5	LYS	267	-10.9	ALA	450	0.4	PRO	84	2.1	LYS	267	-18.6	ALA	450	0.8
THR	85.0	3.8	ASP	268	11.8	SER	451	0.4	THR	85	5.6	ASP	268	20.7	SER	451	0.7
PRO	86.0	-0.3	THR	269	0.1	ILE	452	0.1	PRO	86	0.1	THR	269	0.0	ILE	452	-0.1
ASN	87.0	2.4	GLN	270	0.3	ASP	453	17.5	ASN	87	3.7	GLN	270	0.3	ASP	453	29.4
THR	88.0	0.6	VAL	271	-0.1	GLN	454	0.9	THR	88	0.6	VAL	271	-0.2	GLN	454	1.4
VAL	89.0	-0.6	GLU	272	14.3	SER	455	0.2	VAL	89	-0.7	GLU	272	24.5	SER	455	0.2
HIS	90.0	1.5	MET	273	0.3	ARG	456	-18.7	HIS	90	-97.5	MET	273	0.4	ARG	456	-31.4
TYR	91.0	-0.2	ILE	274	-0.2	GLU	457	18.2	TYR	91	-0.5	ILE	274	-0.3	GLU	457	30.3
ILE	92.0	0.1	TYR	275	-0.1	MET	458	0.8	ILE	92	0.4	TYR	275	-0.2	MET	458	0.9
LEU	93.0	-4.7	PRO	276	-0.2	LYS	459	-17.4	LEU	93	-5.8	PRO	276	-0.4	LYS	459	-28.4
THR	94.0	-3.9	PRO	277	-0.1	TYR	460	-0.4	THR	94	-6.4	PRO	277	-0.1	TYR	460	-0.6
HIS	95.0	-0.5	HIS	278	-0.2	GLN	461	0.0	HIS	95	-2.8	HIS	278	0.2	GLN	461	0.9
PHE	96.0	0.4	ILE	279	-0.2	SER	462	1.5	PHE	96	0.7	ILE	279	-0.5	SER	462	2.4
LYS	97.0	-27.4	PRO	280	0.2	LEU	463	1.1	LYS	97	-45.8	PRO	280	0.3	LEU	463	1.6
GLY	98.0	0.1	GLU	281	11.5	ASN	464	1.9	GLY	98	0.1	GLU	281	19.7	ASN	464	3.0
VAL	99.0	0.4	ASN	282	0.4	GLU	465	27.4	VAL	99	0.6	ASN	282	0.3	GLU	465	43.5
TRP	100.0	-1.7	LEU	283	0.3	TYR	466	1.4	TRP	100	-2.3	LEU	283	0.5	TYR	466	2.1
ASN	101.0	0.6	GLN	284	0.2	ARG	467	-33.9	ASN	101	0.5	GLN	284	0.4	ARG	467	-56.8
ILE	102.0	0.4	PHE	285	0.2	LYS	468	-23.9	ILE	102	0.7	PHE	285	0.2	LYS	468	-38.5
VAL	103.0	-0.5	ALA	286	0.2	ARG	469	-37.1	VAL	103	-0.8	ALA	286	0.5	ARG	469	-59.2
ASN	104.0	0.1	VAL	287	0.0	PHE	470	0.6	ASN	104	0.0	VAL	287	-0.1	PHE	470	0.9
ASN	105.0	-0.9	GLY	288	0.0	SER	471	0.1	ASN	105	-1.2	GLY	288	0.0	SER	471	0.3
PRO	106.0	1.2	GLN	289	-1.3	LEU	472	-2.8	PRO	106	1.8	GLN	289	-2.1	LEU	472	-4.5
PHE	107.0	1.6	GLU	290	16.6	LYS	473	-27.4	PHE	107	2.5	GLU	290	28.2	LYS	473	-45.1
LEU	108.0	2.0	VAL	291	-0.1	PRO	474	-0.2	LEU	108	3.3	VAL	291	-0.3	PRO	474	-0.2
ARG	109.0	-32.0	PHE	292	-0.6	TYR	475	-0.8	ARG	109	-53.8	PHE	292	-1.1	TYR	475	-1.5
SER	110.0	1.7	GLY	293	-0.1	THR	476	-0.9	SER	110	3.0	GLY	293	-0.3	THR	476	-1.5
LEU	111.0	3.1	LEU	294	-0.3	SER	477	0.5	LEU	111	4.5	LEU	294	0.1	SER	477	1.0
ILE	112.0	4.7	VAL	295	-0.1	PHE	478	0.5	ILE	112	6.8	VAL	295	-0.2	PHE	478	1.0
MET	113.0	8.3	PRO	296	0.5	GLU	479	18.9	MET	113	10.3	PRO	296	0.8	GLU	479	32.6
LYS	114.0	-29.8	GLY	297	0.4	GLU	480	25.2	LYS	114	-48.7	GLY	297	0.6	GLU	480	43.2
TYR	115.0	5.1	LEU	298	-0.2	LEU	481	0.4	TYR	115	6.4	LEU	298	-0.4	LEU	481	1.1
VAL	116.0	7.3	MET	299	0.2	THR	482	0.3	VAL	116	8.9	MET	299	0.3	THR	482	0.6
LEU	117.0	5.3	MET	300	0.2	GLY	483	-0.5	LEU	117	7.4	MET	300	0.3	GLY	483	-0.9
THR	118.0	5.2	TYR	301	-0.4	GLU	484	18.7	THR	118	3.0	TYR	301	-0.7	GLU	484	33.0
SER	119.0	7.1	ALA	302	-0.3	LYS	485	-17.0	SER	119	8.3	ALA	302	-0.5	LYS	485	-26.5
ARG	120.0	-163.5	THR	303	-0.1	GLU	486	15.9	ARG	120	-231.4	THR	303	-0.2	GLU	486	27.9
SER	121.0	-3.7	ILE	304	-0.2	MET	487	0.2	SER	121	-6.2	ILE	304	-0.3	MET	487	0.5

TYR	122.0	-0.5	TRP	305	-1.0	ALA	488	-0.3	TYR	122	-4.3	TRP	305	-2.0	ALA	488	-0.6
LEU	123.0	0.1	LEU	306	-0.1	ALA	489	-0.1	LEU	123	-7.1	LEU	306	-0.2	ALA	489	-0.2
ILE	124.0	-4.6	ARG	307	-15.5	GLU	490	15.6	ILE	124	-6.5	ARG	307	-26.9	GLU	490	26.9
ASP	125.0	35.5	GLU	308	17.6	LEU	491	-0.4	ASP	125	53.7	GLU	308	31.5	LEU	491	-0.8
SER	126.0	-1.7	HIS	309	-0.1	LYS	492	-16.3	SER	126	-2.4	HIS	309	0.5	LYS	492	-28.6
PRO	127.0	0.8	ASN	310	0.0	ALA	493	-0.1	PRO	127	1.2	ASN	310	0.0	ALA	493	-0.2
PRO	128.0	-1.0	ARG	311	-16.7	LEU	494	-0.1	PRO	128	-1.6	ARG	311	-29.1	LEU	494	-0.1
THR	129.0	-0.5	VAL	312	-0.4	TYR	495	-0.2	THR	129	-0.6	VAL	312	-0.8	TYR	495	-0.2
TYR	130.0	-0.2	CYS	313	-1.4	SER	496	0.2	TYR	130	-0.2	CYS	313	-2.5	SER	496	0.4
ASN	131.0	-0.5	ASP	314	13.5	ASP	497	18.8	ASN	131	-0.9	ASP	314	23.2	ASP	497	31.2
VAL	132.0	-0.6	ILE	315	-0.3	ILE	498	0.4	VAL	132	-1.0	ILE	315	-0.5	ILE	498	0.6
HIS	133.0	-0.3	LEU	316	-0.5	ASP	499	21.2	HIS	133	0.3	LEU	316	-0.8	ASP	499	35.1
TYR	134.0	0.4	LYS	317	-12.5	VAL	500	0.4	TYR	134	0.8	LYS	317	-20.8	VAL	500	0.7
GLY	135.0	0.2	GLN	318	-0.2	MET	501	0.4	GLY	135	0.4	GLN	318	-0.1	MET	501	0.4
TYR	136.0	0.1	GLU	319	13.5	GLU	502	21.3	TYR	136	0.2	GLU	319	23.5	GLU	502	36.9
LYS	137.0	-24.4	HIS	320	-0.5	LEU	503	-0.7	LYS	137	-37.3	HIP	320	-9.3	LEU	503	-0.2
SER	138.0	0.3	PRO	321	0.0	TYR	504	-0.1	SER	138	0.4	PRO	321	-0.1	TYR	504	0.0
TRP	139.0	0.4	GLU	322	12.5	PRO	505	0.5	TRP	139	0.6	GLU	322	21.5	PRO	505	1.2
GLU	140.0	18.4	TRP	323	-0.3	ALA	506	0.3	GLU	140	29.8	TRP	323	-0.5	ALA	506	1.1
ALA	141.0	0.6	GLY	324	0.2	LEU	507	-0.8	ALA	141	0.9	GLY	324	0.3	LEU	507	-0.7
PHE	142.0	1.7	ASP	325	13.6	LEU	508	-0.3	PHE	142	2.5	ASP	325	23.1	LEU	508	-0.3
SER	143.0	-0.1	GLU	326	14.3	VAL	509	0.2	SER	143	-0.2	GLU	326	24.9	VAL	509	0.5
ASN	144.0	0.9	GLN	327	0.1	GLU	510	30.9	ASN	144	1.6	GLN	327	0.1	GLU	510	58.9
LEU	145.0	0.4	LEU	328	0.5	LYS	511	-24.7	LEU	145	0.7	LEU	328	0.9	LYS	511	-40.8
SER	146.0	0.5	PHE	329	0.4	PRO	512	0.8	SER	146	0.8	PHE	329	0.7	PRO	512	0.6
TYR	147.0	0.8	GLN	330	0.8	ARG	513	-50.8	TYR	147	1.2	GLN	330	1.5	ARG	513	-90.6
TYR	148.0	0.1	THR	331	0.4	PRO	514	0.5	TYR	148	0.0	THR	331	0.8	PRO	514	1.4
THR	149.0	-0.8	SER	332	0.5	ASP	515	26.7	THR	149	-1.0	SER	332	1.0	ASP	515	47.4
ARG	150.0	-26.3	ARG	333	-18.7	ALA	516	-1.4	ARG	150	-41.8	ARG	333	-31.5	ALA	516	-2.0
ALA	151.0	-1.3	LEU	334	0.5	ILE	517	-1.3	ALA	151	-2.0	LEU	334	1.2	ILE	517	-1.5
LEU	152.0	-1.1	ILE	335	0.7	PHE	518	-2.7	LEU	152	-1.5	ILE	335	1.5	PHE	518	-5.2
PRO	153.0	-0.1	LEU	336	0.8	GLY	519	1.7	PRO	153	0.1	LEU	336	1.8	GLY	519	5.5
PRO	154.0	-0.2	ILE	337	0.5	GLU	520	45.2	PRO	154	-0.4	ILE	337	1.6	GLU	520	78.0
VAL	155.0	-0.5	GLY	338	0.3	THR	521	2.6	VAL	155	-0.7	GLY	338	1.2	THR	521	5.6
ALA	156.0	-0.3	GLU	339	20.9	MET	522	0.7	ALA	156	-0.4	GLU	339	39.3	MET	522	4.7
ASP	157.0	16.5	THR	340	0.4	VAL	523	7.9	ASP	157	26.7	THR	340	2.2	VAL	523	13.3
ASP	158.0	15.4	ILE	341	-0.8	GLU	524	90.9	ASP	158	24.8	ILE	341	1.2	GLU	524	137.8
CYS	159.0	0.3	LYS	342	-23.6	LEU	525	9.7	CYS	159	0.5	LYS	342	-43.4	LEU	525	10.6
PRO	160.0	0.0	ILE	343	1.6	GLY	526	15.4	PRO	160	0.1	ILE	343	4.6	GLY	526	14.8
THR	161.0	0.4	VAL	344	1.4	ALA	527	-19.5	THR	161	0.6	VAL	344	6.7	ALA	527	-30.3
PRO	162.0	0.6	ILE	345	1.7	PRO	528	-4.0	PRO	162	0.9	ILE	345	3.8	PRO	528	-4.4
MET	163.0	0.8	GLU	346	30.1	PHE	529	1.7	MET	163	1.1	GLU	346	56.8	PHE	529	2.5
GLY	164.0	-0.4	ASP	347	30.8	SER	530	-92.4	GLY	164	-0.6	ASP	347	57.9	SER	530	-79.0
VAL	165.0	-0.3	TYR	348	1.9	LEU	531	-35.7	VAL	165	-0.5	TYR	348	4.0	LEU	531	-44.0

LYS	166.0	-19.6	VAL	349	-4.6	LYS	532	-54.7	LYS	166	-32.0	VAL	349	-11.2	LYS	532	-79.5
GLY	167.0	-0.3	GLN	350	3.1	GLY	533	-5.8	GLY	167	-0.5	GLN	350	3.2	GLY	533	-7.2
ASN	168.0	0.0	HIS	351	2.2	LEU	534	-8.0	ASN	168	-0.1	HIS	351	4.6	LEU	534	-5.6
LYS	169.0	-13.8	LEU	352	-4.2	MET	535	-8.8	LYS	169	-22.2	LEU	352	-2.5	MET	535	-9.3
GLU	170.0	14.6	SER	353	-8.1	GLY	536	-2.8	GLU	170	25.4	SER	353	-12.9	GLY	536	-3.6
LEU	171.0	-0.5	GLY	354	-0.6	ASN	537	-1.5	LEU	171	-0.8	GLY	354	-2.6	ASN	537	-2.6
PRO	172.0	0.0	TYR	355	-14.5	PRO	538	-0.2	PRO	172	0.0	TYR	355	-17.4	PRO	538	-0.2
ASP	173.0	15.2	HIS	356	-0.2	ILE	539	0.0	ASP	173	25.8	HIS	356	1.8	ILE	539	0.0
SER	174.0	-0.1	PHE	357	-0.8	CYS	540	-2.7	SER	174	-0.1	PHE	357	0.4	CYS	540	-4.1
LYS	175.0	-13.9	LYS	358	-27.8	SER	541	-0.9	LYS	175	-23.6	LYS	358	-51.4	SER	541	-1.5
GLU	176.0	14.9	LEU	359	-8.5	PRO	542	-0.7	GLU	176	25.8	LEU	359	-13.0	PRO	542	-1.0
VAL	177.0	0.0	LYS	360	-32.0	GLN	543	0.0	VAL	177	0.2	LYS	360	-51.5	GLN	543	0.0
LEU	178.0	-0.1	PHE	361	0.8	TYR	544	-0.3	LEU	178	0.1	PHE	361	-0.2	TYR	544	-0.5
GLU	179.0	14.9	ASP	362	27.1	TRP	545	-0.6	GLU	179	26.1	ASP	362	46.5	TRP	545	-1.2
LYS	180.0	-14.5	PRO	363	1.0	LYS	546	-17.8	LYS	180	-24.8	PRO	363	0.9	LYS	546	-29.8
VAL	181.0	0.3	GLU	364	26.6	PRO	547	-0.1	VAL	181	0.7	GLU	364	40.4	PRO	547	-0.1
LEU	182.0	-0.1	LEU	365	0.7	SER	548	0.1	LEU	182	0.0	LEU	365	0.5	SER	548	0.3
LEU	183.0	0.1	LEU	366	-1.3	THR	549	-0.4	LEU	183	0.4	LEU	366	-2.3	THR	549	-0.7
ARG	184.0	-18.9	PHE	367	0.0	PHE	550	-0.6	ARG	184	-34.1	PHE	367	-0.5	PHE	550	-1.0
ARG	185.0	-16.2	ASN	368	1.6	GLY	551	-0.2	ARG	185	-28.8	ASN	368	2.2	GLY	551	-0.3
GLU	186.0	16.1	GLN	369	0.8	GLY	552	0.2	GLU	186	29.0	GLN	369	0.0	GLY	552	0.4
PHE	187.0	0.0	GLN	370	3.3	GLU	553	17.1	PHE	187	0.0	GLN	370	4.4	GLU	553	29.7
ILE	188.0	0.4	PHE	371	-2.6	VAL	554	0.5	ILE	188	0.9	PHE	371	-3.6	VAL	554	0.9
PRO	189.0	0.7	GLN	372	-0.1	GLY	555	0.5	PRO	189	1.4	GLN	372	-0.2	GLY	555	0.9
ASP	190.0	27.3	TYR	373	0.0	PHE	556	0.2	ASP	190	51.3	TYR	373	0.0	PHE	556	0.6
PRO	191.0	0.4	GLN	374	2.0	LYS	557	-16.0	PRO	191	0.5	GLN	374	2.7	LYS	557	-28.2
GLN	192.0	-1.4	ASN	375	0.2	ILE	558	0.7	GLN	192	-1.7	ASN	375	1.0	ILE	558	1.5
GLY	193.0	0.0	ARG	376	-26.0	ILE	559	0.5	GLY	193	0.0	ARG	376	-41.3	ILE	559	1.2
SER	194.0	-1.0	ILE	377	-0.5	ASN	560	0.8	SER	194	-1.4	ILE	377	-0.8	ASN	560	1.9
ASN	195.0	0.8	ALA	378	-1.3	THR	561	0.1	ASN	195	1.5	ALA	378	-1.8	THR	561	0.3
MET	196.0	0.9	SER	379	0.2	ALA	562	0.6	MET	196	1.8	SER	379	0.5	ALA	562	1.3
MET	197.0	0.6	GLU	380	35.4	SER	563	-0.3	MET	197	1.2	GLU	380	56.6	SER	563	-1.0
PHE	198.0	-0.5	PHE	381	-3.8	ILE	564	-1.1	PHE	198	-1.2	PHE	381	-4.2	ILE	564	-2.4
ALA	199.0	0.3	ASN	382	-0.6	GLN	565	-0.5	ALA	199	0.3	ASN	382	-0.1	GLN	565	-1.1
PHE	200.0	0.8	THR	383	-0.3	SER	566	-0.3	PHE	200	1.4	THR	383	0.0	SER	566	-0.6
PHE	201.0	0.5	LEU	384	-3.2	LEU	567	-0.8	PHE	201	0.4	LEU	384	-3.4	LEU	567	-1.5
ALA	202.0	-0.3	TYR	385	-6.8	ILE	568	-0.8	ALA	202	-0.8	TYR	385	-8.1	ILE	568	-1.5
GLN	203.0	0.2	HIS	386	-0.8	CYS	569	-0.5	GLN	203	0.3	HIS	386	0.5	CYS	569	-1.1
HIS	204.0	1.0	TRP	387	-4.1	ASN	570	-0.7	HIS	204	2.8	TRP	387	-5.6	ASN	570	-1.5
PHE	205.0	-0.8	HIS	388	-0.4	ASN	571	-0.7	PHE	205	-2.1	HIS	388	0.1	ASN	571	-1.3
THR	206.0	-2.0	PRO	389	0.0	VAL	572	-0.3	THR	206	-3.3	PRO	389	-0.2	VAL	572	-0.6
HIS	207.0	0.2	LEU	390	-1.0	LYS	573	-12.7	HIS	207	1.3	LEU	390	-2.1	LYS	573	-22.2
GLN	208.0	-0.8	LEU	391	-0.4	GLY	574	0.2	GLN	208	-1.9	LEU	391	-0.6	GLY	574	0.4
PHE	209.0	-1.1	PRO	392	-0.2	CYS	575	0.7	PHE	209	-2.1	PRO	392	-0.5	CYS	575	1.1

PHE	210.0	-2.3	ASP	393	16.3	PRO	576	0.4	PHE	210	-3.2	ASP	393	28.7	PRO	576	0.7
LYS	211.0	-22.0	THR	394	0.3	PHE	577	0.1	LYS	211	-36.7	THR	394	0.7	PHE	577	0.1
THR	212.0	-0.4	PHE	395	-0.5	THR	578	0.7	THR	212	-0.7	PHE	395	-0.8	THR	578	1.3
ASP	213.0	17.5	ASN	396	-0.1	SER	579	-0.2	ASP	213	28.8	ASN	396	-0.2	SER	579	-0.4
HIS	214.0	-0.7	ILE	397	-0.3	PHE	580	-0.3	HIS	214	-0.4	ILE	397	-0.6	PHE	580	-0.9
LYS	215.0	-16.0	GLU	398	13.5	ASN	581	-1.8	LYS	215	-27.0	GLU	398	23.8	ASN	581	-3.7
ARG	216.0	-17.2	ASP	399	12.8	VAL	582	0.0	ARG	216	-28.1	ASP	399	22.2	VAL	582	-0.1

Table S9. Electrostatic interaction energies Ees [kJ/mol] for amino acid residues of chain A and B of the COX-1 protein with the CEL.

Chain A									Chain B								
**aa**	**NO**	**kJ**	**aa**	**NO**	**kJ**	**aa**	**NO**	**kJ**	**aa**	**NO**	**kJ**	**aa**	**NO**	**kJ**	**aa**	**NO**	**kJ**
VAL	33	-0.1	GLY	217	-0.5	ASP	401	-17.0	VAL	33.0	0.0	GLY	217	-0.1	ASP	401	-3.9
ASN	34	0.1	PRO	218	0.3	TYR	402	0.1	ASN	34.0	0.1	PRO	218	0.1	TYR	402	0.0
PRO	35	-0.4	GLY	219	0.6	SER	403	-0.4	PRO	35.0	0.0	GLY	219	0.3	SER	403	-0.2
CYS	36	-0.3	PHE	220	-1.0	TYR	404	0.5	CYS	36.0	0.0	PHE	220	-0.4	TYR	404	0.1
CYS	37	-0.2	THR	221	0.9	GLU	405	-16.5	CYS	37.0	0.0	THR	221	0.0	GLU	405	-3.7
TYR	38	-0.1	LYS	222	0.6	GLN	406	-0.3	TYR	38.0	0.0	LYS	222	-0.3	GLN	406	-0.1
TYR	39	0.3	ALA	223	-0.1	PHE	407	0.2	TYR	39.0	0.1	ALA	223	0.1	PHE	407	0.1
PRO	40	-0.2	LEU	224	7.4	LEU	408	8.7	PRO	40.0	0.1	LEU	224	1.3	LEU	408	2.2
CYS	41	-0.2	GLY	225	-0.5	PHE	409	0.6	CYS	41.0	0.0	GLY	225	0.1	PHE	409	0.2
GLN	42	0.0	HIS	226	-2.4	ASN	410	0.5	GLN	42.0	-0.1	HIS	226	-0.4	ASN	410	0.2
HIS	43	-0.6	GLY	227	0.0	THR	411	0.0	HIS	43.0	0.1	GLY	227	-0.1	THR	411	0.0
GLN	44	1.0	VAL	228	1.8	SER	412	-0.3	GLN	44.0	-0.2	VAL	228	0.5	SER	412	-0.1
GLY	45	-0.1	ASP	229	-23.6	MET	413	-0.5	GLY	45.0	-0.2	ASP	229	-4.3	MET	413	-0.2
ILE	46	0.5	LEU	230	10.8	LEU	414	7.4	ILE	46.0	0.0	LEU	230	1.8	LEU	414	1.6
CYS	47	0.4	GLY	231	0.6	VAL	415	0.2	CYS	47.0	0.0	GLY	231	0.1	VAL	415	0.1
VAL	48	0.2	HIS	232	1.0	ASP	416	-13.6	VAL	48.0	0.0	HIS	232	0.4	ASP	416	-2.5
ARG	49	11.7	ILE	233	1.1	TYR	417	0.4	ARG	49.0	0.5	ILE	233	0.3	TYR	417	0.1
PHE	50	0.1	TYR	234	0.7	GLY	418	-0.5	PHE	50.0	0.0	TYR	234	0.1	GLY	418	-0.1
GLY	51	-0.1	GLY	235	0.3	VAL	419	-0.4	GLY	51.0	0.0	GLY	235	0.0	VAL	419	-0.2
LEU	52	3.5	ASP	236	-18.7	GLU	420	-15.6	LEU	52.0	0.1	ASP	236	-3.5	GLU	420	-2.6
ASP	53	-9.6	ASN	237	-0.1	ALA	421	-0.5	ASP	53.0	-0.1	ASN	237	0.0	ALA	421	-0.2
ARG	54	9.3	LEU	238	5.7	LEU	422	6.7	ARG	54.0	-0.1	LEU	238	0.7	LEU	422	1.4
TYR	55	0.3	GLU	239	-13.2	VAL	423	-0.5	TYR	55.0	0.1	GLU	239	-1.9	VAL	423	-0.3
GLN	56	-0.5	ARG	240	17.1	ASP	424	-18.6	GLN	56.0	0.0	ARG	240	3.2	ASP	424	-4.0
CYS	57	-0.1	GLN	241	0.6	ALA	425	-0.9	CYS	57.0	0.0	GLN	241	0.2	ALA	425	-0.4
ASP	58	-12.9	TYR	242	0.3	PHE	426	-1.1	ASP	58.0	0.8	TYR	242	0.0	PHE	426	-0.5
CYS	59	0.1	GLN	243	0.2	SER	427	-0.5	CYS	59.0	0.1	GLN	243	0.0	SER	427	-0.3
THR	60	-0.2	LEU	244	6.5	ARG	428	18.6	THR	60.0	0.1	LEU	244	1.1	ARG	428	3.8
ARG	61	12.4	ARG	245	13.8	GLN	429	-1.1	ARG	61.0	-1.6	ARG	245	1.5	GLN	429	-0.5
THR	62	0.2	LEU	246	5.1	PRO	430	-0.3	THR	62.0	0.3	LEU	246	0.6	PRO	430	-0.1
GLY	63	0.2	PHE	247	0.3	ALA	431	1.1	GLY	63.0	0.0	PHE	247	0.1	ALA	431	0.5
TYR	64	0.6	LYS	248	-0.7	GLY	432	-2.5	TYR	64.0	-0.2	LYS	248	-0.1	GLY	432	-1.0

SER	65	-0.1	ASP	249	-11.4	ARG	433	36.7	SER	65.0	0.0	ASP	249	-0.8	ARG	433	8.3
GLY	66	-0.2	GLY	250	-0.4	ILE	434	6.3	GLY	66.0	0.0	GLY	250	-0.1	ILE	434	3.1
PRO	67	-0.3	LYS	251	-0.6	GLY	435	2.6	PRO	67.0	0.0	LYS	251	-0.2	GLY	435	0.2
ASN	68	-0.3	LEU	252	6.5	GLY	436	1.1	ASN	68.0	0.0	LEU	252	0.9	GLY	436	-0.2
CYS	69	-0.3	LYS	253	-0.8	GLY	437	1.6	CYS	69.0	0.0	LYS	253	-0.2	GLY	437	0.6
THR	70	0.2	TYR	254	0.5	ARG	438	19.7	THR	70.0	0.2	TYR	254	0.1	ARG	438	3.0
ILE	71	-0.2	GLN	255	-0.4	ASN	439	1.1	ILE	71.0	0.1	GLN	255	-0.1	ASN	439	0.3
PRO	72	0.3	MET	256	-0.1	ILE	440	1.5	PRO	72.0	-0.1	MET	256	-0.1	ILE	440	0.4
GLU	73	-11.0	LEU	257	5.1	ASP	441	-21.9	GLU	73.0	2.7	LEU	257	0.8	ASP	441	-5.5
ILE	74	-0.1	ASN	258	0.2	HIS	442	-1.5	ILE	74.0	0.2	ASN	258	0.1	HIS	442	-0.4
TRP	75	-0.3	GLY	259	-0.2	HIS	443	-4.5	TRP	75.0	0.3	GLY	259	0.0	HIS	443	0.0
THR	76	0.1	GLU	260	-13.5	ILE	444	0.0	THR	76.0	0.2	GLU	260	-2.0	ILE	444	0.0
TRP	77	-0.1	VAL	261	-0.4	LEU	445	9.0	TRP	77.0	0.3	VAL	261	-0.2	LEU	445	2.1
LEU	78	3.8	TYR	262	0.2	HIS	446	3.0	LEU	78.0	-1.3	TYR	262	0.0	HIS	446	0.0
ARG	79	11.7	PRO	263	0.7	VAL	447	-0.2	ARG	79.0	-7.8	PRO	263	0.2	VAL	447	0.0
THR	80	0.0	PRO	264	-0.2	ALA	448	0.4	THR	80.0	0.2	PRO	264	-0.1	ALA	448	0.3
THR	81	-0.1	SER	265	0.0	VAL	449	-0.1	THR	81.0	0.3	SER	265	0.0	VAL	449	0.0
LEU	82	3.9	VAL	266	0.3	ASP	450	-21.7	LEU	82.0	-1.7	VAL	266	0.1	ASP	450	-5.0
ARG	83	16.0	GLU	267	-11.3	VAL	451	0.8	ARG	83.0	-8.2	GLU	267	-1.6	VAL	451	0.4
PRO	84	-0.7	GLU	268	-12.0	ILE	452	1.0	PRO	84.0	0.2	GLU	268	-1.6	ILE	452	0.3
SER	85	0.1	ALA	269	-0.2	LYS	453	-0.2	SER	85.0	1.5	ALA	269	-0.1	LYS	453	-0.1
PRO	86	1.3	PRO	270	-0.3	GLU	454	-25.9	PRO	86.0	1.8	PRO	270	-0.1	GLU	454	-6.2
SER	87	1.0	VAL	271	0.0	SER	455	0.9	SER	87.0	0.3	VAL	271	0.0	SER	455	0.4
PHE	88	0.5	LEU	272	5.8	ARG	456	20.0	PHE	88.0	1.1	LEU	272	1.0	ARG	456	3.4
ILE	89	1.1	MET	273	0.0	VAL	457	0.3	ILE	89.0	1.7	MET	273	0.0	VAL	457	0.1
HIS	90	-16.3	HIS	274	-0.6	LEU	458	10.1	HIS	90.0	-24.2	HIS	274	-0.2	LEU	458	2.2
PHE	91	0.4	TYR	275	0.4	ARG	459	16.8	PHE	91.0	0.2	TYR	275	0.1	ARG	459	2.3
LEU	92	4.0	PRO	276	0.5	LEU	460	11.8	LEU	92.0	-4.9	PRO	276	0.1	LEU	460	2.5
LEU	93	-0.5	ARG	277	13.1	GLN	461	-1.1	LEU	93.0	-15.5	ARG	277	2.7	GLN	461	-0.2
THR	94	-1.5	GLY	278	0.0	PRO	462	-1.4	THR	94.0	-2.4	GLY	278	0.0	PRO	462	-0.3
HIS	95	-0.8	ILE	279	0.5	PHE	463	0.0	HIS	95.0	-0.7	ILE	279	0.1	PHE	463	0.2
GLY	96	0.2	PRO	280	-0.6	ASN	464	-1.1	GLY	96.0	0.4	PRO	280	-0.2	ASN	464	0.0
ARG	97	13.2	PRO	281	0.3	GLU	465	-19.5	ARG	97.0	-2.7	PRO	281	-0.1	GLU	465	-0.4
TRP	98	0.2	GLN	282	-0.4	TYR	466	0.7	TRP	98.0	0.8	GLN	282	-0.1	TYR	466	0.5
LEU	99	3.7	SER	283	-0.3	ARG	467	25.3	LEU	99.0	-3.0	SER	283	-0.2	ARG	467	-1.8
TRP	100	-0.2	GLN	284	0.3	LYS	468	-1.9	TRP	100.0	-0.6	GLN	284	0.1	LYS	468	0.6
ASP	101	-11.2	MET	285	-0.5	ARG	469	24.3	ASP	101.0	5.6	MET	285	-0.2	ARG	469	0.6
PHE	102	0.1	ALA	286	-0.3	PHE	470	1.3	PHE	102.0	0.4	ALA	286	-0.1	PHE	470	1.4
VAL	103	-0.1	VAL	287	0.3	GLY	471	-0.1	VAL	103.0	0.0	VAL	287	0.1	GLY	471	0.6
ASN	104	0.8	GLY	288	0.0	MET	472	0.1	ASN	104.0	0.9	GLY	288	0.0	MET	472	0.3
ALA	105	0.1	GLN	289	1.0	LYS	473	-1.0	ALA	105.0	-0.1	GLN	289	0.4	LYS	473	0.0
THR	106	0.1	GLU	290	-20.6	PRO	474	0.4	THR	106.0	0.2	GLU	290	-4.8	PRO	474	0.2
PHE	107	0.1	VAL	291	-0.8	TYR	475	1.6	PHE	107.0	1.1	VAL	291	-0.3	TYR	475	0.0
ILE	108	0.6	PHE	292	0.6	THR	476	-0.1	ILE	108.0	1.8	PHE	292	0.2	THR	476	-0.3

ARG	109	10.3	GLY	293	0.1	SER	477	-0.6	ARG	109.0	-12.3	GLY	293	0.0	SER	477	0.0
ASP	110	-8.7	LEU	294	7.6	PHE	478	-0.3	ASP	110.0	12.9	LEU	294	1.7	PHE	478	0.2
THR	111	0.3	LEU	295	9.9	GLN	479	-0.3	THR	111.0	1.3	LEU	295	2.7	GLN	479	0.3
LEU	112	3.4	PRO	296	-0.6	GLU	480	-16.8	LEU	112.0	-4.6	PRO	296	-0.2	GLU	480	2.4
MET	113	2.9	GLY	297	-0.3	LEU	481	8.8	MET	113.0	5.5	GLY	297	0.0	LEU	481	0.3
ARG	114	9.3	LEU	298	11.8	THR	482	0.1	ARG	114.0	-11.6	LEU	298	3.4	THR	482	0.4
LEU	115	2.9	MET	299	0.4	GLY	483	0.3	LEU	115.0	-6.0	MET	299	0.2	GLY	483	-0.1
VAL	116	-9.9	LEU	300	8.9	GLU	484	-17.2	VAL	116.0	-10.0	LEU	300	2.2	GLU	484	-0.8
LEU	117	1.5	TYR	301	0.7	LYS	485	-0.6	LEU	117.0	-12.6	TYR	301	0.2	LYS	485	0.1
THR	118	-1.2	ALA	302	0.7	GLU	486	-14.6	THR	118.0	-0.4	ALA	302	0.3	GLU	486	-1.2
VAL	119	-2.3	THR	303	0.2	MET	487	0.0	VAL	119.0	-1.4	THR	303	0.2	MET	487	-0.1
ARG	120	19.7	ILE	304	0.8	ALA	488	0.3	ARG	120.0	-31.8	ILE	304	0.4	ALA	488	-0.1
SER	121	0.2	TRP	305	1.9	ALA	489	-0.1	SER	121.0	-2.2	TRP	305	0.7	ALA	489	-0.1
ASN	122	-1.0	LEU	306	7.1	GLU	490	-15.9	ASN	122.0	-2.0	LEU	306	1.3	GLU	490	-1.7
LEU	123	10.0	ARG	307	16.0	LEU	491	7.8	LEU	123.0	-3.1	ARG	307	2.6	LEU	491	0.7
ILE	124	2.5	GLU	308	-16.8	GLU	492	-14.8	ILE	124.0	0.5	GLU	308	-1.4	GLU	492	-0.5
PRO	125	-0.2	HIP	309	3.2	GLU	493	-13.4	PRO	125.0	-0.1	HIP	309	0.2	GLU	493	-1.3
SER	126	0.8	ASN	310	0.2	LEU	494	6.6	SER	126.0	0.0	ASN	310	0.2	LEU	494	0.8
PRO	127	-0.6	ARG	311	15.5	TYR	495	0.9	PRO	127.0	-0.1	ARG	311	1.2	TYR	495	0.1
PRO	128	0.9	VAL	312	0.6	GLY	496	0.1	PRO	128.0	0.3	VAL	312	0.1	GLY	496	0.1
THR	129	1.2	CYS	313	1.4	ASP	497	-15.5	THR	129.0	0.1	CYS	313	0.1	ASP	497	-0.5
TYR	130	0.2	ASP	314	-12.0	ILE	498	-0.5	TYR	130.0	-0.2	ASP	314	-0.8	ILE	498	-0.2
ASN	131	0.4	LEU	315	5.5	ASP	499	-16.7	ASN	131.0	0.1	LEU	315	0.2	ASP	499	-0.3
ILE	132	0.9	LEU	316	5.5	ALA	500	-0.6	ILE	132.0	0.3	LEU	316	0.2	ALA	500	-0.2
ALA	133	0.4	LYS	317	0.0	LEU	501	8.4	ALA	133.0	0.2	LYS	317	0.2	LEU	501	0.6
HIS	134	-1.1	ALA	318	0.2	GLU	502	-23.4	HIS	134.0	-0.1	ALA	318	0.1	GLU	502	-3.8
ASP	135	-16.9	GLU	319	-10.3	PHE	503	-0.3	ASP	135.0	-1.7	GLU	319	0.3	PHE	503	0.1
TYR	136	0.2	HIS	320	4.7	TYR	504	-0.6	TYR	136.0	0.0	HIS	320	0.9	TYR	504	0.0
ILE	137	0.5	PRO	321	0.0	PRO	505	-0.6	ILE	137.0	0.0	PRO	321	0.0	PRO	505	0.2
SER	138	-0.3	THR	322	-0.2	GLY	506	-0.6	SER	138.0	0.0	THR	322	-0.1	GLY	506	0.4
TRP	139	-0.5	TRP	323	0.1	LEU	507	19.7	TRP	139.0	-0.1	TRP	323	-0.1	LEU	507	5.1
GLU	140	-15.0	GLY	324	-0.2	LEU	508	17.1	GLU	140.0	-1.6	GLY	324	0.0	LEU	508	4.3
SER	141	-0.7	ASP	325	-12.1	LEU	509	10.8	SER	141.0	-0.2	ASP	325	-1.0	LEU	509	1.9
PHE	142	-0.3	GLU	326	-12.7	GLU	510	-29.1	PHE	142.0	-0.2	GLU	326	-1.0	GLU	510	3.8
SER	143	-0.4	GLN	327	-0.4	LYS	511	-2.4	SER	143.0	-0.2	GLN	327	0.0	LYS	511	0.0
ASN	144	-0.3	LEU	328	4.3	CYS	512	1.3	ASN	144.0	-0.1	LEU	328	0.1	CYS	512	-2.9
VAL	145	-0.7	PHE	329	-0.3	HIS	513	-4.6	VAL	145.0	-0.3	PHE	329	0.0	HIS	513	-0.1
SER	146	-0.6	GLN	330	0.0	PRO	514	-3.2	SER	146.0	-0.2	GLN	330	0.1	PRO	514	-2.2
TYR	147	-0.6	THR	331	-0.5	ASN	515	-7.2	TYR	147.0	-0.3	THR	331	0.0	ASN	515	-7.8
TYR	148	0.4	ALA	332	-0.7	SER	516	-7.0	TYR	148.0	0.1	ALA	332	-0.1	SER	516	-6.4
THR	149	-0.5	ARG	333	17.6	ILE	517	-0.1	THR	149.0	-0.2	ARG	333	2.2	ILE	517	9.0
ARG	150	23.4	LEU	334	6.0	PHE	518	9.3	ARG	150.0	3.9	LEU	334	0.3	PHE	518	-21.2
ILE	151	2.3	ILE	335	-0.8	GLY	519	1.0	ILE	151.0	0.7	ILE	335	-0.1	GLY	519	5.3
LEU	152	10.1	LEU	336	6.7	GLU	520	-27.1	LEU	152.0	0.8	LEU	336	0.7	GLU	520	8.1

PRO	153	-0.6	ILE	337	-0.7	SER	521	-5.8	PRO	153.0	-0.3	ILE	337	0.2	SER	521	-3.3
SER	154	0.5	GLY	338	-0.6	MET	522	-32.9	SER	154.0	0.2	GLY	338	0.2	MET	522	-25.5
VAL	155	0.5	GLU	339	-20.8	ILE	523	-49.6	VAL	155.0	0.1	GLU	339	-1.3	ILE	523	-70.8
PRO	156	0.1	THR	340	-0.9	GLU	524	-34.7	PRO	156.0	-0.1	THR	340	0.3	GLU	524	9.2
ARG	157	13.9	ILE	341	0.1	MET	525	-3.1	ARG	157.0	1.6	ILE	341	1.2	MET	525	-2.7
ASP	158	-12.7	LYS	342	-2.3	GLY	526	1.0	ASP	158.0	-0.9	LYS	342	-0.2	GLY	526	0.2
CYS	159	-0.1	ILE	343	-2.6	ALA	527	2.3	CYS	159.0	0.0	ILE	343	-0.4	ALA	527	-3.9
PRO	160	-0.2	VAL	344	-2.5	PRO	528	-0.9	PRO	160.0	-0.1	VAL	344	0.9	PRO	528	-1.3
THR	161	-0.2	ILE	345	1.3	PHE	529	1.7	THR	161.0	-0.1	ILE	345	2.2	PHE	529	1.3
PRO	162	-0.4	GLU	346	-18.7	SER	530	18.0	PRO	162.0	0.0	GLU	346	7.6	SER	530	9.9
MET	163	-0.2	GLU	347	-30.4	LEU	531	14.8	MET	163.0	0.1	GLU	347	-3.3	LEU	531	-13.3
GLY	164	0.4	TYR	348	0.6	LYS	532	-3.6	GLY	164.0	0.1	TYR	348	-3.9	LYS	532	0.4
THR	165	0.0	VAL	349	0.3	GLY	533	2.6	THR	165.0	0.0	VAL	349	-3.3	GLY	533	0.9
LYS	166	-0.9	GLN	350	-3.1	LEU	534	21.3	LYS	166.0	-0.5	GLN	350	-4.4	LEU	534	2.3
GLY	167	0.3	GLN	351	-6.0	LEU	535	9.2	GLY	167.0	0.0	GLN	351	-5.4	LEU	535	-4.9
LYS	168	-0.5	LEU	352	-125.4	GLY	536	1.2	LYS	168.0	0.0	LEU	352	-50.7	GLY	536	-0.2
LYS	169	-1.2	SER	353	-20.3	ASN	537	2.8	LYS	169.0	-0.3	SER	353	-38.7	ASN	537	1.0
GLN	170	-0.6	GLY	354	6.0	PRO	538	0.6	GLN	170.0	-0.1	GLY	354	4.9	PRO	538	0.4
LEU	171	7.1	TYR	355	-9.2	ILE	539	1.0	LEU	171.0	0.9	TYR	355	-27.3	ILE	539	0.5
PRO	172	-0.1	PHE	356	0.6	CYS	540	2.3	PRO	172.0	-0.1	PHE	356	1.2	CYS	540	-0.1
ASP	173	-17.0	LEU	357	-0.6	SER	541	0.7	ASP	173.0	-2.9	LEU	357	-11.9	SER	541	0.0
ALA	174	-0.3	GLN	358	2.3	PRO	542	0.2	ALA	174.0	-0.1	GLN	358	3.6	PRO	542	-0.2
GLU	175	-17.7	LEU	359	-14.0	GLU	543	-12.3	GLU	175.0	-3.3	LEU	359	-37.0	GLU	543	1.3
PHE	176	-0.6	LYS	360	-0.4	TYR	544	0.3	PHE	176.0	-0.2	LYS	360	3.8	TYR	544	0.0
LEU	177	7.5	PHE	361	0.8	TRP	545	0.4	LEU	177.0	1.1	PHE	361	-0.3	TRP	545	-0.2
SER	178	-0.1	ASP	362	-12.7	LYS	546	-0.4	SER	178.0	0.0	ASP	362	8.1	LYS	546	0.1
ARG	179	16.9	PRO	363	0.7	ALA	547	-0.1	ARG	179.0	3.1	PRO	363	0.4	ALA	547	-0.2
ARG	180	16.2	GLU	364	-11.6	SER	548	-0.3	ARG	180.0	2.4	GLU	364	5.7	SER	548	-0.2
PHE	181	-1.2	LEU	365	4.2	THR	549	0.0	PHE	181.0	-0.4	LEU	365	-5.8	THR	549	-0.2
LEU	182	10.6	LEU	366	6.0	PHE	550	0.4	LEU	182.0	2.1	LEU	366	-4.9	PHE	550	-0.1
LEU	183	7.8	PHE	367	0.3	GLY	551	0.1	LEU	183.0	1.6	PHE	367	-0.2	GLY	551	0.0
ARG	184	27.8	GLY	368	0.0	GLY	552	-0.2	ARG	184.0	7.5	GLY	368	-0.1	GLY	552	0.0
ARG	185	18.7	ALA	369	-0.2	GLU	553	-11.9	ARG	185.0	3.2	ALA	369	-0.5	GLU	553	1.7
LYS	186	-0.5	GLN	370	-0.5	VAL	554	-0.5	LYS	186.0	-0.2	GLN	370	-0.2	VAL	554	0.0
PHE	187	0.2	PHE	371	0.5	GLY	555	-0.4	PHE	187.0	0.2	PHE	371	-0.4	GLY	555	0.1
ILE	188	-1.7	GLN	372	-1.0	PHE	556	-0.2	ILE	188.0	-1.0	GLN	372	-0.2	PHE	556	0.1
PRO	189	-1.9	TYR	373	-0.1	ASN	557	-0.6	PRO	189.0	-1.0	TYR	373	-0.4	ASN	557	0.2
ASP	190	-61.9	ARG	374	17.5	LEU	558	5.2	ASP	190.0	-31.2	ARG	374	0.3	LEU	558	0.0
PRO	191	0.4	ASN	375	0.9	VAL	559	-0.4	PRO	191.0	-0.2	ASN	375	0.3	VAL	559	0.3
GLN	192	-35.5	ARG	376	21.3	LYS	560	-1.4	GLN	192.0	9.3	ARG	376	2.4	LYS	560	0.1
GLY	193	0.5	ILE	377	1.3	THR	561	-0.5	GLY	193.0	0.7	ILE	377	0.5	THR	561	-0.4
THR	194	5.6	ALA	378	0.0	ALA	562	-0.4	THR	194.0	3.4	ALA	378	-0.4	ALA	562	0.1
ASN	195	-3.5	MET	379	-0.9	THR	563	-0.3	ASN	195.0	0.2	MET	379	-0.5	THR	563	-0.3
LEU	196	11.5	GLU	380	-37.4	LEU	564	12.3	LEU	196.0	3.6	GLU	380	-9.5	LEU	564	2.2

MET	197	-1.3	PHE	381	1.2	LYS	565	-1.1	MET	197.0	-0.6	PHE	381	0.3	LYS	565	-0.5
PHE	198	1.1	ASN	382	-2.5	LYS	566	-1.1	PHE	198.0	0.4	ASN	382	-1.4	LYS	566	-1.0
ALA	199	0.0	GLN	383	-1.1	LEU	567	9.6	ALA	199.0	0.1	GLN	383	-0.6	LEU	567	1.3
PHE	200	-1.3	LEU	384	28.4	VAL	568	0.9	PHE	200.0	-0.4	LEU	384	10.3	VAL	568	0.2
PHE	201	-1.5	TYR	385	5.7	CYS	569	0.4	PHE	201.0	-0.8	TYR	385	2.6	CYS	569	0.0
ALA	202	1.5	HIS	386	1.9	LEU	570	6.6	ALA	202.0	0.8	HIS	386	0.0	LEU	570	0.4
GLN	203	1.9	TRP	387	6.6	ASN	571	-0.2	GLN	203.0	1.0	TRP	387	-0.6	ASN	571	-0.1
HIS	204	-0.2	HIS	388	0.9	THR	572	0.6	HIS	204.0	0.0	HIS	388	0.1	THR	572	0.2
PHE	205	1.1	PRO	389	0.8	LYS	573	0.1	PHE	205.0	0.5	PRO	389	-0.1	LYS	573	0.0
THR	206	0.1	LEU	390	24.5	THR	574	-0.3	THR	206.0	0.1	LEU	390	9.1	THR	574	0.0
HIS	207	-0.4	MET	391	1.6	CYS	575	-0.5	HIS	207.0	0.1	MET	391	0.7	CYS	575	-0.1
GLN	208	2.8	PRO	392	0.7	PRO	576	-0.1	GLN	208.0	1.3	PRO	392	0.3	PRO	576	0.1
PHE	209	2.0	ASP	393	-21.4	TYR	577	-0.1	PHE	209.0	0.9	ASP	393	-5.4	TYR	577	0.0
PHE	210	3.0	SER	394	-0.9	VAL	578	-0.5	PHE	210.0	1.4	SER	394	-0.4	VAL	578	-0.1
LYS	211	-0.9	PHE	395	0.8	SER	579	-0.7	LYS	211.0	-0.5	PHE	395	0.3	SER	579	-0.4
THR	212	0.7	ARG	396	17.4	PHE	580	-1.9	THR	212.0	0.2	ARG	396	4.9	PHE	580	-1.8
SER	213	0.4	VAL	397	0.4	HIS	581	-1.8	SER	213.0	0.0	VAL	397	0.1	HIS	581	-0.8
GLY	214	0.5	GLY	398	0.3	VAL	582	1.0	GLY	214.0	0.2	GLY	398	0.1	VAL	582	0.6
LYS	215	-0.6	PRO	399	-0.8	PRO	583	-0.2	LYS	215.0	0.2	PRO	399	-0.1	PRO	583	-0.1
MET	216	0.1	GLN	400	-0.7				MET	216.0	0.1	GLN	400	-0.3			

Table S10. Electrostatic interaction energies E_{es} [kJ/mol] for amino acid residues of chain A and B of the COX-2 protein with the CEL.

Chain A									Chain B								
aa	NO	kJ	aa	NO	kJ	aa	NO	kJ	aa	NO	kJ	aa	NO	kJ	aa	NO	kJ
ASN	19	0.1	GLY	203	-0.2	GLU	387	-3.0	ASN	19	0.1	GLY	203	-0.2	GLU	387	-2.8
PRO	20	0.0	PRO	204	-0.3	TYR	388	-0.1	PRO	20	0.0	PRO	204	-0.4	TYR	388	-0.1
CYS	21	0.0	GLY	205	0.0	SER	389	-0.1	CYS	21	0.0	GLY	205	0.0	SER	389	-0.1
CYS	22	0.0	PHE	206	-0.4	PHE	390	0.2	CYS	22	-0.1	PHE	206	-0.4	PHE	390	0.2
SER	23	0.0	THR	207	0.2	LYS	391	2.6	SER	23	0.0	THR	207	0.3	LYS	391	2.5
ASN	24	0.1	ARG	208	3.9	GLN	392	-0.2	ASN	24	0.1	ARG	208	3.9	GLN	392	-0.2
PRO	25	0.1	GLY	209	0.2	PHE	393	0.1	PRO	25	0.1	GLY	209	0.2	PHE	393	0.1
CYS	26	0.0	LEU	210	0.0	LEU	394	0.3	CYS	26	0.0	LEU	210	0.0	LEU	394	0.3
GLN	27	0.2	GLY	211	0.1	TYR	395	0.2	GLN	27	0.2	GLY	211	0.1	TYR	395	0.2
ASN	28	-0.2	HIS	212	-0.3	ASN	396	0.0	ASN	28	0.1	HIS	212	-0.3	ASN	396	0.0
ARG	29	-2.4	GLY	213	-0.1	ASN	397	-0.1	ARG	29	-1.8	GLY	213	0.0	ASN	397	-0.1
GLY	30	-0.2	VAL	214	0.6	SER	398	0.0	GLY	30	-0.2	VAL	214	0.6	SER	398	0.0
GLU	31	0.6	ASP	215	-3.6	ILE	399	0.1	GLU	31	0.4	ASP	215	-3.6	ILE	399	0.1
CYS	32	-0.1	LEU	216	0.2	LEU	400	0.2	CYS	32	-0.1	LEU	216	0.2	LEU	400	0.2
MET	33	0.0	ASN	217	0.0	LEU	401	0.1	MET	33	0.0	ASN	217	0.0	LEU	401	0.1
SER	34	0.0	HIS	218	0.7	GLU	402	-2.0	SER	34	0.0	HIS	218	0.6	GLU	402	-1.9
THR	35	0.0	ILE	219	0.2	HIS	403	0.0	THR	35	0.0	ILE	219	0.2	HIS	403	0.0
GLY	36	0.0	TYR	220	0.0	GLY	404	-0.1	GLY	36	0.0	TYR	220	0.1	GLY	404	-0.1
PHE	37	0.0	GLY	221	0.1	LEU	405	-0.1	PHE	37	0.0	GLY	221	0.1	LEU	405	-0.1

ASP	38	0.0	GLU	222	-3.2	THR	406	-0.2	ASP	38	-0.1	GLU	222	-3.2	THR	406	-0.2
GLN	39	0.0	THR	223	0.0	GLN	407	-0.3	GLN	39	-0.1	THR	223	0.0	GLN	407	-0.3
TYR	40	0.0	LEU	224	0.0	PHE	408	-0.2	TYR	40	0.0	LEU	224	0.0	PHE	408	-0.2
LYS	41	-0.8	ASP	225	-1.5	VAL	409	-0.2	LYS	41	-0.7	ASP	225	-1.5	VAL	409	-0.2
CYS	42	0.0	ARG	226	2.9	GLU	410	-2.9	CYS	42	0.0	ARG	226	2.8	GLU	410	-2.7
ASP	43	1.2	GLN	227	0.1	SER	411	-0.3	ASP	43	1.0	GLN	227	0.2	SER	411	-0.2
CYS	44	0.1	HIS	228	0.0	PHE	412	-0.4	CYS	44	0.1	HIS	228	0.0	PHE	412	-0.4
THR	45	0.1	LYS	229	1.7	THR	413	-0.3	THR	45	0.1	LYS	229	1.7	THR	413	-0.3
ARG	46	-2.0	LEU	230	0.1	ARG	414	3.4	ARG	46	-1.8	LEU	230	0.1	ARG	414	3.1
THR	47	0.3	ARG	231	1.3	GLN	415	-0.4	THR	47	0.3	ARG	231	1.3	GLN	415	-0.4
GLY	48	0.0	LEU	232	0.1	ILE	416	0.0	GLY	48	0.0	LEU	232	0.0	ILE	416	0.0
PHE	49	-0.2	PHE	233	0.1	ALA	417	0.3	PHE	49	-0.2	PHE	233	0.1	ALA	417	0.3
TYR	50	-0.1	LYS	234	0.7	GLY	418	-0.6	TYR	50	0.0	LYS	234	0.7	GLY	418	-0.5
GLY	51	0.0	ASP	235	-0.7	ARG	419	4.9	GLY	51	0.0	ASP	235	-0.7	ARG	419	4.1
GLU	52	0.7	GLY	236	-0.1	VAL	420	1.5	GLU	52	0.6	GLY	236	-0.1	VAL	420	1.3
ASN	53	0.0	LYS	237	0.9	ALA	421	-0.8	ASN	53	0.0	LYS	237	0.8	ALA	421	-0.8
CYS	54	0.1	LEU	238	0.1	GLY	422	-0.3	CYS	54	0.0	LEU	238	0.1	GLY	422	-0.3
THR	55	0.1	LYS	239	1.4	GLY	423	-0.1	THR	55	0.1	LYS	239	1.3	GLY	423	-0.1
THR	56	0.1	TYR	240	0.1	ARG	424	2.1	THR	56	0.1	TYR	240	0.1	ARG	424	2.1
PRO	57	-0.1	GLN	241	0.1	ASN	425	0.0	PRO	57	-0.1	GLN	241	0.1	ASN	425	0.0
GLU	58	2.6	VAL	242	0.0	VAL	426	0.3	GLU	58	2.4	VAL	242	0.0	VAL	426	0.3
PHE	59	0.2	ILE	243	0.0	PRO	427	0.2	PHE	59	0.2	ILE	243	0.0	PRO	427	0.2
LEU	60	0.2	GLY	244	0.0	ILE	428	-0.1	LEU	60	0.2	GLY	244	0.0	ILE	428	-0.1
THR	61	0.2	GLY	245	0.0	ALA	429	-0.1	THR	61	0.2	GLY	245	0.0	ALA	429	-0.1
ARG	62	-2.4	GLU	246	-1.9	VAL	430	0.0	ARG	62	-2.2	GLU	246	-1.8	VAL	430	0.0
ILE	63	0.3	VAL	247	-0.1	GLN	431	-0.1	ILE	63	0.3	VAL	247	-0.1	GLN	431	-0.1
LYS	64	-4.7	TYR	248	0.1	ALA	432	-0.2	LYS	64	-4.4	TYR	248	0.1	ALA	432	-0.2
LEU	65	0.2	PRO	249	0.2	VAL	433	-0.1	LEU	65	0.2	PRO	249	0.1	VAL	433	-0.1
LEU	66	0.3	PRO	250	-0.1	ALA	434	0.1	LEU	66	0.3	PRO	250	-0.1	ALA	434	0.1
LEU	67	0.7	THR	251	0.0	LYS	435	3.0	LEU	67	0.6	THR	251	0.0	LYS	435	3.0
LYS	68	-9.9	VAL	252	0.1	ALA	436	-0.1	LYS	68	-9.1	VAL	252	0.1	ALA	436	-0.1
PRO	69	0.2	LYS	253	1.4	SER	437	-0.1	PRO	69	0.1	LYS	253	1.4	SER	437	-0.1
THR	70	1.7	ASP	254	-1.4	ILE	438	0.1	THR	70	1.5	ASP	254	-1.4	ILE	438	0.1
PRO	71	1.0	THR	255	0.0	ASP	439	-3.3	PRO	71	0.9	THR	255	0.0	ASP	439	-3.4
ASN	72	1.8	GLN	256	0.0	GLN	440	-0.3	ASN	72	1.7	GLN	256	-0.1	GLN	440	-0.3
THR	73	0.2	VAL	257	0.0	SER	441	0.2	THR	73	0.2	VAL	257	0.0	SER	441	0.2
VAL	74	-0.3	GLU	258	-2.4	ARG	442	2.9	VAL	74	-0.2	GLU	258	-2.3	ARG	442	3.0
HIS	75	4.0	MET	259	0.0	GLU	443	-3.1	HIS	75	5.8	MET	259	0.0	GLU	443	-3.2
TYR	76	-1.0	ILE	260	0.0	MET	444	0.2	TYR	76	-0.9	ILE	260	0.0	MET	444	0.2
ILE	77	-0.4	TYR	261	0.0	LYS	445	1.7	ILE	77	-0.3	TYR	261	0.0	LYS	445	1.8
LEU	78	-4.7	PRO	262	0.1	TYR	446	0.1	LEU	78	-4.2	PRO	262	0.1	TYR	446	0.1
THR	79	-0.4	PRO	263	0.0	GLN	447	-0.1	THR	79	-0.2	PRO	263	0.0	GLN	447	-0.2
HIS	80	-0.4	HIS	264	0.1	SER	448	-0.1	HIS	80	-0.3	HIS	264	0.1	SER	448	-0.1
PHE	81	0.1	ILE	265	0.1	LEU	449	0.2	PHE	81	0.1	ILE	265	0.1	LEU	449	0.2

LYS	82	2.3	PRO	266	-0.1	ASN	450	0.3	LYS	82	1.9	PRO	266	0.0	ASN	450	0.2
GLY	83	0.4	GLU	267	-1.7	GLU	451	0.2	GLY	83	0.3	GLU	267	-1.6	GLU	451	-0.2
VAL	84	0.3	ASN	268	-0.1	TYR	452	0.6	VAL	84	0.4	ASN	268	-0.1	TYR	452	0.5
TRP	85	-0.3	LEU	269	-0.1	ARG	453	-3.0	TRP	85	-0.2	LEU	269	-0.1	ARG	453	-2.3
ASN	86	0.1	GLN	270	-0.1	LYS	454	-0.8	ASN	86	0.1	GLN	270	0.0	LYS	454	-0.4
ILE	87	0.4	PHE	271	-0.1	ARG	455	-0.1	ILE	87	0.4	PHE	271	-0.1	ARG	455	0.4
VAL	88	0.0	ALA	272	-0.1	PHE	456	1.3	VAL	88	0.0	ALA	272	-0.1	PHE	456	1.3
ASN	89	0.9	VAL	273	0.0	SER	457	0.2	ASN	89	0.9	VAL	273	0.0	SER	457	0.2
ASN	90	0.0	GLY	274	0.0	LEU	458	-0.5	ASN	90	0.0	GLY	274	-0.1	LEU	458	-0.4
ILE	91	0.5	GLN	275	0.5	LYS	459	-3.7	ILE	91	0.5	GLN	275	0.6	LYS	459	-3.3
PRO	92	0.7	GLU	276	-3.6	PRO	460	0.2	PRO	92	0.7	GLU	276	-3.6	PRO	460	0.2
PHE	93	0.8	VAL	277	0.0	TYR	461	0.2	PHE	93	0.8	VAL	277	0.1	TYR	461	0.2
LEU	94	1.4	PHE	278	0.2	THR	462	-0.3	LEU	94	1.4	PHE	278	0.2	THR	462	-0.2
ARG	95	-9.5	GLY	279	0.0	SER	463	0.0	ARG	95	-9.9	GLY	279	0.0	SER	463	0.0
SER	96	1.0	LEU	280	0.0	PHE	464	0.2	SER	96	1.2	LEU	280	0.0	PHE	464	0.2
LEU	97	1.7	VAL	281	0.1	GLU	465	0.6	LEU	97	1.7	VAL	281	0.1	GLU	465	0.5
ILE	98	3.2	PRO	282	-0.2	GLU	466	2.8	ILE	98	3.2	PRO	282	-0.2	GLU	466	2.4
MET	99	3.1	GLY	283	-0.1	LEU	467	0.4	MET	99	3.2	GLY	283	-0.1	LEU	467	0.4
LYS	100	-8.5	LEU	284	0.4	THR	468	0.3	LYS	100	-8.5	LEU	284	0.4	THR	468	0.3
TYR	101	1.6	MET	285	0.2	GLY	469	-0.2	TYR	101	1.6	MET	285	0.2	GLY	469	-0.2
VAL	102	-2.3	MET	286	0.0	GLU	470	-0.5	VAL	102	-1.9	MET	286	-0.1	GLU	470	-0.6
LEU	103	-1.1	TYR	287	0.5	LYS	471	-0.2	LEU	103	-1.2	TYR	287	0.6	LYS	471	-0.2
THR	104	-0.5	ALA	288	0.3	GLU	472	-1.3	THR	104	-0.6	ALA	288	0.3	GLU	472	-1.2
SER	105	-1.3	THR	289	0.2	MET	473	-0.2	SER	105	-1.3	THR	289	0.2	MET	473	-0.2
ARG	106	-43.3	ILE	290	0.3	ALA	474	-0.1	ARG	106	-40.2	ILE	290	0.3	ALA	474	-0.1
SER	107	-1.6	TRP	291	0.6	ALA	475	-0.1	SER	107	-1.7	TRP	291	0.5	ALA	475	-0.1
TYR	108	-1.6	LEU	292	0.2	GLU	476	-1.3	TYR	108	-1.5	LEU	292	0.2	GLU	476	-1.3
LEU	109	-1.6	ARG	293	2.4	LEU	477	0.1	LEU	109	-1.6	ARG	293	2.2	LEU	477	0.1
ILE	110	0.2	GLU	294	-1.3	LYS	478	0.0	ILE	110	0.2	GLU	294	-1.1	LYS	478	0.1
ASP	111	0.5	HIS	295	0.2	ALA	479	0.0	ASP	111	0.1	HIS	295	0.2	ALA	479	0.0
SER	112	0.0	ASN	296	0.2	LEU	480	0.1	SER	112	0.3	ASN	296	0.2	LEU	480	0.1
PRO	113	-0.1	ARG	297	1.1	TYR	481	0.1	PRO	113	-0.1	ARG	297	0.9	TYR	481	0.1
PRO	114	0.2	VAL	298	0.1	SER	482	0.1	PRO	114	0.3	VAL	298	0.1	SER	482	0.1
THR	115	-0.2	CYS	299	0.1	ASP	483	-0.1	THR	115	-0.2	CYS	299	0.1	ASP	483	-0.3
TYR	116	-0.2	ASP	300	-0.7	ILE	484	-0.2	TYR	116	-0.3	ASP	300	-0.6	ILE	484	-0.2
ASN	117	0.1	ILE	301	0.1	ASP	485	0.2	ASN	117	0.1	ILE	301	0.1	ASP	485	-0.1
VAL	118	0.3	LEU	302	0.0	VAL	486	-0.2	VAL	118	0.3	LEU	302	0.0	VAL	486	-0.2
HIS	119	0.2	LYS	303	0.5	MET	487	-0.2	HIS	119	0.2	LYS	303	0.4	MET	487	-0.2
TYR	120	0.0	GLN	304	0.0	GLU	488	-3.1	TYR	120	0.0	GLN	304	0.0	GLU	488	-3.3
GLY	121	0.1	GLU	305	0.3	LEU	489	-0.1	GLY	121	0.1	GLU	305	0.4	LEU	489	0.0
TYR	122	-0.1	HIS	306	-0.1	TYR	490	-0.2	TYR	122	-0.1	HIS	306	-0.1	TYR	490	-0.2
LYS	123	-0.3	PRO	307	0.0	PRO	491	0.1	LYS	123	-0.1	PRO	307	0.0	PRO	491	0.1
SER	124	0.0	GLU	308	0.0	ALA	492	0.2	SER	124	0.0	GLU	308	0.1	ALA	492	0.2
TRP	125	-0.1	TRP	309	-0.1	LEU	493	1.4	TRP	125	-0.1	TRP	309	-0.1	LEU	493	1.5

GLU	126	-1.3	GLY	310	0.0	LEU	494	1.0	GLU	126	-1.5	GLY	310	0.0	LEU	494	1.0
ALA	127	-0.1	ASP	311	-0.9	VAL	495	0.6	ALA	127	-0.1	ASP	311	-0.8	VAL	495	0.6
PHE	128	-0.2	GLU	312	-0.8	GLU	496	3.7	PHE	128	-0.3	GLU	312	-0.8	GLU	496	3.3
SER	129	-0.1	GLN	313	0.0	LYS	497	-2.1	SER	129	-0.1	GLN	313	0.0	LYS	497	-2.0
ASN	130	-0.3	LEU	314	-0.1	PRO	498	0.8	ASN	130	-0.3	LEU	314	-0.1	PRO	498	0.7
LEU	131	-0.4	PHE	315	0.0	ARG	499	-50.2	LEU	131	-0.4	PHE	315	0.0	ARG	499	-48.4
SER	132	-0.3	GLN	316	0.0	PRO	500	-4.1	SER	132	-0.3	GLN	316	0.0	PRO	500	-3.8
TYR	133	-0.2	THR	317	0.0	ASP	501	-11.5	TYR	133	-0.2	THR	317	0.0	ASP	501	-10.3
TYR	134	0.3	SER	318	-0.1	ALA	502	3.3	TYR	134	0.3	SER	318	-0.1	ALA	502	2.6
THR	135	-0.2	ARG	319	2.0	ILE	503	7.0	THR	135	-0.2	ARG	319	2.0	ILE	503	6.8
ARG	136	3.8	LEU	320	0.0	PHE	504	-6.0	ARG	136	4.1	LEU	320	0.0	PHE	504	-7.2
ALA	137	0.8	ILE	321	-0.1	GLY	505	2.6	ALA	137	0.8	ILE	321	-0.1	GLY	505	2.3
LEU	138	-0.2	LEU	322	-0.1	GLU	506	8.6	LEU	138	-0.1	LEU	322	-0.1	GLU	506	7.5
PRO	139	-0.2	ILE	323	0.1	THR	507	-2.4	PRO	139	-0.2	ILE	323	0.2	THR	507	-2.5
PRO	140	0.2	GLY	324	0.1	MET	508	-12.9	PRO	140	0.2	GLY	324	0.2	MET	508	-12.9
VAL	141	0.0	GLU	325	-1.2	VAL	509	-16.0	VAL	141	0.0	GLU	325	-0.9	VAL	509	-15.5
ALA	142	-0.1	THR	326	-0.1	GLU	510	16.3	ALA	142	-0.1	THR	326	-0.1	GLU	510	13.4
ASP	143	-1.4	ILE	327	0.8	LEU	511	-2.6	ASP	143	-1.5	ILE	327	0.9	LEU	511	-2.8
ASP	144	-0.6	LYS	328	-4.3	GLY	512	0.2	ASP	144	-0.8	LYS	328	-4.7	GLY	512	-0.2
CYS	145	0.0	ILE	329	-0.1	ALA	513	-4.8	CYS	145	0.0	ILE	329	-0.1	ALA	513	-5.5
PRO	146	-0.1	VAL	330	1.5	PRO	514	-1.7	PRO	146	-0.1	VAL	330	1.9	PRO	514	-1.7
THR	147	-0.1	ILE	331	1.9	PHE	515	0.7	THR	147	-0.1	ILE	331	1.6	PHE	515	0.8
PRO	148	0.0	GLU	332	6.4	SER	516	11.1	PRO	148	0.0	GLU	332	7.0	SER	516	12.1
MET	149	0.1	ASP	333	-2.1	LEU	517	-20.2	MET	149	0.1	ASP	333	-1.6	LEU	517	-10.7
GLY	150	0.0	TYR	334	1.6	LYS	518	-2.4	GLY	150	0.1	TYR	334	1.4	LYS	518	-1.7
VAL	151	0.0	VAL	335	-1.7	GLY	519	1.0	VAL	151	0.0	VAL	335	-4.7	GLY	519	1.2
LYS	152	-0.2	GLN	336	-1.6	LEU	520	1.2	LYS	152	0.0	GLN	336	-1.5	LEU	520	1.2
GLY	153	0.0	HIS	337	-6.8	MET	521	-0.4	GLY	153	0.0	HIS	337	-6.7	MET	521	-0.4
ASN	154	0.0	LEU	338	-44.7	GLY	522	-0.2	ASN	154	0.0	LEU	338	-39.4	GLY	522	-0.1
LYS	155	0.6	SER	339	-36.8	ASN	523	1.1	LYS	155	0.7	SER	339	-37.1	ASN	523	1.1
GLU	156	-1.3	GLY	340	6.2	PRO	524	0.4	GLU	156	-1.4	GLY	340	5.7	PRO	524	0.4
LEU	157	0.1	TYR	341	-18.5	ILE	525	0.5	LEU	157	0.1	TYR	341	-19.0	ILE	525	0.5
PRO	158	-0.1	HIS	342	2.0	CYS	526	-0.3	PRO	158	-0.1	HIS	342	1.9	CYS	526	-0.3
ASP	159	-2.1	PHE	343	-0.5	SER	527	0.0	ASP	159	-2.2	PHE	343	-0.5	SER	527	0.0
SER	160	-0.1	LYS	344	-3.0	PRO	528	-0.3	SER	160	0.0	LYS	344	-3.3	PRO	528	-0.2
LYS	161	2.2	LEU	345	-9.3	GLN	529	0.0	LYS	161	2.2	LEU	345	-8.4	GLN	529	0.0
GLU	162	-1.9	LYS	346	-9.3	TYR	530	-0.1	GLU	162	-1.9	LYS	346	-9.4	TYR	530	0.0
VAL	163	-0.1	PHE	347	-0.2	TRP	531	-0.1	VAL	163	-0.1	PHE	347	-0.3	TRP	531	-0.1
LEU	164	0.1	ASP	348	7.8	LYS	532	-1.7	LEU	164	0.1	ASP	348	8.1	LYS	532	-1.8
GLU	165	-2.3	PRO	349	0.4	PRO	533	-0.2	GLU	165	-2.3	PRO	349	0.4	PRO	533	-0.2
LYS	166	1.6	GLU	350	5.3	SER	534	-0.1	LYS	166	1.6	GLU	350	5.4	SER	534	-0.1
VAL	167	-0.2	LEU	351	-0.4	THR	535	-0.2	VAL	167	-0.2	LEU	351	-0.4	THR	535	-0.2
LEU	168	-0.1	LEU	352	-0.9	PHE	536	-0.1	LEU	168	-0.1	LEU	352	-0.9	PHE	536	-0.1
LEU	169	0.0	PHE	353	-0.2	GLY	537	0.0	LEU	169	0.0	PHE	353	-0.2	GLY	537	0.0

ARG	170	5.5	ASN	354	0.4	GLY	538	0.0	ARG	170	5.2	ASN	354	0.4	GLY	538	0.0
ARG	171	2.5	GLN	355	-0.7	GLU	539	1.6	ARG	171	2.4	GLN	355	-0.7	GLU	539	1.7
GLU	172	-3.0	GLN	356	0.7	VAL	540	0.0	GLU	172	-2.8	GLN	356	0.7	VAL	540	0.0
PHE	173	0.1	PHE	357	-0.5	GLY	541	0.1	PHE	173	0.2	PHE	357	-0.5	GLY	541	0.1
ILE	174	-0.6	GLN	358	-0.4	PHE	542	0.0	ILE	174	-0.5	GLN	358	-0.4	PHE	542	0.0
PRO	175	-0.3	TYR	359	-0.5	LYS	543	-1.1	PRO	175	-0.3	TYR	359	-0.5	LYS	543	-1.2
ASP	176	-15.5	GLN	360	-0.3	ILE	544	0.1	ASP	176	-14.4	GLN	360	-0.3	ILE	544	0.1
PRO	177	-1.1	ASN	361	0.6	ILE	545	0.3	PRO	177	-1.1	ASN	361	0.5	ILE	545	0.3
GLN	178	-5.1	ARG	362	1.7	ASN	546	0.5	GLN	178	-5.2	ARG	362	2.0	ASN	546	0.5
GLY	179	0.2	ILE	363	0.5	THR	547	-0.3	GLY	179	0.2	ILE	363	0.4	THR	547	-0.3
SER	180	0.9	ALA	364	-0.8	ALA	548	0.2	SER	180	0.9	ALA	364	-0.8	ALA	548	0.3
ASN	181	-0.9	SER	365	-1.4	SER	549	-0.3	ASN	181	-1.0	SER	365	-1.4	SER	549	-0.5
MET	182	-0.6	GLU	366	-8.9	ILE	550	0.3	MET	182	-0.5	GLU	366	-9.5	ILE	550	0.2
MET	183	-0.5	PHE	367	0.2	GLN	551	0.0	MET	183	-0.5	PHE	367	0.3	GLN	551	-0.1
PHE	184	0.2	ASN	368	-1.7	SER	552	-0.3	PHE	184	0.1	ASN	368	-1.6	SER	552	-0.3
ALA	185	-0.4	THR	369	-1.3	LEU	553	0.1	ALA	185	-0.4	THR	369	-1.2	LEU	553	0.1
PHE	186	-0.5	LEU	370	-1.8	ILE	554	0.3	PHE	186	-0.5	LEU	370	-0.8	ILE	554	0.3
PHE	187	-0.9	TYR	371	-5.2	CYS	555	0.2	PHE	187	-0.8	TYR	371	-3.6	CYS	555	0.2
ALA	188	0.5	HIS	372	0.9	ASN	556	0.0	ALA	188	0.4	HIS	372	0.9	ASN	556	0.0
GLN	189	0.2	TRP	373	-6.8	ASN	557	0.0	GLN	189	0.2	TRP	373	-1.8	ASN	557	0.1
HIS	190	-0.4	HIS	374	0.3	VAL	558	0.2	HIS	190	-0.5	HIS	374	0.7	VAL	558	0.2
PHE	191	0.8	PRO	375	0.0	LYS	559	1.5	PHE	191	0.6	PRO	375	0.0	LYS	559	1.3
THR	192	2.5	LEU	376	1.5	GLY	560	0.0	THR	192	2.5	LEU	376	1.4	GLY	560	0.0
HIS	193	1.2	LEU	377	0.5	CYS	561	0.0	HIS	193	1.2	LEU	377	0.5	CYS	561	0.0
GLN	194	1.2	PRO	378	0.2	PRO	562	0.0	GLN	194	1.2	PRO	378	0.2	PRO	562	0.0
PHE	195	1.2	ASP	379	-4.1	PHE	563	-0.2	PHE	195	1.2	ASP	379	-3.9	PHE	563	-0.2
PHE	196	1.8	THR	380	-0.2	THR	564	-0.2	PHE	196	1.9	THR	380	-0.2	THR	564	-0.1
LYS	197	5.3	PHE	381	0.3	SER	565	-0.7	LYS	197	5.3	PHE	381	0.3	SER	565	-0.7
THR	198	0.3	ASN	382	0.1	PHE	566	-1.0	THR	198	0.3	ASN	382	0.1	PHE	566	-1.1
ASP	199	-3.2	ILE	383	0.2	ASN	567	1.1	ASP	199	-3.3	ILE	383	0.2	ASN	567	1.7
HIS	200	0.5	GLU	384	-2.4	VAL	568	0.3	HIS	200	0.5	GLU	384	-2.2	VAL	568	0.2
LYS	201	2.8	ASP	385	-2.3				LYS	201	2.8	ASP	385	-2.1			
ARG	202	2.5	GLN	386	-0.2				ARG	202	2.5	GLN	386	-0.2			

Table S11. Electrostatic interaction energies E_{es} [kJ/mol] for amino acid residues of chain C and D of the COX-2 protein with the CEL.

Chain C									Chain D								
**aa**	**NO**	**kJ**	**aa**	**NO**	**kJ**	**aa**	**NO**	**kJ**	**aa**	**NO**	**kJ**	**aa**	**NO**	**kJ**	**aa**	**NO**	**kJ**
ASN	19.0	0.1	GLY	203	-0.3	GLU	387	-3.8	ASN	19	0.1	GLY	203	-0.2	GLU	387	-3.0
PRO	20.0	-0.1	PRO	204	-0.4	TYR	388	-0.2	PRO	20	0.0	PRO	204	-0.3	TYR	388	-0.1
CYS	21.0	-0.1	GLY	205	0.1	SER	389	-0.2	CYS	21	0.0	GLY	205	0.0	SER	389	-0.1
CYS	22.0	-0.2	PHE	206	-0.5	PHE	390	0.5	CYS	22	-0.1	PHE	206	-0.4	PHE	390	0.2

SER	23.0	-0.1	THR	207	0.4	LYS	391	4.1	SER	23	0.0	THR	207	0.2	LYS	391	2.6
ASN	24.0	0.2	ARG	208	5.9	GLN	392	-0.2	ASN	24	0.1	ARG	208	3.7	GLN	392	-0.1
PRO	25.0	0.1	GLY	209	0.2	PHE	393	0.2	PRO	25	0.1	GLY	209	0.2	PHE	393	0.1
CYS	26.0	-0.1	LEU	210	0.1	LEU	394	0.5	CYS	26	0.0	LEU	210	0.1	LEU	394	0.3
GLN	27.0	0.1	GLY	211	0.0	TYR	395	0.4	GLN	27	0.2	GLY	211	0.1	TYR	395	0.2
ASN	28.0	0.2	HIS	212	-0.3	ASN	396	0.1	ASN	28	0.2	HIS	212	-0.2	ASN	396	0.0
ARG	29.0	0.5	GLY	213	0.0	ASN	397	0.0	ARG	29	-2.0	GLY	213	-0.1	ASN	397	-0.1
GLY	30.0	-0.2	VAL	214	0.7	SER	398	0.0	GLY	30	-0.2	VAL	214	0.6	SER	398	0.0
GLU	31.0	-1.1	ASP	215	-4.7	ILE	399	0.2	GLU	31	0.4	ASP	215	-3.2	ILE	399	0.1
CYS	32.0	0.0	LEU	216	0.2	LEU	400	0.3	CYS	32	-0.1	LEU	216	0.2	LEU	400	0.2
MET	33.0	0.0	ASN	217	0.0	LEU	401	0.1	MET	33	0.0	ASN	217	0.0	LEU	401	0.1
SER	34.0	0.1	HIS	218	0.8	GLU	402	-2.2	SER	34	0.0	HIS	218	0.6	GLU	402	-2.0
THR	35.0	0.0	ILE	219	0.2	HIS	403	0.0	THR	35	0.0	ILE	219	0.2	HIS	403	0.0
GLY	36.0	0.0	TYR	220	0.1	GLY	404	-0.1	GLY	36	0.0	TYR	220	0.0	GLY	404	-0.1
PHE	37.0	-0.1	GLY	221	0.2	LEU	405	0.0	PHE	37	0.0	GLY	221	0.1	LEU	405	-0.1
ASP	38.0	-1.2	GLU	222	-4.5	THR	406	-0.2	ASP	38	-0.1	GLU	222	-3.0	THR	406	-0.2
GLN	39.0	-0.1	THR	223	0.1	GLN	407	-0.3	GLN	39	-0.1	THR	223	0.0	GLN	407	-0.3
TYR	40.0	0.1	LEU	224	0.0	PHE	408	-0.1	TYR	40	0.0	LEU	224	-0.1	PHE	408	-0.2
LYS	41.0	0.1	ASP	225	-2.2	VAL	409	-0.1	LYS	41	-0.7	ASP	225	-1.4	VAL	409	-0.2
CYS	42.0	0.0	ARG	226	4.0	GLU	410	-1.9	CYS	42	0.0	ARG	226	2.7	GLU	410	-2.8
ASP	43.0	-0.1	GLN	227	0.2	SER	411	-0.3	ASP	43	1.1	GLN	227	0.1	SER	411	-0.3
CYS	44.0	0.1	HIS	228	0.0	PHE	412	-0.2	CYS	44	0.1	HIS	228	0.0	PHE	412	-0.4
THR	45.0	0.1	LYS	229	2.5	THR	413	-0.1	THR	45	0.1	LYS	229	1.6	THR	413	-0.3
ARG	46.0	-1.1	LEU	230	0.2	ARG	414	2.3	ARG	46	-1.9	LEU	230	0.1	ARG	414	3.2
THR	47.0	0.3	ARG	231	1.6	GLN	415	-0.3	THR	47	0.3	ARG	231	1.1	GLN	415	-0.4
GLY	48.0	0.0	LEU	232	0.0	ILE	416	-0.7	GLY	48	0.0	LEU	232	0.0	ILE	416	0.0
PHE	49.0	-0.1	PHE	233	0.1	ALA	417	0.0	PHE	49	-0.2	PHE	233	0.1	ALA	417	0.3
TYR	50.0	-0.1	LYS	234	0.8	GLY	418	-2.2	TYR	50	-0.1	LYS	234	0.6	GLY	418	-0.6
GLY	51.0	-0.1	ASP	235	-0.5	ARG	419	16.5	GLY	51	0.0	ASP	235	-0.6	ARG	419	5.2
GLU	52.0	-0.5	GLY	236	-0.1	VAL	420	4.2	GLU	52	0.6	GLY	236	-0.1	VAL	420	1.4
ASN	53.0	-0.1	LYS	237	0.6	ALA	421	4.4	ASN	53	0.0	LYS	237	0.8	ALA	421	-0.6
CYS	54.0	0.0	LEU	238	0.1	GLY	422	1.4	CYS	54	0.0	LEU	238	0.1	GLY	422	-0.3
THR	55.0	0.2	LYS	239	1.7	GLY	423	1.0	THR	55	0.1	LYS	239	1.2	GLY	423	0.0
THR	56.0	0.1	TYR	240	0.1	ARG	424	7.3	THR	56	0.1	TYR	240	0.0	ARG	424	2.4
PRO	57.0	0.0	GLN	241	0.1	ASN	425	1.2	PRO	57	-0.1	GLN	241	0.1	ASN	425	0.1
GLU	58.0	2.1	VAL	242	0.0	VAL	426	0.9	GLU	58	2.5	VAL	242	0.0	VAL	426	0.3
PHE	59.0	0.2	ILE	243	0.1	PRO	427	0.4	PHE	59	0.2	ILE	243	0.0	PRO	427	0.2
LEU	60.0	0.2	GLY	244	0.0	ILE	428	-0.1	LEU	60	0.2	GLY	244	0.0	ILE	428	-0.1
THR	61.0	0.3	GLY	245	0.0	ALA	429	-0.1	THR	61	0.3	GLY	245	0.0	ALA	429	-0.1
ARG	62.0	-1.9	GLU	246	-1.7	VAL	430	-0.1	ARG	62	-2.3	GLU	246	-1.8	VAL	430	0.0
ILE	63.0	0.4	VAL	247	-0.2	GLN	431	-0.4	ILE	63	0.3	VAL	247	-0.1	GLN	431	-0.2
LYS	64.0	-4.4	TYR	248	0.1	ALA	432	-0.4	LYS	64	-4.5	TYR	248	0.1	ALA	432	-0.2
LEU	65.0	0.3	PRO	249	0.2	VAL	433	-0.3	LEU	65	0.2	PRO	249	0.1	VAL	433	-0.1
LEU	66.0	0.5	PRO	250	-0.1	ALA	434	0.1	LEU	66	0.3	PRO	250	-0.1	ALA	434	0.1

LEU	67.0	0.7	THR	251	0.0	LYS	435	6.4	LEU	67	0.7	THR	251	0.0	LYS	435	3.1
LYS	68.0	-9.0	VAL	252	0.2	ALA	436	-0.3	LYS	68	-9.4	VAL	252	0.1	ALA	436	-0.1
PRO	69.0	-0.3	LYS	253	1.9	SER	437	-0.2	PRO	69	0.2	LYS	253	1.3	SER	437	-0.1
THR	70.0	2.4	ASP	254	-1.8	ILE	438	0.4	THR	70	1.6	ASP	254	-1.4	ILE	438	0.1
PRO	71.0	2.7	THR	255	0.0	ASP	439	-7.1	PRO	71	1.0	THR	255	0.0	ASP	439	-3.5
ASN	72.0	3.3	GLN	256	-0.1	GLN	440	-0.5	ASN	72	1.8	GLN	256	0.0	GLN	440	-0.3
THR	73.0	1.6	VAL	257	-0.1	SER	441	0.4	THR	73	0.2	VAL	257	0.0	SER	441	0.2
VAL	74.0	2.0	GLU	258	-3.5	ARG	442	7.3	VAL	74	-0.3	GLU	258	-2.3	ARG	442	3.2
HIS	75.0	-12.9	MET	259	0.0	GLU	443	-6.1	HIS	75	3.4	MET	259	0.0	GLU	443	-3.2
TYR	76.0	0.4	ILE	260	0.1	MET	444	0.3	TYR	76	-1.0	ILE	260	0.0	MET	444	0.2
ILE	77.0	1.3	TYR	261	0.0	LYS	445	4.4	ILE	77	-0.3	TYR	261	0.0	LYS	445	1.9
LEU	78.0	-2.3	PRO	262	0.2	TYR	446	0.2	LEU	78	-4.4	PRO	262	0.1	TYR	446	0.1
THR	79.0	-4.4	PRO	263	0.1	GLN	447	-0.6	THR	79	-0.3	PRO	263	0.0	GLN	447	-0.2
HIS	80.0	-0.8	HIS	264	0.1	SER	448	-0.4	HIS	80	-0.3	HIS	264	0.1	SER	448	-0.1
PHE	81.0	0.5	ILE	265	0.2	LEU	449	0.4	PHE	81	0.1	ILE	265	0.1	LEU	449	0.2
LYS	82.0	-11.2	PRO	266	0.0	ASN	450	-0.1	LYS	82	2.1	PRO	266	0.0	ASN	450	0.2
GLY	83.0	0.1	GLU	267	-2.2	GLU	451	-3.8	GLY	83	0.3	GLU	267	-1.6	GLU	451	-0.3
VAL	84.0	0.3	ASN	268	-0.1	TYR	452	0.4	VAL	84	0.4	ASN	268	-0.1	TYR	452	0.5
TRP	85.0	-0.9	LEU	269	-0.2	ARG	453	6.4	TRP	85	-0.3	LEU	269	-0.1	ARG	453	-1.8
ASN	86.0	0.1	GLN	270	0.0	LYS	454	2.6	ASN	86	0.1	GLN	270	0.0	LYS	454	-0.3
ILE	87.0	0.3	PHE	271	-0.1	ARG	455	4.1	ILE	87	0.4	PHE	271	0.0	ARG	455	0.4
VAL	88.0	-0.3	ALA	272	-0.1	PHE	456	1.5	VAL	88	0.0	ALA	272	0.0	PHE	456	1.3
ASN	89.0	0.1	VAL	273	0.0	SER	457	0.2	ASN	89	1.0	VAL	273	0.0	SER	457	0.2
ASN	90.0	-0.4	GLY	274	-0.1	LEU	458	0.2	ASN	90	0.0	GLY	274	-0.1	LEU	458	-0.4
ILE	91.0	0.4	GLN	275	0.7	LYS	459	-1.5	ILE	91	0.5	GLN	275	0.5	LYS	459	-3.3
PRO	92.0	0.7	GLU	276	-5.3	PRO	460	0.2	PRO	92	0.7	GLU	276	-3.5	PRO	460	0.2
PHE	93.0	1.0	VAL	277	0.1	TYR	461	0.9	PHE	93	0.8	VAL	277	0.1	TYR	461	0.3
LEU	94.0	1.6	PHE	278	0.3	THR	462	-0.1	LEU	94	1.4	PHE	278	0.2	THR	462	-0.2
ARG	95.0	-15.6	GLY	279	0.0	SER	463	-0.4	ARG	95	-9.9	GLY	279	0.0	SER	463	0.0
SER	96.0	1.2	LEU	280	0.0	PHE	464	-0.2	SER	96	1.1	LEU	280	0.0	PHE	464	0.2
LEU	97.0	1.8	VAL	281	0.3	GLU	465	-2.9	LEU	97	1.7	VAL	281	0.2	GLU	465	0.2
ILE	98.0	2.9	PRO	282	-0.1	GLU	466	-2.4	ILE	98	3.3	PRO	282	-0.2	GLU	466	2.2
MET	99.0	3.1	GLY	283	0.1	LEU	467	-0.4	MET	99	3.5	GLY	283	0.0	LEU	467	0.4
LYS	100.0	-10.6	LEU	284	0.7	THR	468	0.3	LYS	100	-8.8	LEU	284	0.4	THR	468	0.3
TYR	101.0	1.1	MET	285	0.4	GLY	469	0.2	TYR	101	1.7	MET	285	0.2	GLY	469	-0.2
VAL	102.0	-3.0	MET	286	0.0	GLU	470	-5.2	VAL	102	-2.2	MET	286	-0.1	GLU	470	-0.9
LEU	103.0	-1.8	TYR	287	0.8	LYS	471	1.8	LEU	103	-1.2	TYR	287	0.5	LYS	471	0.0
THR	104.0	-0.8	ALA	288	0.5	GLU	472	-4.5	THR	104	-0.4	ALA	288	0.3	GLU	472	-1.5
SER	105.0	-1.9	THR	289	0.3	MET	473	-0.3	SER	105	-1.5	THR	289	0.2	MET	473	-0.2
ARG	106.0	-42.1	ILE	290	0.3	ALA	474	0.1	ARG	106	-42.4	ILE	290	0.3	ALA	474	-0.1
SER	107.0	-2.0	TRP	291	0.6	ALA	475	0.0	SER	107	-1.7	TRP	291	0.5	ALA	475	-0.1
TYR	108.0	-2.0	LEU	292	0.3	GLU	476	-4.6	TYR	108	-1.6	LEU	292	0.2	GLU	476	-1.5
LEU	109.0	-1.7	ARG	293	2.1	LEU	477	0.5	LEU	109	-1.6	ARG	293	2.2	LEU	477	0.1
ILE	110.0	0.5	GLU	294	-0.4	LYS	478	3.0	ILE	110	0.2	GLU	294	-1.0	LYS	478	0.3

ASP	111.0	-2.0	HIS	295	0.2	ALA	479	0.0	ASP	111	0.3	HIS	295	0.2	ALA	479	0.0
SER	112.0	0.5	ASN	296	0.2	LEU	480	0.2	SER	112	0.0	ASN	296	0.2	LEU	480	0.1
PRO	113.0	-0.2	ARG	297	0.1	TYR	481	0.2	PRO	113	-0.1	ARG	297	0.8	TYR	481	0.1
PRO	114.0	0.3	VAL	298	0.1	SER	482	0.1	PRO	114	0.2	VAL	298	0.1	SER	482	0.1
THR	115.0	-0.2	CYS	299	0.1	ASP	483	-3.6	THR	115	-0.2	CYS	299	0.1	ASP	483	-0.5
TYR	116.0	-0.3	ASP	300	-0.2	ILE	484	-0.3	TYR	116	-0.2	ASP	300	-0.5	ILE	484	-0.2
ASN	117.0	0.0	ILE	301	0.0	ASP	485	-3.5	ASN	117	0.1	ILE	301	0.1	ASP	485	-0.2
VAL	118.0	0.4	LEU	302	0.0	VAL	486	-0.5	VAL	118	0.3	LEU	302	0.0	VAL	486	-0.2
HIS	119.0	0.3	LYS	303	0.3	MET	487	-0.1	HIS	119	0.2	LYS	303	0.4	MET	487	-0.2
TYR	120.0	-0.1	GLN	304	0.0	GLU	488	-9.2	TYR	120	0.0	GLN	304	0.0	GLU	488	-3.6
GLY	121.0	0.1	GLU	305	1.0	LEU	489	-0.9	GLY	121	0.1	GLU	305	0.4	LEU	489	-0.1
TYR	122.0	0.0	HIS	306	-0.1	TYR	490	-1.5	TYR	122	-0.1	HIS	306	-0.1	TYR	490	-0.2
LYS	123.0	1.3	PRO	307	-0.1	PRO	491	-1.0	LYS	123	-0.2	PRO	307	0.0	PRO	491	0.0
SER	124.0	0.0	GLU	308	0.3	ALA	492	-1.5	SER	124	0.0	GLU	308	0.2	ALA	492	0.0
TRP	125.0	-0.2	TRP	309	-0.1	LEU	493	-0.7	TRP	125	-0.1	TRP	309	-0.1	LEU	493	1.4
GLU	126.0	-2.8	GLY	310	0.0	LEU	494	0.9	GLU	126	-1.3	GLY	310	0.0	LEU	494	1.0
ALA	127.0	-0.1	ASP	311	-0.9	VAL	495	0.4	ALA	127	-0.1	ASP	311	-0.7	VAL	495	0.6
PHE	128.0	-0.4	GLU	312	-0.9	GLU	496	-16.8	PHE	128	-0.2	GLU	312	-0.7	GLU	496	2.2
SER	129.0	-0.2	GLN	313	0.0	LYS	497	-0.3	SER	129	-0.1	GLN	313	0.0	LYS	497	-1.7
ASN	130.0	-0.4	LEU	314	-0.1	PRO	498	4.8	ASN	130	-0.3	LEU	314	-0.1	PRO	498	0.9
LEU	131.0	-0.5	PHE	315	0.1	ARG	499	-42.1	LEU	131	-0.4	PHE	315	0.0	ARG	499	-49.4
SER	132.0	-0.4	GLN	316	0.0	PRO	500	0.9	SER	132	-0.3	GLN	316	0.0	PRO	500	-4.0
TYR	133.0	-0.3	THR	317	0.0	ASP	501	2.4	TYR	133	-0.2	THR	317	0.0	ASP	501	-11.3
TYR	134.0	0.5	SER	318	0.0	ALA	502	-9.6	TYR	134	0.3	SER	318	-0.1	ALA	502	2.7
THR	135.0	-0.4	ARG	319	2.5	ILE	503	-2.8	THR	135	-0.2	ARG	319	1.7	ILE	503	6.5
ARG	136.0	7.7	LEU	320	0.1	PHE	504	20.0	ARG	136	4.0	LEU	320	0.0	PHE	504	-5.3
ALA	137.0	1.1	ILE	321	-0.1	GLY	505	-1.0	ALA	137	0.8	ILE	321	-0.1	GLY	505	2.1
LEU	138.0	0.0	LEU	322	-0.1	GLU	506	-4.1	LEU	138	-0.1	LEU	322	-0.1	GLU	506	6.6
PRO	139.0	-0.4	ILE	323	0.3	THR	507	-2.6	PRO	139	-0.2	ILE	323	0.2	THR	507	-2.5
PRO	140.0	0.3	GLY	324	0.3	MET	508	-6.4	PRO	140	0.2	GLY	324	0.2	MET	508	-15.6
VAL	141.0	0.2	GLU	325	0.1	VAL	509	-10.8	VAL	141	0.0	GLU	325	-0.7	VAL	509	-16.8
ALA	142.0	-0.1	THR	326	0.1	GLU	510	9.0	ALA	142	-0.1	THR	326	0.0	GLU	510	13.3
ASP	143.0	-3.6	ILE	327	1.3	LEU	511	-1.7	ASP	143	-1.5	ILE	327	1.0	LEU	511	-2.6
ASP	144.0	-2.4	LYS	328	-7.5	GLY	512	3.5	ASP	144	-0.8	LYS	328	-4.8	GLY	512	-0.6
CYS	145.0	0.0	ILE	329	0.4	ALA	513	-2.3	CYS	145	0.0	ILE	329	-0.2	ALA	513	-3.9
PRO	146.0	-0.1	VAL	330	2.7	PRO	514	-1.1	PRO	146	-0.1	VAL	330	1.5	PRO	514	-1.3
THR	147.0	-0.1	ILE	331	3.3	PHE	515	1.9	THR	147	-0.1	ILE	331	1.6	PHE	515	1.2
PRO	148.0	-0.1	GLU	332	12.6	SER	516	12.9	PRO	148	0.0	GLU	332	6.9	SER	516	10.8
MET	149.0	0.1	ASP	333	5.2	LEU	517	-13.8	MET	149	0.1	ASP	333	-1.7	LEU	517	-12.9
GLY	150.0	0.2	TYR	334	3.6	LYS	518	-0.5	GLY	150	0.1	TYR	334	-1.8	LYS	518	-2.5
VAL	151.0	0.0	VAL	335	3.1	GLY	519	1.4	VAL	151	0.0	VAL	335	-2.7	GLY	519	1.0
LYS	152.0	2.5	GLN	336	2.7	LEU	520	1.1	LYS	152	0.1	GLN	336	-1.5	LEU	520	0.9
GLY	153.0	0.1	HIS	337	4.5	MET	521	-0.3	GLY	153	0.0	HIS	337	-6.8	MET	521	-0.4
ASN	154.0	0.1	LEU	338	13.9	GLY	522	-0.1	ASN	154	0.0	LEU	338	-35.7	GLY	522	-0.2

LYS	155.0	2.3	SER	339	-9.2	ASN	523	1.3	LYS	155	0.7	SER	339	-39.8	ASN	523	1.0
GLU	156.0	-3.7	GLY	340	-0.4	PRO	524	0.5	GLU	156	-1.5	GLY	340	5.8	PRO	524	0.4
LEU	157.0	0.2	TYR	341	-19.6	ILE	525	0.6	LEU	157	0.1	TYR	341	-19.6	ILE	525	0.5
PRO	158.0	-0.1	HIS	342	0.4	CYS	526	-0.3	PRO	158	-0.1	HIS	342	2.0	CYS	526	-0.3
ASP	159.0	-5.4	PHE	343	0.6	SER	527	0.0	ASP	159	-2.3	PHE	343	-0.6	SER	527	0.0
SER	160.0	0.0	LYS	344	-10.7	PRO	528	-0.2	SER	160	0.0	LYS	344	-3.3	PRO	528	-0.2
LYS	161.0	4.8	LEU	345	-10.4	GLN	529	0.1	LYS	161	2.3	LEU	345	-9.6	GLN	529	0.0
GLU	162.0	-5.1	LYS	346	-13.2	TYR	530	0.0	GLU	162	-2.1	LYS	346	-9.6	TYR	530	0.0
VAL	163.0	-0.3	PHE	347	-0.3	TRP	531	-0.2	VAL	163	-0.1	PHE	347	-0.3	TRP	531	-0.2
LEU	164.0	0.0	ASP	348	10.2	LYS	532	-2.3	LEU	164	0.1	ASP	348	8.4	LYS	532	-1.9
GLU	165.0	-5.6	PRO	349	0.4	PRO	533	-0.2	GLU	165	-2.5	PRO	349	0.3	PRO	533	-0.2
LYS	166.0	4.6	GLU	350	6.9	SER	534	-0.1	LYS	166	1.8	GLU	350	5.6	SER	534	-0.1
VAL	167.0	-0.7	LEU	351	-0.7	THR	535	-0.2	VAL	167	-0.2	LEU	351	-0.4	THR	535	-0.2
LEU	168.0	-0.2	LEU	352	-1.0	PHE	536	-0.2	LEU	168	-0.1	LEU	352	-1.0	PHE	536	-0.2
LEU	169.0	-0.1	PHE	353	-0.3	GLY	537	0.0	LEU	169	0.0	PHE	353	-0.2	GLY	537	0.0
ARG	170.0	11.2	ASN	354	0.4	GLY	538	0.1	ARG	170	5.6	ASN	354	0.4	GLY	538	0.0
ARG	171.0	6.7	GLN	355	-0.7	GLU	539	2.8	ARG	171	2.7	GLN	355	-0.7	GLU	539	1.8
GLU	172.0	-5.6	GLN	356	0.6	VAL	540	0.0	GLU	172	-3.1	GLN	356	0.7	VAL	540	0.0
PHE	173.0	0.1	PHE	357	-0.5	GLY	541	0.1	PHE	173	0.1	PHE	357	-0.6	GLY	541	0.1
ILE	174.0	-0.7	GLN	358	-0.5	PHE	542	0.1	ILE	174	-0.6	GLN	358	-0.4	PHE	542	0.0
PRO	175.0	-1.0	TYR	359	-0.6	LYS	543	-2.8	PRO	175	-0.3	TYR	359	-0.5	LYS	543	-1.3
ASP	176.0	-16.2	GLN	360	-0.5	ILE	544	0.2	ASP	176	-15.3	GLN	360	-0.3	ILE	544	0.1
PRO	177.0	2.0	ASN	361	0.5	ILE	545	0.5	PRO	177	-1.0	ASN	361	0.5	ILE	545	0.3
GLN	178.0	-3.7	ARG	362	3.5	ASN	546	0.8	GLN	178	-5.4	ARG	362	1.6	ASN	546	0.5
GLY	179.0	0.2	ILE	363	0.4	THR	547	-0.2	GLY	179	0.2	ILE	363	0.4	THR	547	-0.3
SER	180.0	0.9	ALA	364	-1.1	ALA	548	0.5	SER	180	0.9	ALA	364	-0.8	ALA	548	0.2
ASN	181.0	-2.2	SER	365	-2.0	SER	549	-0.8	ASN	181	-0.9	SER	365	-1.4	SER	549	-0.5
MET	182.0	-0.9	GLU	366	-15.6	ILE	550	0.0	MET	182	-0.5	GLU	366	-9.4	ILE	550	0.3
MET	183.0	-1.0	PHE	367	-0.5	GLN	551	-0.2	MET	183	-0.5	PHE	367	0.2	GLN	551	-0.2
PHE	184.0	0.2	ASN	368	-2.9	SER	552	-0.5	PHE	184	0.2	ASN	368	-1.7	SER	552	-0.2
ALA	185.0	-0.5	THR	369	-2.0	LEU	553	-0.1	ALA	185	-0.3	THR	369	-1.2	LEU	553	0.0
PHE	186.0	-0.6	LEU	370	-3.2	ILE	554	0.2	PHE	186	-0.5	LEU	370	-1.0	ILE	554	0.3
PHE	187.0	-1.3	TYR	371	-5.7	CYS	555	0.1	PHE	187	-0.8	TYR	371	-4.5	CYS	555	0.2
ALA	188.0	0.7	HIS	372	0.1	ASN	556	-0.2	ALA	188	0.5	HIS	372	0.8	ASN	556	-0.1
GLN	189.0	0.5	TRP	373	-5.6	ASN	557	0.0	GLN	189	0.2	TRP	373	-1.8	ASN	557	0.0
HIS	190.0	-0.5	HIS	374	0.0	VAL	558	0.2	HIS	190	-0.4	HIS	374	0.6	VAL	558	0.2
PHE	191.0	0.7	PRO	375	1.1	LYS	559	1.0	PHE	191	0.6	PRO	375	0.1	LYS	559	1.3
THR	192.0	3.0	LEU	376	3.2	GLY	560	0.1	THR	192	2.4	LEU	376	1.4	GLY	560	0.0
HIS	193.0	1.4	LEU	377	0.7	CYS	561	0.3	HIS	193	1.2	LEU	377	0.4	CYS	561	0.0
GLN	194.0	1.3	PRO	378	0.0	PRO	562	0.2	GLN	194	1.2	PRO	378	0.2	PRO	562	0.0
PHE	195.0	1.1	ASP	379	-6.9	PHE	563	-0.5	PHE	195	1.0	ASP	379	-4.1	PHE	563	-0.2
PHE	196.0	2.3	THR	380	-0.2	THR	564	0.2	PHE	196	1.7	THR	380	-0.2	THR	564	-0.1
LYS	197.0	7.3	PHE	381	0.6	SER	565	-0.6	LYS	197	5.0	PHE	381	0.3	SER	565	-0.6
THR	198.0	0.5	ASN	382	0.1	PHE	566	-1.3	THR	198	0.3	ASN	382	0.1	PHE	566	-1.0

ASP	199.0	-5.3	ILE	383	0.3	ASN	567	-0.3	ASP	199	-3.1	ILE	383	0.2	ASN	567	0.7
HIS	200.0	0.6	GLU	384	-2.3	VAL	568	1.0	HIS	200	0.4	GLU	384	-2.3	VAL	568	0.2
LYS	201	4.5	ASP	385	-2.2				LYS	201	2.7	ASP	385	-2.2			
ARG	202	4.2	GLN	386	-0.3				ARG	202	2.4	GLN	386	-0.2			
